# Breaking Boundaries in Histone Modification MS-Based Detection: A Tailored Search Strategy for Unrestricted Identification of Novel Epigenetic Marks

**DOI:** 10.1016/j.mcpro.2025.101080

**Published:** 2025-09-30

**Authors:** Alessandro Vai, Roberta Noberini, Andrea Graziadei, Daniel A. Polasky, Fengchao Yu, Alexey I. Nesvizhskii, Tiziana Bonaldi

**Affiliations:** 1 Department of Experimental Oncology, IEO, European Institute of Oncology IRCSS, Milan, Italy; 2 Human Technopole, Milan, Italy; 3 Department of Pathology, University of Michigan, Ann Arbor, Michigan, USA; 4 Gilbert S. Omenn Department of Computational Medicine and Bioinformatics, University of Michigan, Ann Arbor, Michigan, USA; 5 Department of Oncology and Hematology-Oncology, University of Milan, Milan, Italy

**Keywords:** post-translational modifications, histone PTM discovery, epigenetics, mass spectrometry, bioinformatics workflow

## Abstract

Histone post-translational modifications (PTMs) play a crucial role in regulating gene expression and maintaining DNA integrity, and their aberrations are linked to various diseases, including cancer. While lysine acetylation and methylation have been extensively studied, recent research has uncovered additional PTMs that significantly contribute to chromatin structure and function. Mass spectrometry is the most effective analytical method for studying histone PTMs; however, computational limitations often restrict the analysis to common modifications. Unrestrictive search strategies have the potential to enable a more comprehensive characterization of the histone modification landscape. In this work, we systematically assess the application of unrestrictive search approaches to histone data. After evaluating the limitations of these methods, we develop a novel bioinformatics workflow, named HiP-Frag (histone PTM analysis with FragPipe), which enables the identification of 96 sites decorated with uncommon PTMs on core histones—60 of which were previously unreported—as well as 55 histone marks on linker histones, including 13 novel ones, purified from human cell lines and primary samples. The expanded histone PTM analysis enabled by this strategy is among the first to extract previously unexplored epigenetic information from mass spectrometry raw data. This approach paves the way for a facilitated and more streamlined identification of uncommon and yet unannotated histone modifications, supporting a deeper dissection of the histone code and the understanding of the potential biological role of the novel epigenetic marks.

## Highlights

• Novel histone PTMs have emerged as regulators of chromatin structure.
• HiP-Frag integrates closed, open, and detailed mass offset searches.
• HiP-Frag simplifies the discovery of novel histone modifications.
• HiP-Frag identified 60 novel PTMs on core histones and 13 on linker histones.

## Graphical Abstract

**Figure:**
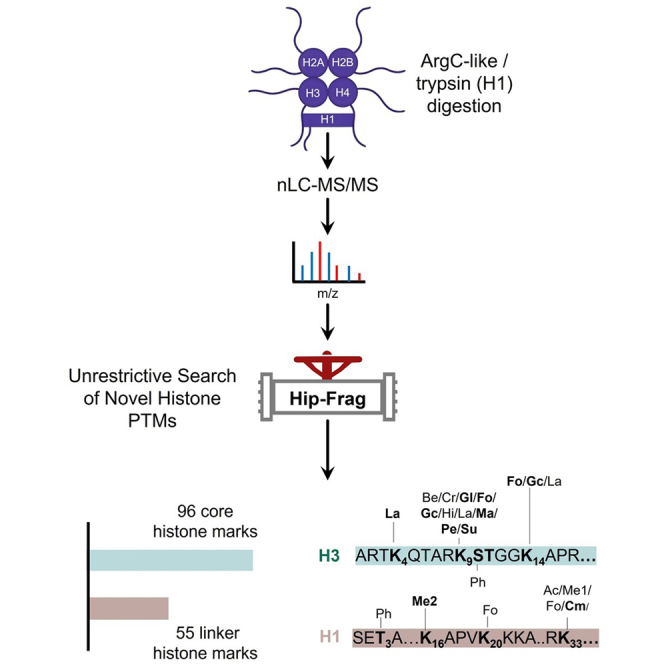

## In Brief

This study introduces and describes HiP-Frag, a bioinformatics workflow that exploits unrestrictive search strategies to expand the analysis of histone post-translational modifications (PTMs) to unknown ones, from mass spectrometry data. By expanding the catalog of histone PTMs with 60 marks on core histones and 13 on linker histones, HiP-Frag enables a more comprehensive investigation of the histone code, giving access to previously unexplored epigenetic information.

Histone post-translational modifications (PTMs) refer to the chemical changes that occur on the amino acid residues of histone proteins upon their synthesis. The chemical groups that can be deposited to histone residues are diverse in nature. Besides the well-known lysine (K) acetylation and methylation, a wide variety of modification classes have been reported, including K-acylations, glutamine monoaminylation (serotonylation and dopaminylation), and glycation (1). Histone PTMs play a crucial role in regulating chromatin structure and function, influencing gene expression, DNA repair, and other nuclear processes (2). Since they regulate fundamental nuclear functions, their dysregulation is often observed in pathological conditions, especially in cancer. In this context, two classes of novel modifications have been shown to play relevant roles: K-acylation and glycations (3, 4). Acylations are a class of PTMs where the epsilon-amino group of Ks is modified by either short-chain acyl moieties—such as propionyl, butyryl, crotonyl—or long-chain moieties—such as hexanoyl, octanoyl, decanoyl, etc. One of the most studied acylation types is histone lactylation, which was shown to have detrimental roles in different cancer types. For instance, histone lactylation has been shown to promote metastatization and progression of clear cell renal cell carcinoma (5) and to contribute to chemoresistance of colorectal cancer stem cells (6) and of hepatocellular carcinoma (7); elevated levels of histone lactylation have also been observed in triple-negative breast cancer tissues (8). Protein glycation consists in the modification of K and arginine (R) residues by the so-called “advanced glycation end products” (AGEs) that include carboxymethyl, carboxyethyl, pyrraline, etc. In cancer, glyoxal and methylglyoxal are toxic byproducts of the enhanced glycolysis driven by the Warburg effect (9). These molecules easily permeate the nucleus and react with histones, introducing AGEs at Ks and Rs, leading to disruption of histone–DNA interactions, interference with nucleosome stability and alteration of chromatin structure, thereby compromising gene expression.

Mass spectrometry (MS) has emerged as the preferred technique for studying protein PTMs, since it enables not only their detection but also precise localization on the polypeptide sequence and the accurate quantification of their abundance. However, the MS-based analysis of histone PTMs remains a challenging task, both from an analytical and computational perspective. The distinct sequences of histones, coupled with the chemical complexity of their modifications, have driven continuous advancements in sample preparation, MS acquisition, and computational analysis workflows specifically tailored for their study (10). Core histones are rich in K and R residues, requiring specialized sample preparation for proteolytic digestion in bottom-up MS workflows. Indeed, trypsin digestion results in histone peptides too short for proper MS analysis. To address this, two alternative digestion methods have been developed: an in-solution ArgC enzyme digestion, which cleaves at R residues, or an “ArgC-like” approach, where K residues are chemically modified prior to the subsequent tryptic activity, so that the enzyme produces an ArgC-like pattern (11). For the derivatization step, either deuterated acetic anhydride (D3 protocol) or propionic anhydride (PRO protocol) can be used. The latter is usually followed by a second derivatization of N termini with either propionic anhydride (PRO2 protocol) or phenyl isocyanate (PRO-PIC protocol) to enhance chromatographic retention.

Histone PTM MS-based profiling typically focuses on core histone proteins, although linker histone H1 proteins are also extensively post-translationally modified (12), with several novel PTMs recently identified. For instance, in breast cancer cell lines, methylglyoxal levels appear to be more abundant on H1 variants than on the core histones (13), highlighting the importance of methods to investigate known and novel modifications also on the linker histones.

While sample preparation strategies for enriching histones from total protein extracts and improving the detection of conventional histone PTMs have undergone continuous refinement and significant implementations (14, 15), data analysis remains a bottleneck in identifying the full repertoire of histone modifications beyond the classical ones. Commonly, peptide identification from MS/MS spectra is performed by a “closed search” approach, where mass spectra are fed into database search engines. In these search engines, an experimental fragmentation spectrum is compared to a list of theoretical spectra derived from an *in-silico* digestion of a protein (modified) sequences database. The comparison yields a list of peptide-to-spectrum matches (PSMs), which are ranked according to a score, and eventually the highest-scoring (modified) peptide is assigned to the spectrum (16). A limitation of this strategy is that the presence of multiple variable modifications to be searched increases exponentially the number of modified forms in which each peptide can virtually exist. As a result, the experimental spectra must be matched against a tremendously large number of theoretical spectra, increasing the odds of obtaining high-scoring spurious matches and thus reducing the sensitivity of the search at a specific false discovery rate (FDR) (17). One solution to mitigate this problem is to adopt the so-called “open modification search” (OS) strategy (18, 19): while in the (closed) database search the allowed MS1 mass difference between experimentally observed precursors and candidate peptides is narrow; in the OS a large mass difference between peptide sequences and experimentally observed precursors is allowed; and the frequency of observing a specific modification mass relative to the unmodified version of a peptide (delta mass) can also be used to assess the likelihood of its presence. Hence, this approach bypasses the need to prespecify which PTMs to search for, since it automatically considers a wide range of possible modifications, based on the measured mass differences. OS strategies have been already used to identify novel histone PTMs (20, 21, 22). However, their primary application has been the identification of novel delta masses, rather than the comprehensive dissection of individual protein microheterogeneity arising from the combinatorial presence of a wide variety of PTMs, as seen in histones. Moreover, not all delta masses reported by the OS represent genuine *in vivo* modifications, since sample preparation methods involve the use of reagents that can potentially introduce chemical adducts on proteins. In addition, the delta mass reported by the OS can correspond to the sum of multiple modifications. Consequently, when multiple delta masses are under investigation, a systematic assessment of the OS results is mandatory to fully characterize the modification(s) and/or artifact(s) associated to each delta mass.

Given these challenges, here we optimized an “unrestrictive search” approach for analyzing MS data from hypermodified histone proteins to enable the accurate and robust identification of a larger number of histone sites bearing less common and less abundant modifications. We optimized and tested a computational workflow based on an unrestrictive search of PTMs on histone MS data and we then applied it to various datasets derived from the MS analysis of core histones extracted from a panel of cancer cell lines and linker histones H1 enriched from breast cancer patient tissues. This study represents the first assessment of an unrestrictive approach applied to histone bottom-up MS RAW data. Upon stringent filtering, we identified 60 previously unreported marks on core histones and 13 on linker histones, delivering an important analytical resource for the comprehensive identification and characterization of histone PTMs.

## Experimental Procedures

### Cell Lines and Patient Tissues

The following human cancer cell lines were analyzed: UM-SCC-6, Panc1, MCF7, MDA-MB-231, HCEC, MCF10A, A2780, SK-OV-3, and NB-4. Growth media are reported in Supplemental Table S1.

Breast cancer tissue specimens were obtained from patients undergoing surgery for the removal of clinically confirmed neoplasia at the European Institute of Oncology (Milan). The patients provided informed consent, and this study was approved by the Ethical Committee of the European Institute of Oncology (Study UID 2550). This study abides by the Declaration of Helsinki principles. Breast cancer subtypes were defined as described (23). Samples were selected to have a tumor cellularity of at least 50%, as assessed by hematoxylin and eosin staining.

### Histone Enrichment From Cell Lines and Fresh-Frozen Tissues

Histones were enriched from cell lines as described (24). For histone H1 analysis, approximately 20 to 70 mg of frozen tissue were thawed on ice, cut in small pieces with scissors, and homogenized in 1 ml of nuclei isolation buffer composed of PBS containing 0.1% Triton X-100 and protease inhibitors (0.5 mM PMSF, 5 μM aprotinin, 5 μM leupeptin, and 5 mM Na-butyrate) using a 1 ml Dounce homogenizer. The homogenate was filtered through a 100 μm cell strainer to remove tissue debris and pipetted several times using a 200 μl pipette tip. Nuclei were then obtained as described (24).

### Histone Derivatization and Proteolytic Digestion

Prior to enzymatic digestion, 3 μg to 5 μg of core histones were mixed with an equal amount of a histone super-stable isotope labeling by amino acids in cell culture (super-SILAC) mix, which had been generated as previously described (25) used as internal standard for relative quantification. Quantification was not performed in this work; however, in this study, we exploited already acquired MS RAW data for histones purified from cells that were employed in other studies, where histone PTMs quantification was the aim. Histones were separated on a 17% PAA SDS-PAGE gel. A gel bands spanning the molecular weight of 10-15 KDa and corresponding to the whole histone octamer (comprising H3, H2A, H2B, and H4) was excised, and the proteins were in-gel digested as previously described (26), using a double derivatization protocol, whereby K residues are first chemically acylated with PRO, then trypsin is used for in gel digestion, and the proteolytic peptide N termini are then derivatized with phenyl isocyanate (PIC). This results in an “ArgC-like” digestion pattern, when derivatization is complete. For histone H1 analysis, 10 μg of proteins were loaded on a 4 to 12% PAA precast SDS-PAGE gel (Invitrogen). A large band around the size of histone H1 variants (20–45 kDa) is excised for standard in-gel digestion with trypsin (27). All histone peptides were eluted from the gel and concentrated desalted on handmade C18 StageTips, prior to liquid chromatography (LC)-MS/MS.

The AQUA peptide H-DAVTYTEHA(K/malonyl)R(13C,15N4)-OH (purity ≥95%, Biosynth) was reconstituted in 5% acetonitrile to a stock concentration of 3.6 nmol/μl. The peptide was derivatized with PIC and loaded on StageTips. 10 pmol were analyzed by MS.

### LC-MS/MS Analysis

Core histone peptide mixtures were separated by reversed-phase chromatography on an EASY-nLC 1200 high-performance liquid chromatography system through an EASY-Spray column (Thermo Fisher Scientific), 25-cm long (inner diameter 75 μm, PepMap C18, 2 μm particles), which was connected online to a Q Exactive HF or a Q Exactive Plus (Thermo Fisher Scientific) instrument through an EASY-Spray Ion Source (Thermo Fisher Scientific). Solvent A was 0.1% formic acid in double-distilled water (ddH2O), and solvent B was 80% CAN plus 0.1% formic acid. Peptides were injected in an aqueous 1% TFA solution at a flow rate of 500 nl/min and were separated with a 50-min linear gradient of 10 to 45% for PRO-PIC digested samples. Linker histone H1 peptides were separated with a 95 min 3%–60% gradient of solvent B (80 min 3–30%, 10 min 30–40%, 5 min 40–60%), at a flow rate of 250 nl/min. Survey full scan MS spectra (m/z 375–1650) were analyzed in the Orbitrap detector with a resolution of 60,000 at m/z 200. The MS instruments were operated in the data-dependent acquisition mode to automatically switch between full-scan MS and MS/MS acquisition. Survey full-scan MS spectra (m/z 300–1350) were analyzed in the Orbitrap detector with a resolution of 60,000 to 70,000 at m/z 200. The 10 to 12 most intense peptide ions with charge states comprised between two and four were sequentially isolated to a target value for MS1 of 3 × 10^6^ and fragmented by HCD with a normalized collision energy setting of 28%. The maximum allowed ion accumulation times were 20 ms for full scans and 80 ms for MS/MS, and the target value for MS/MS was set to 1 × 10^5^. The dynamic exclusion time was set to 10 s, and the standard mass spectrometric conditions for all experiments were as follows: spray voltage of 1.8 kV and no sheath and auxiliary gas flow.

### Search Settings

All searches were performed using MSFragger (version 4.1) (28), using built-in mass calibration. The UniProt reference proteome UP000005640 (release 11/21) containing canonical and isoform protein sequences, as well contaminants sequences added by Philosopher (29), was used for the searches against the full human proteome. For the searches that were run against the histone database only, this database was filtered to retain only histone sequences. Due to the derivatization of Ks in the sample processing, enzyme specificity was set to Arg-C. Alternatively, the enzyme specificity was set to trypsin, to account for the possible presence of tryptic peptides in the sample. The same was done for the analysis of linker histones, which were not derivatized. PeptideProphet (30) was used to compute probabilities used as input for estimating the FDR, which was set to 1% at PSM and peptide level, and to 100% at the protein level, unless proteins identification was the aim, where it was set to 1%. IonQuant (31) was used for protein quantification specifying ‘Top N ions’ equals 6. The maximum number of variable modifications was set to 5, and the ‘max combinations number’ was increased to 99,000. The option ‘use all mods in first search’ was enabled. The minimum peptide length was set to six amino acids for core histones and seven for the linker histones. Fragment mass tolerance was set to 20 ppm. Precursor mass tolerance was left as default in FragPipe (10 ppm for detailed mass offset, −150/+500 Da for OS, and 20 ppm for closed search). Other parameters that were specific to each search, including the list of variable modifications and the mass offsets searched, are reported in Supplemental Table S2. The MSFragger version used in this study did not allow for a mass offset to occur simultaneously on the peptide N terminus and on the first amino acid. To overcome this limitation, a predigested FASTA was supplied to MSFragger, where non-amino acidic letters (B and J) were added at the beginning of peptide sequences, to mimic N-terminal modifications. Then, the masses of N-term variable modifications were set as fixed modifications on the non-amino acidic letters. Only PSMs for which the mass offset was unambiguously localized at exactly one unique position as reported in the columns ‘Number Best Positions’ of the ‘psm.tsv’ table were retained in the results.

### Experimental Design and Statistical Rationale

The focus of this study was discovering novel histone PTMs rather than quantifying differences between samples. Therefore, no statistical procedure, such as power calculations, was employed to compute the sample size; instead, the sample size was chosen based on the size of previously acquired or published datasets. The set of core histones RAW data consists of nine human cancer cell lines, three biological replicates each (Supplemental Table S1). The set of linker histone H1 RAW data consists of 29 fresh-frozen breast cancer samples, 12 belonging to the Luminal A subtype and 17 to the triple negative subtype. All the data downloaded from published studies consist of three biological replicates, unless otherwise stated. These data are described throughout the ‘Results’ section, and all the accession codes are provided under the ‘Data availability’ statement.

## Results

### Implementation of a Search Strategy to Improve the Identification of Uncommon Histone PTMs

**Fig. 1:**
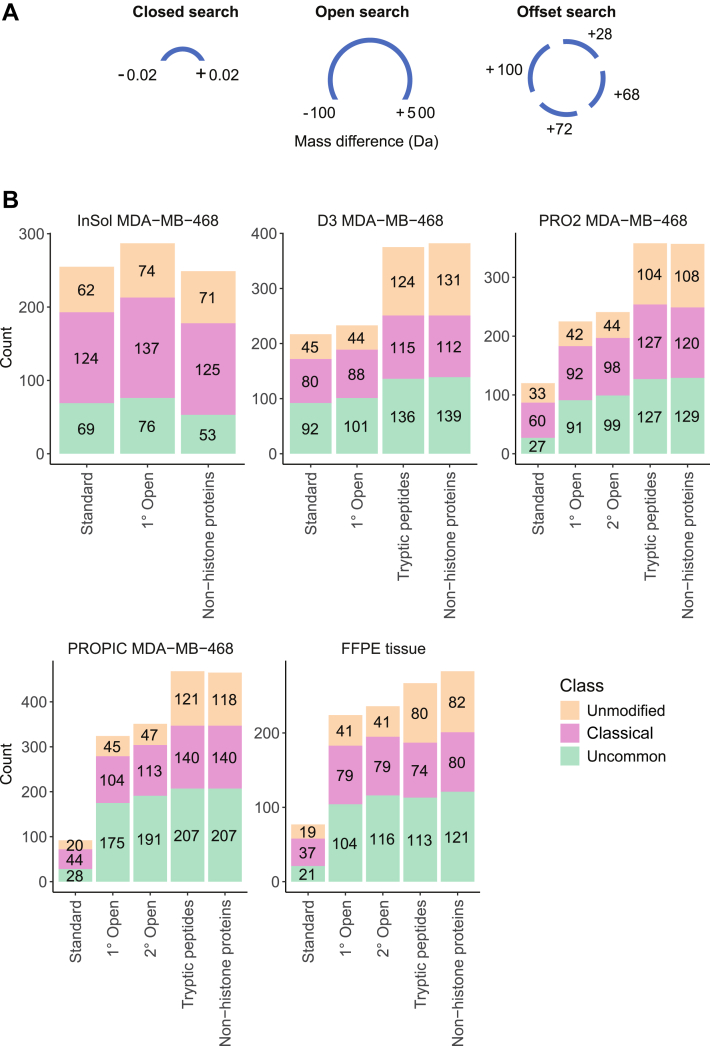
**Implementation of HiP-Frag for the discovery of known and novel histone PTMs.***A*, schema of the search strategies implemented in MSFragger. *B*, bar chart visualization of the number of histone marks identified at each step of the optimization strategy. 1° and 2° Open refers to detailed mass offset performed after including the modifications found after, respectively, the first and second round of open search. Tryptic peptides: same as 2° Open, but the cleavage rule was changed from ArgC to Trypsin. Non-histone proteins: same as ‘Tryptic peptides’ but including the top 100 non-histone proteins with the highest intensity in the FASTA file. Classical modifications: K-acetylation and K-methylation; the complete list of uncommon modifications is reported in Supplemental Table S2. Da, *Dalton*; FFPE, Formalin-Fixed Paraffin-Embedded; HiP-Frag, histone PTMs analysis with FragPipe; PTM, post-translational modification.

We tested MSFragger, one of the most popular tools implementing the open database search concept, on different histone MS datasets. In addition to the OS, MSFragger offers the user an alternative option for PTM discovery: the “mass offset search”. While in OS any mass within a large range is allowed between the precursor and the candidate peptide, in the mass offset search only a list of mass differences specified by the user is allowed (Fig. 1*A*). Another difference between the two search approaches is that the delta mass identified in the OS can be potentially localized on any amino acid, while in the mass offset the user can restrict each mass offset to occur only at specific amino acid(s). This type of offset search is called “detailed mass offset search” (DMO) (32).

We tested both the OS and the DMO on a small dataset consisting of MS RAW data—acquired in a data-dependent acquisition mode on a Q Exactive Plus Orbitrap high-resolution mass spectrometer—of core histones enriched from three replicates of the triple negative breast cancer cell line MDA-MB-231 and processed following the PRO-PIC protocol. The OS produced a long list of delta masses (Supplemental Table S3), which was, however, difficult to interpret, because the delta masses generated could not be associated unambiguously to one specific modification. Indeed, the OS was designed primarily for whole-proteome studies, where modifications are inferred based on the mass difference between the experimentally measured peptide and its theoretical unmodified counterpart. For histones, this assumption does not hold, because the chemical derivatization performed during sample preparation for MS leads to propionylation (pr) of unmodified and mono-methylated Ks. As a result, the delta mass reported by OS often cannot be directly interpreted as a single modification, as it may instead correspond to a combination of various biological and chemical modifications. To illustrate one case where the delta mass requires an in-depth interpretation to dissect the PTMs decorating the peptide, we report a representative example (Supplemental Fig. S1) whereby OS reported the presence of a delta mass of +15.99 Da (oxidation) on the peptide 9 to 17 of histone H3, carrying both a trimethylation (me3) at K9 and a pr at K14 (Kme3STGGKprAPR). The delta mass corresponding to oxidation is localized at K14, which—however—already bears a propionyl group with a mass of +56.02 Da. The sum of the mass of oxidation with that of pr is +72.02 Da, which is isobaric to the mass of a lactylation. Therefore, the true modification is more likely to be a K-lactylation rather than the oxidation and the pr co-occurring on the same residue K14. This issue arises because the OS is designed to minimize the delta mass assigned to a given candidate peptide.

DMO searching greatly reduces the search space compared to OS and simplifies the localization of modifications compared to OS, where any modification can be localized at any site. In cases like the example of peptide 9 to 17 of H3 mentioned above, modification localization can often be ambiguous, because as few as a single pair of fragment ions allowing to distinguish between adjacent potential modification sites may exist in a MS/MS spectrum. This makes the DMO output easier to interpret compared to the OS output. Nonetheless, when performing an initial DMO search on the same dataset, we were able to identify only 30 sites carrying less common PTMs (Supplemental Table S4 and S5). Unlike OS, DMO requires that modifications and artefacts are defined *a priori* to be found by the search; therefore, we hypothesized that the lower number of identifications might be due to the omission of some abundant modifications and/or artifacts in the initial modification set specified for the DMO search. It is known, in fact, that chemical derivatization can introduce byproducts, such as serine and threonine propionylations, or, on the contrary, be incomplete, which leaves “free Ks” that are cleaved by trypsin, leading to the production of fully tryptic histone peptides (33). Both these factors can reduce identifications, especially when extending the analysis to rarer modifications. This consideration prompted us to optimize the DMO by running sequential DMO searches to explore whether accounting for chemical artefacts introduced with the sample preparation, tryptic peptides, and non-histone proteins could increase the identification rate. To assess if the pipeline works independently from the specific sample preparation protocol used for histone digestion prior to LC-MS/MS, we tested these options on histones extracted from the same cell line (MDA-MB-468, triple negative breast cancer) processed in-solution or in-gel with three of the the most common histone derivation protocols (D3, PRO2, and PRO-PIC) (26). With each step of the optimization, DMO identified an increasing number of histone marks (Fig. 1*B*) and PSMs (Supplemental Fig. S2) compared to the previous step, regardless of the specific protocol used, indicating that this workflow is applicable in different experimental settings. The increase in the number of observed modifications at each optimization step can be attributed to a more accurate representation of the sample chemical complexity. This is because the closed and mass offset searches cannot identify spectra of peptides bearing modifications or artifacts that are not included in the search space, so adding commonly observed features results in a higher number of spectra identified. While OS can, by definition, identify unspecified modifications, the large search space that results from considering any modification ultimately reduces its sensitivity. Thus, using OS to determine the most abundant modifications, followed by an optimized mass offset search, results in the largest number of identified spectra (Supplemental Fig. S2).

In the field of MS-analysis of histone marks, it is a common practice to search for histone data against a database that contains only histone sequences. Here, we also tested for the presence of contaminating non-histone proteins to evaluate whether spectra lacking the corresponding protein in the database could be assigned to decoys, potentially reducing identification sensitivity. Because a search carried out considering both biological and chemical modifications potentially occurring on the whole proteome increases exponentially the computing time and would become prohibitive (Supplemental Fig. S3), we performed the searches against a database containing histone sequences plus the addition of the top 100 most abundant non-histone proteins identified by running a search against the whole human proteome and specifying only the chemical modifications produced by the specific sample preparation undertaken. We observed a decrease in identifications in the in-solution dataset, likely due to ArgC digestion being suboptimal for non-histone proteins. This was due to the expansion of the search space to account for peptides that might be too long to be detected by the bottom-up approach. For the other protocols, the inclusion of non-histone proteins in the analysis did not result in a significant increase in identifications, likely because histones extracted from cell lines typically exhibit a higher degree of purity. For this reason, we also repeated the searches on histones extracted from formalin-fixed paraffin-embedded tissues (34) which are characterized by higher protein background signal. As a matter of fact, the number of histone marks detected improved, although slightly (Fig. 1*B*), in these samples.

### Performance Assessment of the Search Framework for High-Confidence Results

**Fig. 2:**
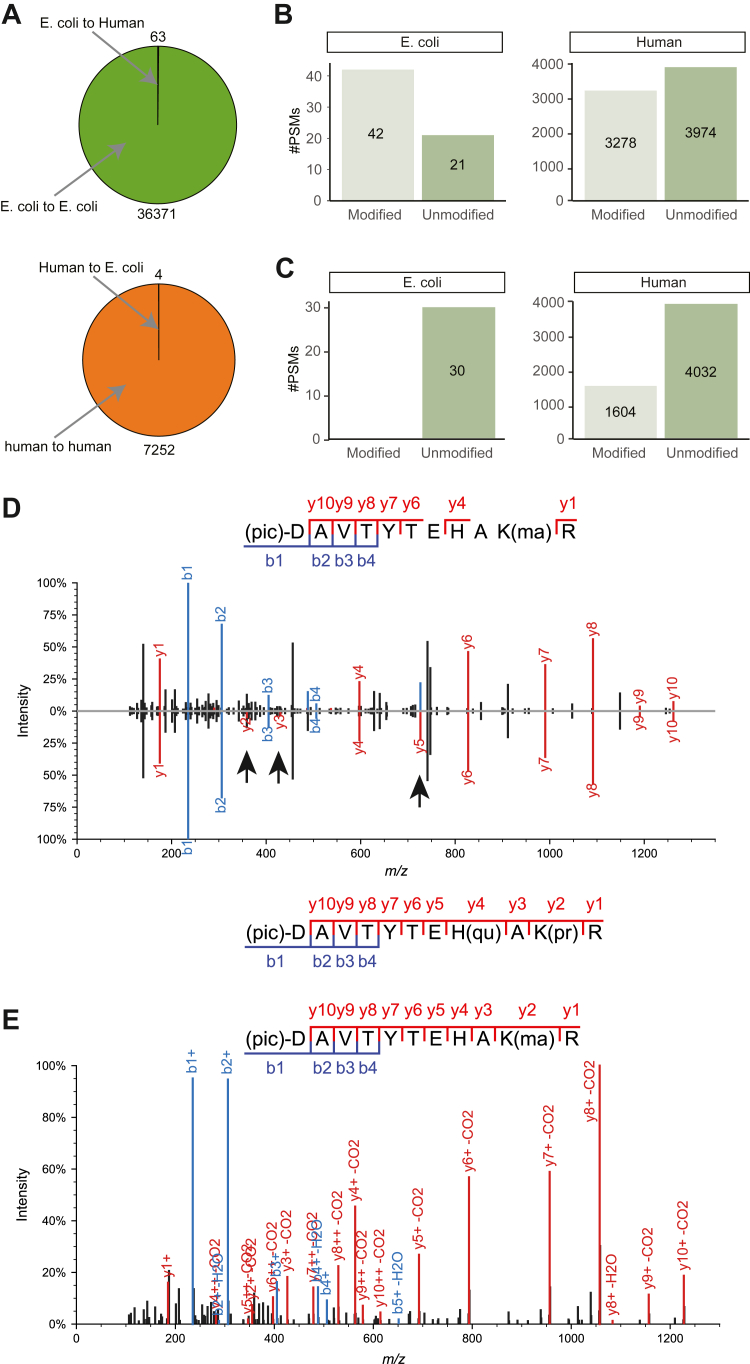
**Quality control of HiP-Frag.***A*, pie chart displaying the number of correct and incorrect PSMs assigned to *Escherichia coli* and *Homo sapiens. B*, *top*: breakdown of the PSMs originating from *E. coli* dataset and assigned to histones, categorized into modified and modified. *Bottom*: breakdown of the PSMs originated from *H. sapiens* dataset and assigned to histones, categorized into modified and modified peptides. The “unmodified” category comprises peptides carrying only chemical modifications introduced with the sample preparation protocol, while “modified” peptides carry at least one biological modification. *C*, same as (*B*) but showing the entrapment results when adopting the ‘group FDR’ method. *D*, mirror plot comparing the assignment of the same spectrum to different modified forms of the peptide H4 68 to 78 DAVTYTEHAKR. *Arrows* indicate fragment ions that are additionally matched with the OS. *E*, MS/MS annotated spectrum of the synthetic peptide H4 78 to 98 (pic)-DAVTYTEHAK(Malonyl)R(13C,15N4). FDR, false discovery rate; HiP-Frag, histone PTMs analysis with FragPipe; OS, open search; ma, malonylation; pr, propionylation; PSM, peptide-to-spectrum match; qu, quinone.

When including chemical artifacts and tryptic peptides, the search space expanded approximately 175 folds compared to a standard search considering only common modifications and ArgC peptides; hence, it is crucial to assess the confidence of the FDR estimate. This task was accomplished by employing an entrapment approach, searching together spectra of two different species, *Homo sapiens* and *Escherichia coli*. Three raw files consisting of core histones extracted from MDA-MB-436 and processed with the PRO-PIC protocol and three raw files consisting of *E. coli* total proteome (35) were analyzed together in FragPipe with the DMO workflow (Supplemental Fig. S4). The rationale is as follows: since *E. coli* is a prokaryote and does not have histones, we can *bona fide* assume that a spectrum assigned to a histone peptide in the *E. coli* dataset is a false match. We computed the error rate by dividing the number of histone-modified PSMs derived from the *H. Sapiens* dataset by the number of histone-modified PSMs derived from the *E. coli* dataset. For this search, we included in the FASTA the top 100 most abundant *E. coli* proteins, to allow for mass calibration. All spectra were virtually assigned to their correct species of origin (Fig. 2*A*), and only 42 out of the 3320 PSMs matched to histone-modified sequence derived from the *E. coli* dataset (Fig. 2*B*), leading to a calculated error of 1.26%. We observed that the number of *E. coli* PSMs assigned to modified histone peptides was double that of unmodified ones (which include also peptides carrying only chemical modifications). This prompted us to repeat the analysis performing a group FDR estimation, a strategy that calculates FDRs separately for the following three groups: (1) unmodified peptides, (2) peptides modified with chemical modifications only, and (3) peptides bearing genuine biological modifications. When performing the standard target decoy approach, the assumption is that the spectra have overall the same characteristics. However, the data may contain heterogenous groups. For instance, spectra of unmodified and modified peptides likely do not have the same characteristics. As a result, the threshold to accept identifications at 1% FDR can be significantly different between groups. Hence, ignoring the group structure might cause some groups to present several false identifications, while few for others. As a matter of fact, with this strategy, we observed no *E. coli* PSM to be erroneously assigned to histone modified peptides (Fig. 2*C*). For this reason, for the searches carried out in the following sections, we adopted the group FDR strategy.

Another key aspect in the analysis of MS data of histone PTMs is combinatorics. Specifically, confident assignment of modifications to individual histone residues is complicated by the fact that multiple PTM combinations can be isobaric, producing identical mass shifts. This issue is particularly relevant in an unrestrictive search context, in which a broad range of modifications is considered, thereby increasing the likelihood of encountering isobaric PTM combinations. For example, the DMO reported a malonylation (+86.0004 Da) on H4K77 (Fig. 2*D*), whereas the OS, for the same spectrum, reported a propionyl group (+56.0262 Da) together with a +29.9742 Da mass shift on histidine 75, annotated in Unimod as “quinone” (accession number: 392). The sum of these two PTMs is 86.0004 Da, isobaric with the mass of a malonyl group. Relative to the DMO, the OS additionally matched the y2, y3, and y5 fragment ions (Fig. 2*D*), yielding a complete y-ion series. However, these y ions displayed an extremely low signal-to-noise ratio, raising the concern that the OS could generate a better match by selecting random noise rather than true fragment ions. To resolve this ambiguity, we purchased a synthetic peptide bearing H4K77 malonylation and compared the fragmentation pattern. The MS/MS spectrum of the synthetic peptide showed a distinctive feature: all y ions were accompanied by a neutral loss of carbon dioxide (Fig. 2*E*), which is consistent with the structure of the modification. Hence, we concluded that the PSM reported by the DMO was incorrect.

**Fig. 3:**
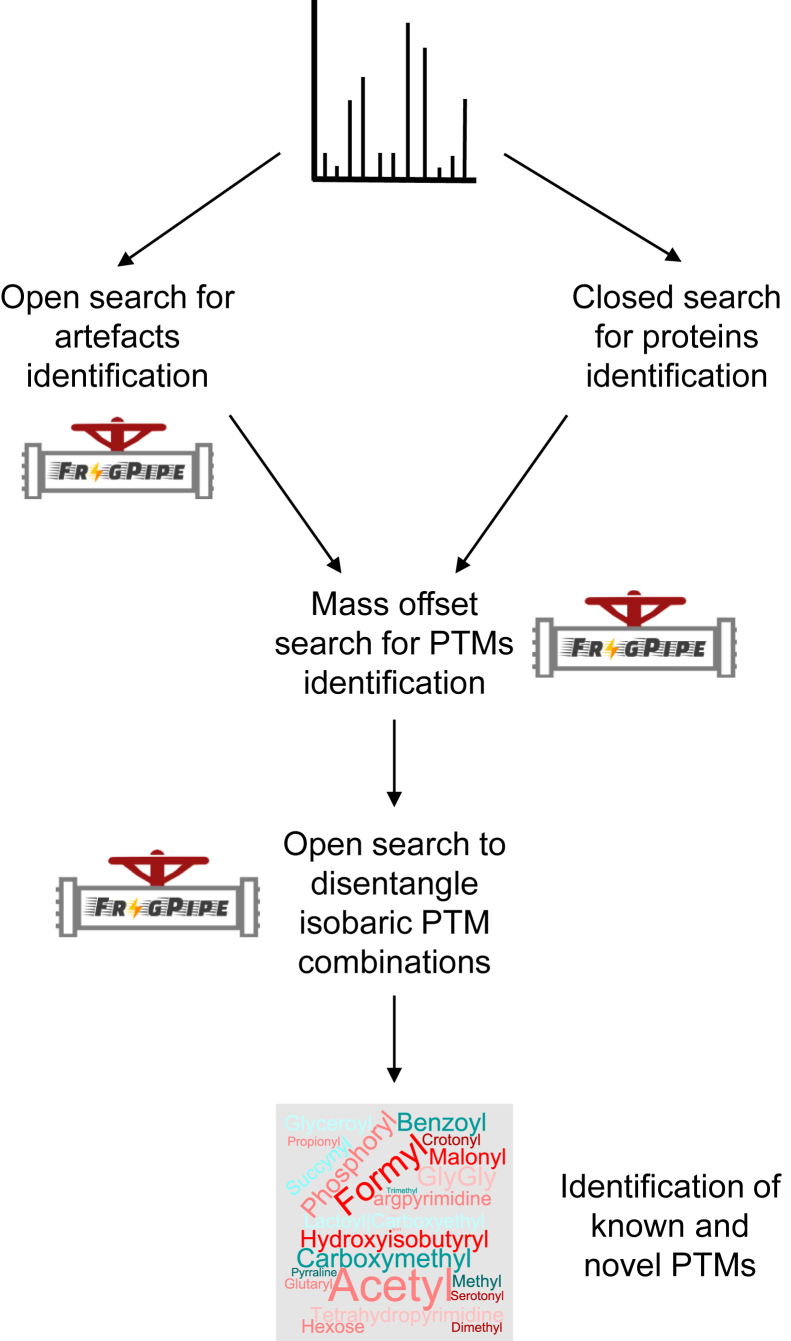
**Scheme of the HiP-Frag pipeline for the comprehensive identification of known and novel histone marks.** HiP-Frag, histone PTMs analysis with FragPipe; PTM, post-translational modification.

These assessments led us to define a search pipeline, which we named HiP-Frag (Histone PTMs Analysis with FragPipe), consisting of the following steps: (1) a closed search for protein identification; (2) an OS; (3) a DMO search for the biological modifications of interest; and (4) a further round of OS performed specifically on the PSMs identified in step 3 (Fig. 3). The first step aims to identify the most abundant proteins, aside from histones, by comparing their intensities and assessing the extent of contamination from non-histone proteins, thereby guiding the decision of whether to include them in the search database. The second step identifies the most frequent experimental artefacts, which can then be included in the subsequent DMO search, enabling a more reliable identification of histone PTMs. The fourth step evaluates whether previously unconsidered PTM combinations provide a more accurate assignment of the identified spectra; when this occurs, the corresponding PSMs are discarded.

As an additional control, we applied HiP-Frag to recombinant histone H3.1, reasoning that bacterial expression should mostly yield unmodified histones, even though bacteria can express protein-modifying enzymes and that a variety of PTMs are present in bacterial proteomes (36, 37). In addition to FDR filtering, we required PSMs to have 50% of the theoretical b- and y-ions matched, a criterion shown to ensure high-confidence histone PSMs (38). We also excluded PSMs with modifications not localized to a single position. Following these filters, 10 of 821 PSMs were retained (Supplemental Fig. S5*A*), of which seven were phosphorylated. Manual inspection confirmed these PSMs to be good quality matches, with neutral loss of phosphoric acid supporting the authenticity of the phosphorylations. This resulted in only three putative misassignments (0.37% error), below the accepted 1% threshold (Supplemental Fig. S5*B*).

### HiP-Frag Allows the Identification of 60 Previously Unreported Histone Marks on Core Histones

**Fig. 4:**
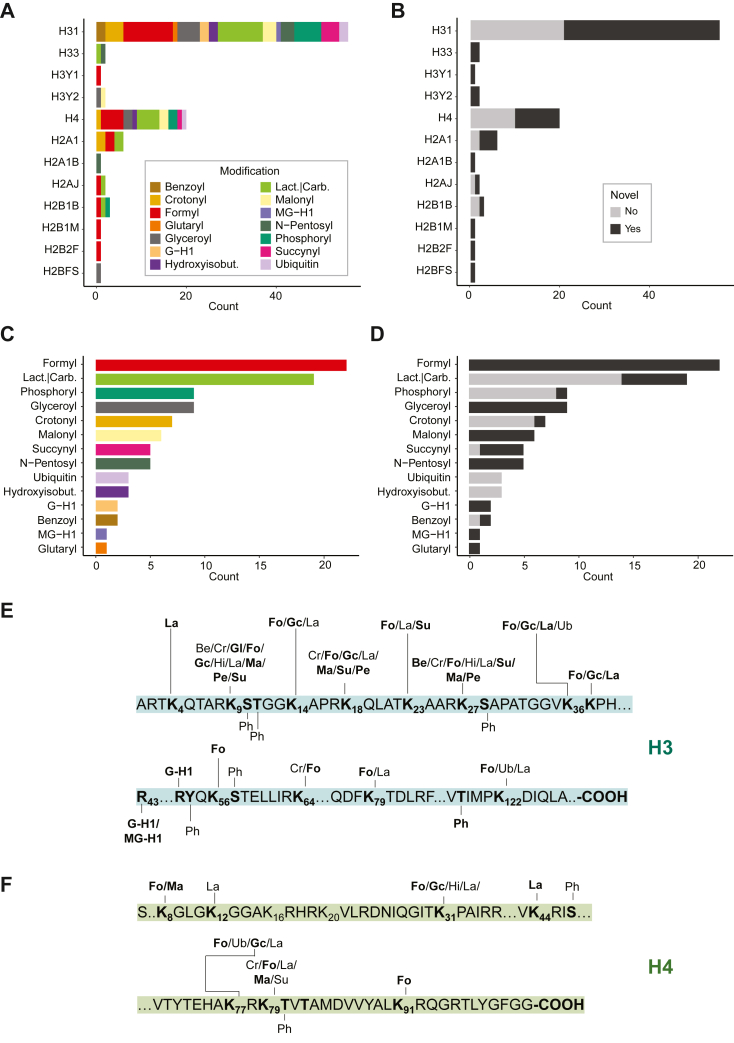
**Global characterization of the core histones modification landscape.***A*, bar chart displaying the number of marks identified for each modification class on the different core histones and core histone variants. *B*, bar chart summarizing the number of known and novel marks identified on canonical and variant core histones. *C*, bar chart displaying the total number of marks identified for each modification class. *D*, bar chart displaying the number of known and novel marks identified for the modification classes. *E*, map of the PTMs identified on histone H3. *F*, map of the PTMs identified on histone H4. Novel marks are *bold*. Be, benzoy; Cr, crotonyl; La, lactyl; Gl, glutaryl; Fo, formyl; Gc, glyceroyl; Ub, ubiquitin; Hi, hydroxyisobutyryl; Ma, malonyl; He, hexose; Ph, phosphoryl; Pe, N-Pentosyl Lysine; G-H1, glyoxal-derived hydroimiadazolone; MG-H1, methylglyoxal-derived hydroimidazolone; Lact.|Carb, either lactylation or carboxyethylation (indistinguishable by MS because of the isobaric masses); Hydroxyisobut., hydroxyisobutyrylation; 3−Deoxygluc, 3−Deoxyglucosone. Acetylation and Methylation were not annotated.

To test the power of HiP-Frag in expanding the spectrum of histone modifications beyond common PTMs, we applied this workflow to core histones purified from several different human cancer cell lines (Supplemental Table S1). In addition to the 1% FDR threshold, we adopted a 50% b and y ions matched cut-off. With these stringent quality filters, we identified a total of 96 histone marks, belonging to 14 modification classes, spread across the four canonical core histones and eight core histone variants (Fig. 4*A*). For the mapping of modified sites, we prioritized the canonical variant*, i.e.,* if the same peptide sequence was shared between multiple histone variants, we assigned it to the canonical one. For instance, the peptide KSTGGKAPR can be mapped to the H3.1, H3.2, H3.3, H3.1t, H3-7, and H3.3C variants but was assigned to the canonical variant H3.1. Supplemental Table S4 lists the modified sites that can be uniquely mapped to a single histone variant, while Supplemental Table S5 contains those that map to multiple variants, with detailed reporting of all possible variant assignments. Histone H3.1 carried the largest number of modifications, followed by histone H4 and histone H2A (Fig. 4*A*).

To verify the incidence of novel marks within this list, we intersected our dataset with the manually curated catalog of histone modifications (39). Notably, 60 out of 96 (62%) were previously unreported (Fig. 4*B*), indicating that the histone landscape remains underexplored and that this new strategy is helping to illuminate it. K-formylation, K-lactylation, and serine/threonine/tyrosine-phosphorylation emerged as the three PTMs with the highest number of identifications. (Fig. 4*C*). Quite intriguingly, K-glyceroylation, an AGEs originating from glyceraldehyde (40), was the fourth most abundant modification found. Despite having been described on histones for the first time more than 15 years ago (41), formylation is the modification for which we identified the highest number of novel sites (Fig. 4*D*). Its high frequency suggests a possible significance in chromatin dynamics and gene regulation that might have been overlooked. Analyzing the PTM distribution within the peptide sequence, we observed no specific regions with a higher frequency of modifications, suggesting that uncommon PTMs may compete for the same positions with classical marks (Fig. 4, *E* and *F*).

To strengthen the validity of our findings, we applied HiP-Frag to a previously published core histone MS dataset. For this purpose, we downloaded the dataset of Provez *et al*. (42), where the authors provided a histone PTM atlas of 21 T-cell acute lymphoblastic leukemia cell lines. This dataset consists of 149 MS RAW data acquired with a Sciex TripleTOF 6600+. We compared the distribution of modification classes and observed a strong correlation in their frequencies (r = 0.85, *p* = 5.3e-7; Supplemental Fig. S6), indicating that the overall patterns of PTM identification are consistent across the two datasets. This result suggested that - despite substantial differences in MS analyzers (Orbitrap *versus* Time-of-Flight), biological samples (cancer *versus* T-cell leukemia cell lines), and histone processing protocols (in-gel PRO-PIC *versus* in-solution PRO-PRO)- the class and the frequency of novel PTMs identified in our study were also observed in an independent dataset, thereby supporting the reproducibility of our findings.

The core histones extracted from the cancer cell lines we analyze for novel PTMs were in-gel digested. To rule out the possibility that the in-gel step generated artifacts with similar mass to the biological modification searched, we analyzed four samples (HeLa, two replicates, MCF7 and MDA-MB-231, one replicate each) processed with the PRO-PIC protocol either in-solution and in-gel. We carried out an OS to systemically compare the frequency of chemical artefacts between the two methods. The +13.9792 Da delta mass was the only modification with higher number of IDs in the in-gel compared to in-solution samples (Supplemental Fig. S7*A*). This modification co-occurred with the propionyl group (+56.0262 Da) on K residues, generating a +70.0054 Da delta mass, which is isobaric to the mass of the pyruvoyl group, a potentially interesting biological modification that however was excluded from our investigation for this reason. In addition, we also performed the DMO search to evaluate whether the other biological modifications specified as mass offsets were identified exclusively in-gel. Notably, this was not the case, since all the modification classes were detected both in-gel and in-solution (Supplemental Fig. S7*B*).

### The Application of HiP-Frag to the Linker Histone H1 Allows the Detection of 55 Histone Marks

Although histone H1 is not considered a component of the core nucleosome, since it binds to the DNA linker region outside the core structure, it is equally important in regulating chromatin function. In fact, it binds to the DNA between nucleosomes, stabilizing the higher-order chromatin structure and playing a key role in the compaction and accessibility of DNA for transcription, replication, and repair (43). Furthermore, altered levels of histone H1 variants have been observed in cancer (44). Although the modification landscape of histone H1 is less characterized compared to core histones, several modified sites have been identified (45). Therefore, we reasoned that applying our workflow to a histone H1 dataset could provide novel insights about PTMs specifically occurring on linker histones. Our group has previously developed a protocol to quantify linker histone variants in clinical samples (46). The MS RAW dataset analyzed consisted of breast cancer patient samples belonging to Luminal A and Triple-Negative subtypes. Because this protocol is different from the one employed for core histones, we first adapted HiP-Frag workflow to these settings (Supplemental Fig. S8). Furthermore, acetylations and methylations were also included in the PTMs annotation, due to the less comprehensive characterization of these modifications on histone H1 compared to core histones. Finally, because of the absence of a reference histone H1 variant, modifications mapping to multiple variants were reported individually for each variant but counted only once in the overall total. To illustrate this, we consider the peptide K(ac)ASGPPVSELITK as an example. This peptide is acetylated at the first K, and the acetylation can be mapped to K33 of histone H1.2, H1.3, and H1.4. Thus, we reported the K33 acetylation separately for each of the three variants, but we considered it as one when computing the total count of the modifications identified.

**Fig. 5:**
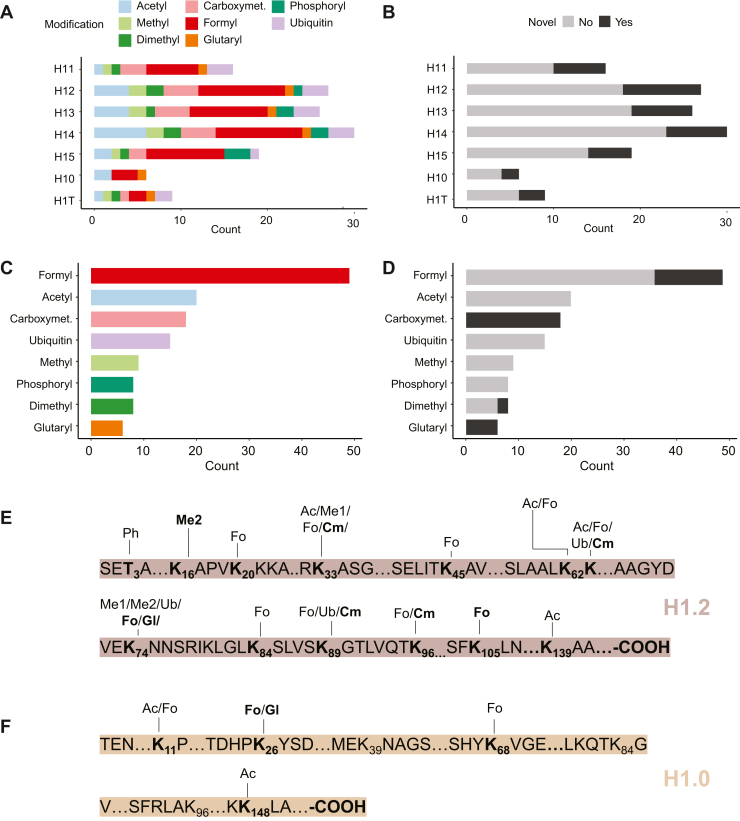
**Global characterization of the linker histone modification landscape.***A*, bar chart displaying the number of marks identified for each modification class on linker histones. *B*, bar chart displaying the number of known and novel marks identified on linker histones. *C*, bar chart summarizing the total number of marks identified for each modification class. *D*, bar chart depicting the number of known and novel marks identified for the modification classes. *E*, map of the PTMs identified on histone H1.2. *F*, map of the PTMs identified on histone H1.0. Novel marks are bold. Ac, acetyl; carboxymet, carboxymethyl; Cm, carboxymethyl; Fo, formyl; G − H1, glyoxal-derived hydroimiadazolone; Gl, glutaryl; Me1, mono-methyl; Me2, di-methyl; Ph, phosphoryl; Ub, ubiquitin.

In total, we identified 55 marks on seven variants (Fig. 5*A*); of these, 13 were novel (24%, Fig. 5*B*). Interestingly, K-formylation and acetylation were the most predominant ones (Fig. 5*C*), whereas K-carboxymethylation and K-formylation were the two most represented classes in terms of number of novel sites (Fig. 5*D*). Similar to what was observed in core histones, the modifications were distributed all throughout the protein sequences and did not present site-specific preference (Fig. 5, *E* and *F*).

## Discussion

Histone acetylation and methylation were first discovered over 60 years ago (47) and have since become central to our understanding of gene regulation and cellular processes. Over the decades, the study of these modifications has illuminated the dynamic regulation of our genomes and contributed to advances in epigenetics (48). Today, their significance extends beyond basic biology, with the development of epigenetic-based therapies and drugs aiming to treat a range of diseases, including cancer (49). In recent years, additional classes of histone modifications have been reported, emerging as an important new layer in the regulation of gene expression (50). However, their investigation is not yet as widespread as that of more well-known modifications. This is largely due to the absence of computational strategies that can effectively extend profiling beyond these classical modifications. Global proteomic studies typically involve affinity enrichment of the modification of interest, followed by LC-MS/MS analysis and identification using closed search engines that focus on detecting as many sites as possible. In contrast, MS-based histone PTM profiling aims to identify multiple modifications on a limited number of protein sequences. Here, closed search algorithms become inadequate due to the exponentially increased search space, which accounts for all possible PTM combinations on histone peptides, ultimately reducing the number of true identifications.

To address this issue, we have developed and tested a novel computational workflow, HiP-Frag, which represents the first implementation of an analytical strategy specifically designed to confidently identify novel histone PTMs. HiP-Frag enables the identification of known and novel histone marks by integrating DMO, OS, and closed search. These three types of searches are already available in FragPipe, and tutorials are available at the FragPipe website (https://fragpipe.nesvilab.org/docs/tutorial_fragpipe.html). Users can adjust the parameters based on the specific derivatization protocol adopted. HiP-Frag can scale up to whole proteome samples; indeed, we showed that it can complete the analysis of a small set of nuclear fraction samples (51), searched against the whole human proteome, in about 1 h (Supplemental Fig. S3*F*).

Caution is warranted when extending analyses beyond established histone marks (52). Despite the entrapment analysis shows that the FDR is controlled, the target-decoy approach alone does not address exhaustively the combinatorics problem of histone-modified peptides. Hence, in HiP-Frag, we incorporated an additional filtering step consisting of an OS applied to PSMs passing the FDR threshold to minimize misassignments arising from isobaric PTM combinations. Furthermore, we required PSMs to have at least 50% of the theoretical b and y ions matched, a criterion proven to deliver high-confidence histone peptide identifications (38). While these computational filters greatly reduce the potential for misidentification, validation with synthetic peptides remains the gold standard for confirming the existence of peptides bearing novel PTMs. Nonetheless, the synthesis of peptides bearing rare or chemically unstable modifications remains technically challenging and costly, making large-scale validation impractical at this stage. Future efforts could therefore prioritize synthesis to validate the most biologically relevant or novel PTMs.

We noticed that uncommon modifications tend to be localized at the same sites already known to be decorated by acetyl- and methyl-groups: this result indicates that our current definition of “chromatin states”, based only on the combinatorial presence of acetylations and methylations, may not fully recapitulate the real complexity of chromatin regulation and may be the mere reflection of the detection bias intrinsic to antibody-based analyses. Indeed, the variations in size, charge, polarity, and steric effects introduced by the specific modification can help to further fine-tune gene expression and chromatin states, providing cells with the ability to respond to different signals with higher precision and accuracy. On the other hand, the presence of bulky modifications, such as AGEs, at key sites has been proposed to contribute to the disruption of the histone code, particularly in cancer, where increased levels of AGEs are observed because of the Warburg effect. These modifications may disrupt the histone code either by directly modifying key histone sites, preventing the binding of other modifications or by inducing steric hindrance, which interferes with nucleosome assembly and stability (13, 53).

Our expanded analysis revealed the presence of several uncommon modifications occurring on histone variants as well, even though to a lesser extent than on the canonical counterparts. However, it must be noted that sample preparation methods commonly employed for histone PTMs studies have been optimized to study mainly histone H3 and H4. Therefore, the fact that these two histones bear the greatest number of modifications might be a bias resulting from the current sample processing prior to MS. Indeed, histone H2A and H2B, along with their variants (H2AX, H2AJ, H2AZ, etc.), are characterized by regions of amino acid sequence for which an ArgC-like digestion yields peptides too long for efficient MS analysis. Alternative sample preparation strategies could be tested upstream to MS and our new unrestricted search workflow to explore the complete modification landscape of such histone variants. Along the same line, the relative occurrence of the modifications we observed might be biased by the specific sample preparation and LC-MS/MS settings employed. It is known, in fact, that labile modifications, such as phosphorylation, are sensitive to experimental conditions, including heat, pH, and ionization conditions. In addition, the chemical structure of a PTM might alter the physiochemical properties of a peptide favoring (or hindering) its MS detection.

Our analytical pipeline currently focuses on histone PTM identification, but quantification is essential for understanding their regulation and role in cellular functional states. A major challenge is that uncommon modifications are often present at very low abundances (Supplemental Fig. S9 and Supplemental Table S6), sometimes approaching the detection limit of current MS instruments. As a result, their identification can be highly stochastic, with low signal-to-noise ratios, which hinders the robust and confident profiling of these PTMs. A solution to this problem may be offered by the data-independent acquisition (DIA) mode: DIA, in fact, offers enhanced sensitivity toward less abundant modifications, the ability to quantify co-eluting, isobaric peptidoforms, and an overall higher quantification accuracy (54). To this aim, the HiP-Frag pipeline could be strategically used for the generation of a comprehensive histone spectral library, to be then employed to query DIA data. This could be particularly useful since an *in-silico* spectral library is not currently an option for histone PTMs due to the lack of deep learning models capable of handling peptides with multiple modification types on different sites simultaneously. Besides the use of DIA, specific MS methods and improvements in sample preparation should be considered to ensure accurate quantification. As a matter of fact, while sample preparation artifacts—such as incomplete derivatization—are typically of a magnitude that does not significantly affect high-abundant modifications, low-abundant ones are more susceptible to these technical influences because their signal levels are comparable to those of the artifacts. Therefore, alongside the computational advancements introduced here with HiP-Frag, continued progress in wet-lab techniques will be essential for enabling the reliable profiling of novel histone modifications. This is particularly important in clinically relevant contexts where regulatory changes can be subtle, and conventional profiling of acetylation and methylation may fail to provide sufficient insight into the resistance mechanisms.

In conclusion, HiP-Frag is a novel computational tool that can aid researchers in unveiling the ‘dark matter’ of cellular epigenomes. This was showcased here by its application to a relatively small set of MS histone datasets, including core histones from various human cancer cell lines and linker histones from breast cancer tissues, which revealed several sites modified by new classes of modifications.

## Data availability

The mass spectrometry proteomics data have been deposited to the ProteomeXchange Consortium *via* the PRIDE (55) partner repository with the dataset identifier PXD061934. The mass spectrometry data related to MDA-MB-468 cell lines processed with different digestion protocols were previously deposited on the ProteomeXchange Consortium *via* the PRIDE partner repository with the dataset identifier PXD024745. *E. coli* dataset used for the entrapment analysis can be accessed with the dataset identifier PXD011189. FFPE tissues used in the DMO optimization process can be accessed with the dataset identifier PXD043551. The dataset of Provez *et al*. can be retrieved with the identifier PXD031500. Nuclear fraction samples were previously deposited to the MassIVE repository with the dataset identifier MSV000092560. The code used for the analyses as well as data visualization is available at https://github.com/alessandro-vai/HiP-Frag.

## Supplemental Data

This article contains supplemental data.

## Conflict of interest

A.I.N. is the founder of Fragmatics and serves on the scientific advisory boards of Protai Bio, Infinitopes, and Mobilion Systems. A.I.N., F.Y., and D.A.P. have financial interest due to the licensing of MSFragger and IonQuant to commercial entities. The other authors declare that they have no competing interests.

## Supplementary data

Supplementary Table S6

Annotated Spectra
