# Supplementary material for: Breaking Boundaries in Histone Modification MS-Based Detection: A Tailored Search Strategy for Unrestricted Identification of Novel Epigenetic Marks
Source: Mol Cell Proteomics. 2025 Sep 30;24(11):101080. doi: 10.1016/j.mcpro.2025.101080 (PMC12634847; doi:10.1016/j.mcpro.2025.101080)
Supplement: Supplementary Information [file mmc1.docx]

| **Cell line** | **Origin** | **Growth medium** |
| --- | --- | --- |
| MCF10A | Breast, normal | DMEM:Ham's F12K (1:1)+5% Horse Serum+2 mM L-Glutamine+20ng/ml hEGF+50ng/ml Cholera Toxin+10ug/ml Insulin+0.5ug/ml hydrocortisone |
| MCF7 | Luminal A breast cancer | DMEM + 10% FBS |
| MDA-MB-231 | Triple Negative breast cancer | DMEM + 10% FBS |
| NB4 | Promyelocytic Leukemia | RPMI 1640+10% FBS+2mM L-Glutamine |
| A2780 | Ovarian cancer | DMEM +10% FBS |
| SKOV3 | Ovarian cancer | RPMI 1640 + 10% FBS+2mM L-Glutamine |
| UM-SCC-6 | Head and neck squamous cell carcinoma | DMEM + 10% FBS + Non-Essential Amino Acids+ 2mM L-Glutamine |
| **Cell line** | **Origin** | **PRIDE acc. #** |
| HCEC | Colon cancer | PXD039819 |
| Panc1 | Pancreatic ductal adenocarcinoma | PXD061465 |

Table S1 Cell lines used for the annotation of core histones modified sites. Three raw files for each cell line corresponding to three biological replicates were searched

Table S3 Summary of delta masses identified generated by PTMSpheperd, related to the open search results of MDA-MB-231 cell line.


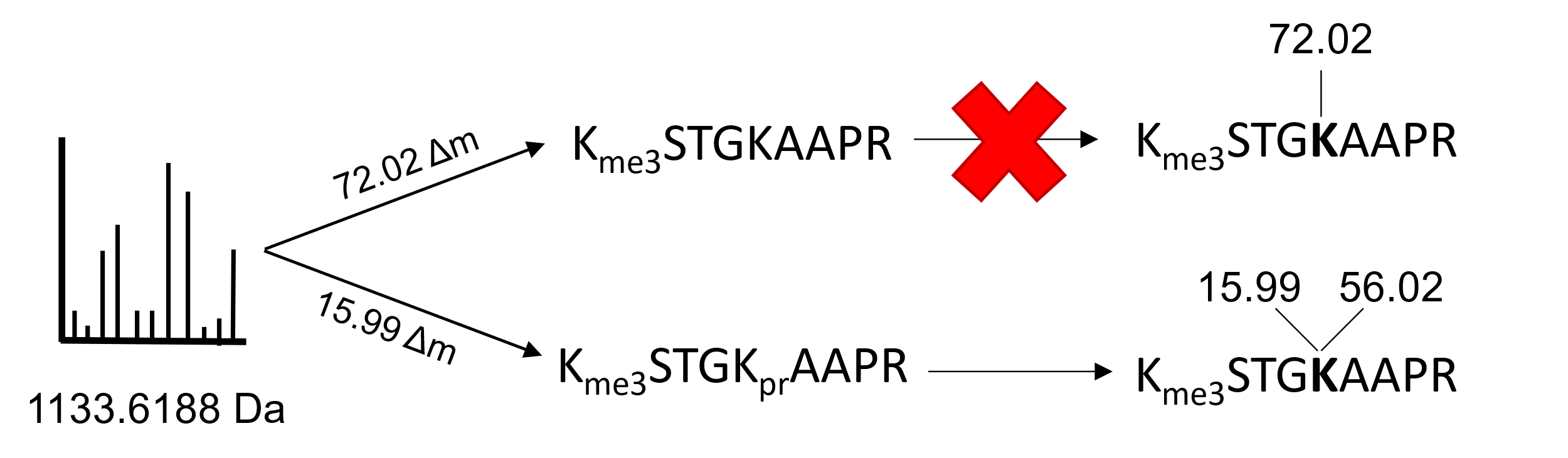


Figure S1 Illustration depicting the challenge of delta mass minimization in the open search. The open search fails to identify lysine 9 lactylation because it prioritizes matching the candidate peptide with a propionyl (pr) group at that position, to minimize the delta mass between the observed precursor and the candidate peptide. me3: tri-methylation; Δm: delta mass


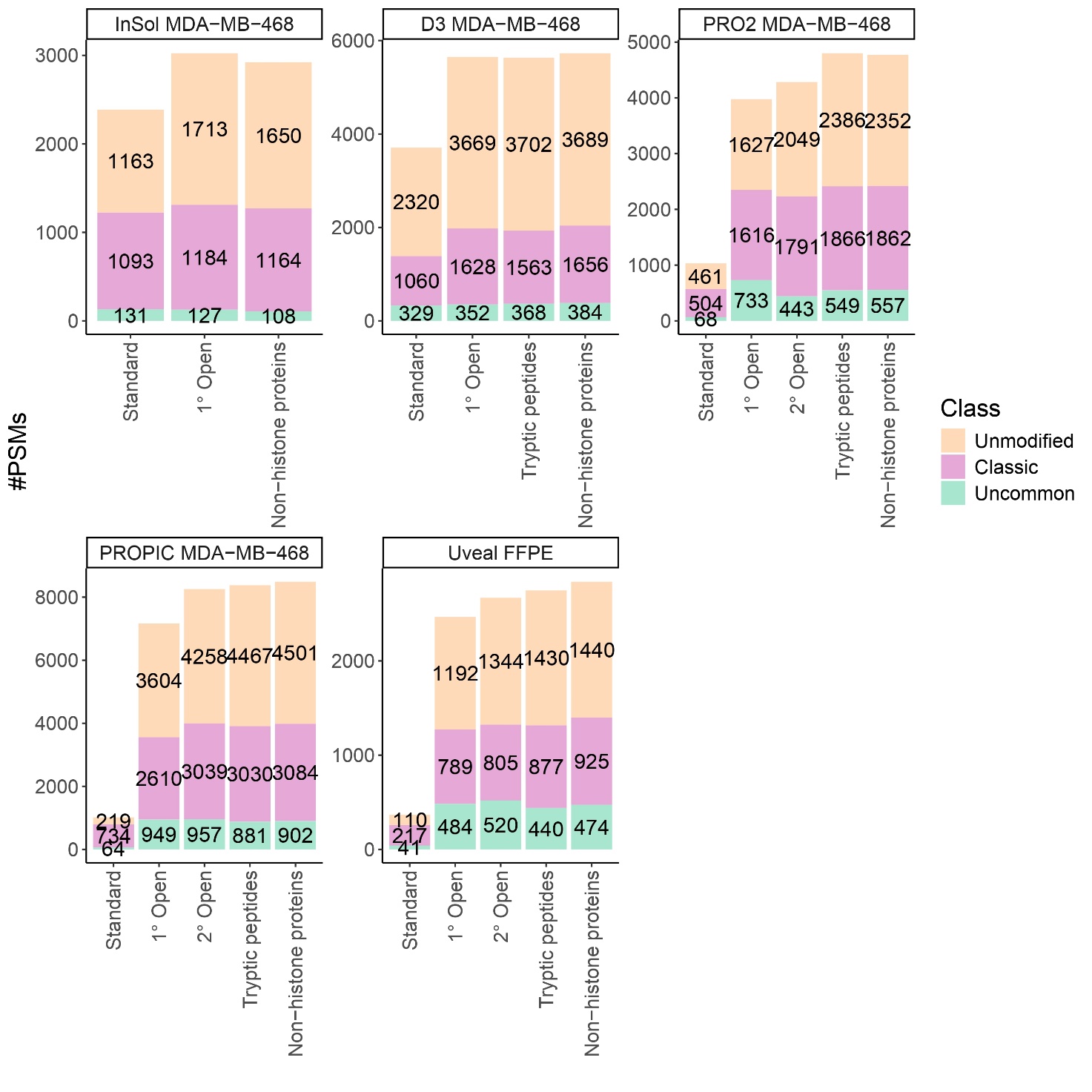


Figure S2 Number of peptide-to-spectrum matches (PSMs) identified at each step of the optimization strategy in the various datasets.


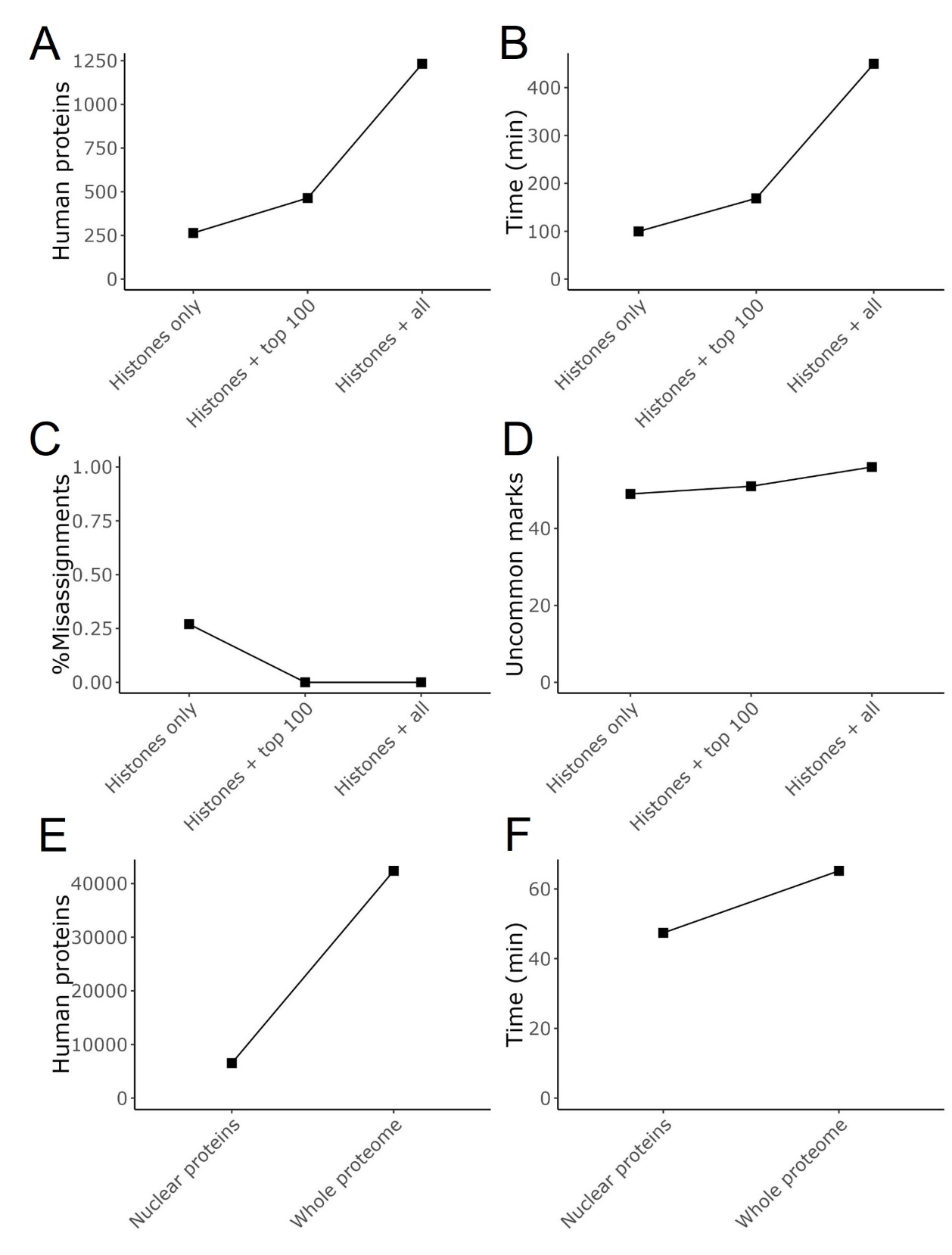
Figure S3. Computing time and error rates in different search spaces. A) Numbers of human proteins present in the FASTA file. B) Computing time for each search space. C) Percentage of *E. coli* spectra assigned to histone modified peptides. D) Number of uncommon histone marks identified in the different search spaces. E) Number of proteins present in the FASTA file used for searching nuclear fractions samples. F) Computing time for the search against the indicated database. Histone only: FASTA contains only histone proteins. Histones + top 100: FASTA file contains histone proteins plus the top 100 most abundant non-histone proteins identified through a closed search. Histones + all: FASTA file contains histone proteins plus all the non-histone proteins identified through the closed search. Nuclear proteins: FASTA file containing only the proteins identified in the closed search. Whole proteome: UniProt human proteome FASTA. Min: minutes


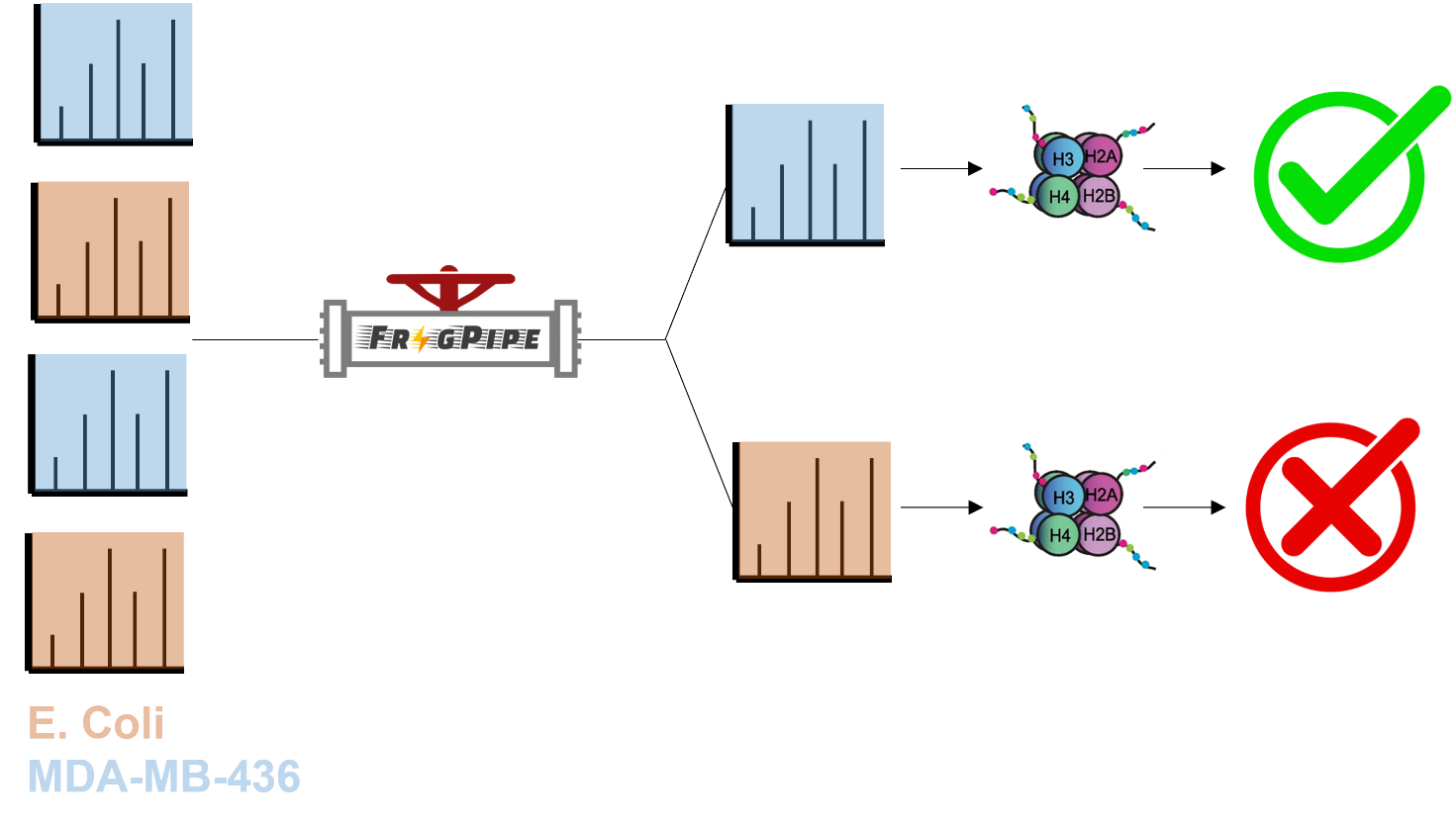


Figure S4. Scheme of the entrapment search to estimate the percentage of HiP-Frag misassignments. *E. coli* RAW files and MDA-MB-436 RAW files were analyzed together as a single group and searched against a database consisting of histone proteins and 100 *E. coli* proteins to allow for mass spectra calibration. Since *E. coli* is an organism lacking histones, any *E. coli* spectrum assigned to a histone peptide can be considered an incorrect identification.


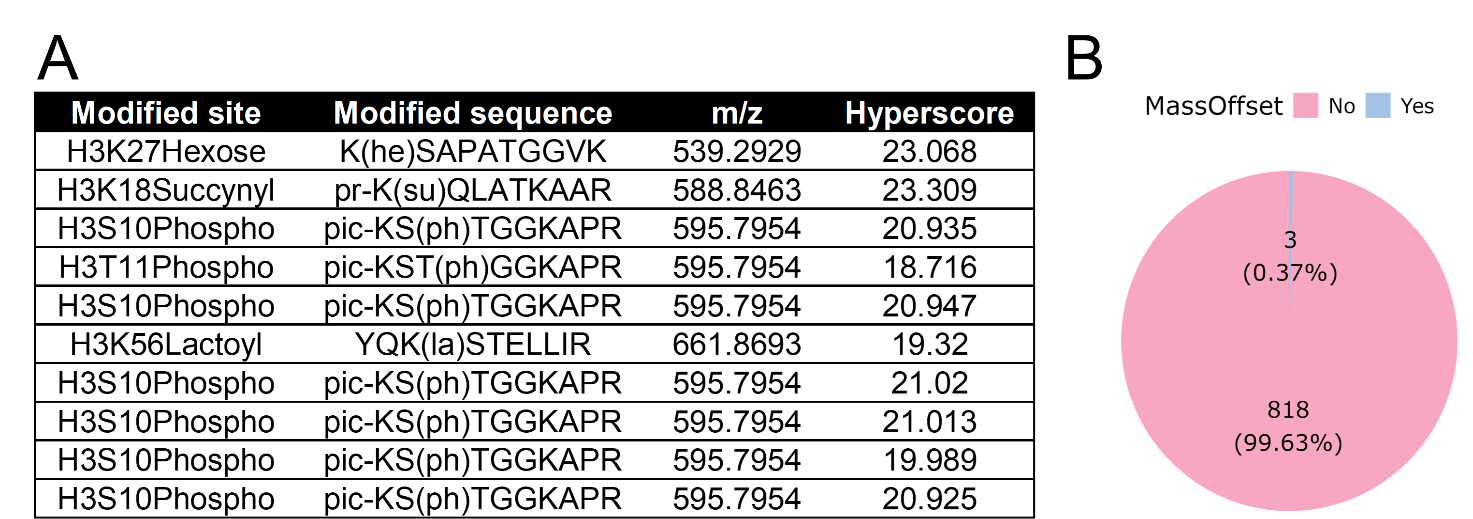


Figure S5 Hip-Frag application on recombinant histone H3.1. A) Table of PSMs having at least 50% of y or b ions matched assigned to uncommon modifications. B) Pie chart showing the percentage of PSMs assigned to uncommon modifications searched as mass offsets (not considering phosphorylated peptides) over the total number of histone H3.1 peptides with at least 50% of y or b ions matched.


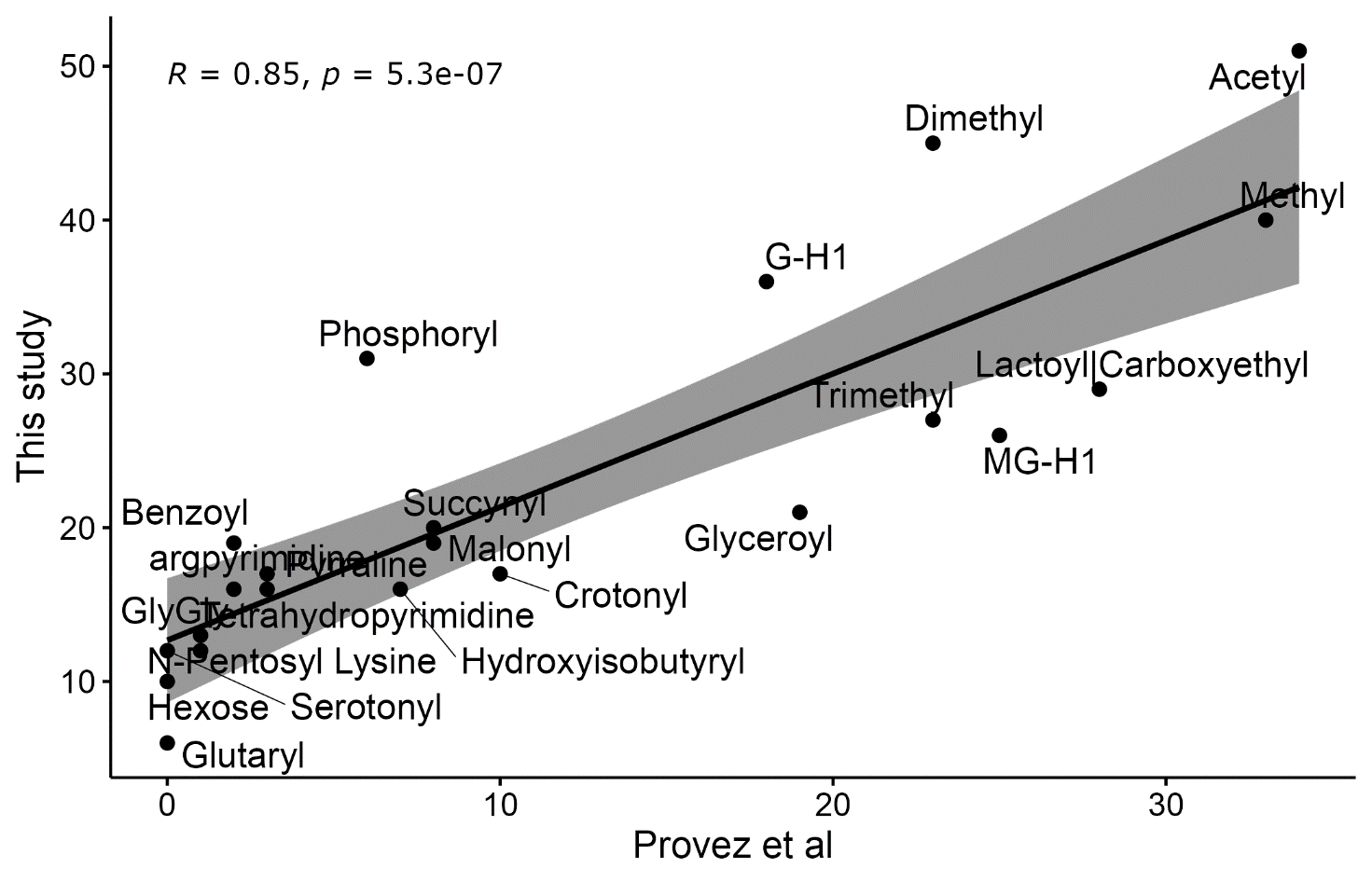


Figure S6. Scatterplot showing the correlation between the frequency of the modification classes identified in this study and in that of Provez et al. Formylation was excluded from the comparison because Provez et al. used formic acid, which can artificially introduce formylation at lysine residues. R: Pearson’s correlation coefficient.

B+B

A


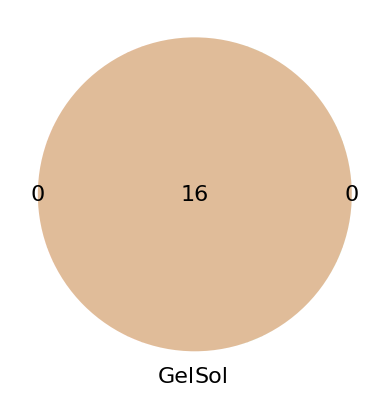

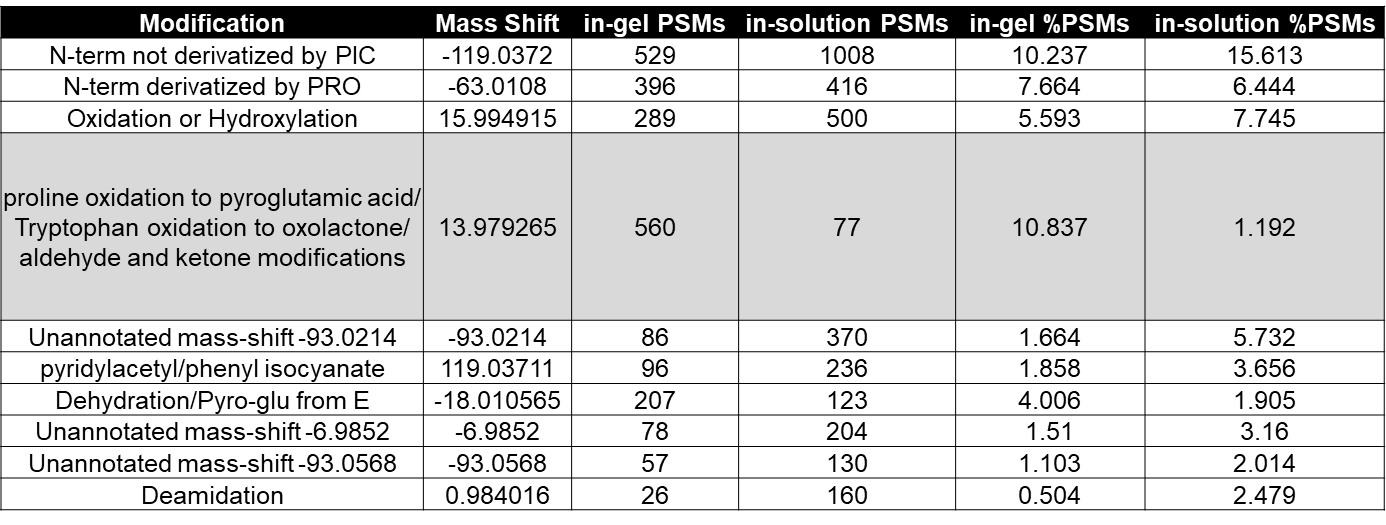


Figure S7 Assessing the extent of chemical artefacts generated with the in-gel digestion. A) Table showing the top 10 most identified delta masses by the open search as reported by PTMSpheperd. The delta mass highlighted in gray is the only artefacts significantly more frequent in in-gel than in-solution samples. B) Venn diagram depicting the overlap of the modification classes identified in in-gel and in-solution samples.


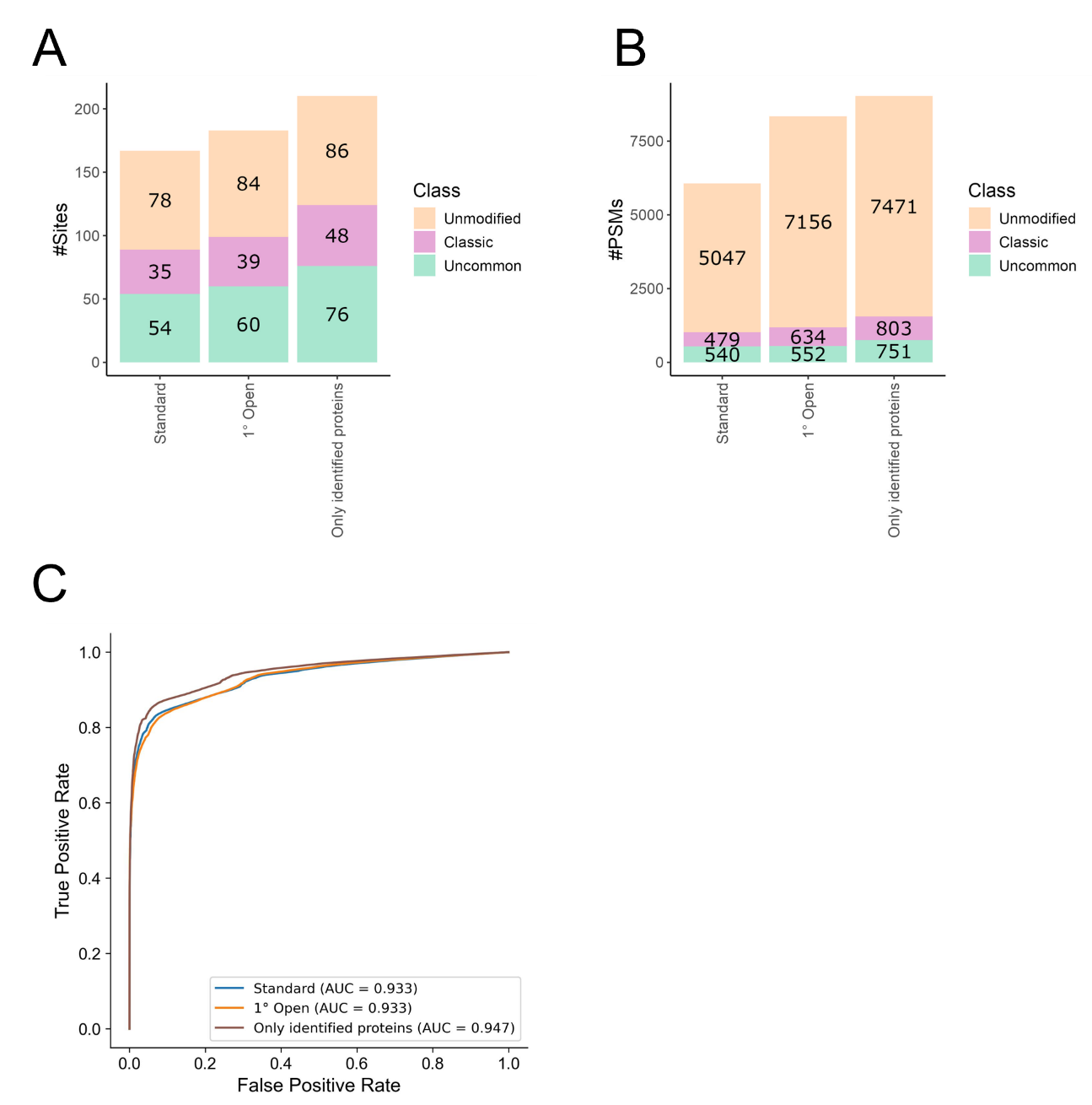


Figure S8. Optimization of the search settings for linker histones H1. A) Number of modified sites identified at each step. B) Number of PSMs identified at each step. C) ROC curve displaying the proportion of target/decoy matches at each step. Standard: search with default settings for a total proteome search; 1° Open: Search including the most abundant modification identified by an open search; Only identified proteins: same as in 1° Open but using as database restricted to the proteins identified in the previous step.


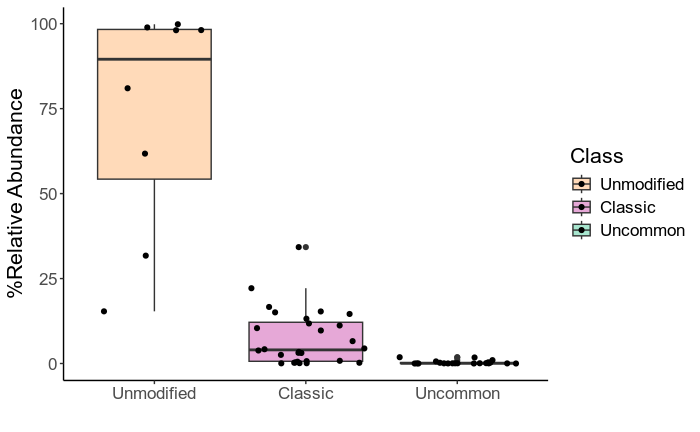
Figure S9. Boxplot display of the percentage relative abundance (%RA) of the quantified modifications for the different classes. Classic: K-acetylation and K-methylation, median %RA: 4%. Uncommon: mass offsets listed in Table S2, median %RA 0.06%.
