# Supplementary material for: Breaking Boundaries in Histone Modification MS-Based Detection: A Tailored Search Strategy for Unrestricted Identification of Novel Epigenetic Marks
Source: Mol Cell Proteomics. 2025 Sep 30;24(11):101080. doi: 10.1016/j.mcpro.2025.101080 (PMC12634847; doi:10.1016/j.mcpro.2025.101080)

Recombinant H3.1

Raw file: HF231017_CG_H3_1_D3Ac_wo_NaAc_1, Scan: 9175, m/z: 539.2929, Charge: 2, RT (min): 21.78, Score: 23.068 (H3K27he)

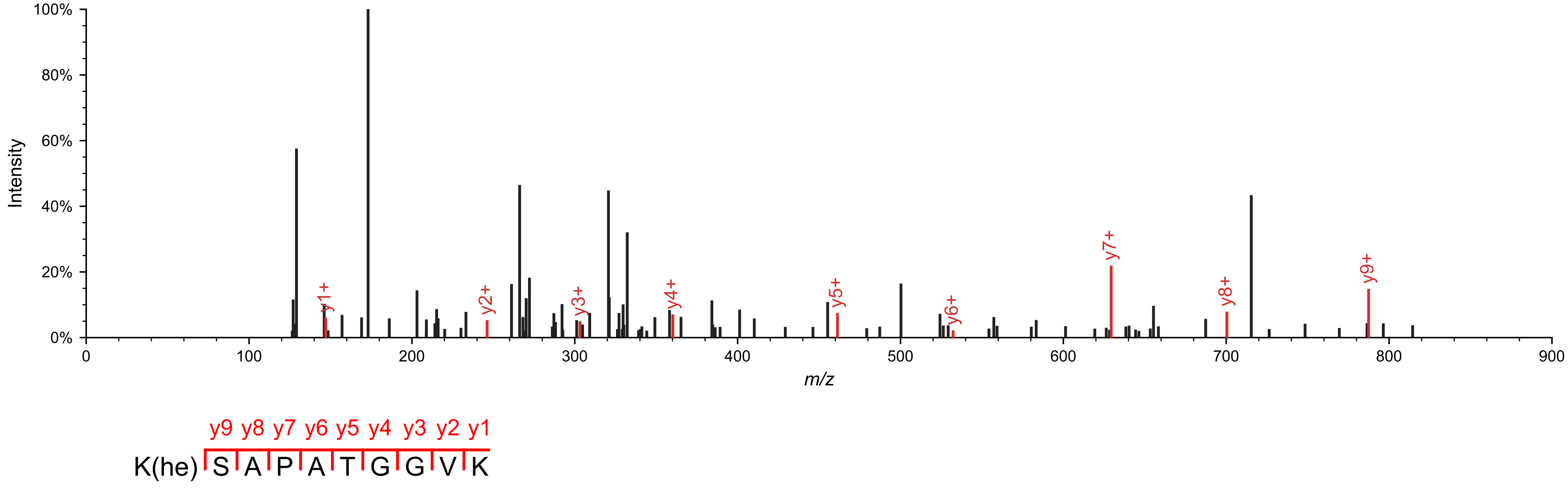

Raw file: HF231017_CG_H3_1_D3Ac_wo_NaAc_1, Scan: 9761, m/z: 588.8463, Charge: 2, RT (min): 23.00, Score: 23.309 (H3K18su)

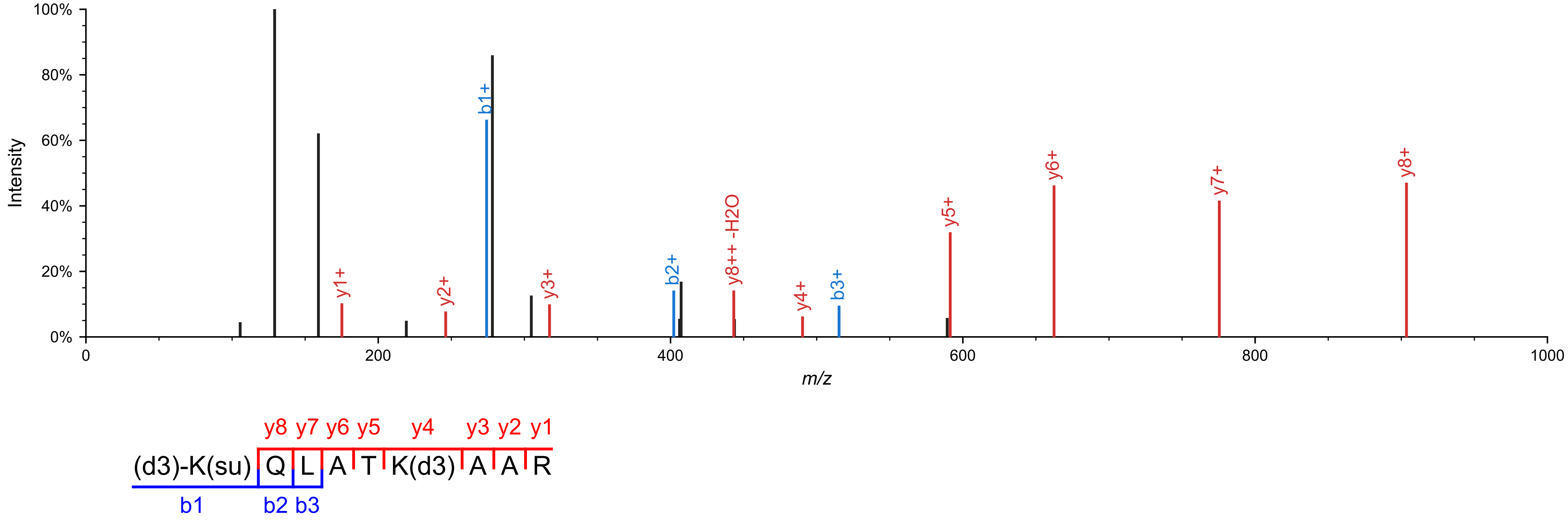

Raw file: HF231017_CG_H3_1_D3Ac_wo_NaAc_1, Scan: 12210, m/z: 595.7954, Charge: 2, RT (min): 28.63, Score: 20.935 (H3S10ph)

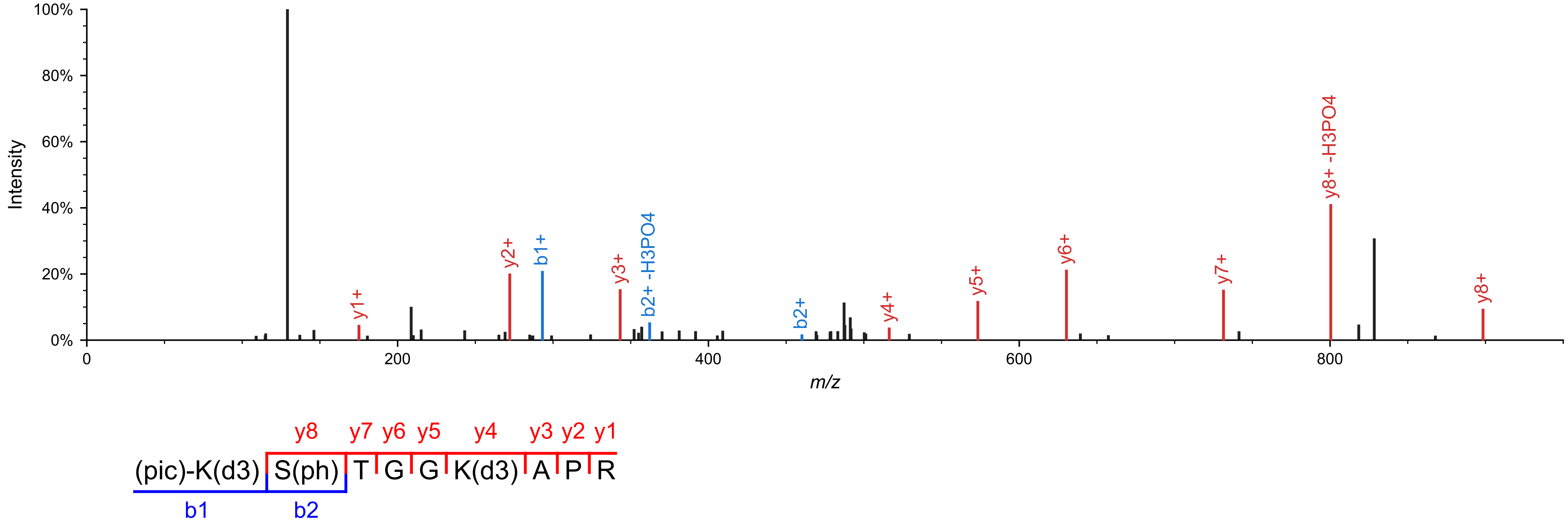

Raw file: HF231017_CG_H3_1_D3Ac_wo_NaAc_1, Scan: 12281, m/z: 595.7954, Charge: 2, RT (min): 28.80, Score: 15.723 (H3S10ph)

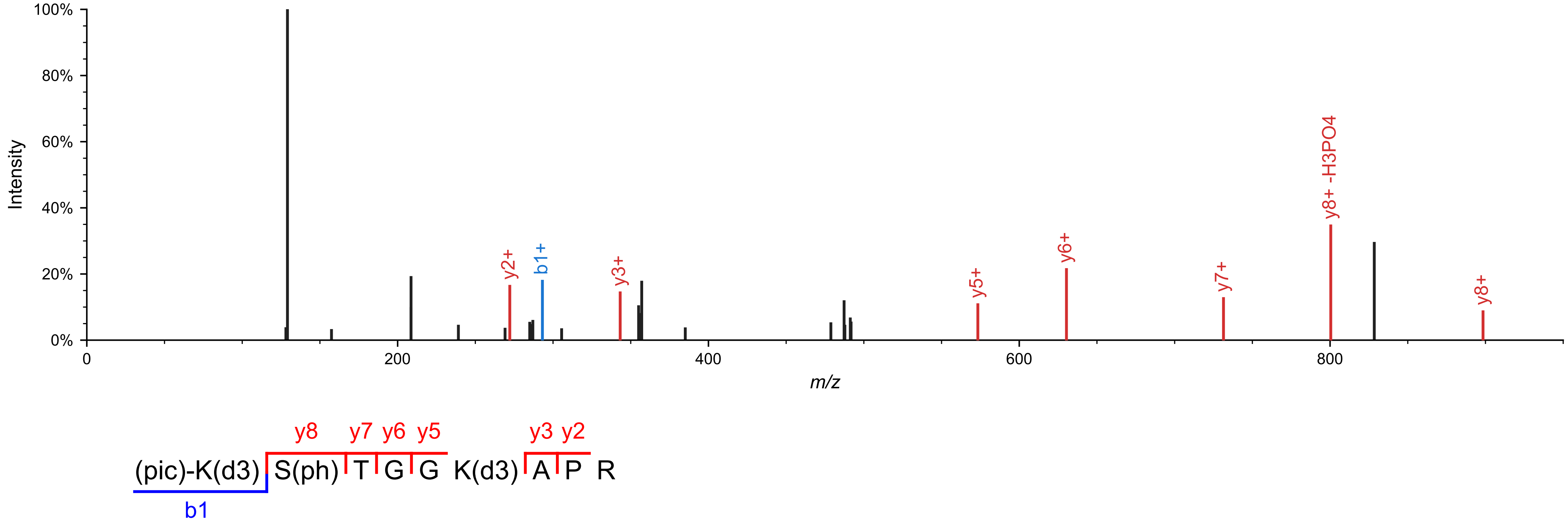

Raw file: HF231017_CG_H3_1_D3Ac_wo_NaAc_1, Scan: 12458, m/z: 595.7954, Charge: 2, RT (min): 29.26, Score: 18.716 (H3T11ph)

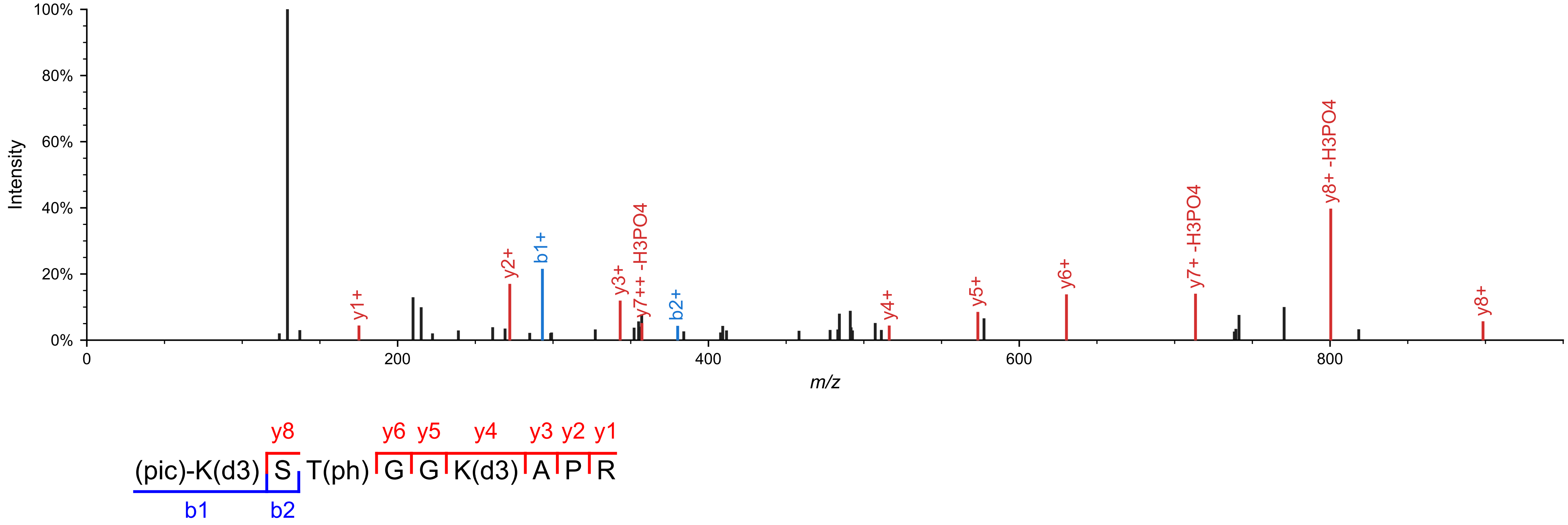

Raw file: HF231018_CG_H3_1_D3Ac_wo_NaAc_2, Scan: 11516, m/z: 595.7954, Charge: 2, RT (min): 28.69, Score: 20.947 (H3S10ph)

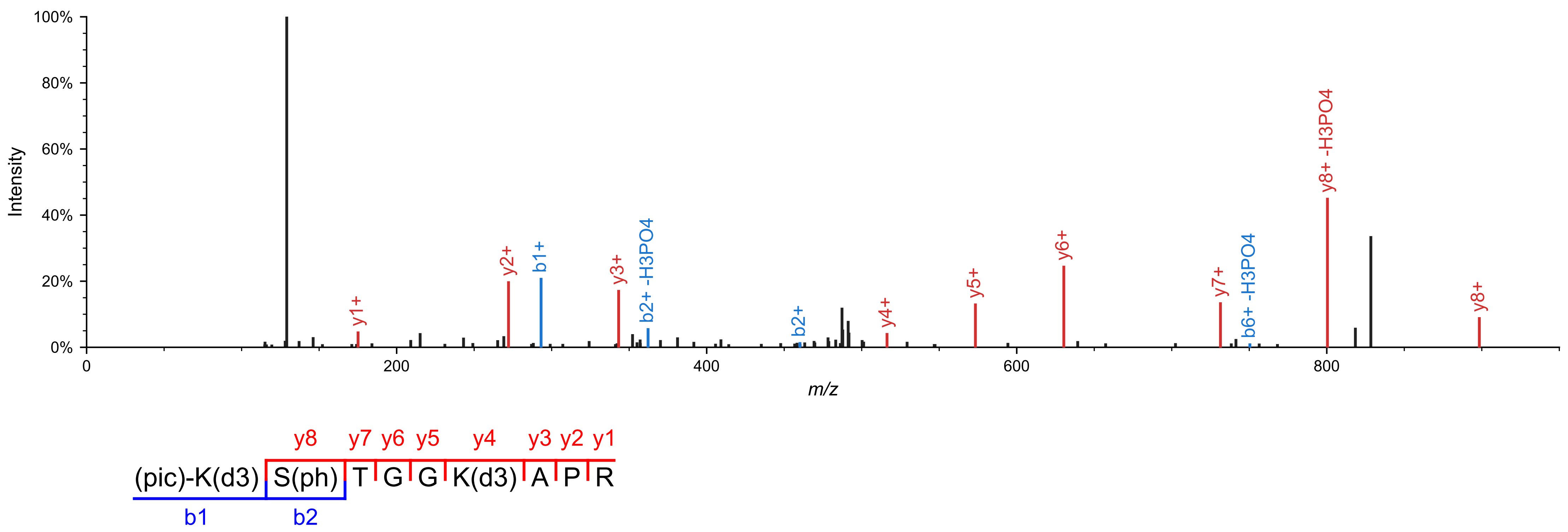

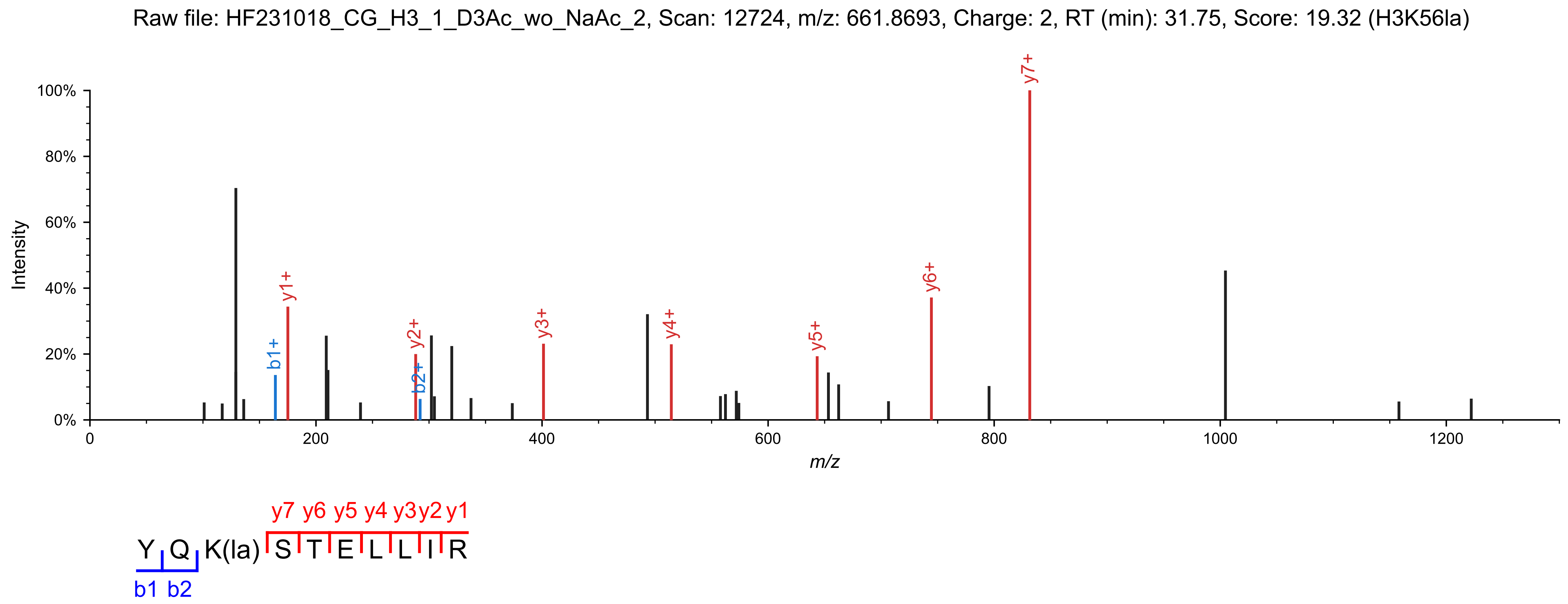

Raw file: HF231018_CG_H3_1_D3Ac_wo_NaAc_2, Scan: 12724, m/z: 661.8693, Charge: 2, RT (min): 31.75, Score: 19.32 (H3K56la)
Intensity
100%
80%
60%
40%
20%
0%
0
200
400
600
800
1000
1200
m/z
b1+
y1+
y2+
b2+
y3+
y4+
y5+
y6+
y7+
y7 y6 y5 y4 y3 y2 y1
Y Q K(la) S T E L L I R
b1 b2

Raw file: HF231018_CG_H3_1_D3Ac_wo_NaAc_3, Scan: 11702, m/z: 595.7954, Charge: 2, RT (min): 28.68, Score: 21.02 (H3S10ph)

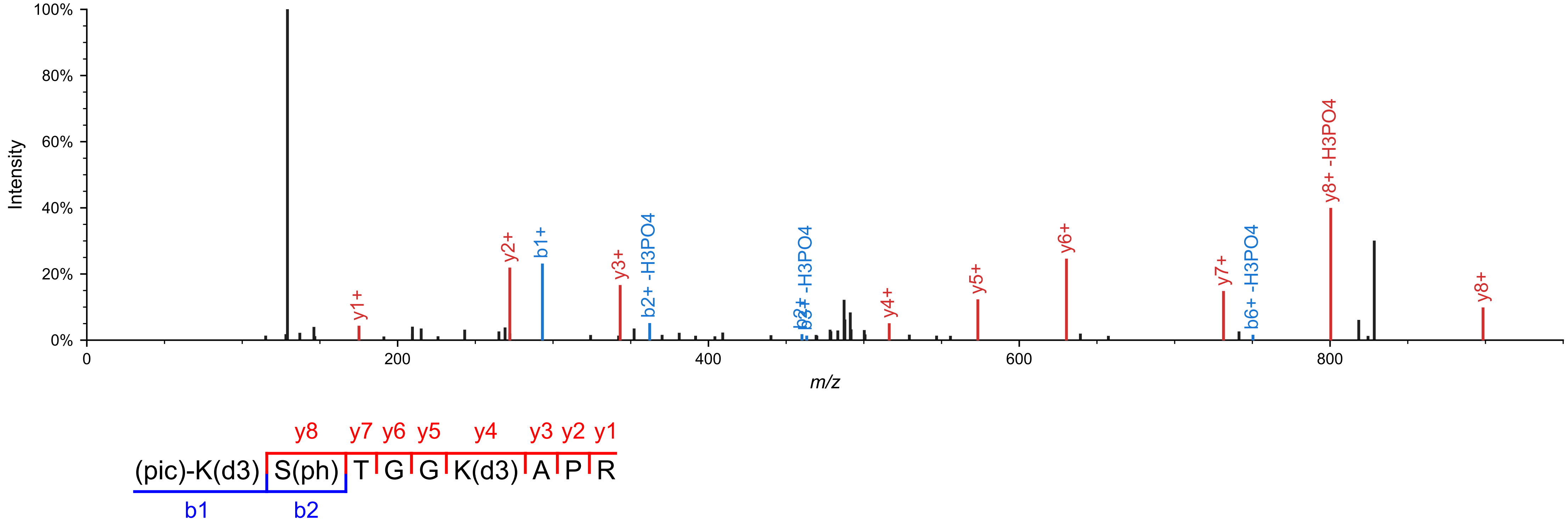

Raw file: HF231018_CG_H3_1_D3Ac_wo_NaAc_3, Scan: 11779, m/z: 595.7954, Charge: 2, RT (min): 28.85, Score: 21.013 (H3S10ph)

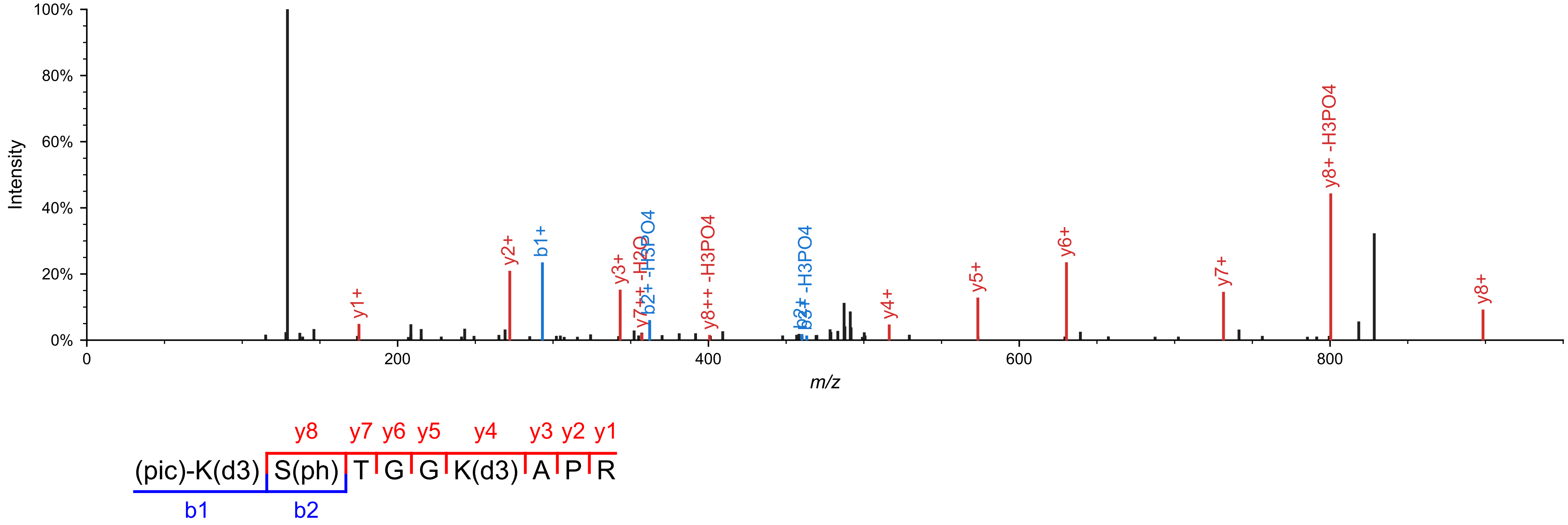

Raw file: HF231018_CG_H3_1_D3Ac_wo_NaAc_3, Scan: 11843, m/z: 595.7954, Charge: 2, RT (min): 29.02, Score: 19.989 (H3S10ph)

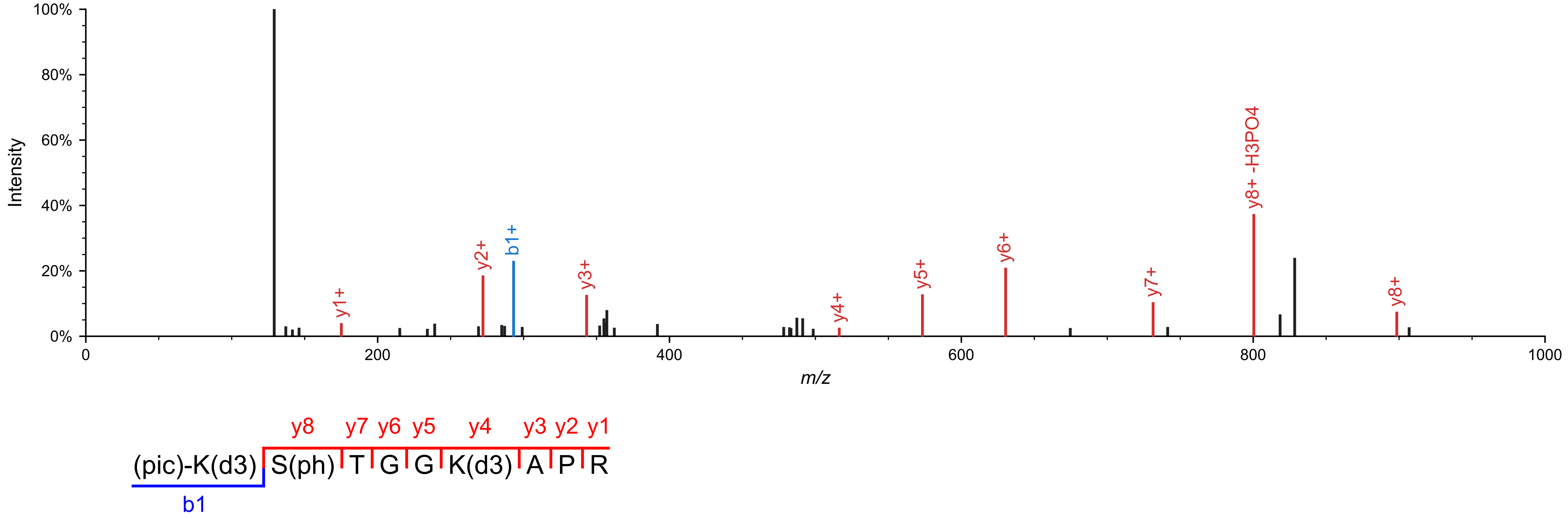

Raw file: HF231018_CG_H3_1_D3Ac_wo_NaAc_3, Scan: 11908, m/z: 595.7954, Charge: 2, RT (min): 29.19, Score: 20.925 (H3S10ph)

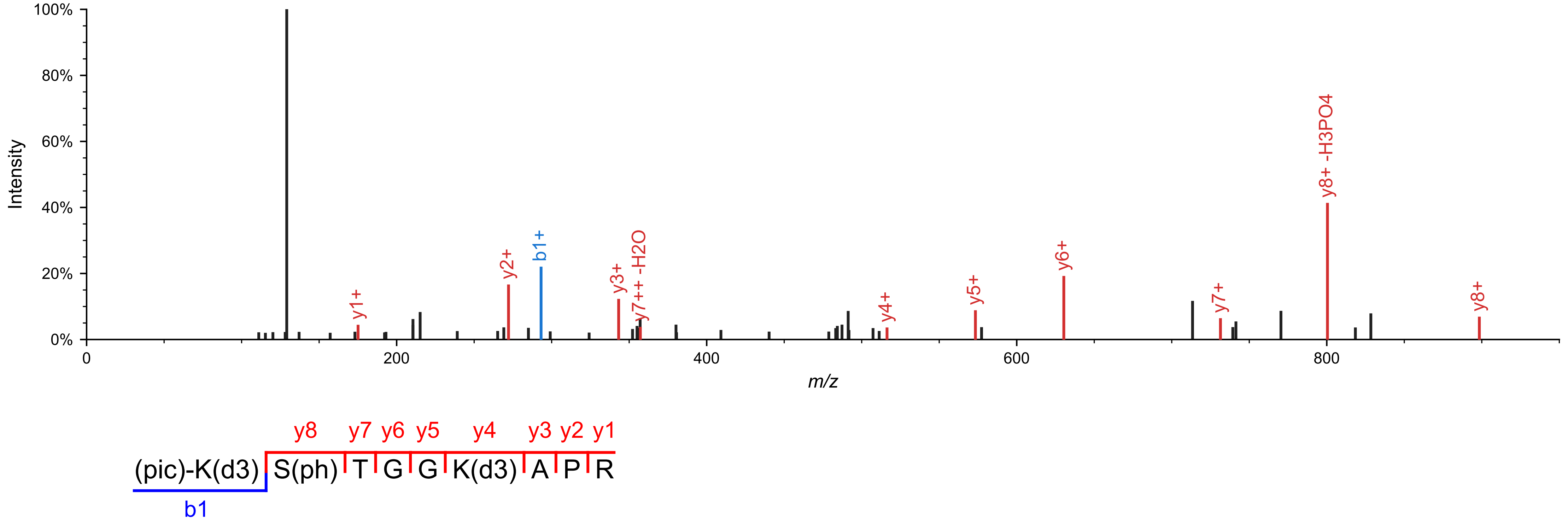

Core histones

Raw file: HF220822_RN_33_H_N, Scan: 7052, m/z: 528.7766, Charge: 2, RT (min): 15.88, Score: 17.106 (H2AJK9Formyl)

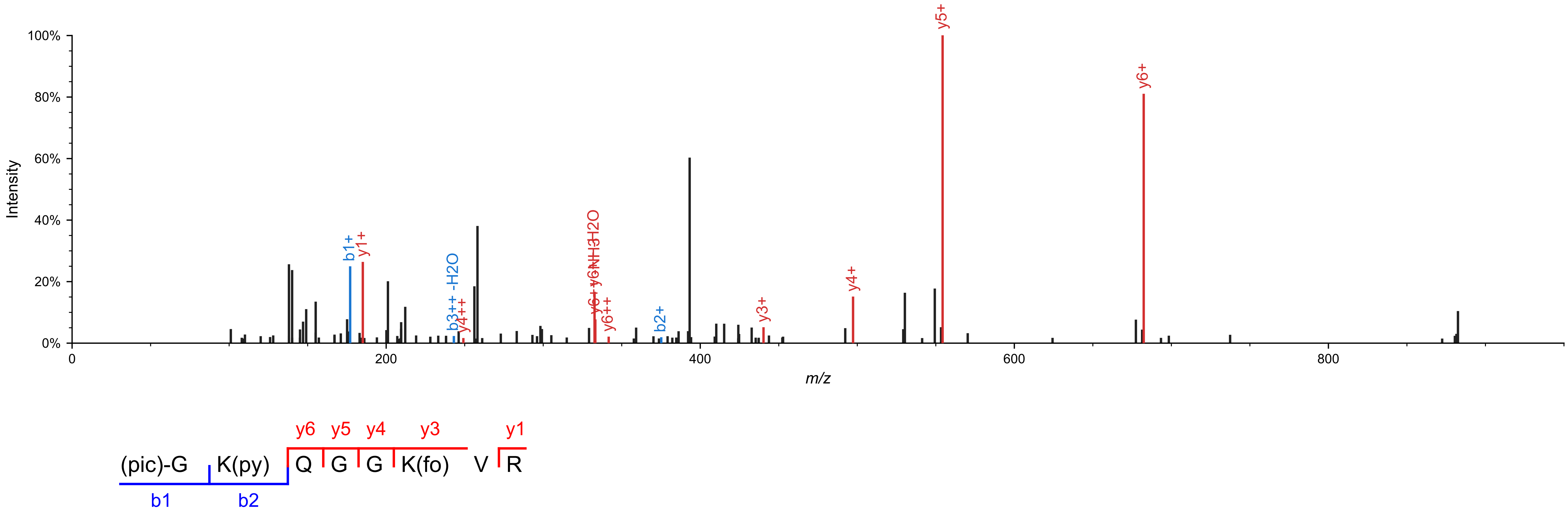

Raw file: HF220822_RN_33_H_N, Scan: 8146, m/z: 437.245, Charge: 2, RT (min): 17.90, Score: 14.4 (H2B1BK12Formyl)

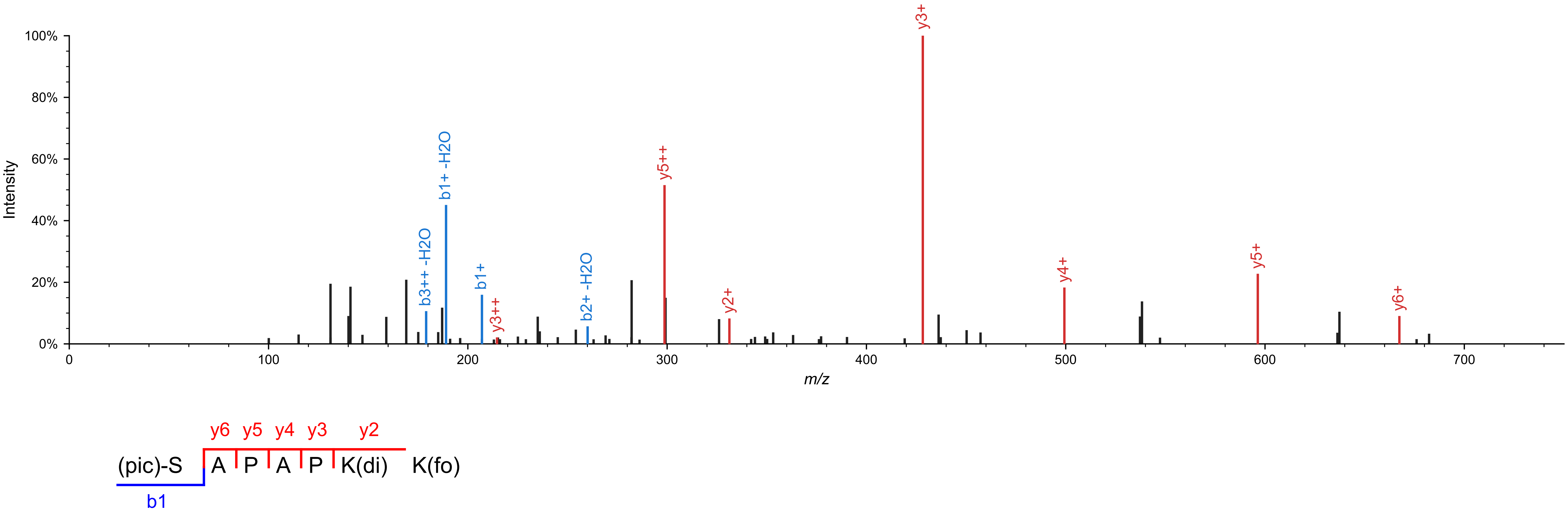

Raw file: HF220822_RN_33_H_N, Scan: 13588, m/z: 668.3663, Charge: 2, RT (min): 28.01, Score: 26.426 (H4K8Malonyl)

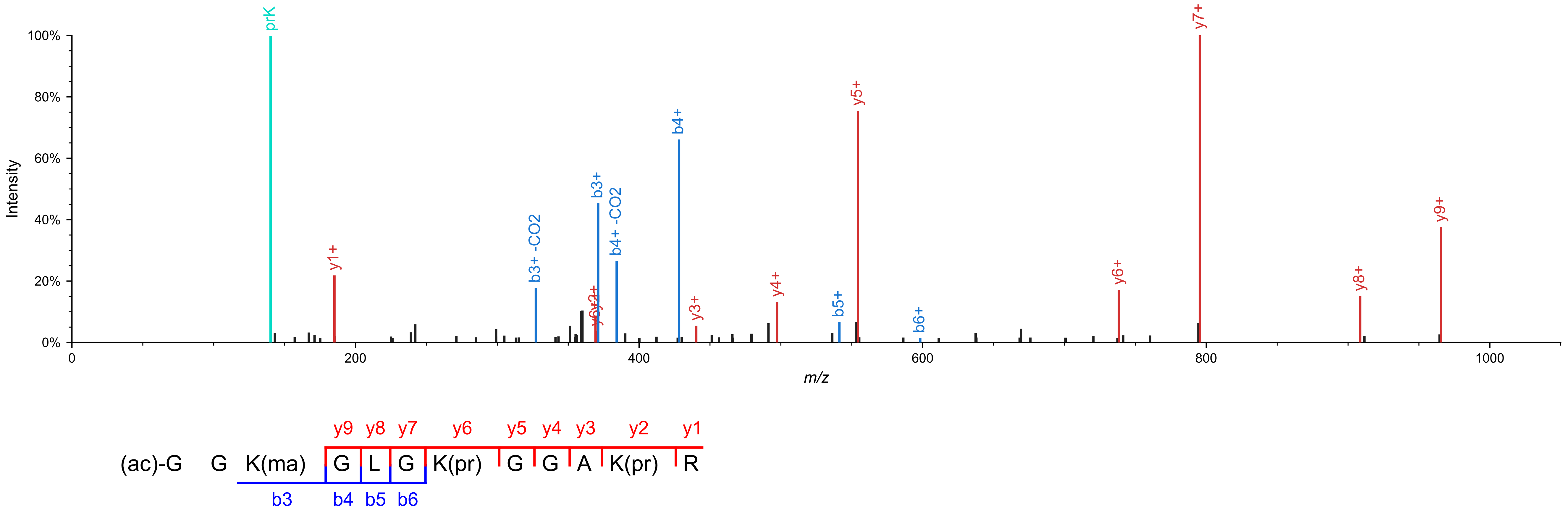

Raw file: HF220822_RN_34_H_N, Scan: 4179, m/z: 549.2949, Charge: 2, RT (min): 10.73, Score: 19.948 (H31K9Glyceroyl)

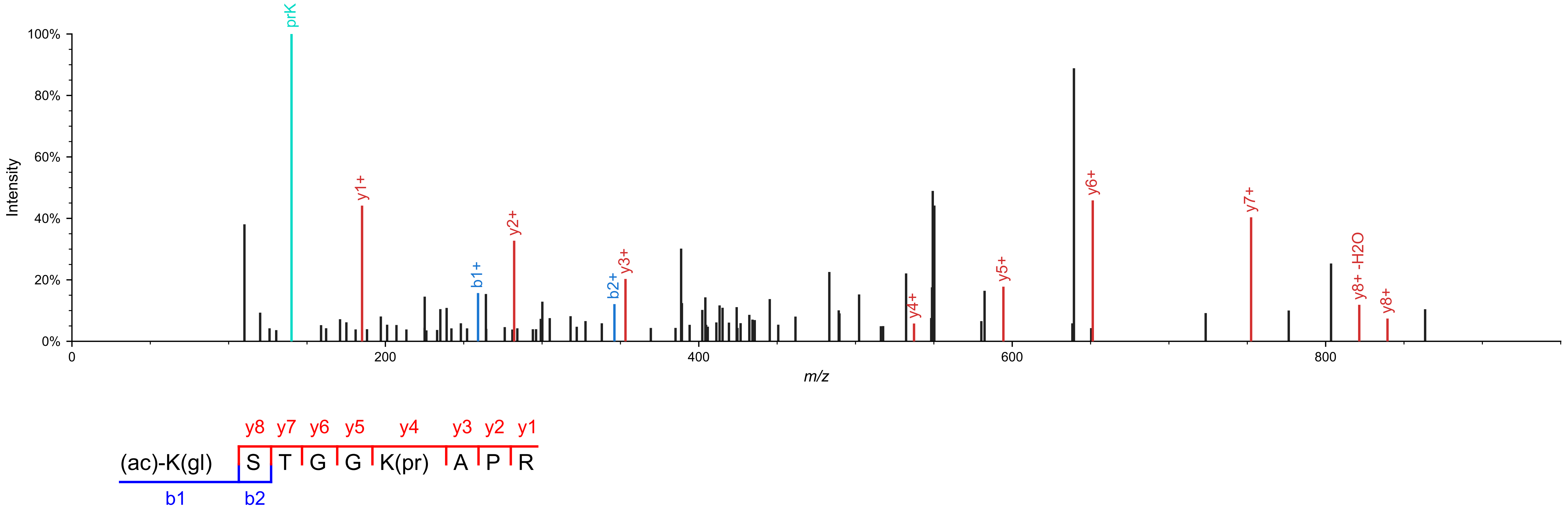

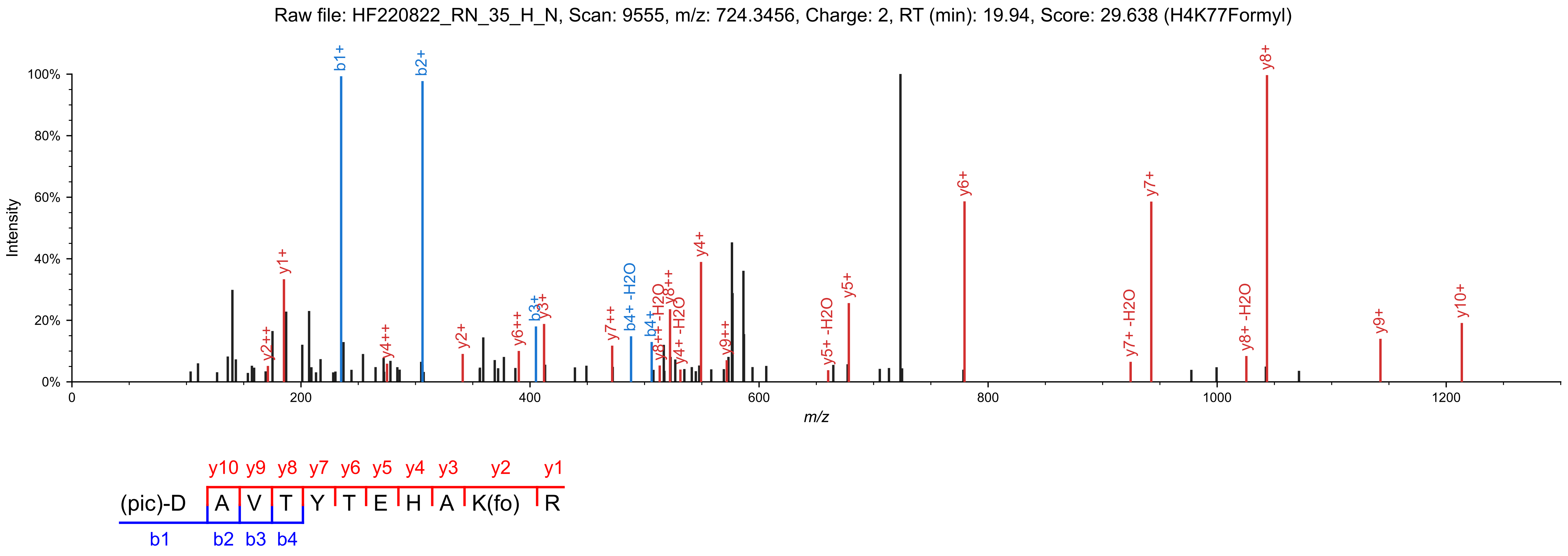

Raw file: HF220822_RN_35_H_N, Scan: 9555, m/z: 724.3456, Charge: 2, RT (min): 19.94, Score: 29.638 (H4K77Formyl)
Intensity
m/z
y10 y9 y8 y7 y6 y5 y4 y3 y2 y1
(pic)-D A V T Y T E H A K(fo) R
b1 b2 b3 b4

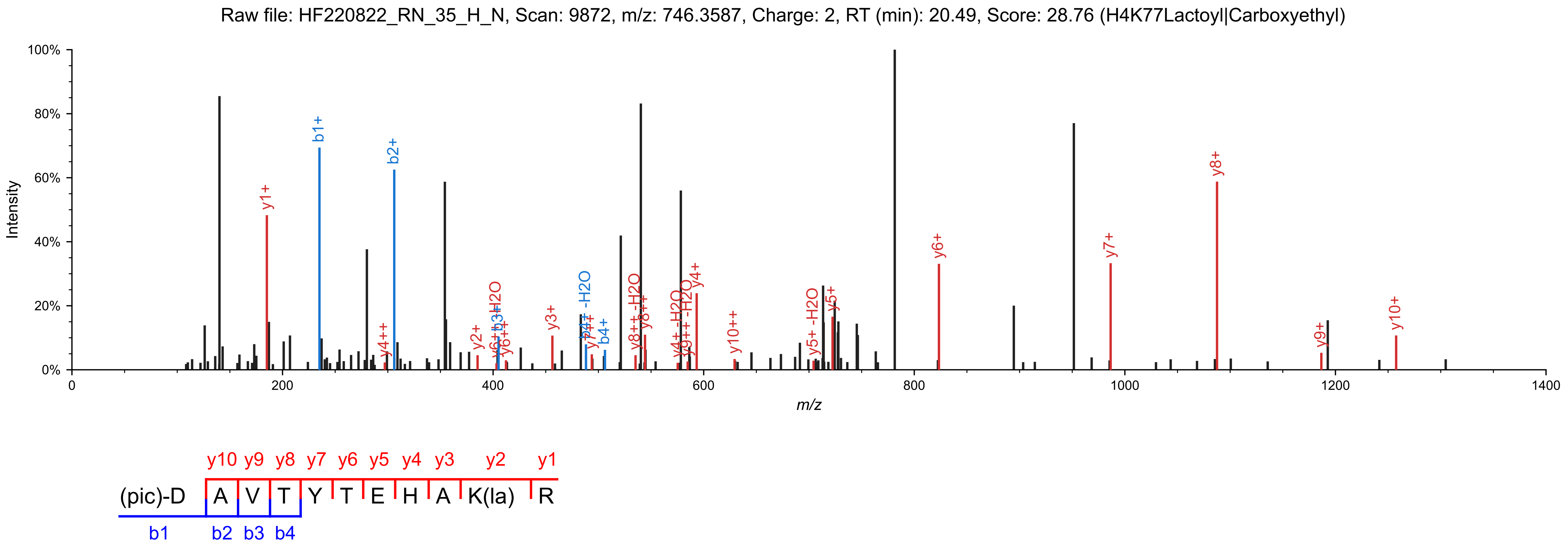

Raw file: HF220822_RN_35_H_N, Scan: 9872, m/z: 746.3587, Charge: 2, RT (min): 20.49, Score: 28.76 (H4K77Lactoyl|Carboxyethyl)
Intensity
m/z
y1+
b1+
b2+
y4++
y2+
y3+
b4+
y6+
y7+
y8+
y9+
y10+
y4+
y5+
y10++
y8++
y5+ -H2O
y8++ -H2O
y4+ -H2O
y9++ -H2O
(pic)-D A V T Y T E H A K(la) R
y10 y9 y8 y7 y6 y5 y4 y3 y2 y1
b1 b2 b3 b4

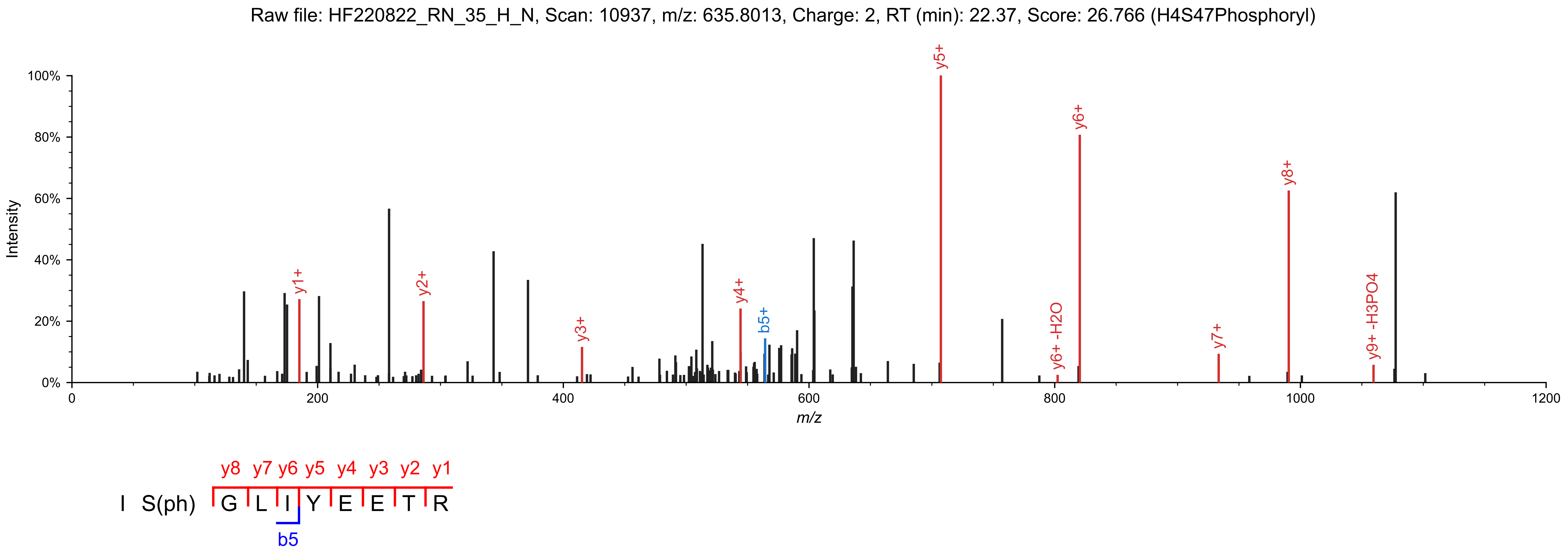
Raw file: HF220822_RN_35_H_N, Scan: 10937, m/z: 635.8013, Charge: 2, RT (min): 22.37, Score: 26.766 (H4S47Phosphoryl)
Intensity
100%
80%
60%
40%
20%
0%
0
200
400
600
800
1000
1200
m/z
y1+
y2+
y3+
y4+
b5+
y5+
y6+ -H2O
y6+
y7+
y8+
y9+ -H3PO4
y8 y7 y6 y5 y4 y3 y2 y1
I S(ph) G L I Y E E T R
b5

Raw file: HF220822_RN_35_H_N, Scan: 14678, m/z: 566.3296, Charge: 2, RT (min): 29.05, Score: 19.406 (H2B1MK24Formyl)

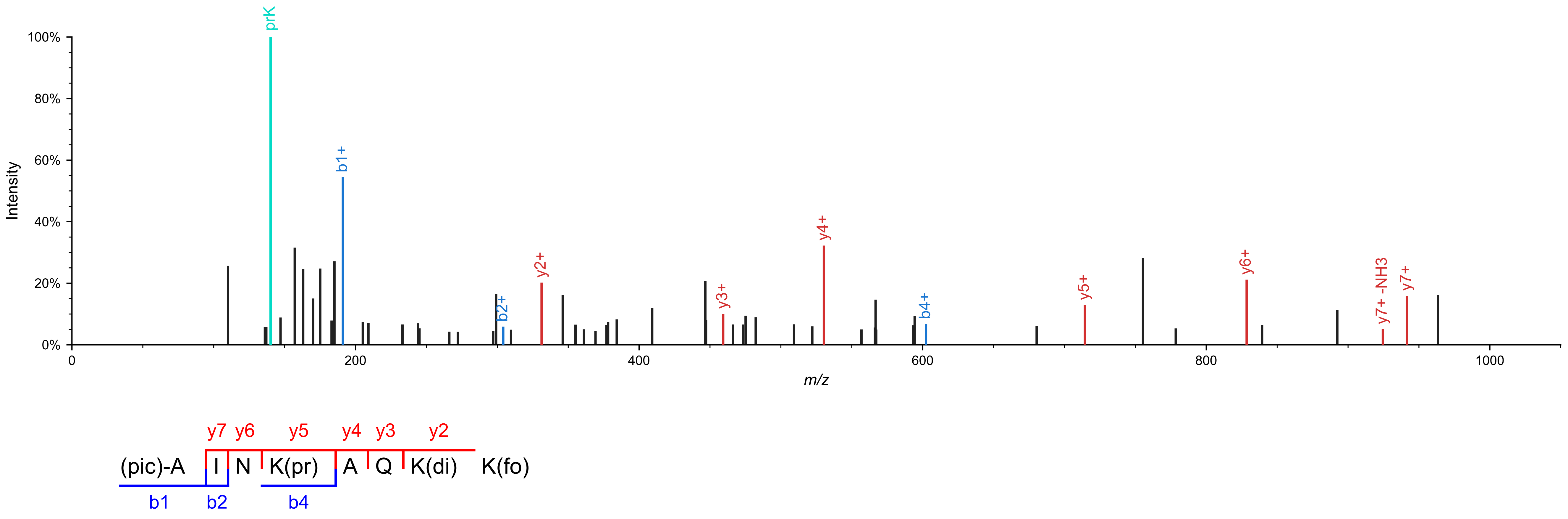

Raw file: HF220822_RN_35_H_N, Scan: 17315, m/z: 615.8638, Charge: 2, RT (min): 33.78, Score: 21.843 (H31K18N-Pentosyl Lysine)

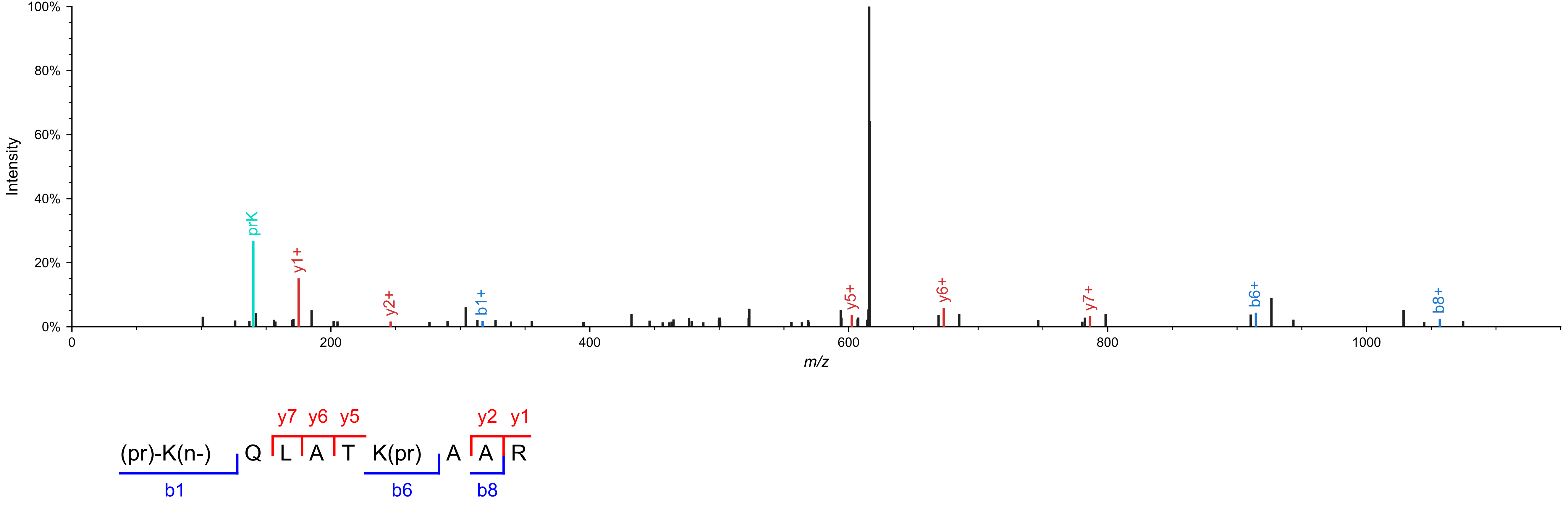

Raw file: HF220822_RN_35_H_N, Scan: 18357, m/z: 622.3552, Charge: 2, RT (min): 35.69, Score: 21.44 (H31K23Lactoyl|Carboxyethyl)

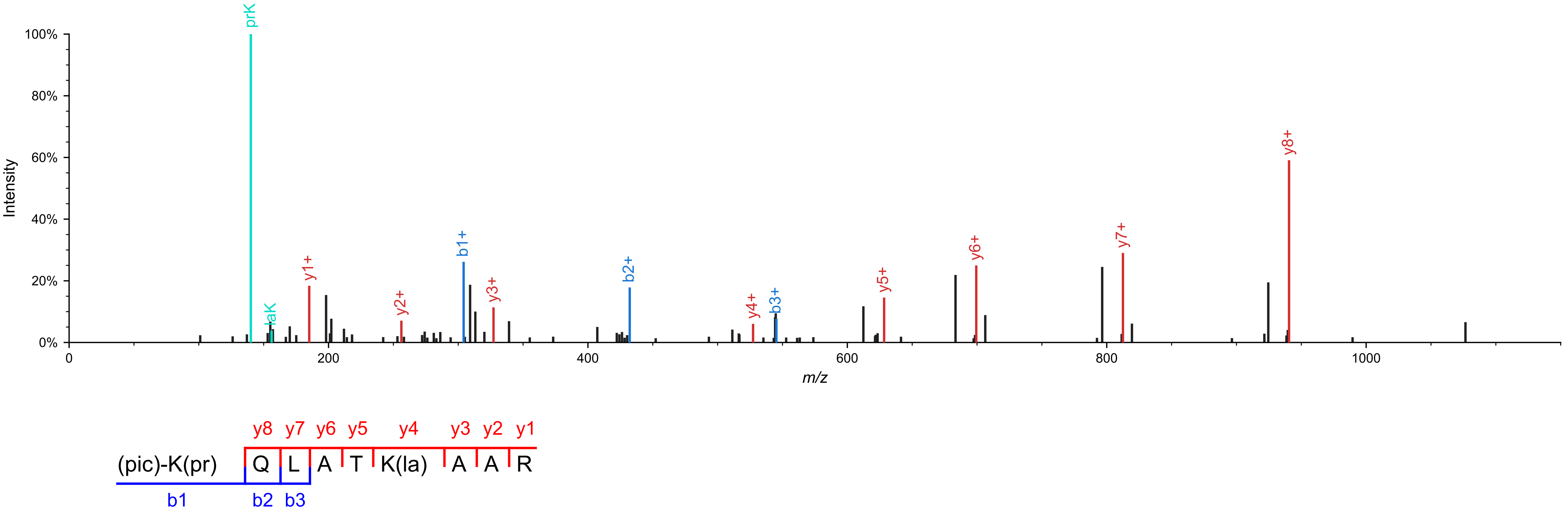

Raw file: HF220822_RN_35_H_N, Scan: 19109, m/z: 437.1907, Charge: 2, RT (min): 37.07, Score: 13.566 (H2B1BT88Phosphoryl)

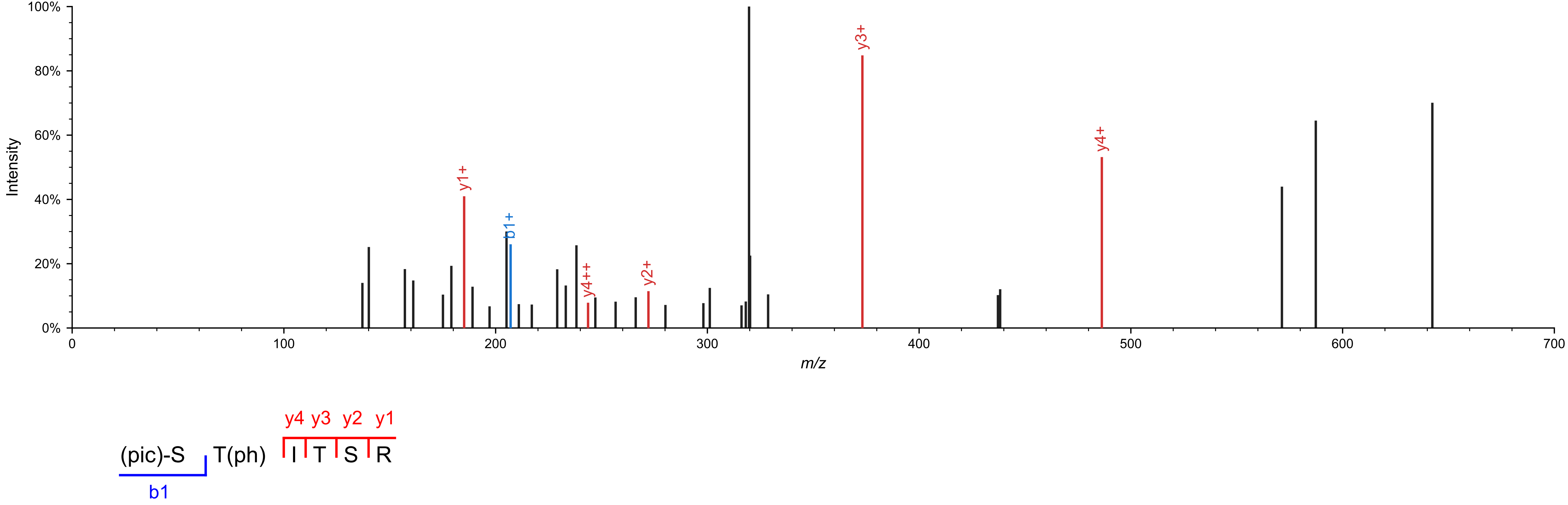

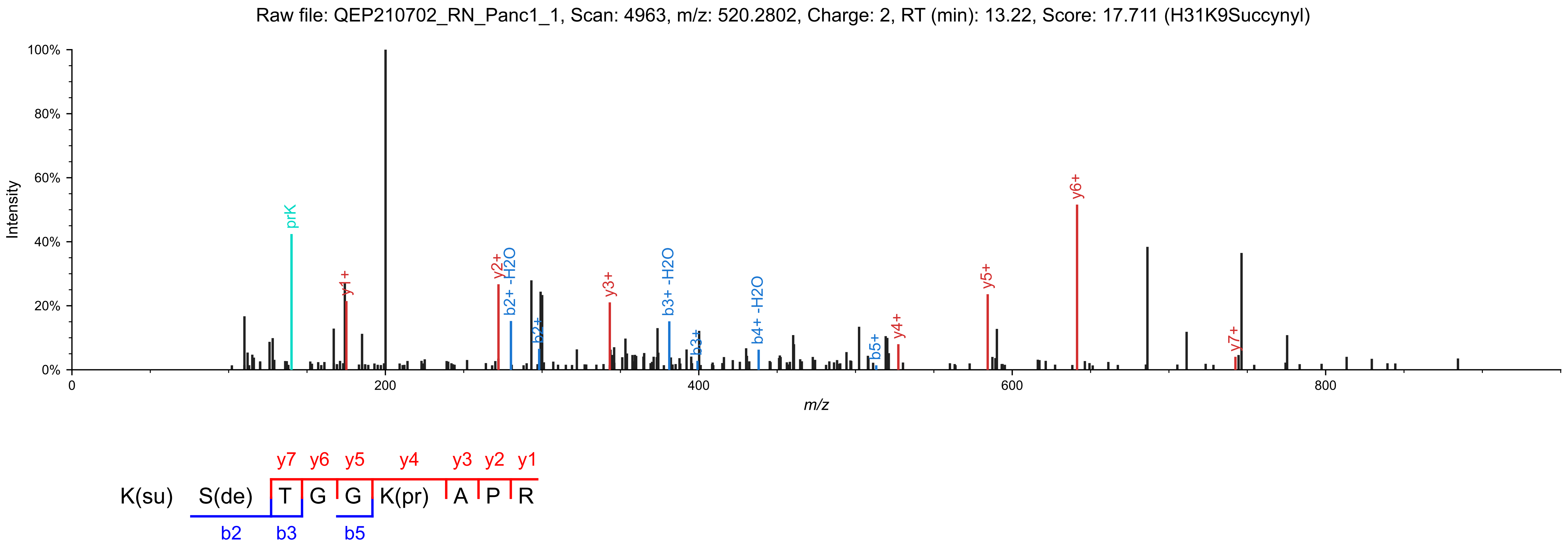

Raw file: QEP210702_RN_Panc1_1, Scan: 4963, m/z: 520.2802, Charge: 2, RT (min): 13.22, Score: 17.711 (H31K9Succynyl)
Intensity
100%
80%
60%
40%
20%
0%
0
200
400
600
800
m/z
prK
y1+
y2+
b2+ -H2O
b2+
y3+
b3+ -H2O
b3+
b4+ -H2O
b5+
y4+
y5+
y6+
y7+
y7 y6 y5 y4 y3 y2 y1
K(su) S(de) T G G K(pr) A P R
b2 b3 b5

Raw file: QEP210702_RN_Panc1_1, Scan: 5763, m/z: 543.8063, Charge: 2, RT (min): 14.83, Score: 23.33 (H31R42MG-H1)

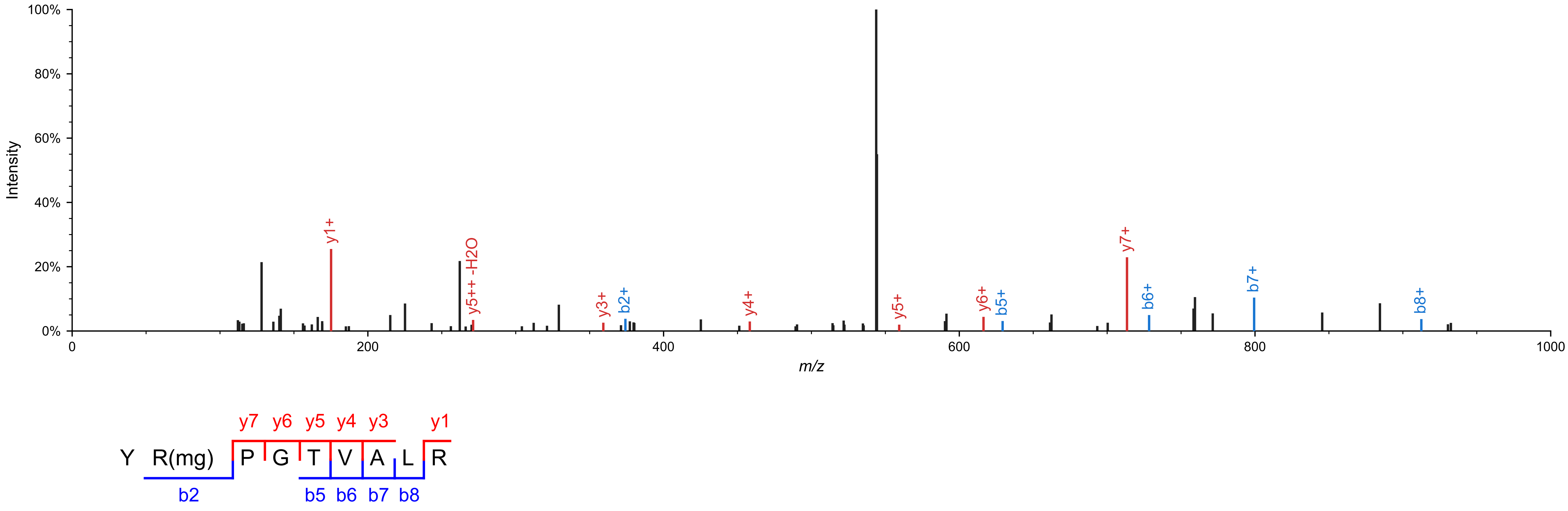

Raw file: QEP210702_RN_Panc1_1, Scan: 7928, m/z: 599.843, Charge: 2, RT (min): 19.16, Score: 16.793 (H31K18Succynyl)

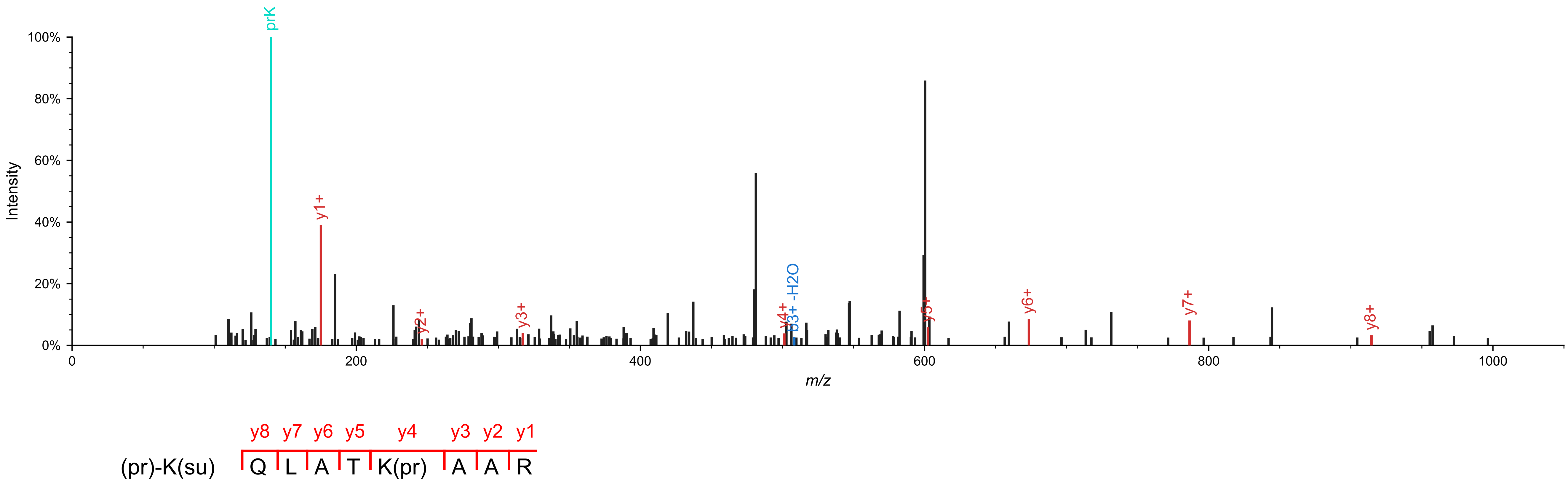

Raw file: QEP210702_RN_Panc1_1, Scan: 15918, m/z: 555.8351, Charge: 2, RT (min): 34.98, Score: 22.621 (H31K18Crotonyl)

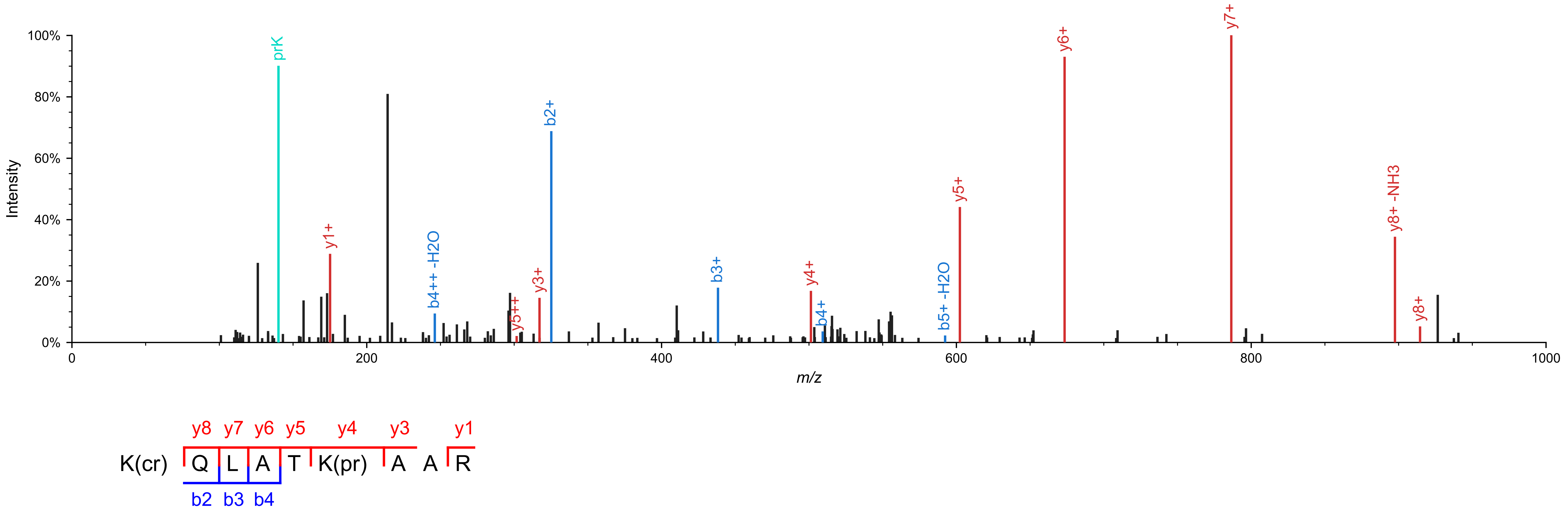

Raw file: QEP210702_RN_Panc1_1, Scan: 17088, m/z: 631.3485, Charge: 2, RT (min): 37.35, Score: 21.587 (H31K23Succynyl)

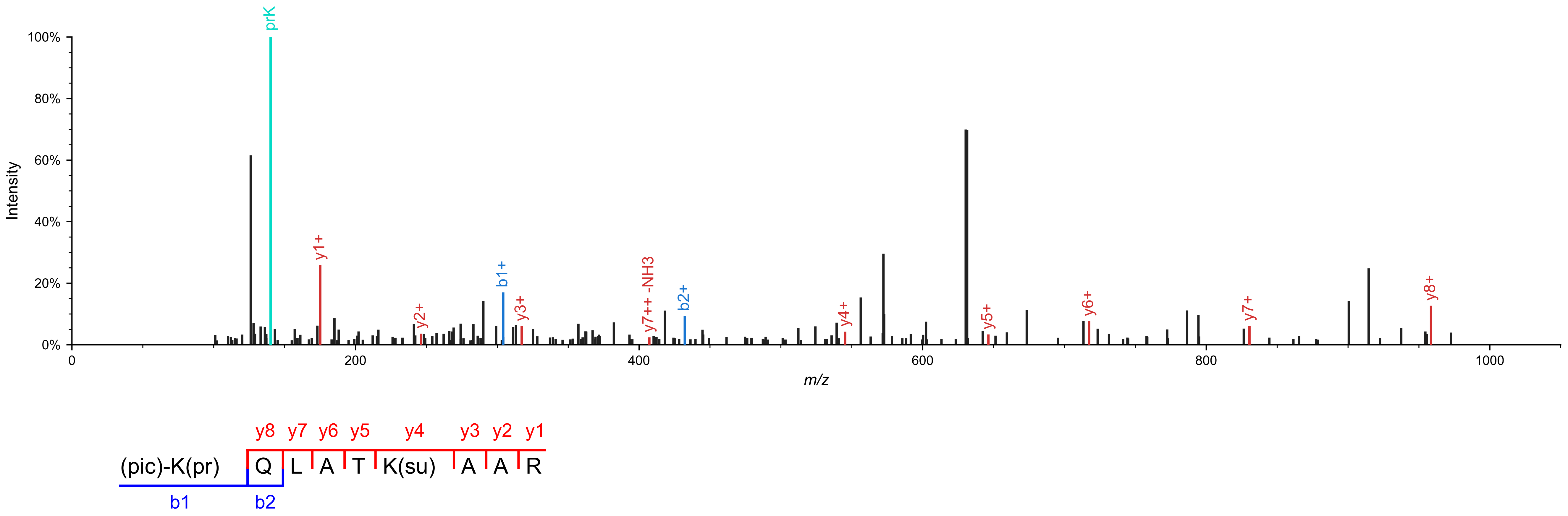

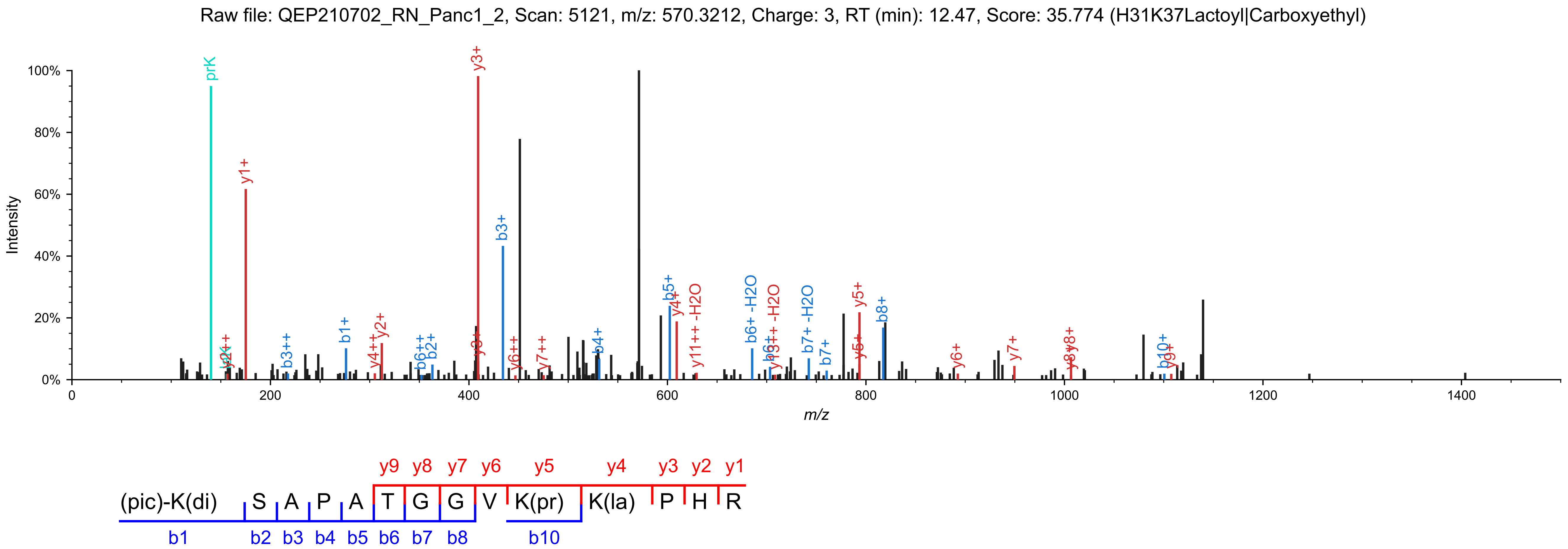

Raw file: QEP210702_RN_Panc1_2, Scan: 5121, m/z: 570.3212, Charge: 3, RT (min): 12.47, Score: 35.774 (H31K37Lactoyl|Carboxyethyl)
Intensity
m/z
0%
20%
40%
60%
80%
100%
0
200
400
600
800
1000
1200
1400
prK
y1+
y3+
b3+
b5+
y4+
y11++ -H2O
b6+ -H2O
y13++ -H2O
b7+ -H2O
y5+
b8+
b1+
y2+
b3++
b6++
b2+
b4+
y6+
y7+
y8+
b10+
y9+
(pic)-K(di) S A P A T G G V K(pr) K(la) P H R
y9 y8 y7 y6 y5 y4 y3 y2 y1
b1 b2 b3 b4 b5 b6 b7 b8 b10

Raw file: QEP210702_RN_Panc1_2, Scan: 5314, m/z: 851.4677, Charge: 2, RT (min): 12.81, Score: 27.89 (H31K27Succynyl)

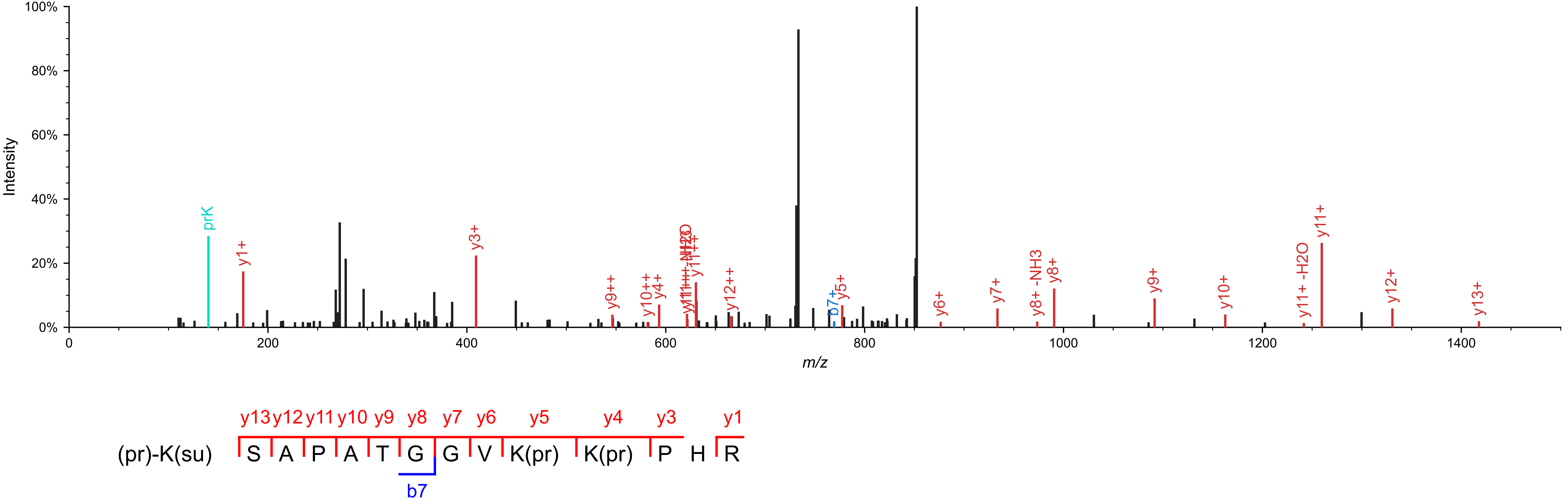

Raw file: QEP210702_RN_Panc1_2, Scan: 5812, m/z: 533.9703, Charge: 3, RT (min): 13.70, Score: 36.145 (H31K27Lactoyl|Carboxyethyl)

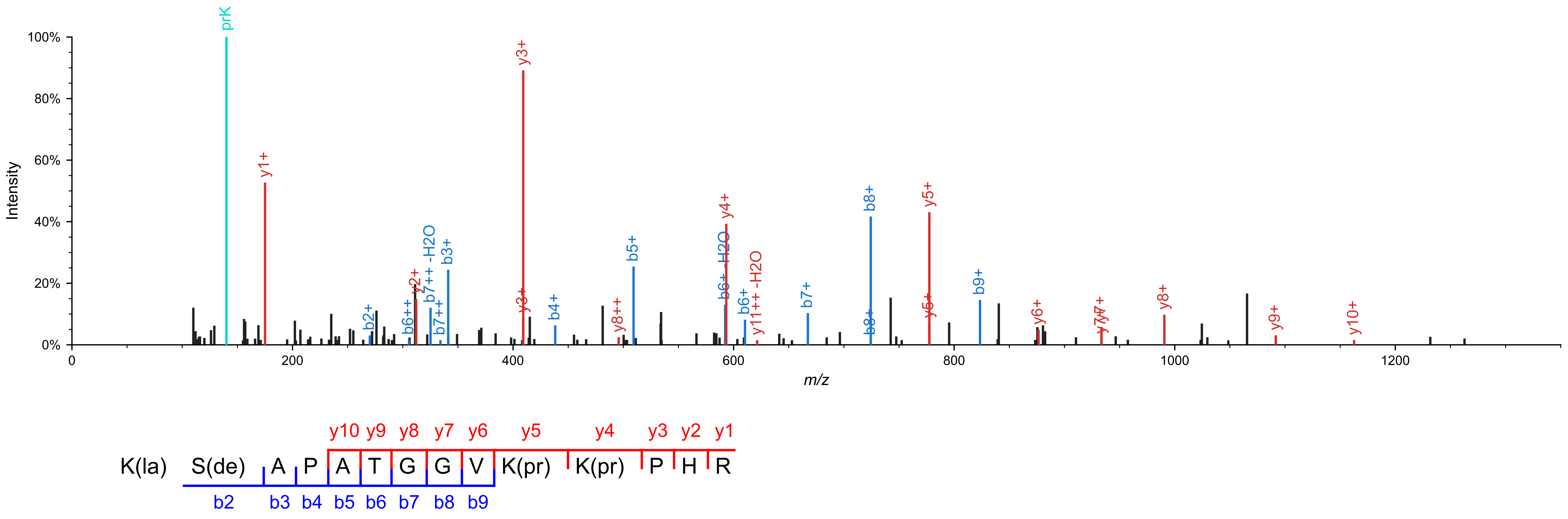

Raw file: QEP210702_RN_Panc1_2, Scan: 11405, m/z: 595.8118, Charge: 2, RT (min): 24.06, Score: 14.914 (H31K9Glutaryl)

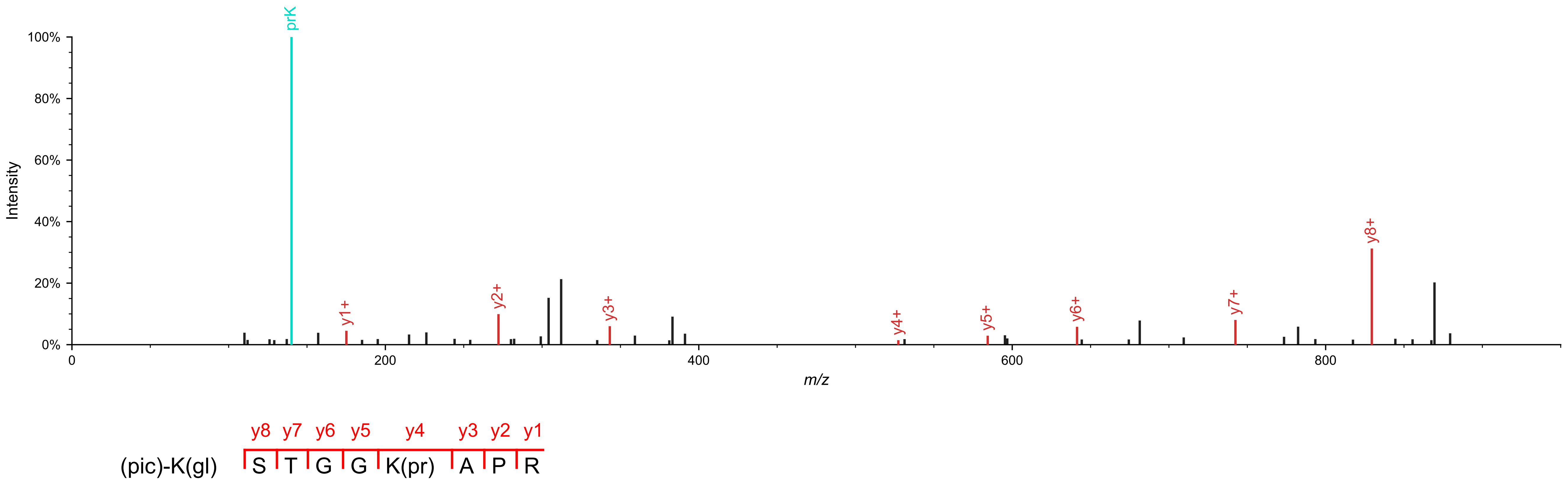

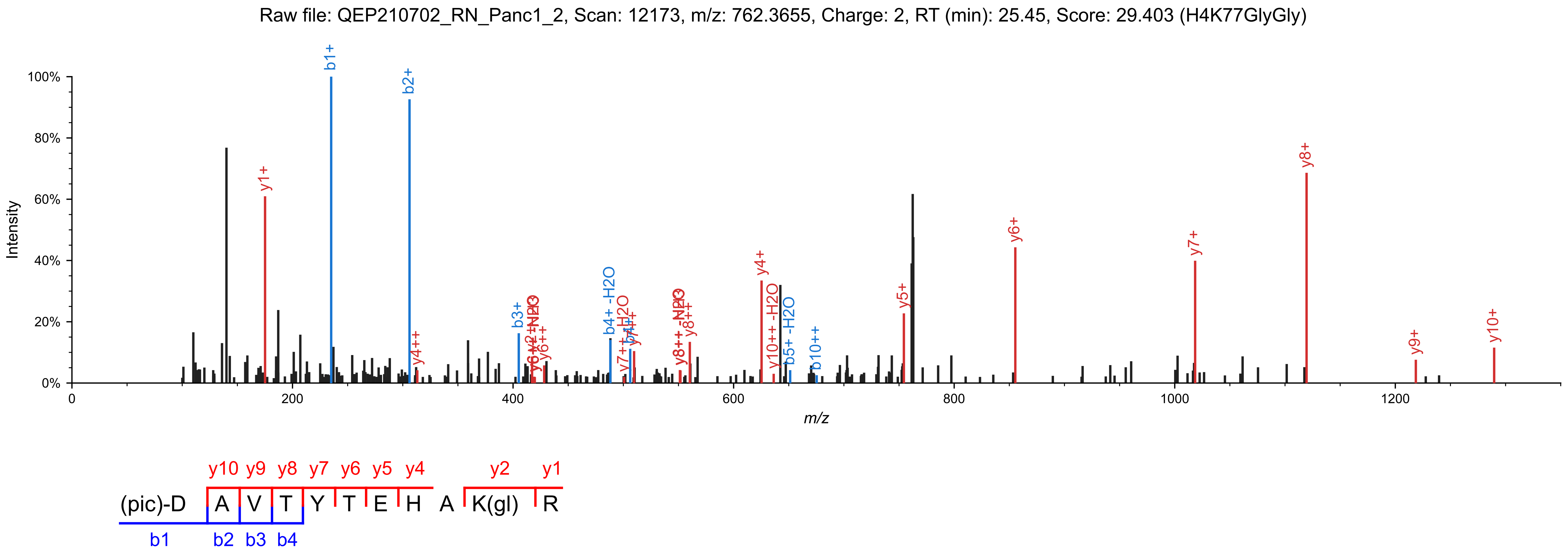

Raw file: QEP210702_RN_Panc1_2, Scan: 12173, m/z: 762.3655, Charge: 2, RT (min): 25.45, Score: 29.403 (H4K77GlyGly)
Intensity
100%
80%
60%
40%
20%
0%
0
200
400
600
800
1000
1200
m/z
b1+
b2+
y1+
y4++
b3+
y6++
b4+ -H2O
y4+
y10++ -H2O
b5+ -H2O
b10++
y5+
y6+
y7+
y8+
y9+
y10+
y8++
y10 y9 y8 y7 y6 y5 y4 y2 y1
(pic)-D A V T Y T E H A K(gl) R
b1 b2 b3 b4

Raw file: QEP210702_RN_Panc1_2, Scan: 21961, m/z: 774.4161, Charge: 2, RT (min): 43.96, Score: 28.756 (H31K122Formyl)

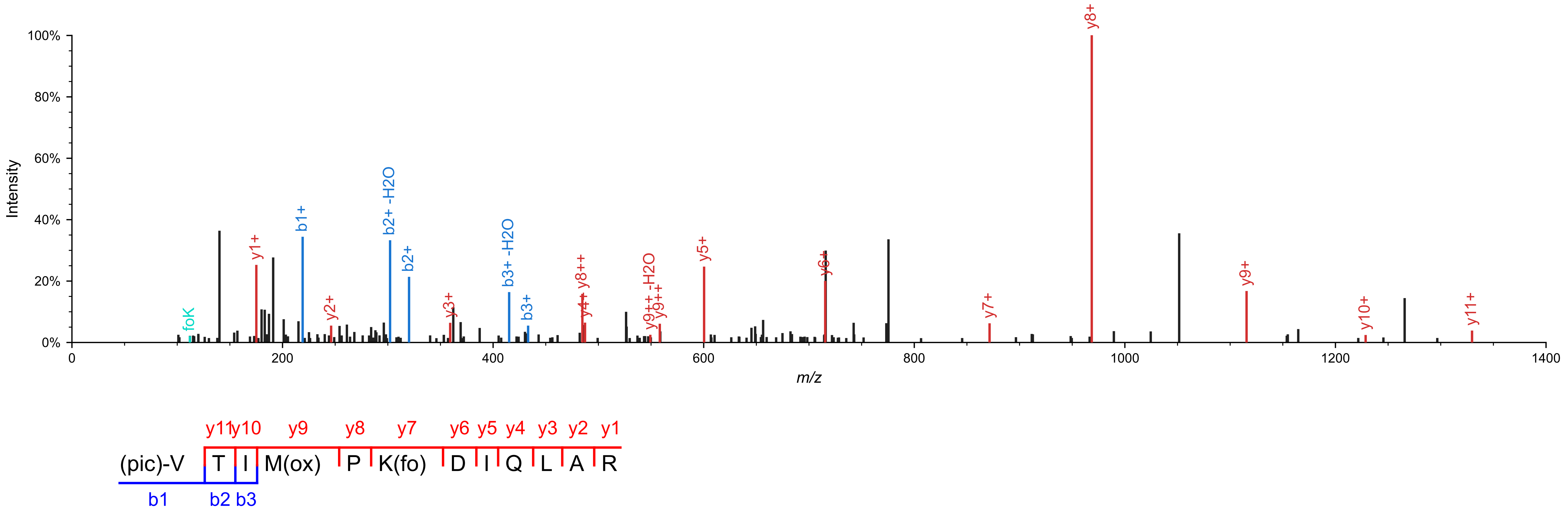

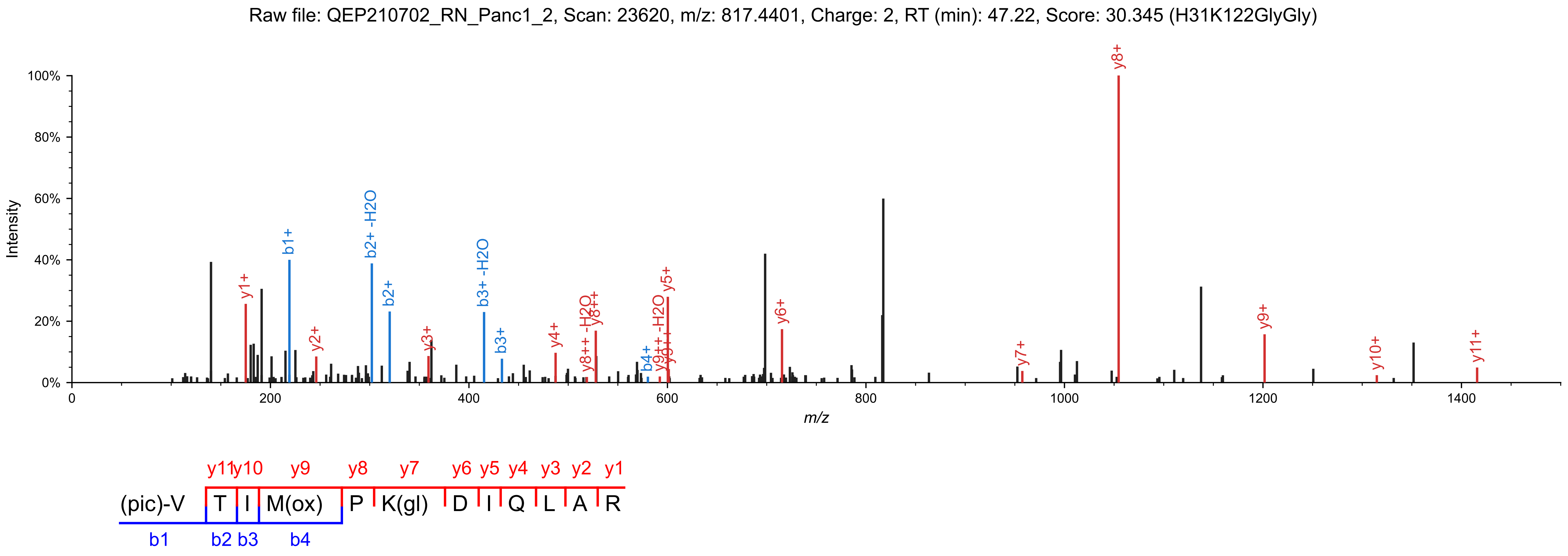
Raw file: QEP210702_RN_Panc1_2, Scan: 23620, m/z: 817.4401, Charge: 2, RT (min): 47.22, Score: 30.345 (H31K122GlyGly)
Intensity
100%
80%
60%
40%
20%
0%
0
200
400
600
800
1000
1200
1400
m/z
y8+
b2+ -H2O
b1+
b3+ -H2O
y1+
b2+
y5+
y6+
y9+
y2+
y3+
b3+
y4+
y8++
b4+
y7+
y10+
y11+
y11 y10 y9 y8 y7 y6 y5 y4 y3 y2 y1
(pic)-V T I M(ox) P K(gl) D I Q L A R
b1 b2 b3 b4

Raw file: QEP210702_RN_Panc1_3, Scan: 1612, m/z: 509.2894, Charge: 2, RT (min): 6.68, Score: 20.548 (H31K9Crotonyl)

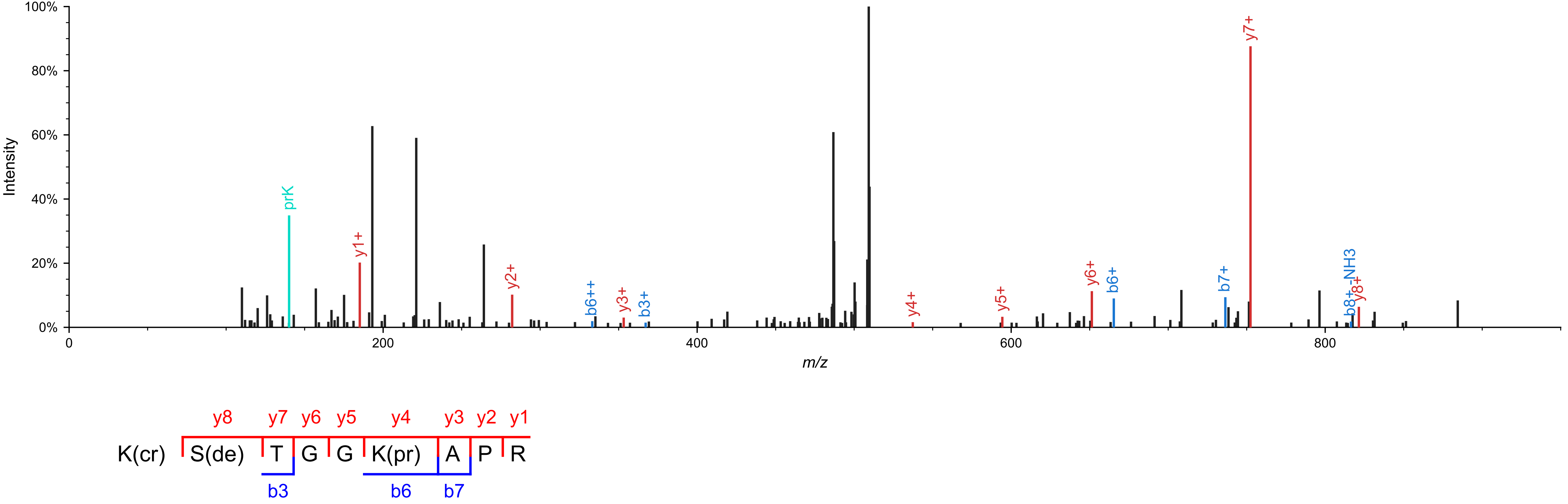

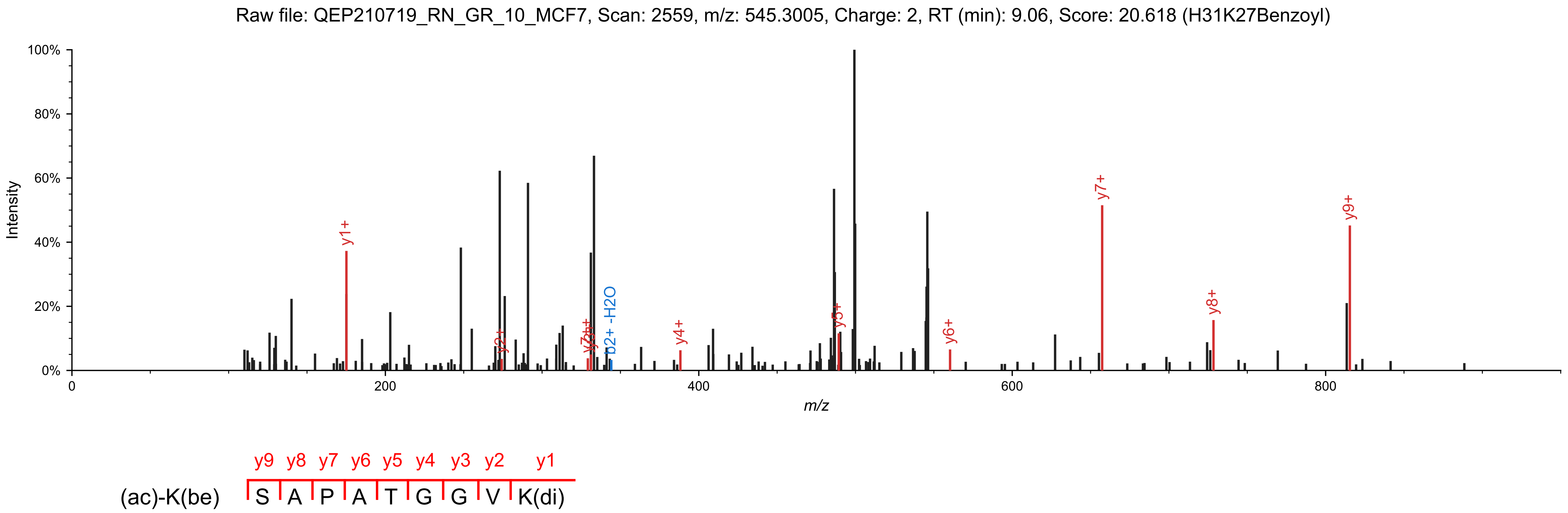

Raw file: QEP210719_RN_GR_10_MCF7, Scan: 2559, m/z: 545.3005, Charge: 2, RT (min): 9.06, Score: 20.618 (H31K27Benzoyl)
Intensity
100%
80%
60%
40%
20%
0%
0
200
400
600
800
m/z
y1+
y2+
b2+ -H2O
y4+
y5+
y6+
y7+
y8+
y9+
y9 y8 y7 y6 y5 y4 y3 y2 y1
(ac)-K(be) S A P A T G G V K(di)

Raw file: QEP210719_RN_GR_10_MCF7, Scan: 11429, m/z: 441.7572, Charge: 2, RT (min): 27.41, Score: 15.404 (H31K64Formyl)

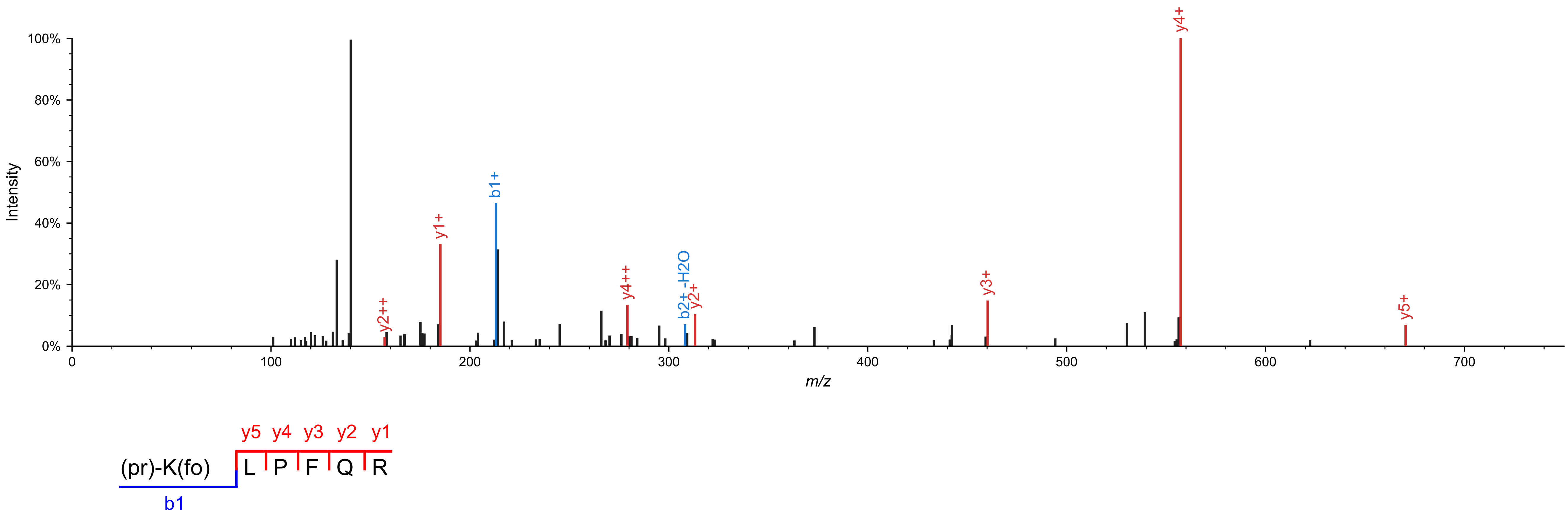

Raw file: QEP210719_RN_GR_11_MCF7, Scan: 7480, m/z: 538.796, Charge: 2, RT (min): 19.79, Score: 14.567 (H2AJK9Lactoyl|Carboxyethyl)

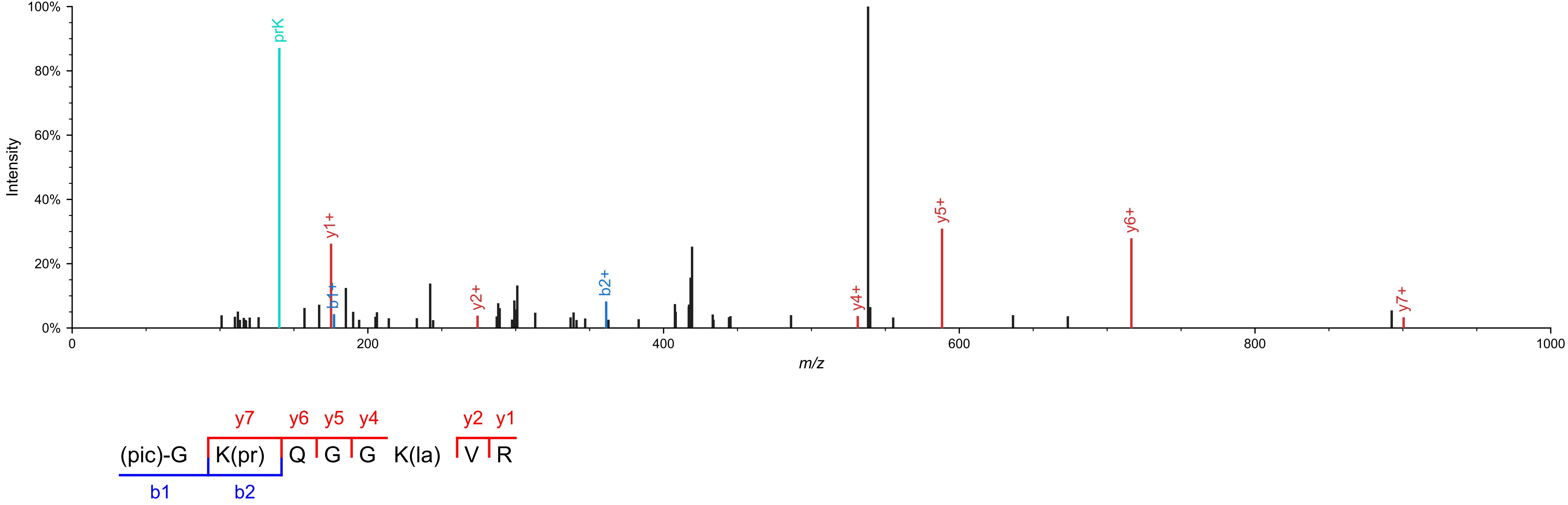

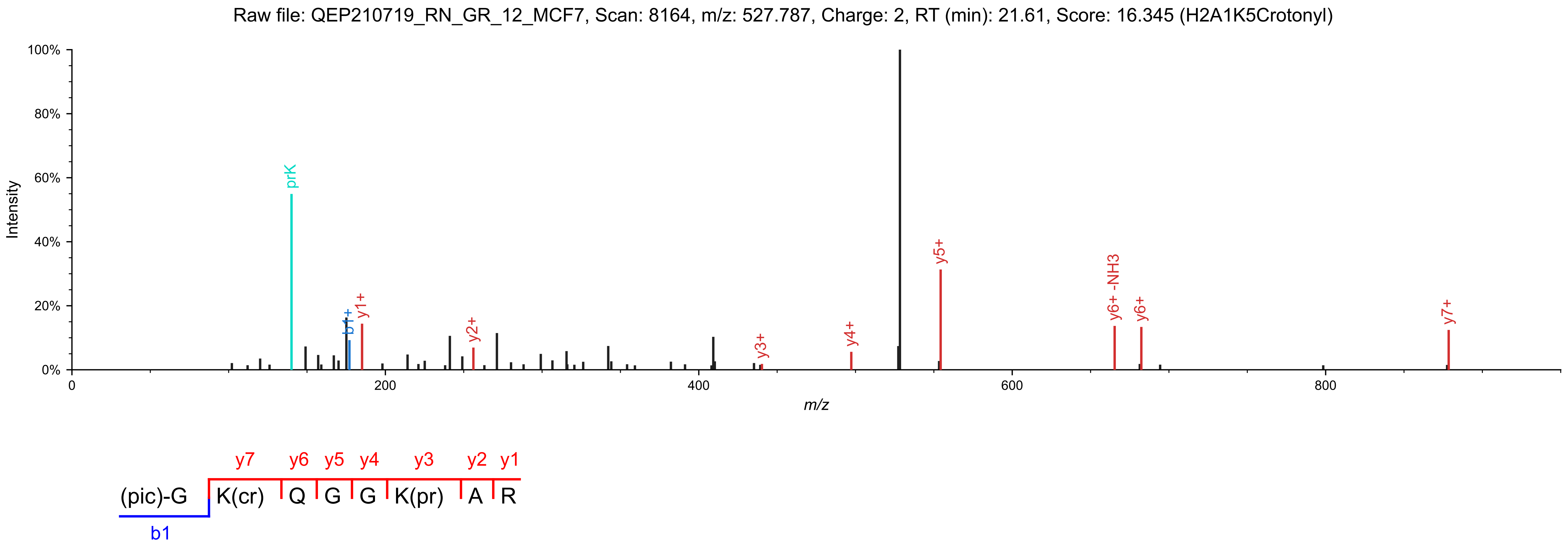

Raw file: QEP210719_RN_GR_12_MCF7, Scan: 8164, m/z: 527.787, Charge: 2, RT (min): 21.61, Score: 16.345 (H2A1K5Crotonyl)
Intensity
100%
80%
60%
40%
20%
0%
0
200
400
600
800
m/z
prK
b1+
y1+
y2+
y3+
y4+
y5+
y6+ -NH3
y6+
y7+
y7 y6 y5 y4 y3 y2 y1
(pic)-G K(cr) Q G G K(pr) A R
b1

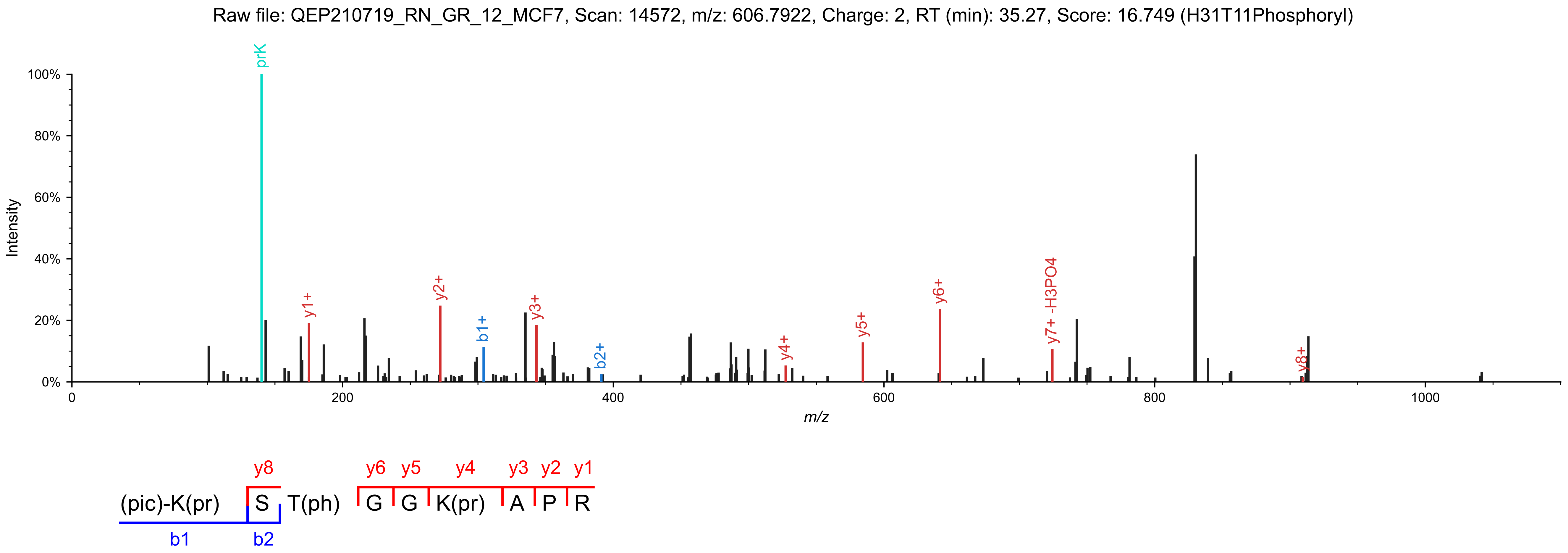

Raw file: QEP210719_RN_GR_12_MCF7, Scan: 14572, m/z: 606.7922, Charge: 2, RT (min): 35.27, Score: 16.749 (H31T11Phosphoryl)
prK
y1+
y2+
b1+
y3+
b2+
y4+
y5+
y6+
y7+ -H3PO4
y8+
100%
80%
60%
40%
20%
0%
Intensity
0
200
400
600
800
1000
m/z
y8
y6
y5
y4
y3
y2
y1
(pic)-K(pr)
S
T(ph)
G
G
K(pr)
A
P
R
b1
b2

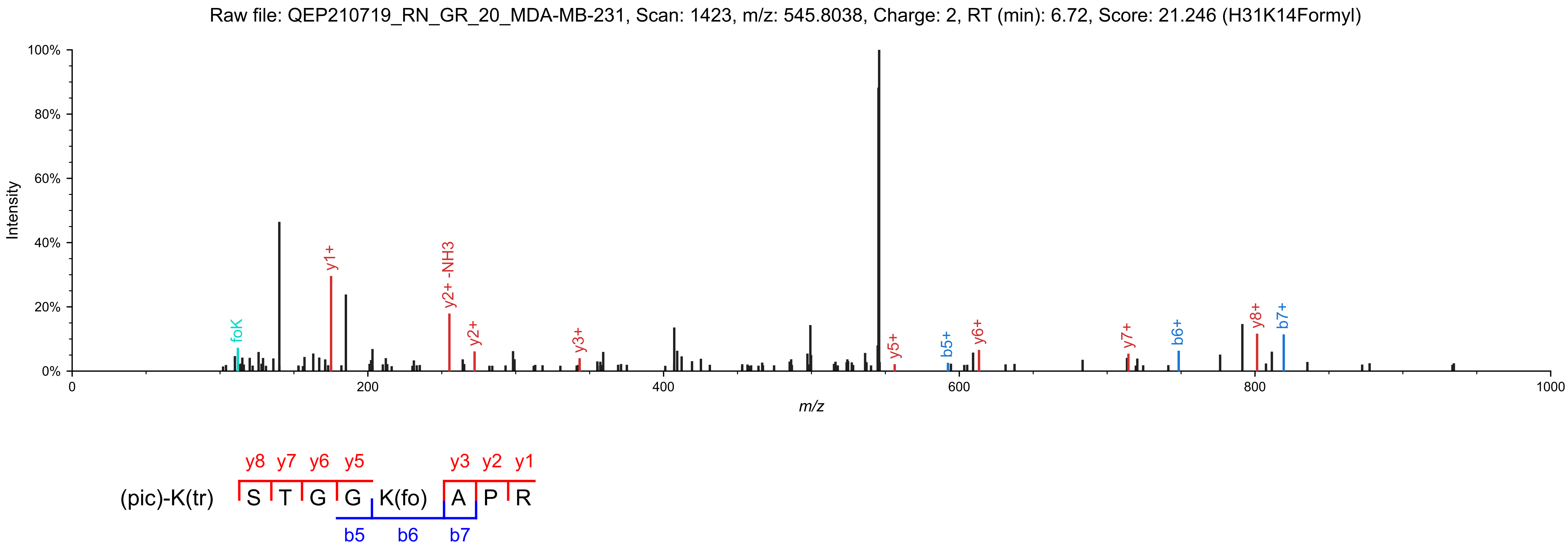
Raw file: QEP210719_RN_GR_20_MDA-MB-231, Scan: 1423, m/z: 545.8038, Charge: 2, RT (min): 6.72, Score: 21.246 (H31K14Formyl)
Intensity
100%
80%
60%
40%
20%
0%
0
200
400
600
800
1000
m/z
foK
y1+
y2+ -NH3
y2+
y3+
y5+
b5+
y6+
y7+
b6+
y8+
b7+
y8 y7 y6 y5 y3 y2 y1
(pic)-K(tr) S T G G K(fo) A P R
b5 b6 b7

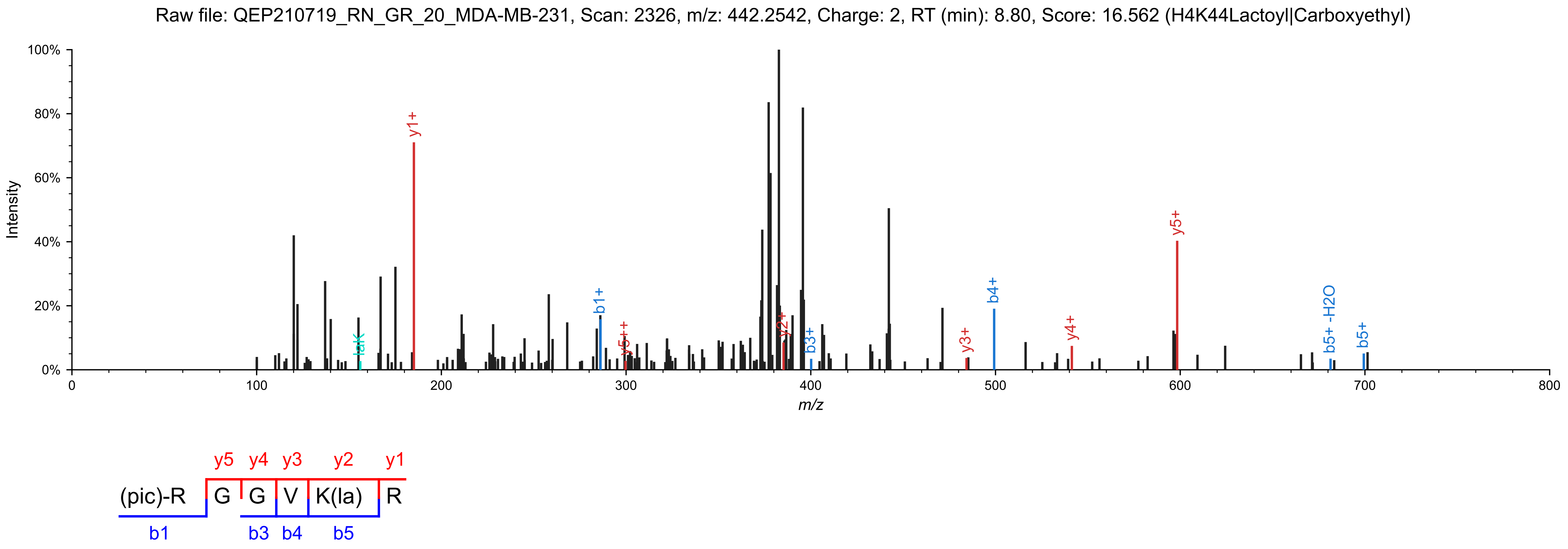

Raw file: QEP210719_RN_GR_20_MDA-MB-231, Scan: 2326, m/z: 442.2542, Charge: 2, RT (min): 8.80, Score: 16.562 (H4K44Lactoyl|Carboxyethyl)
Intensity
100%
80%
60%
40%
20%
0%
0
100
200
300
400
500
600
700
800
m/z
y1+
b1+
y5++
y2+
b3+
y3+
b4+
y4+
y5+
b5+ -H2O
b5+
y5 y4 y3 y2 y1
(pic)-R G G V K(la) R
b1 b3 b4 b5

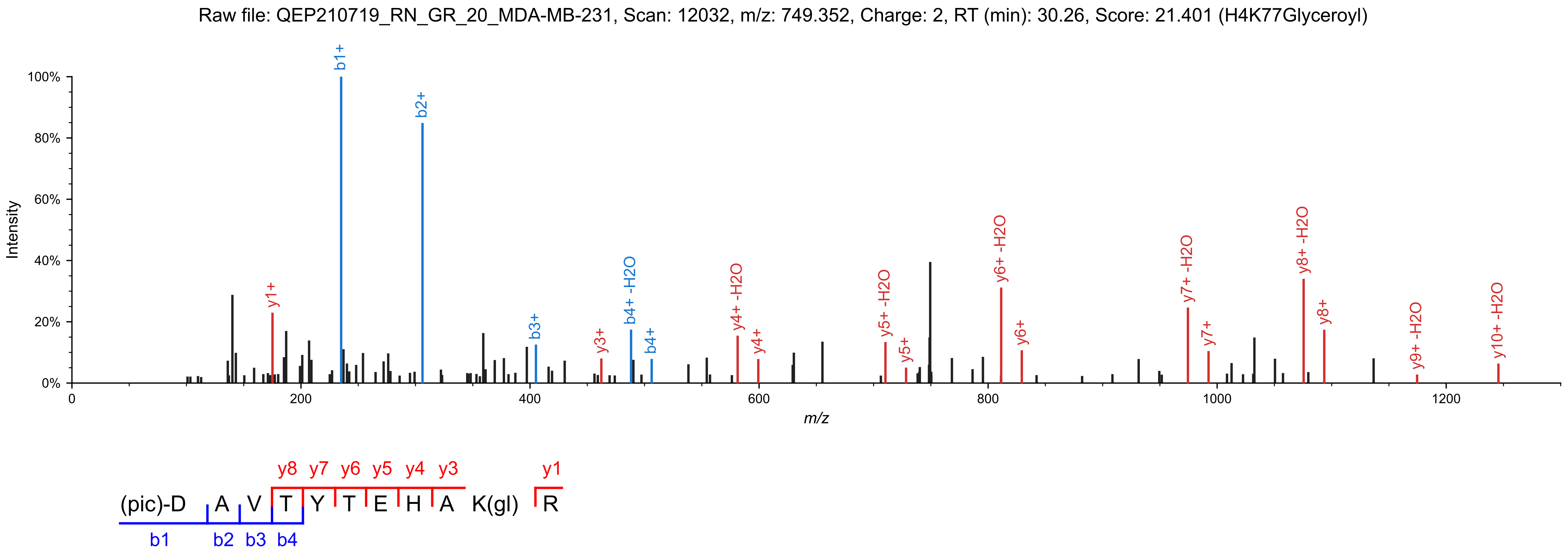

Raw file: QEP210719_RN_GR_20_MDA-MB-231, Scan: 12032, m/z: 749.352, Charge: 2, RT (min): 30.26, Score: 21.401 (H4K77Glyceroyl)
Intensity
100%
80%
60%
40%
20%
0%
0
200
400
600
800
1000
1200
m/z
b1+
b2+
b3+
b4+ -H2O
b4+
y1+
y3+
y4+ -H2O
y4+
y5+ -H2O
y5+
y6+ -H2O
y6+
y7+ -H2O
y7+
y8+ -H2O
y8+
y9+ -H2O
y10+ -H2O
y8 y7 y6 y5 y4 y3 y1
(pic)-D A V T Y T E H A K(gl) R
b1 b2 b3 b4

Raw file: QEP210719_RN_GR_20_MDA-MB-231, Scan: 14724, m/z: 758.9095, Charge: 2, RT (min): 35.92, Score: 19.058 (H4K31Lactoyl|Carboxyethyl)

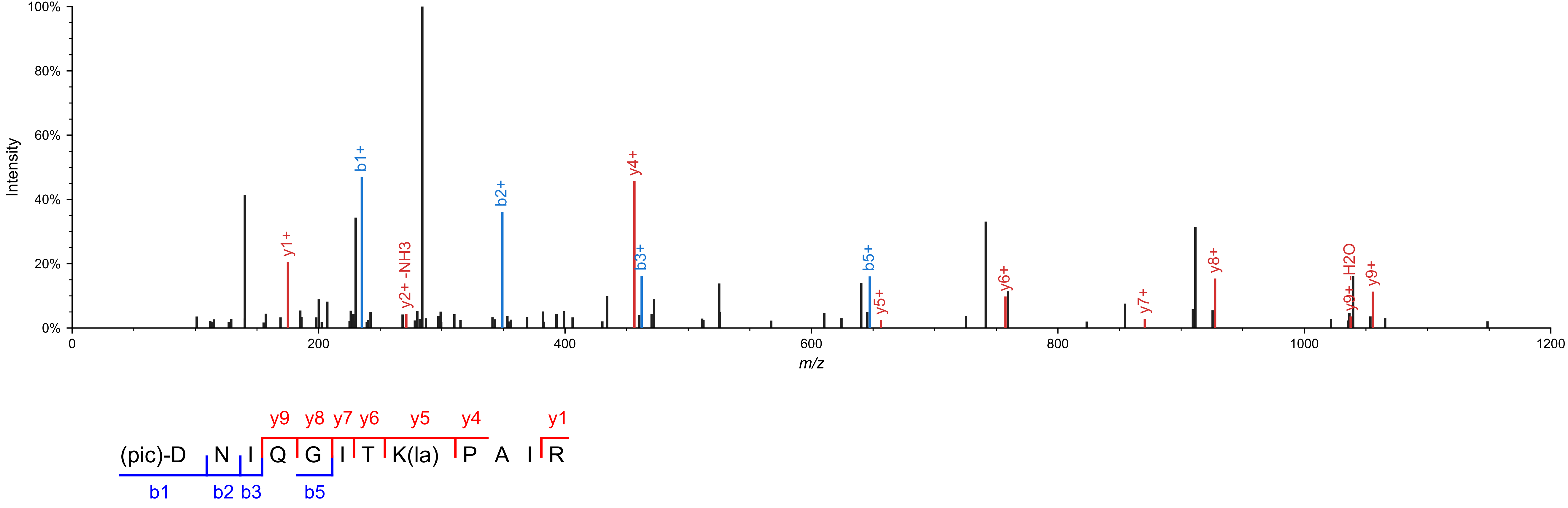

Raw file: QEP210719_RN_GR_21_MDA-MB-231, Scan: 1468, m/z: 556.6379, Charge: 3, RT (min): 6.91, Score: 30.86 (H31S28Phosphoryl)

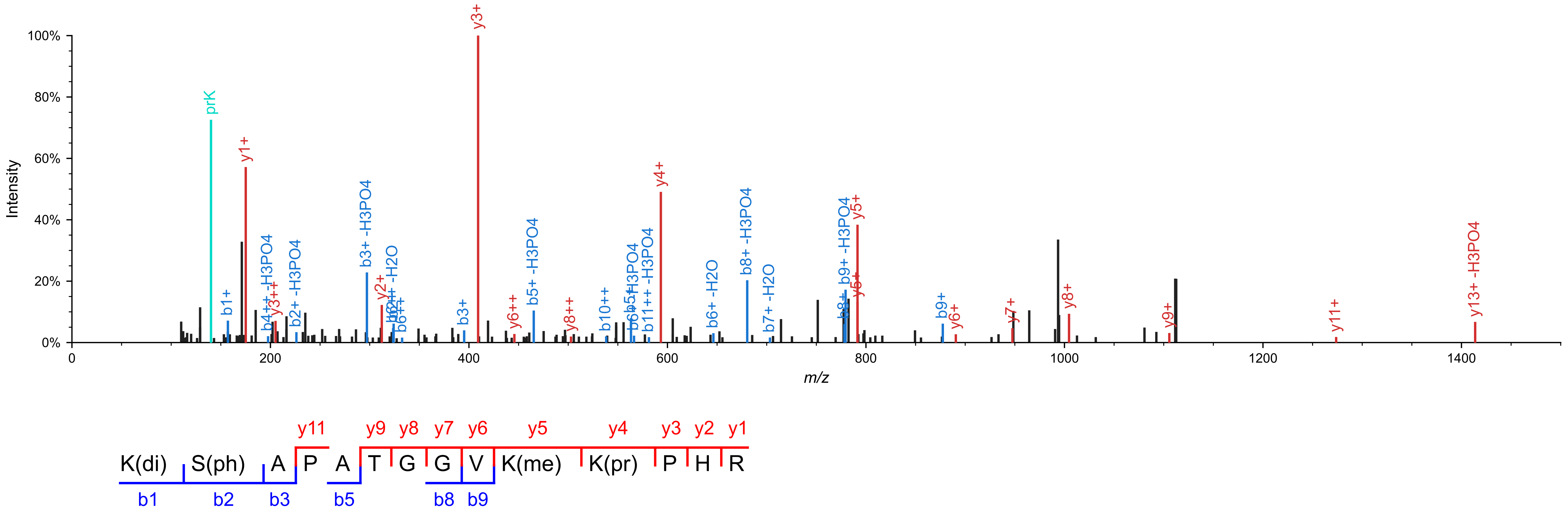

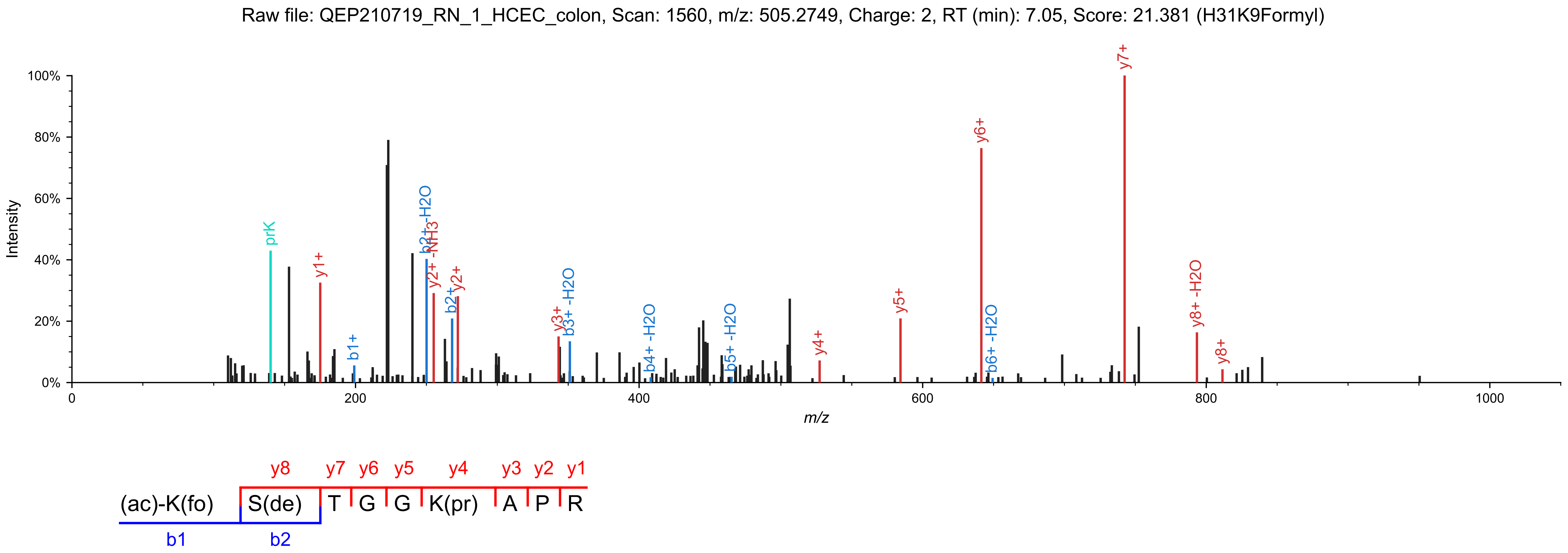
Raw file: QEP210719_RN_1_HCEC_colon, Scan: 1560, m/z: 505.2749, Charge: 2, RT (min): 7.05, Score: 21.381 (H31K9Formyl)
Intensity
100%
80%
60%
40%
20%
0%
0
200
400
600
800
1000
m/z
prK
y1+
b1+
b2+ -H2O
y2+ -NH3
y2+
b2+
y3+
b3+ -H2O
b4+ -H2O
b5+ -H2O
y4+
y5+
y6+
b6+ -H2O
y7+
y8+ -H2O
y8+
y8
y7
y6
y5
y4
y3
y2
y1
(ac)-K(fo)
S(de)
T
G
G
K(pr)
A
P
R
b1
b2

Raw file: QEP210719_RN_1_HCEC_colon, Scan: 11847, m/z: 872.4926, Charge: 2, RT (min): 27.64, Score: 33.326 (H31K27N-Pentosyl Lysine)

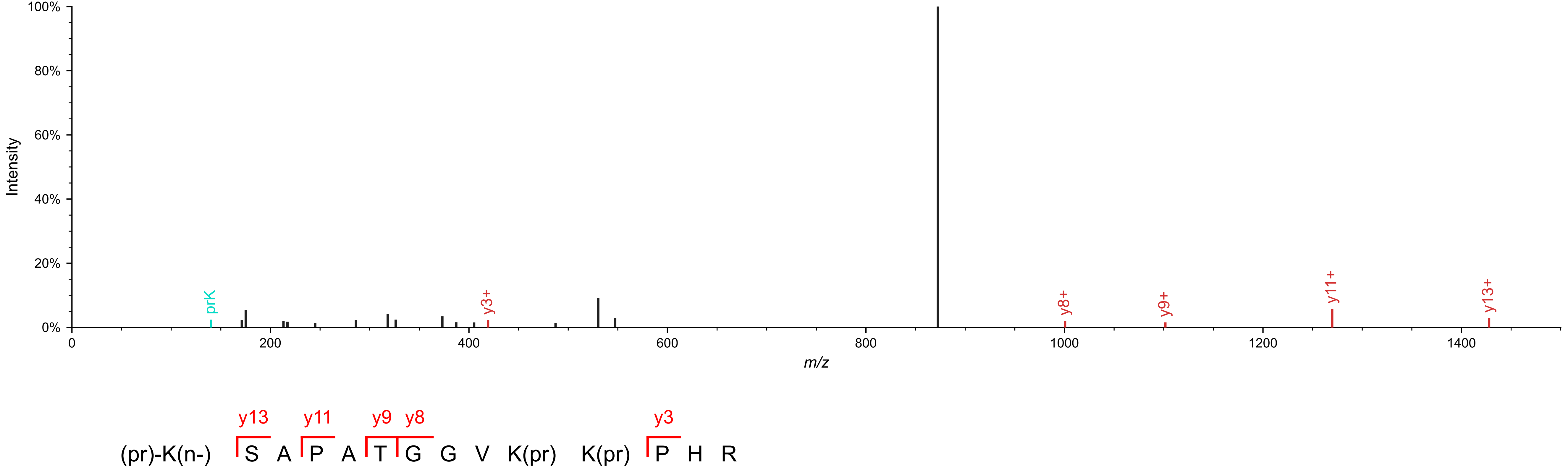

Raw file: QEP210719_RN_1_HCEC_colon, Scan: 15935, m/z: 467.5776, Charge: 3, RT (min): 35.90, Score: 21.039 (H31S57Phosphoryl)

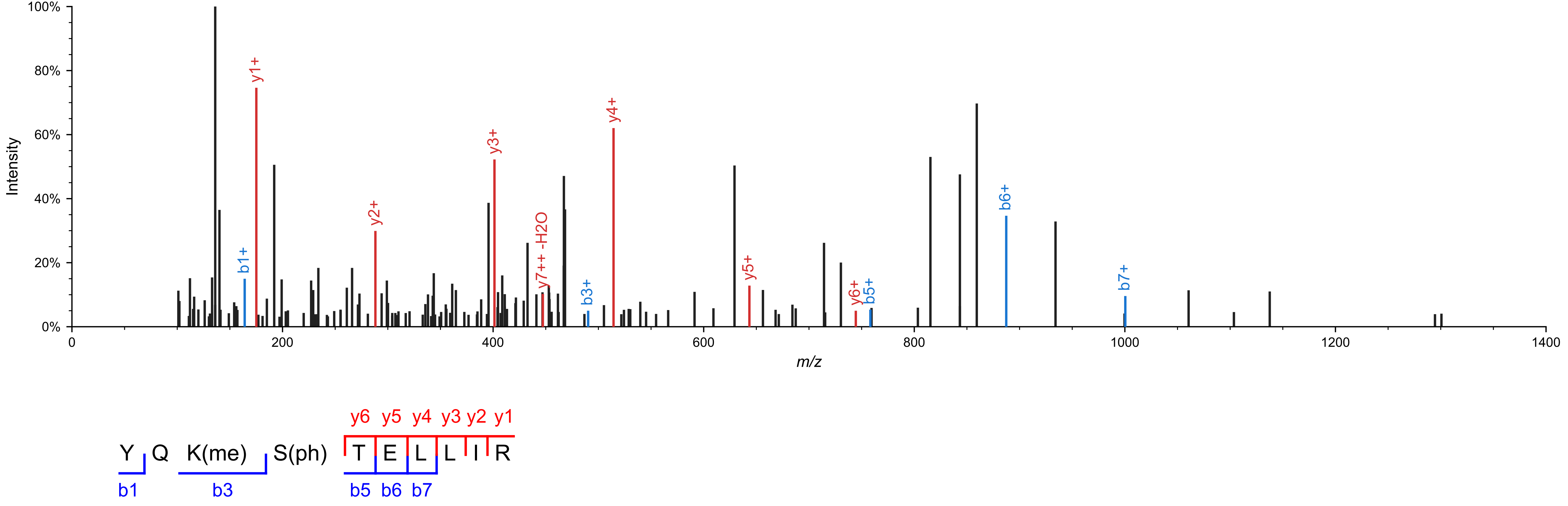

Raw file: QEP210719_RN_2_HCEC_colon, Scan: 7036, m/z: 548.2946, Charge: 2, RT (min): 18.56, Score: 18.31 (H31K9Benzoyl)

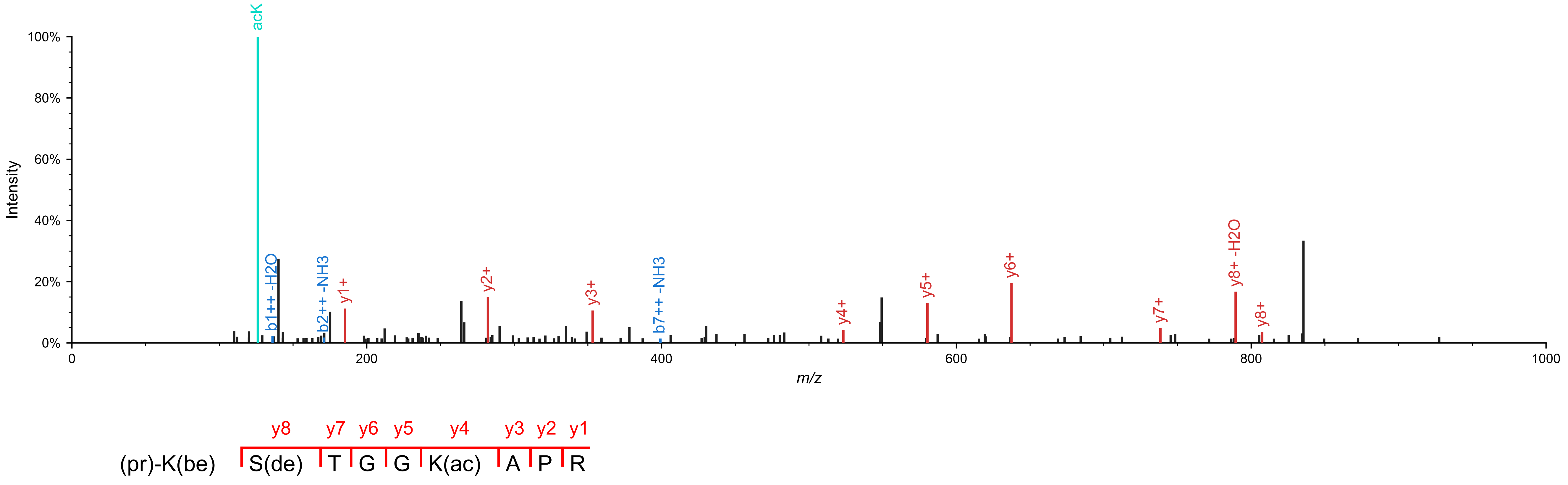

Raw file: QEP210719_RN_3_HCEC_colon, Scan: 10864, m/z: 585.3131, Charge: 2, RT (min): 26.68, Score: 21.214 (H31K9N-Pentosyl Lysine)

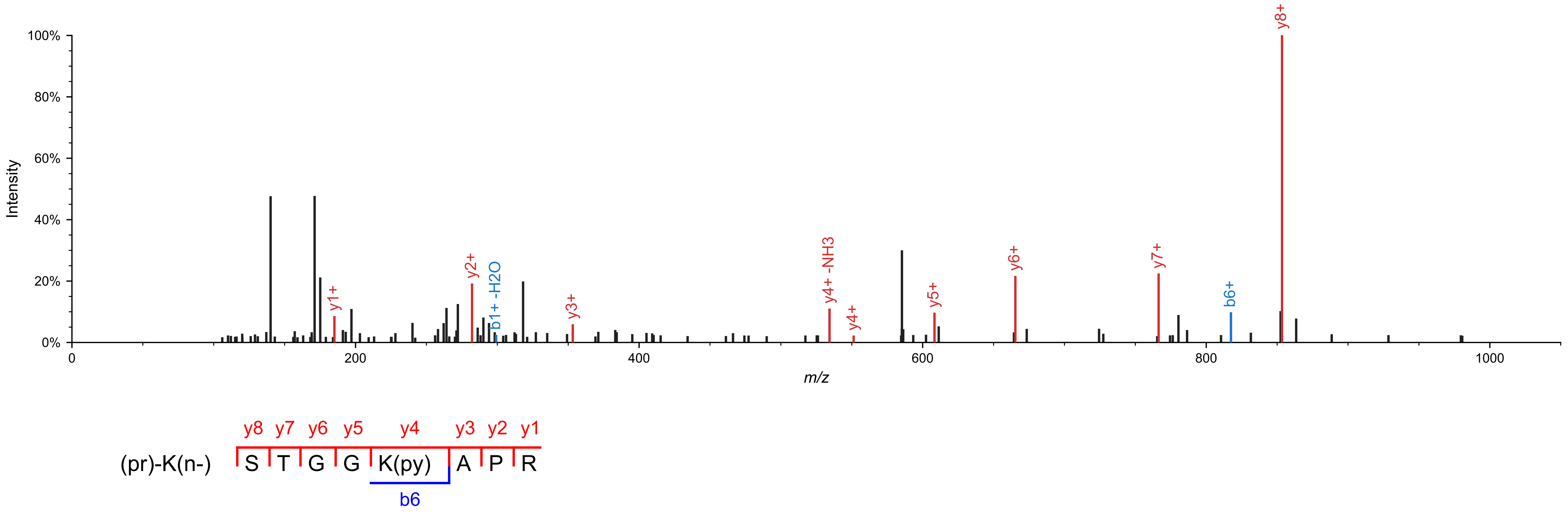

Raw file: QEP220103_RN_A2780_13, Scan: 4904, m/z: 832.9651, Charge: 2, RT (min): 13.37, Score: 30.15 (H31K36Formyl)

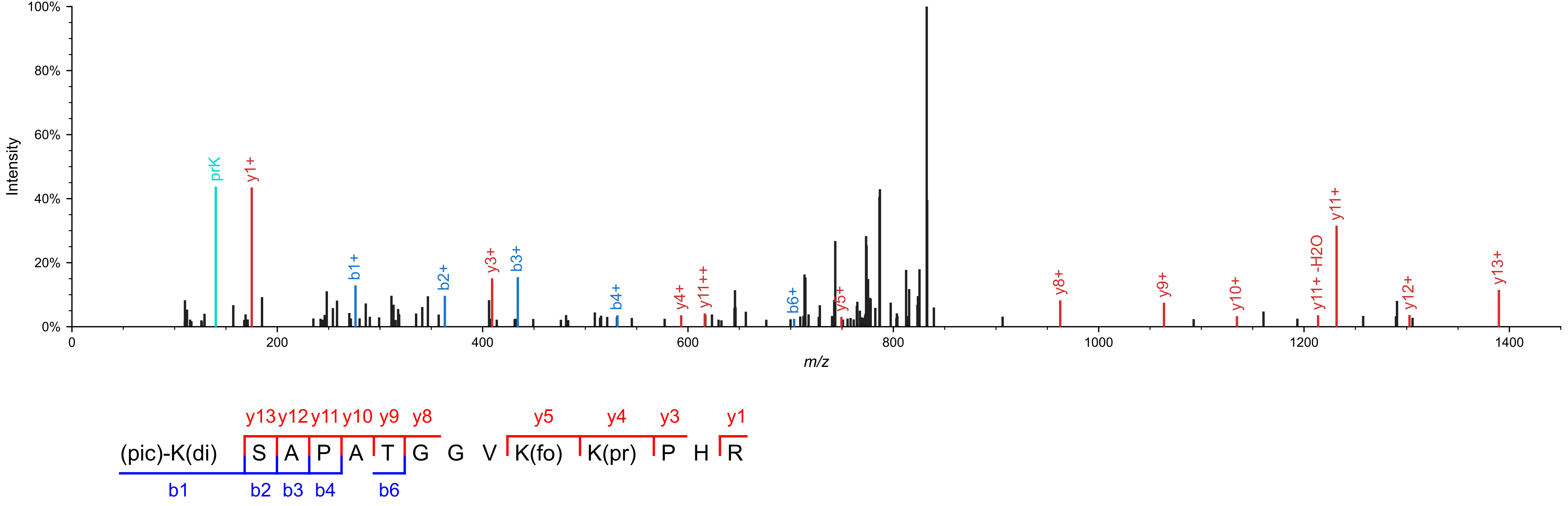

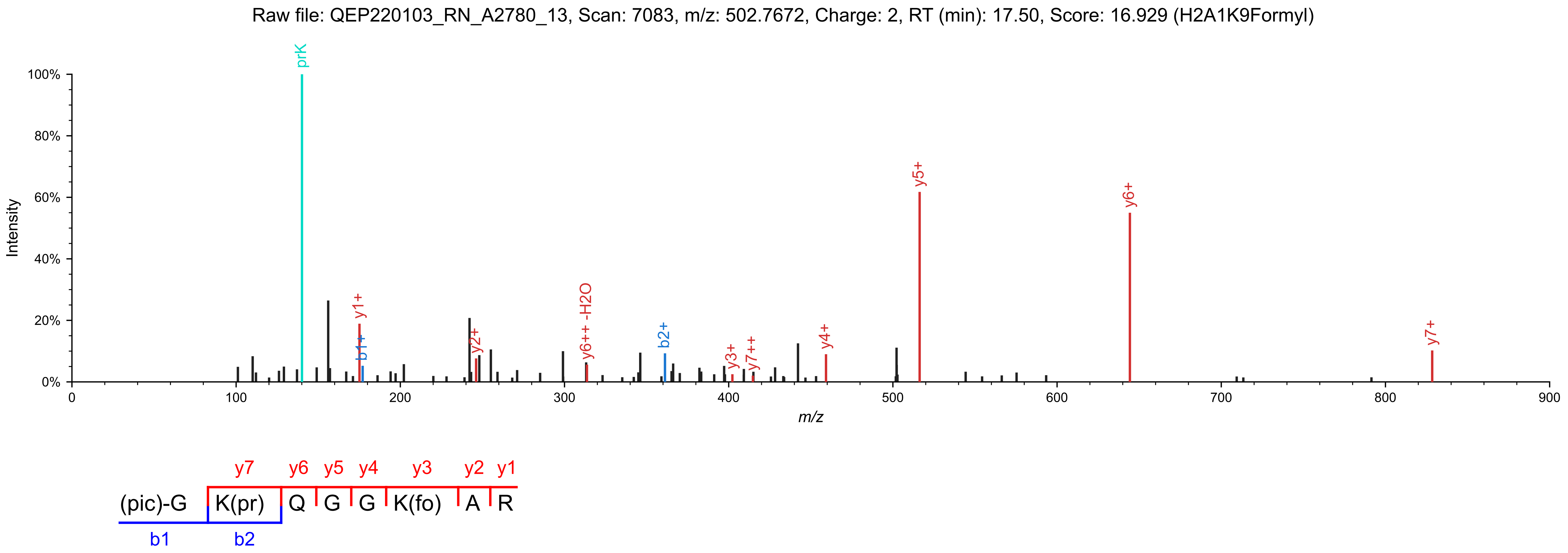

Raw file: QEP220103_RN_A2780_13, Scan: 7083, m/z: 502.7672, Charge: 2, RT (min): 17.50, Score: 16.929 (H2A1K9Formyl)
Intensity
100%
80%
60%
40%
20%
0%
0
100
200
300
400
500
600
700
800
900
m/z
prK
y1+
b1+
y2+
y6++ -H2O
b2+
y3+
y7++
y4+
y5+
y6+
y7+
y7 y6 y5 y4 y3 y2 y1
(pic)-G K(pr) Q G G K(fo) A R
b1 b2

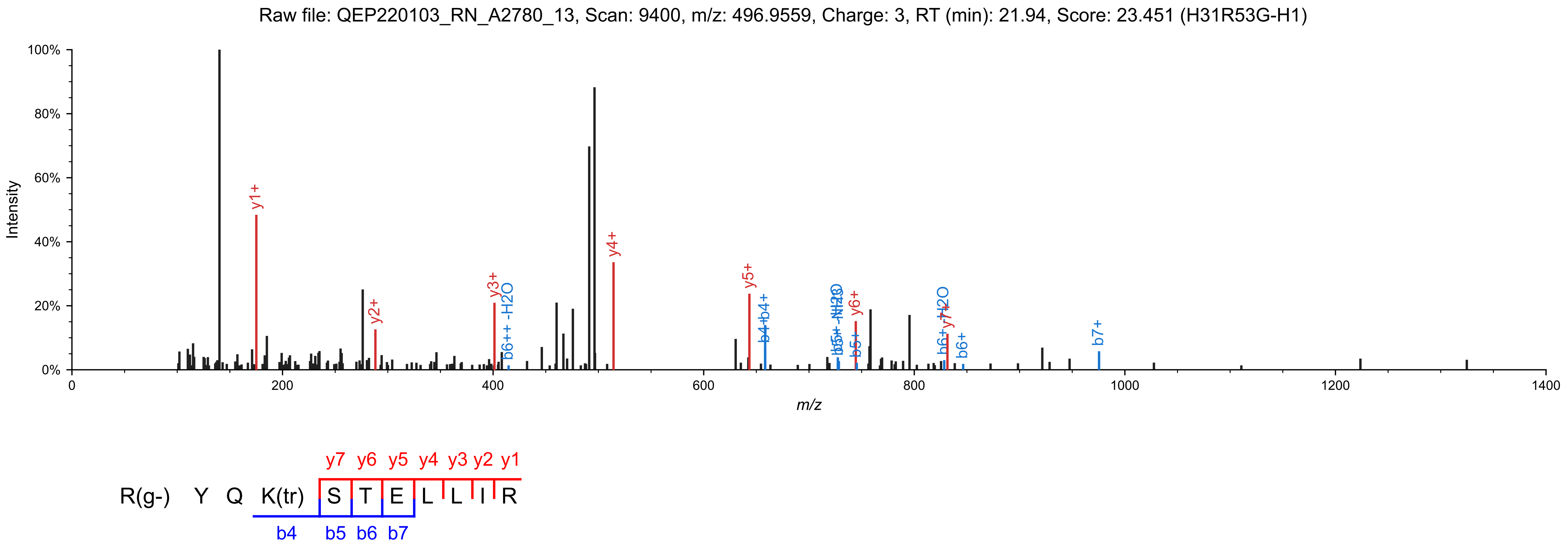

Raw file: QEP220103_RN_A2780_13, Scan: 9400, m/z: 496.9559, Charge: 3, RT (min): 21.94, Score: 23.451 (H31R53G-H1)
Intensity
m/z
y1+
y2+
y3+
y4+
y5+
y6+
y7+
b4+
b5+
b6+
b7+
b6++ -H2O
R(g-) Y Q K(tr) S T E L L I R
y7 y6 y5 y4 y3 y2 y1
b4 b5 b6 b7

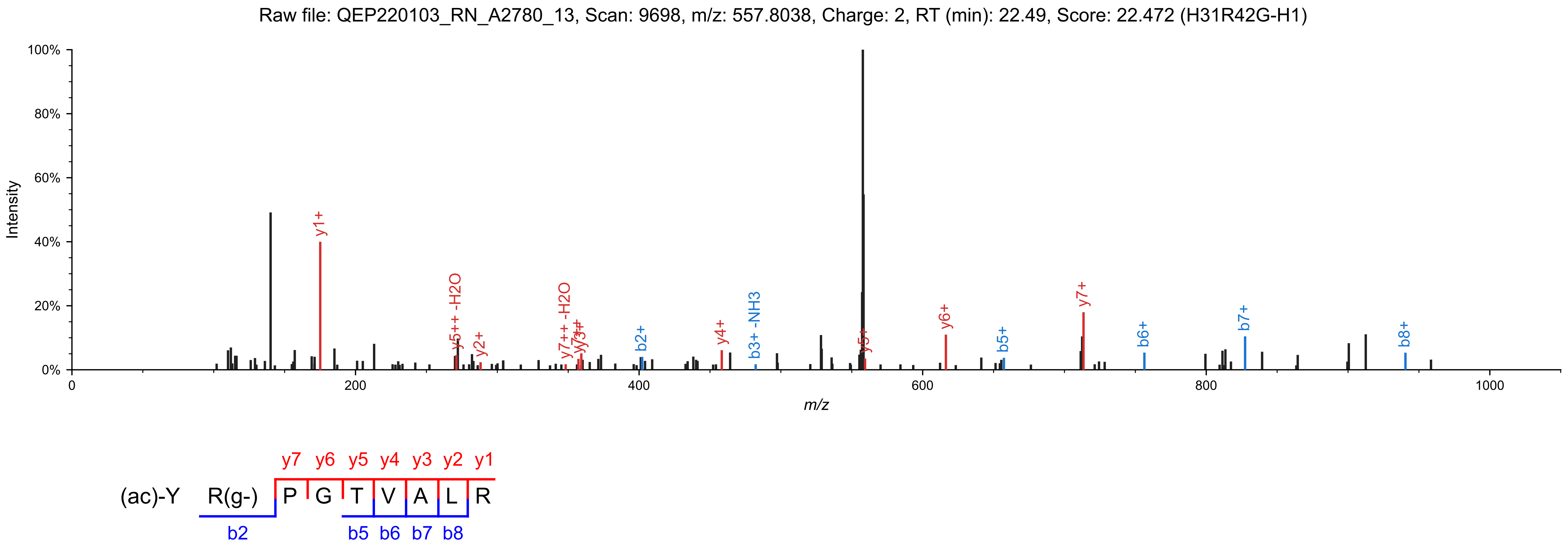

Raw file: QEP220103_RN_A2780_13, Scan: 9698, m/z: 557.8038, Charge: 2, RT (min): 22.49, Score: 22.472 (H31R42G-H1)
Intensity
100%
80%
60%
40%
20%
0%
0
200
400
600
800
1000
m/z
y1+
y5++ -H2O
y2+
y7++ -H2O
y3+
b2+
y4+
b3+ -NH3
y5+
y6+
b5+
y7+
b6+
b7+
b8+
y7 y6 y5 y4 y3 y2 y1
(ac)-Y R(g-) P G T V A L R
b2 b5 b6 b7 b8

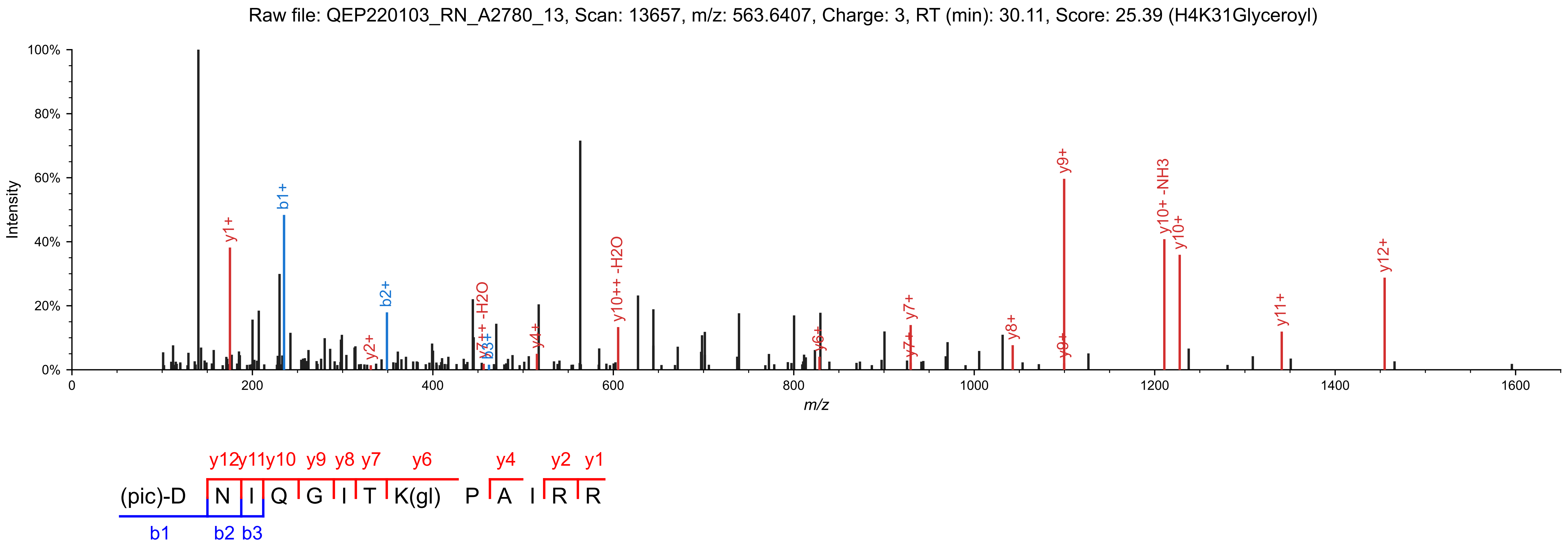

Raw file: QEP220103_RN_A2780_13, Scan: 13657, m/z: 563.6407, Charge: 3, RT (min): 30.11, Score: 25.39 (H4K31Glyceroyl)
Intensity
m/z
y1+
b1+
y2+
b2+
y7++ -H2O
b3+
y4+
y10++ -H2O
y6+
y7+
y8+
y9+
y10+ -NH3
y10+
y11+
y12+
y12 y11 y10 y9 y8 y7 y6 y4 y2 y1
(pic)-D N I Q G I T K(gl) P A I R R
b1 b2 b3

Raw file: QEP220103_RN_A2780_13, Scan: 14050, m/z: 698.8591, Charge: 2, RT (min): 30.89, Score: 23.155 (H31Y54Phosphoryl)

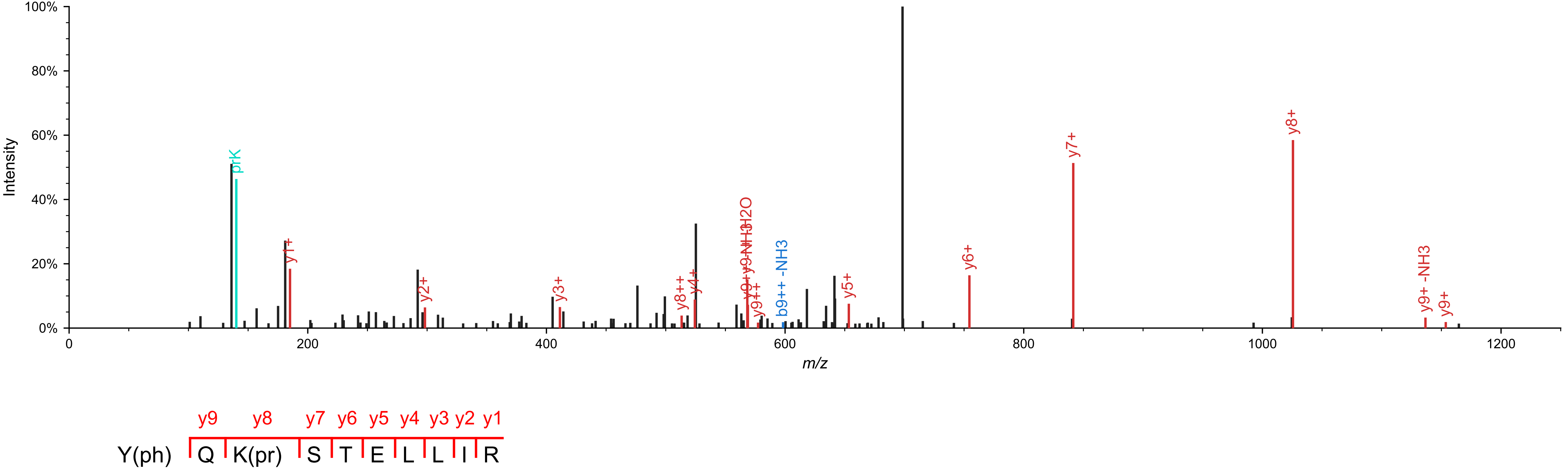

Raw file: QEP220103_RN_A2780_14, Scan: 1416, m/z: 453.2274, Charge: 2, RT (min): 6.72, Score: 16.243 (H2A1K36Crotonyl)

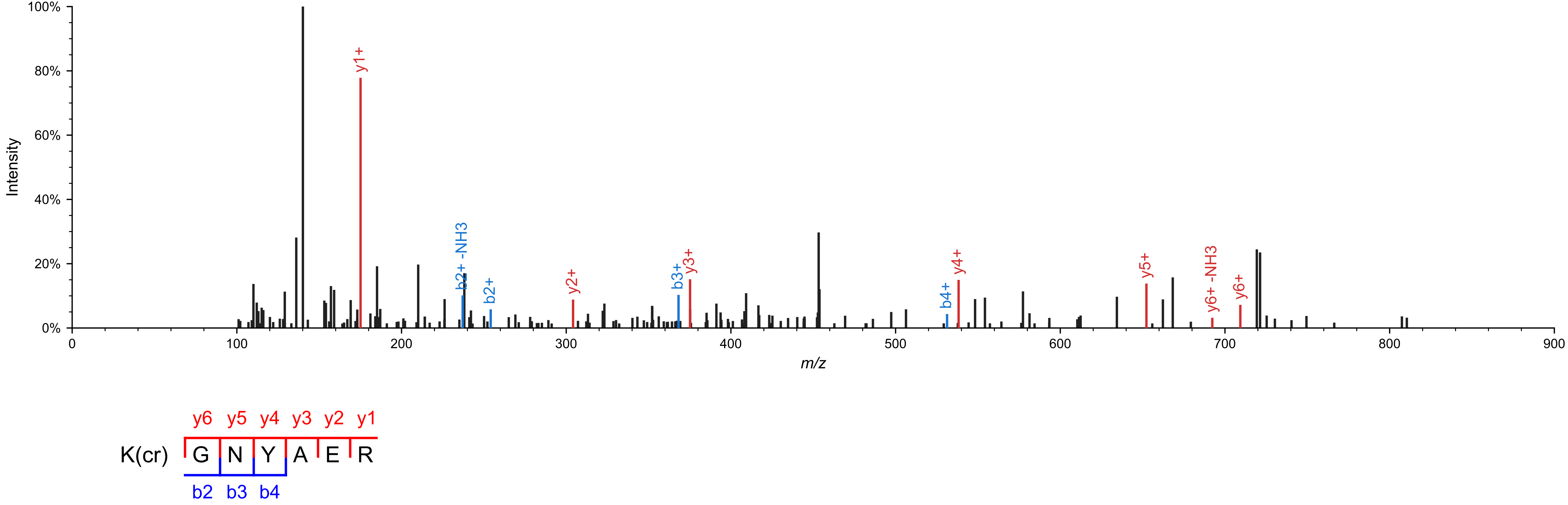

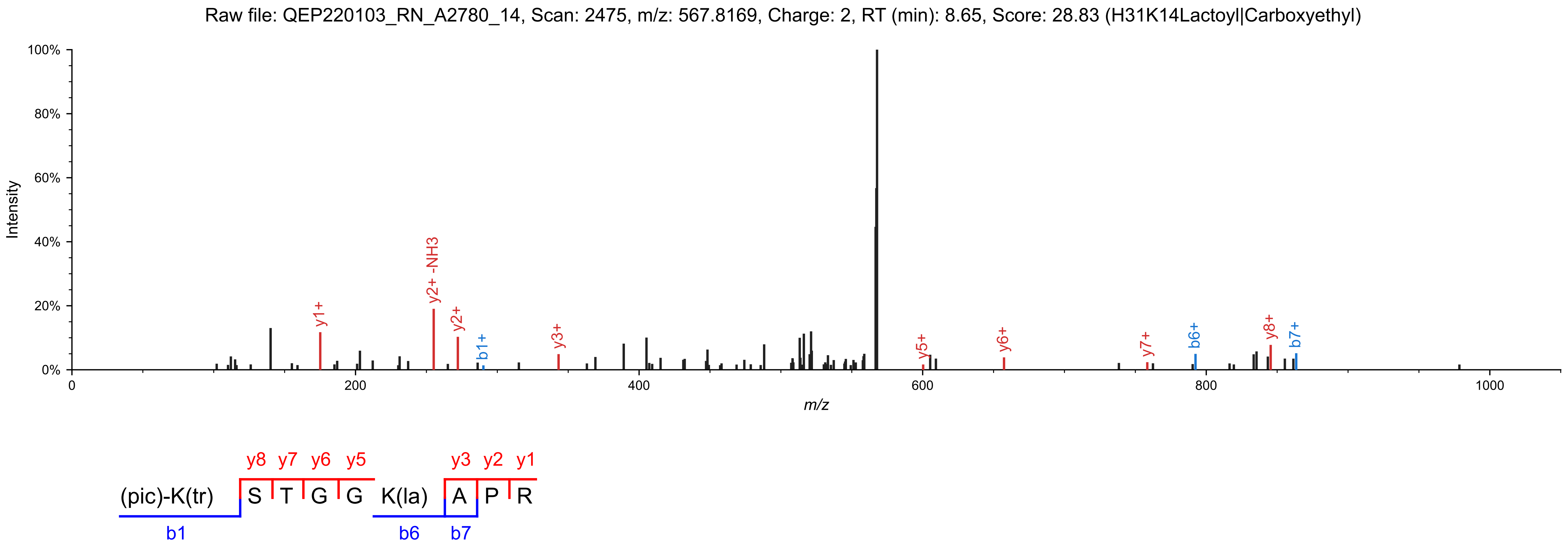

Raw file: QEP220103_RN_A2780_14, Scan: 2475, m/z: 567.8169, Charge: 2, RT (min): 8.65, Score: 28.83 (H31K14Lactoyl|Carboxyethyl)
Intensity
100%
80%
60%
40%
20%
0%
0
200
400
600
800
1000
m/z
y1+
y2+ -NH3
y2+
b1+
y3+
y5+
y6+
y7+
b6+
y8+
b7+
y8 y7 y6 y5
y3 y2 y1
(pic)-K(tr) S T G G K(la) A P R
b1
b6 b7

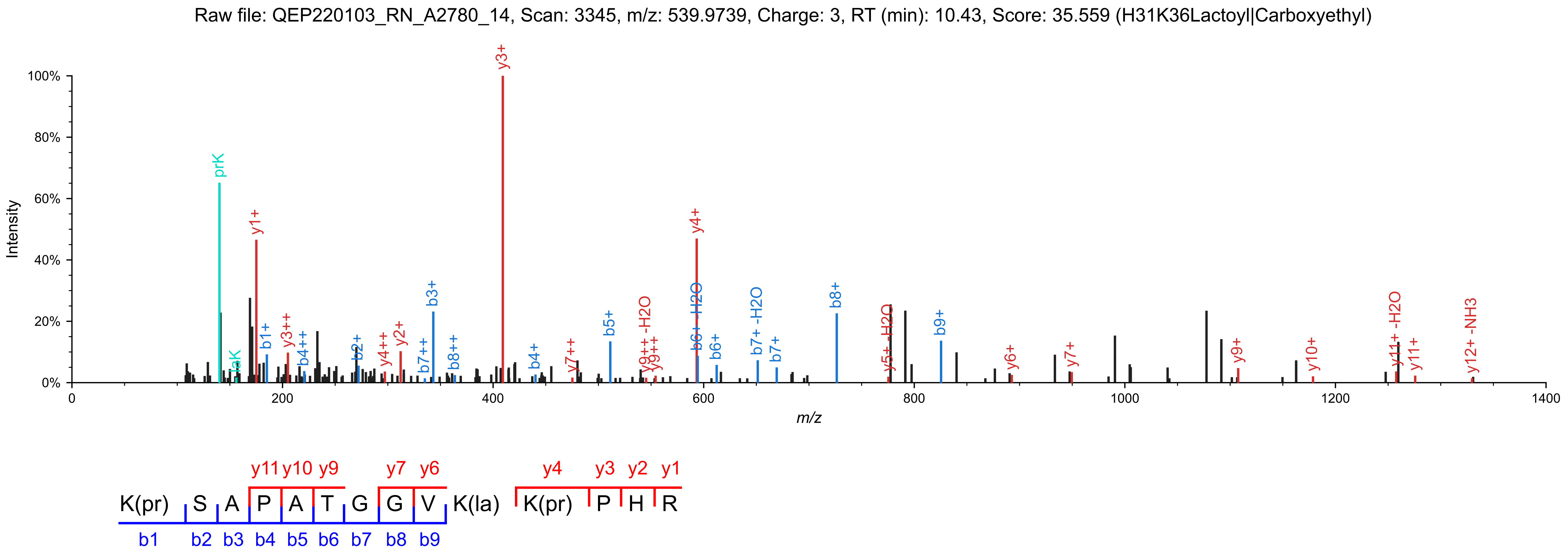
Raw file: QEP220103_RN_A2780_14, Scan: 3345, m/z: 539.9739, Charge: 3, RT (min): 10.43, Score: 35.559 (H31K36Lactoyl|Carboxyethyl)
Intensity
m/z
K(pr) S A P A T G G V K(la) K(pr) P H R
y11 y10 y9 y7 y6 y4 y3 y2 y1
b1 b2 b3 b4 b5 b6 b7 b8 b9

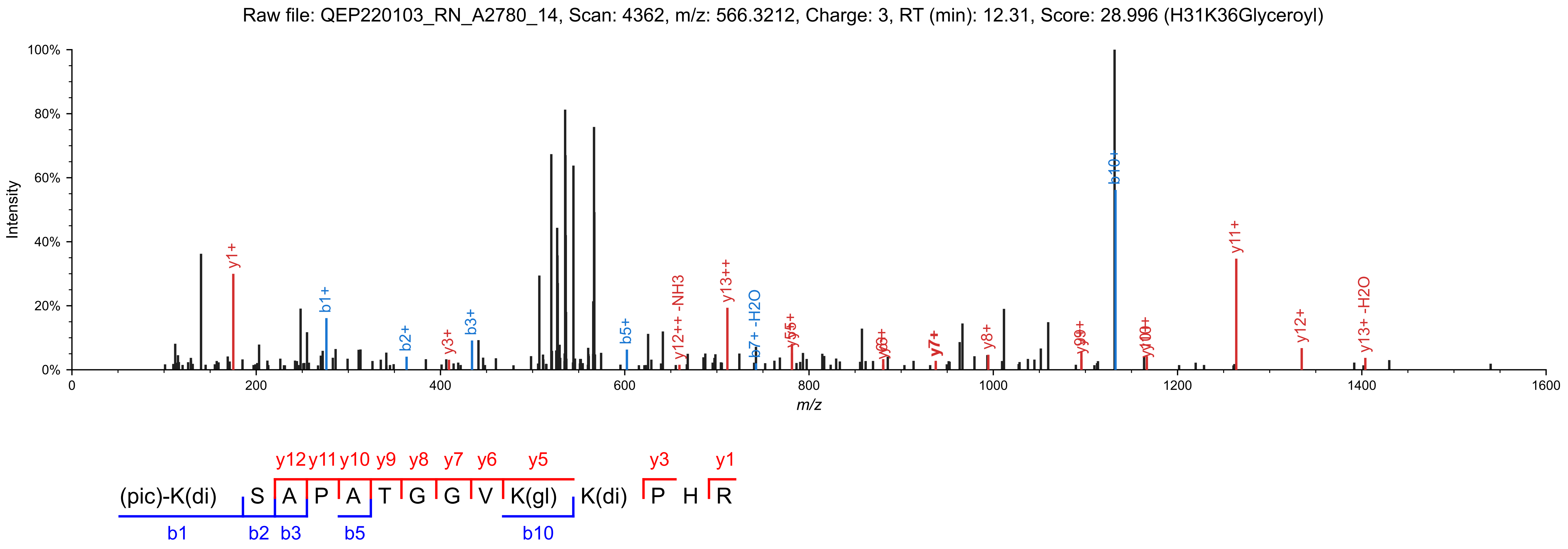
Raw file: QEP220103_RN_A2780_14, Scan: 4362, m/z: 566.3212, Charge: 3, RT (min): 12.31, Score: 28.996 (H31K36Glyceroyl)
Intensity
100%
80%
60%
40%
20%
0%
0
200
400
600
800
1000
1200
1400
1600
m/z
y1+
b1+
b2+
y3+
b3+
b5+
y12++ -NH3
y13++
b7+ -H2O
y5+
y7+
y8+
y9+
b10+
y10+
y11+
y12+
y13+ -H2O
y12 y11 y10 y9 y8 y7 y6 y5 y3 y1
(pic)-K(di) S A P A T G G V K(gl) K(di) P H R
b1 b2 b3 b5 b10

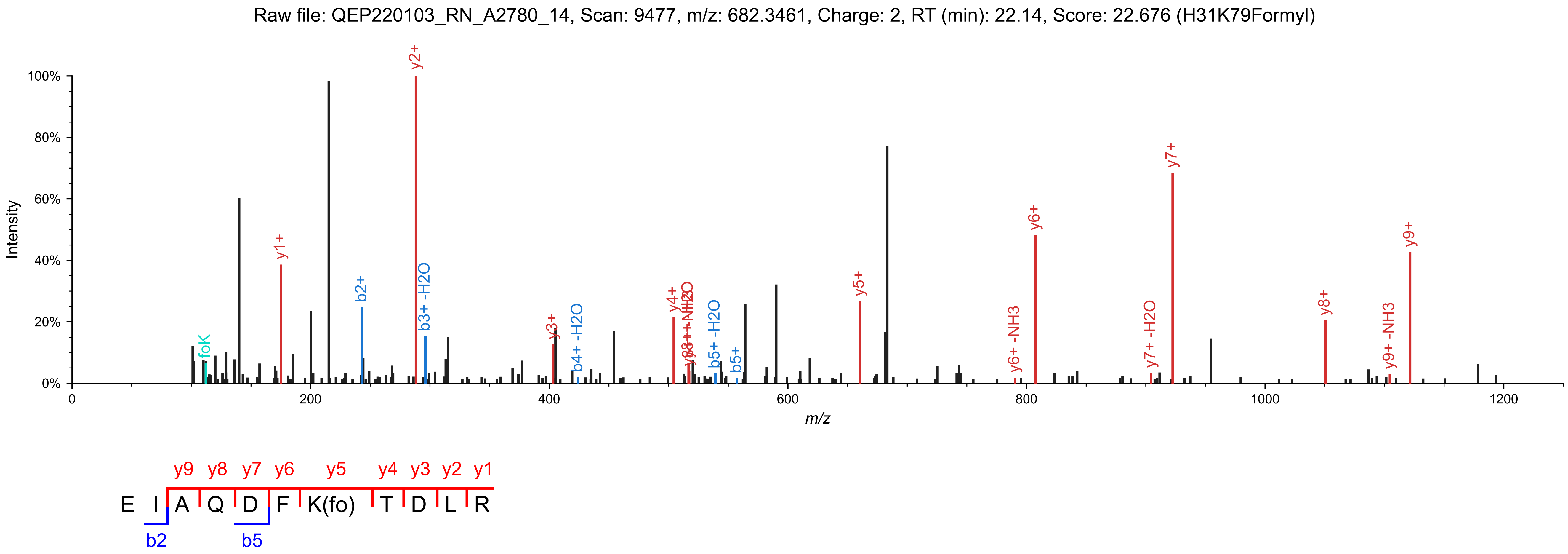

Raw file: QEP220103_RN_A2780_14, Scan: 9477, m/z: 682.3461, Charge: 2, RT (min): 22.14, Score: 22.676 (H31K79Formyl)
Intensity
m/z
100%
80%
60%
40%
20%
0%
0
200
400
600
800
1000
1200
foK
y1+
b2+
y2+
b3+ -H2O
y3+
b4+ -H2O
y4+
b5+ -H2O
b5+
y5+
y6+ -NH3
y6+
y7+ -H2O
y7+
y8+
y9+ -NH3
y9+
y9 y8 y7 y6 y5 y4 y3 y2 y1
E I A Q D F K(fo) T D L R
b2 b5

Raw file: QEP220103_RN_A2780_14, Scan: 11023, m/z: 526.2578, Charge: 2, RT (min): 25.13, Score: 14.287 (H2A1BK36N-Pentosyl Lysine)

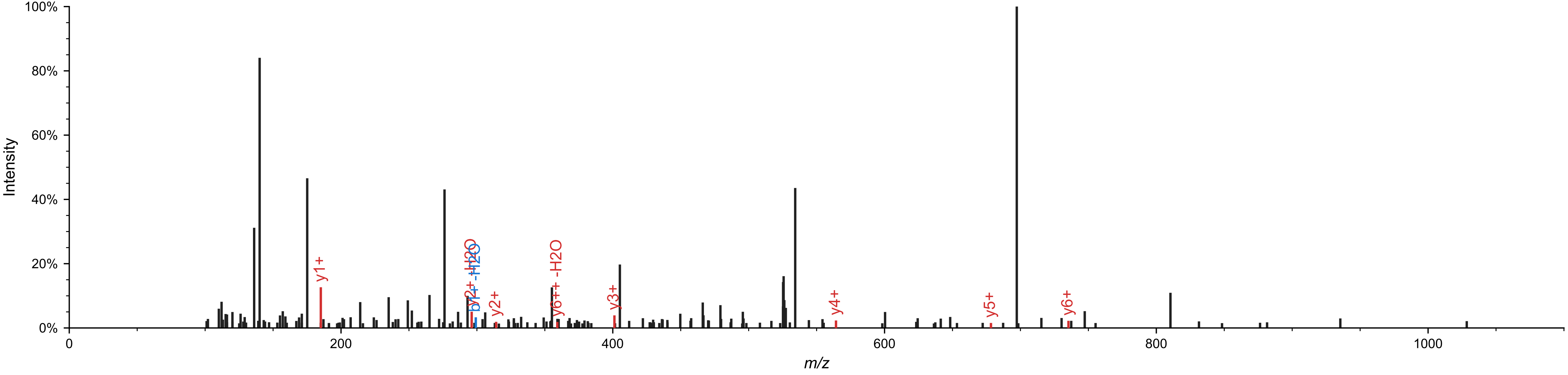

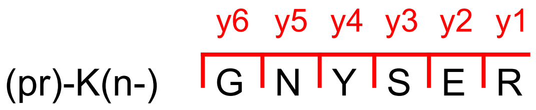

Raw file: QEP220103_RN_A2780_14, Scan: 14780, m/z: 428.7556, Charge: 2, RT (min): 32.48, Score: 12.978 (H31K64Crotonyl)

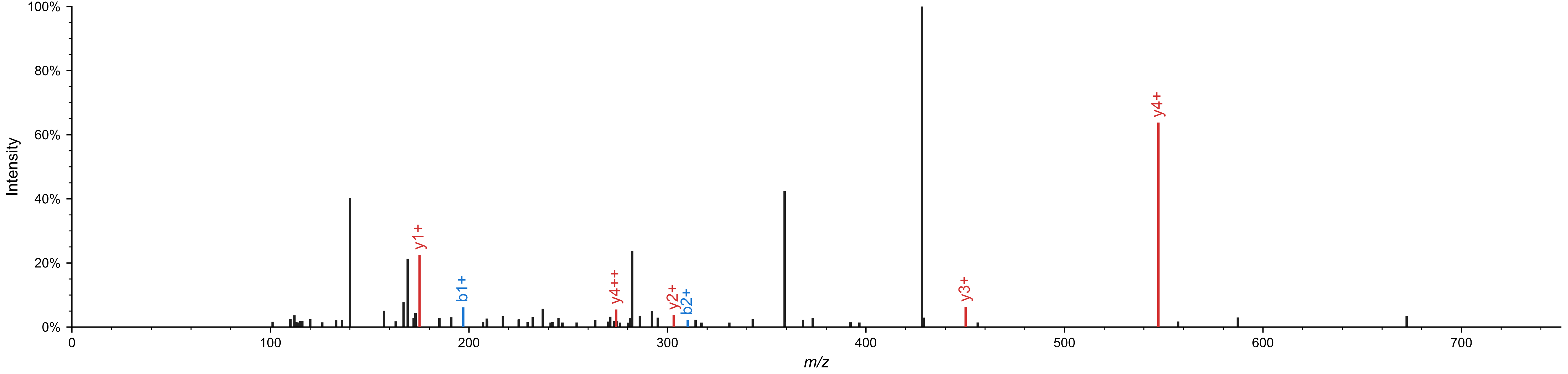

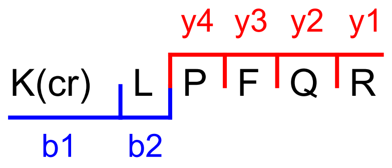

Raw file: QEP220103_RN_A2780_14, Scan: 17473, m/z: 578.9868, Charge: 3, RT (min): 37.61, Score: 33.965 (H4K79Crotonyl)

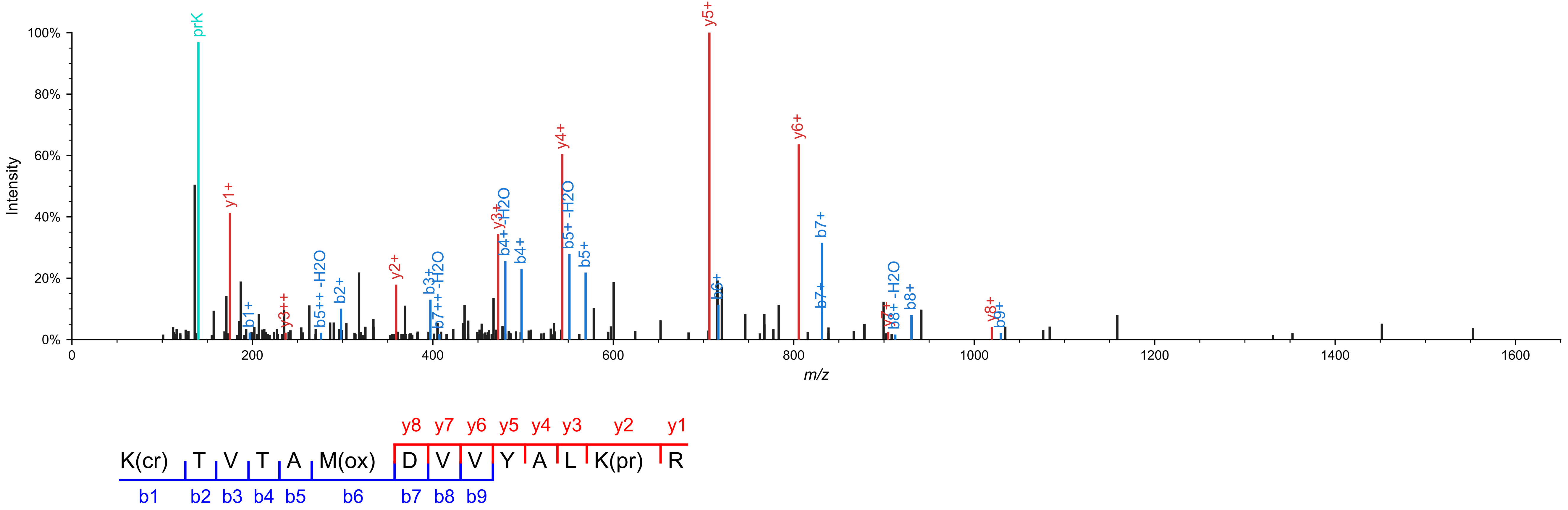

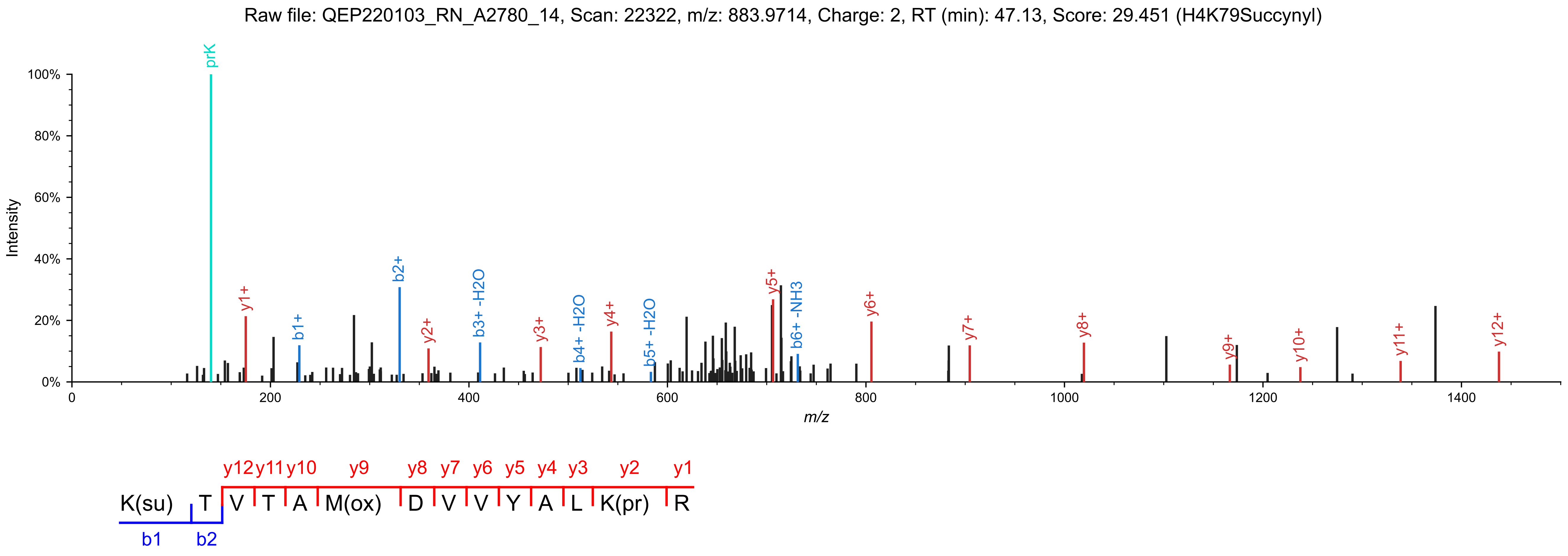

Raw file: QEP220103_RN_A2780_14, Scan: 22322, m/z: 883.9714, Charge: 2, RT (min): 47.13, Score: 29.451 (H4K79Succynyl)
Intensity
100%
80%
60%
40%
20%
0%
0
200
400
600
800
1000
1200
1400
m/z
prK
y1+
b1+
b2+
y2+
b3+ -H2O
y3+
b4+ -H2O
y4+
b5+ -H2O
y5+
b6+ -NH3
y6+
y7+
y8+
y9+
y10+
y11+
y12+
y12 y11 y10 y9 y8 y7 y6 y5 y4 y3 y2 y1
K(su) T V T A M(ox) D V V Y A L K(pr) R
b1 b2

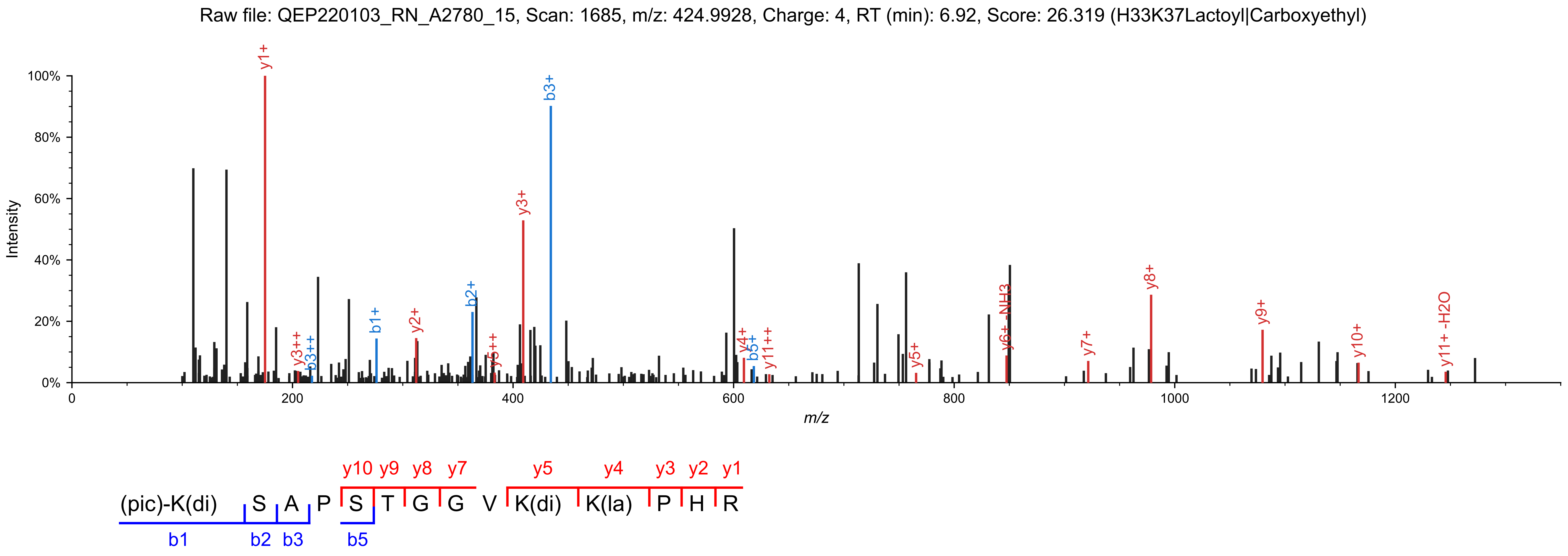

Raw file: QEP220103_RN_A2780_15, Scan: 1685, m/z: 424.9928, Charge: 4, RT (min): 6.92, Score: 26.319 (H33K37Lactoyl|Carboxyethyl)
Intensity
m/z
y10 y9 y8 y7 y5 y4 y3 y2 y1
(pic)-K(di) S A P S T G G V K(di) K(la) P H R
b1 b2 b3 b5

Raw file: QEP220103_RN_A2780_15, Scan: 3669, m/z: 506.2827, Charge: 2, RT (min): 10.81, Score: 21.793 (H31K9Lactoyl|Carboxyethyl)

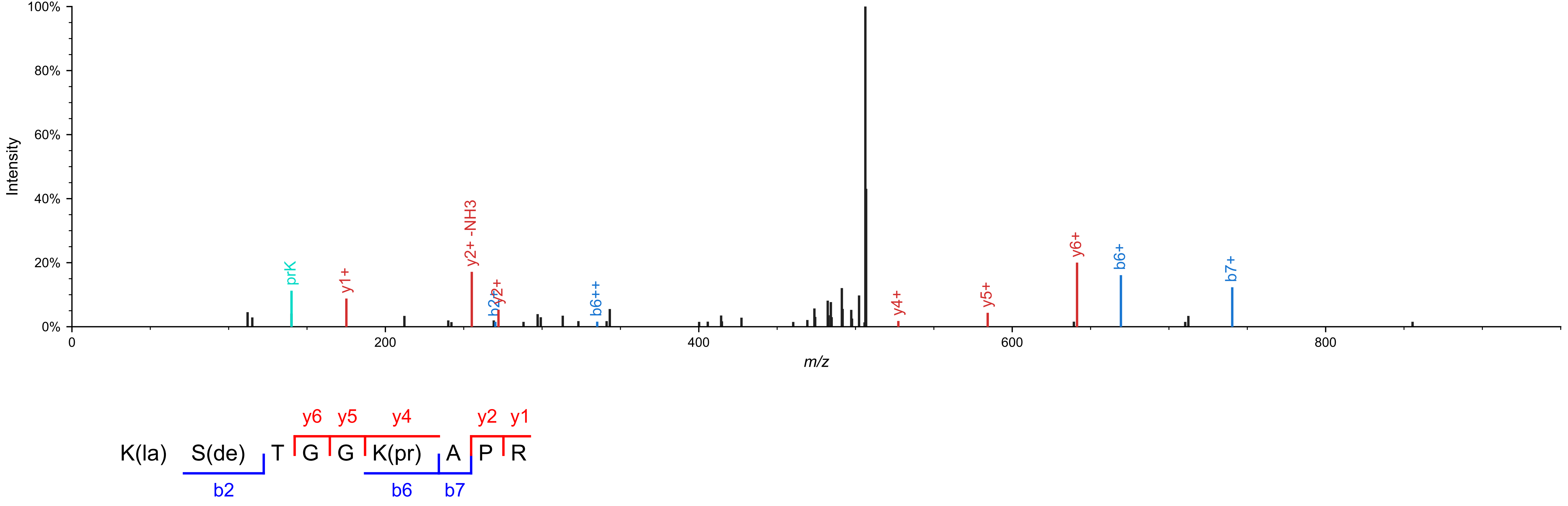

Raw file: QEP220103_RN_A2780_15, Scan: 7717, m/z: 524.7803, Charge: 2, RT (min): 18.67, Score: 18.273 (H2A1K9Lactoyl|Carboxyethyl)

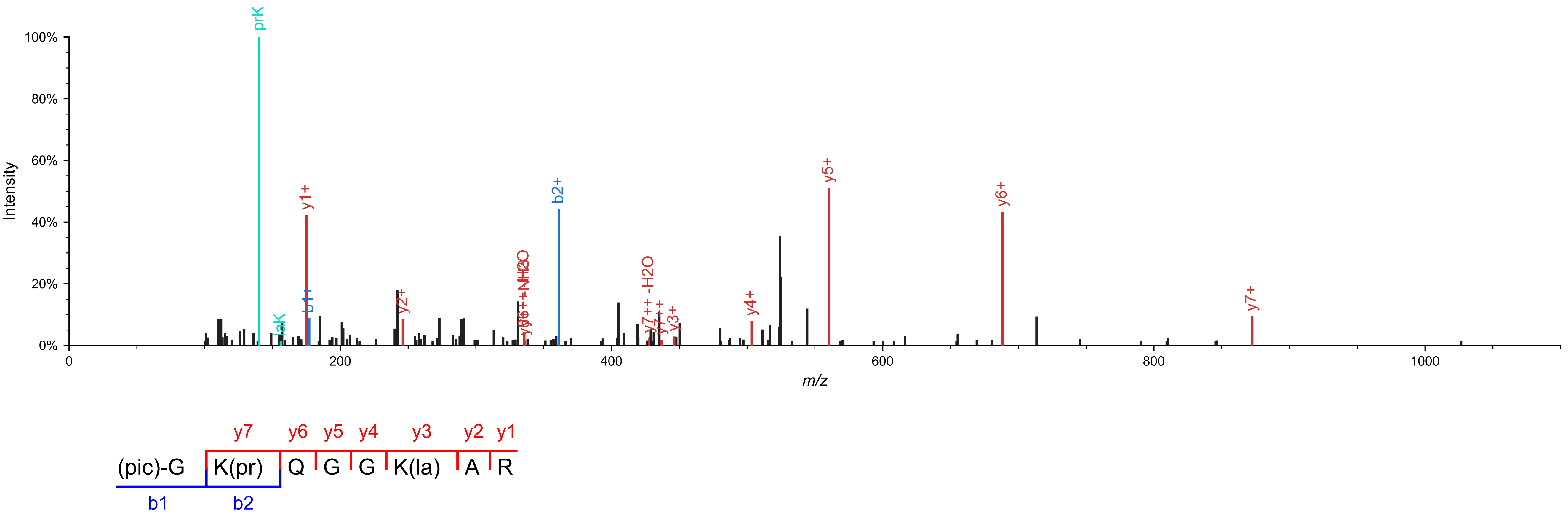

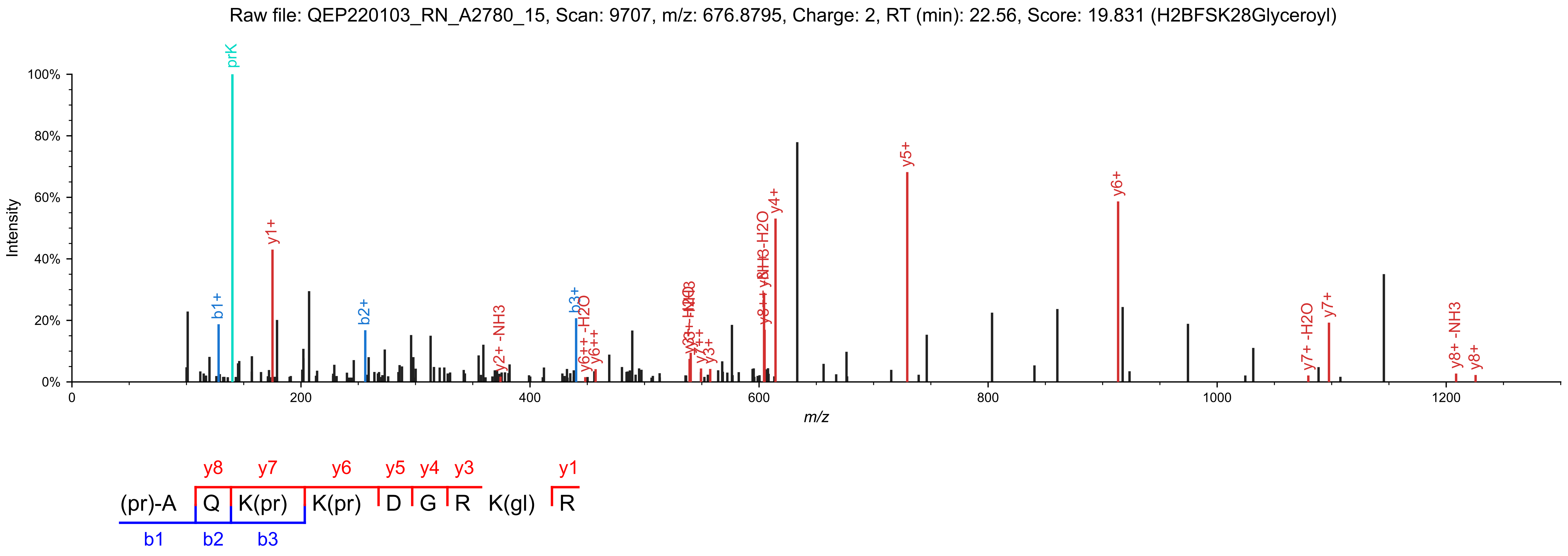

Raw file: QEP220103_RN_A2780_15, Scan: 9707, m/z: 676.8795, Charge: 2, RT (min): 22.56, Score: 19.831 (H2BFSK28Glyceroyl)
Intensity
m/z
prK
y1+
b1+
b2+
b3+
y5+
y6+
y4+
y7+
y8+
y8+ -NH3
y7+ -H2O
y2+ -NH3
y3+
(pr)-A Q K(pr) K(pr) D G R K(gl) R
y8 y7 y6 y5 y4 y3 y1
b1 b2 b3

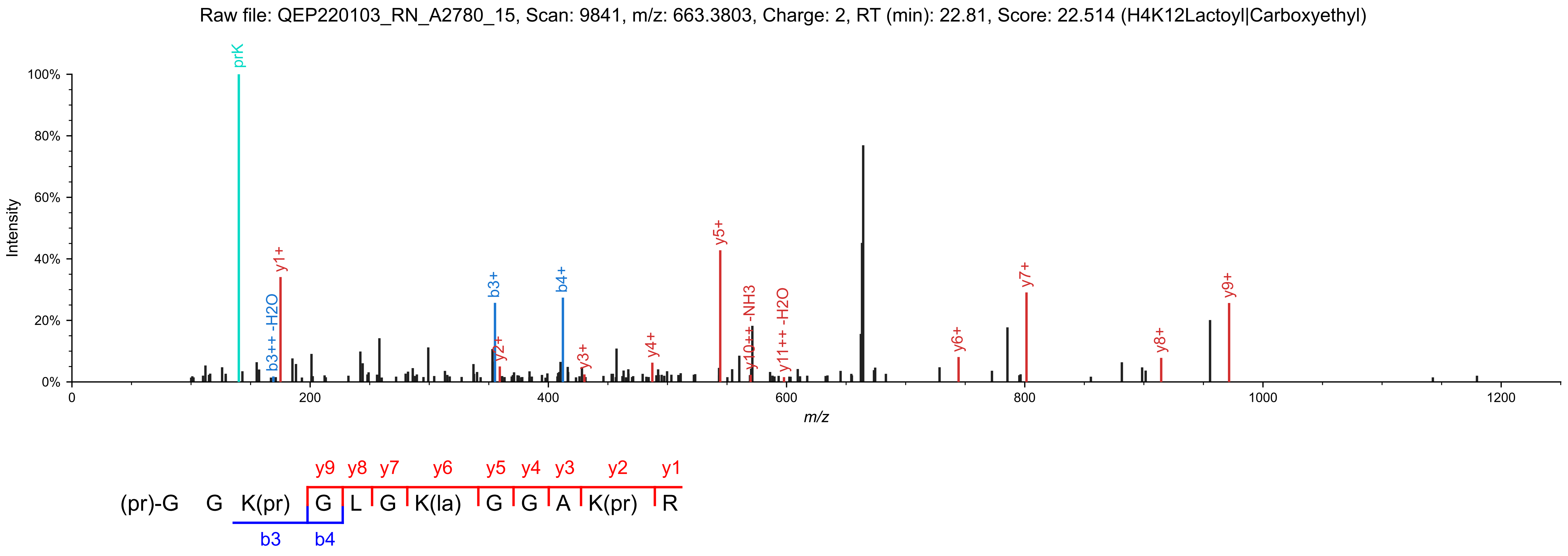
Raw file: QEP220103_RN_A2780_15, Scan: 9841, m/z: 663.3803, Charge: 2, RT (min): 22.81, Score: 22.514 (H4K12Lactoyl|Carboxyethyl)
Intensity
100%
80%
60%
40%
20%
0%
0
200
400
600
800
1000
1200
m/z
prK
b3++ -H2O
y1+
b3+
y2+
b4+
y3+
y4+
y5+
y10++ -NH3
y11++ -H2O
y6+
y7+
y8+
y9+
y9 y8 y7 y6 y5 y4 y3 y2 y1
(pr)-G G K(pr) G L G K(la) G G A K(pr) R
b3 b4

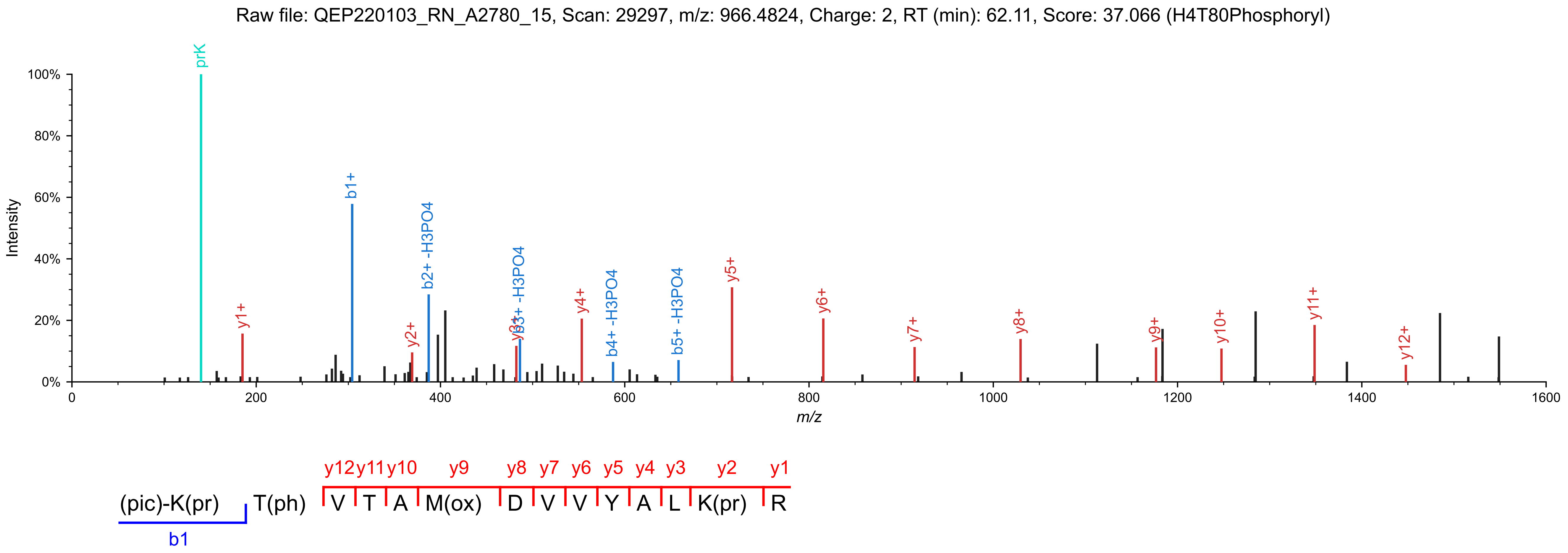
Raw file: QEP220103_RN_A2780_15, Scan: 29297, m/z: 966.4824, Charge: 2, RT (min): 62.11, Score: 37.066 (H4T80Phosphoryl)
prK
b1+
b2+ -H3PO4
b3+ -H3PO4
b4+ -H3PO4
b5+ -H3PO4
y1+
y2+
y3+
y4+
y5+
y6+
y7+
y8+
y9+
y10+
y11+
y12+
Intensity
100%
80%
60%
40%
20%
0%
0
200
400
600
800
1000
1200
1400
1600
m/z
y12 y11 y10 y9 y8 y7 y6 y5 y4 y3 y2 y1
(pic)-K(pr) T(ph) V T A M(ox) D V V Y A L K(pr) R
b1

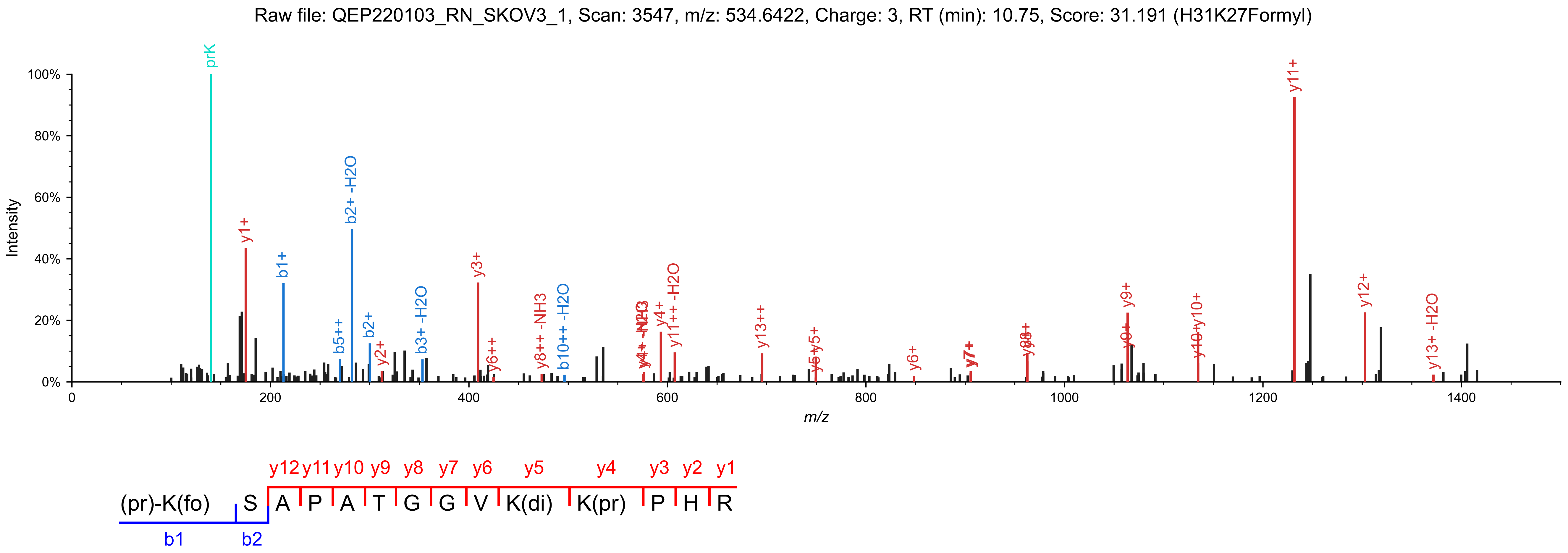
Raw file: QEP220103_RN_SKOV3_1, Scan: 3547, m/z: 534.6422, Charge: 3, RT (min): 10.75, Score: 31.191 (H31K27Formyl)
Intensity
m/z
prK
y1+
b1+
b2+ -H2O
b2+
b5++
y2+
b3+ -H2O
y3+
y6++
y8++ -NH3
b10++ -H2O
y4+
y11++ -H2O
y13++
y5+
y6+
y7+
y8+
y9+
y10+
y11+
y12+
y13+ -H2O
y12 y11 y10 y9 y8 y7 y6 y5 y4 y3 y2 y1
(pr)-K(fo) S A P A T G G V K(di) K(pr) P H R
b1 b2

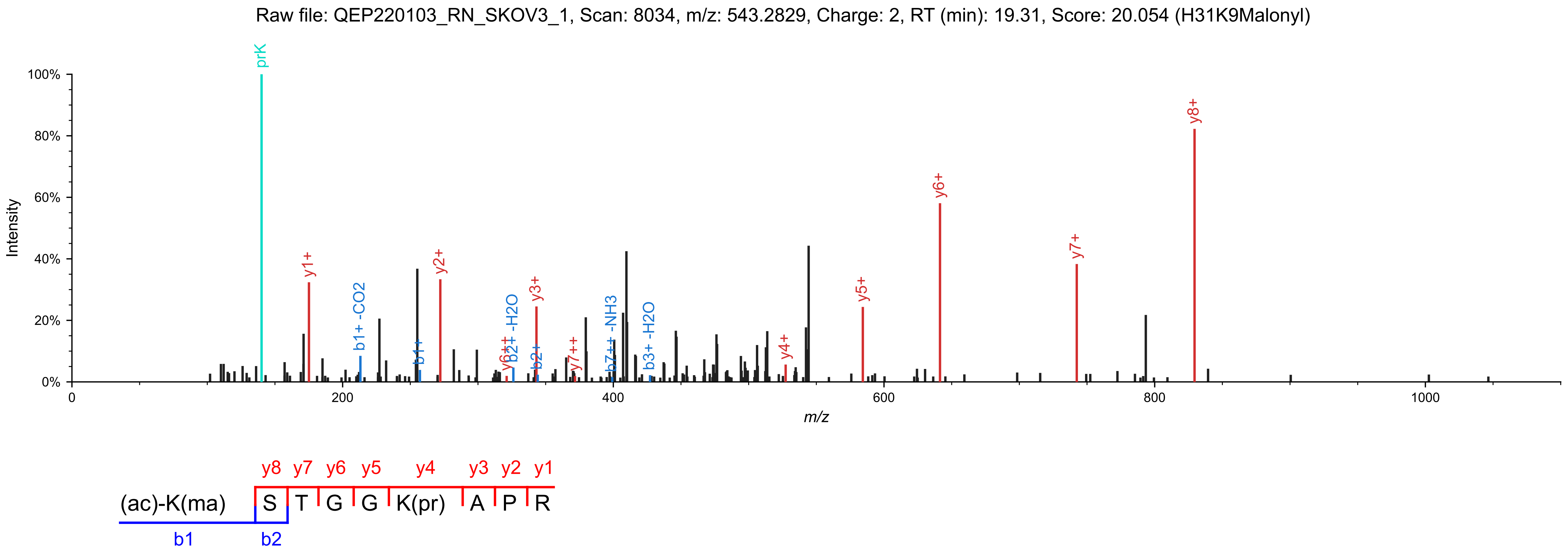
Raw file: QEP220103_RN_SKOV3_1, Scan: 8034, m/z: 543.2829, Charge: 2, RT (min): 19.31, Score: 20.054 (H31K9Malonyl)
Intensity
100%
80%
60%
40%
20%
0%
0
200
400
600
800
1000
m/z
prK
y1+
b1+ -CO2
b1+
y2+
y6++
b2+ -H2O
b2+
y3+
y7++
b7++ -NH3
b3+ -H2O
y4+
y5+
y6+
y7+
y8+
y8 y7 y6 y5 y4 y3 y2 y1
(ac)-K(ma) S T G G K(pr) A P R
b1 b2

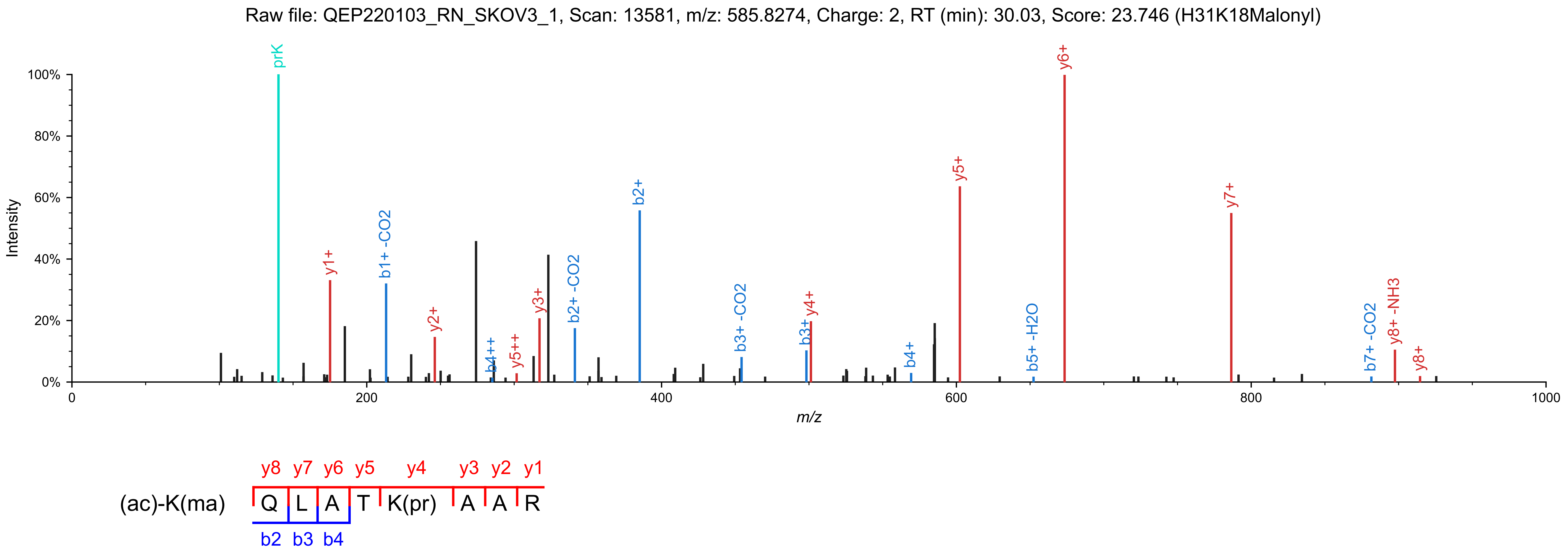

Raw file: QEP220103_RN_SKOV3_1, Scan: 13581, m/z: 585.8274, Charge: 2, RT (min): 30.03, Score: 23.746 (H31K18Malonyl)
Intensity
100%
80%
60%
40%
20%
0%
0
200
400
600
800
1000
m/z
prK
y1+
b1+ -CO2
y2+
b4++
y5++
y3+
b2+ -CO2
b2+
b3+ -CO2
b3+
y4+
b4+
y5+
b5+ -H2O
y6+
y7+
b7+ -CO2
y8+ -NH3
y8+
y8 y7 y6 y5 y4 y3 y2 y1
(ac)-K(ma) Q L A T K(pr) A A R
b2 b3 b4

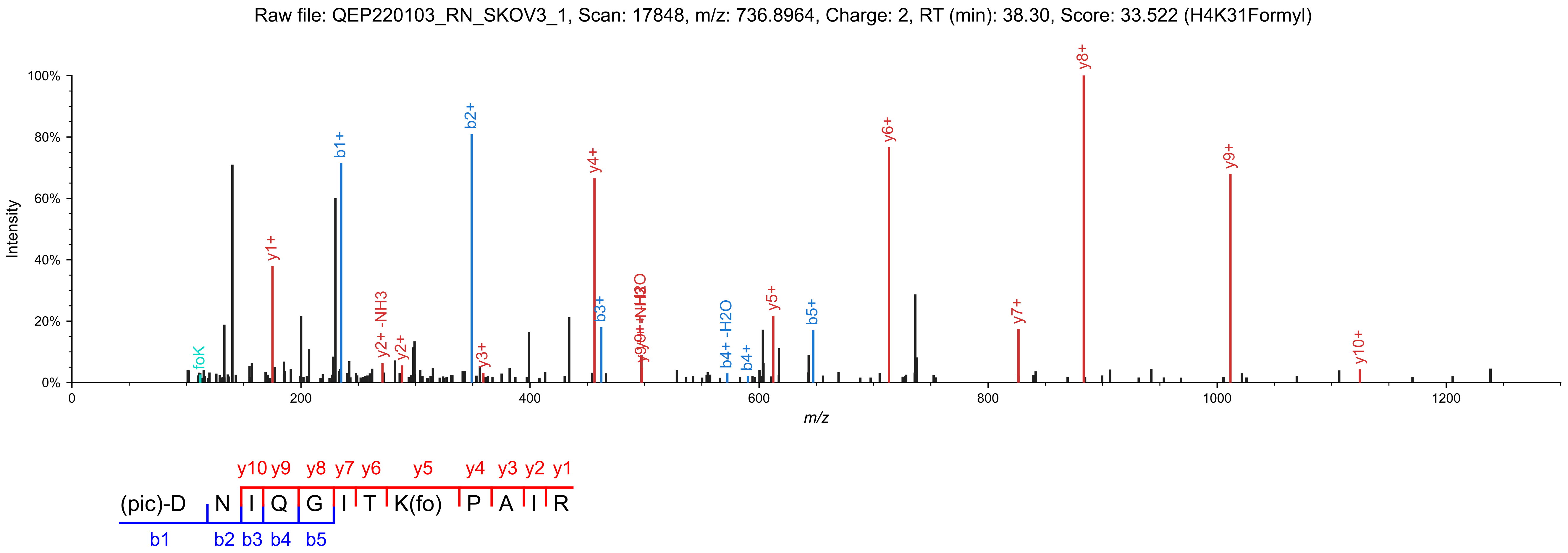
Raw file: QEP220103_RN_SKOV3_1, Scan: 17848, m/z: 736.8964, Charge: 2, RT (min): 38.30, Score: 33.522 (H4K31Formyl)
Intensity
m/z
(pic)-D N I Q G I T K(fo) P A I R
y10 y9 y8 y7 y6 y5 y4 y3 y2 y1
b1 b2 b3 b4 b5

Raw file: QEP220103_RN_SKOV3_1, Scan: 29231, m/z: 833.4191, Charge: 2, RT (min): 61.67, Score: 29.931 (H31T118Phosphoryl)

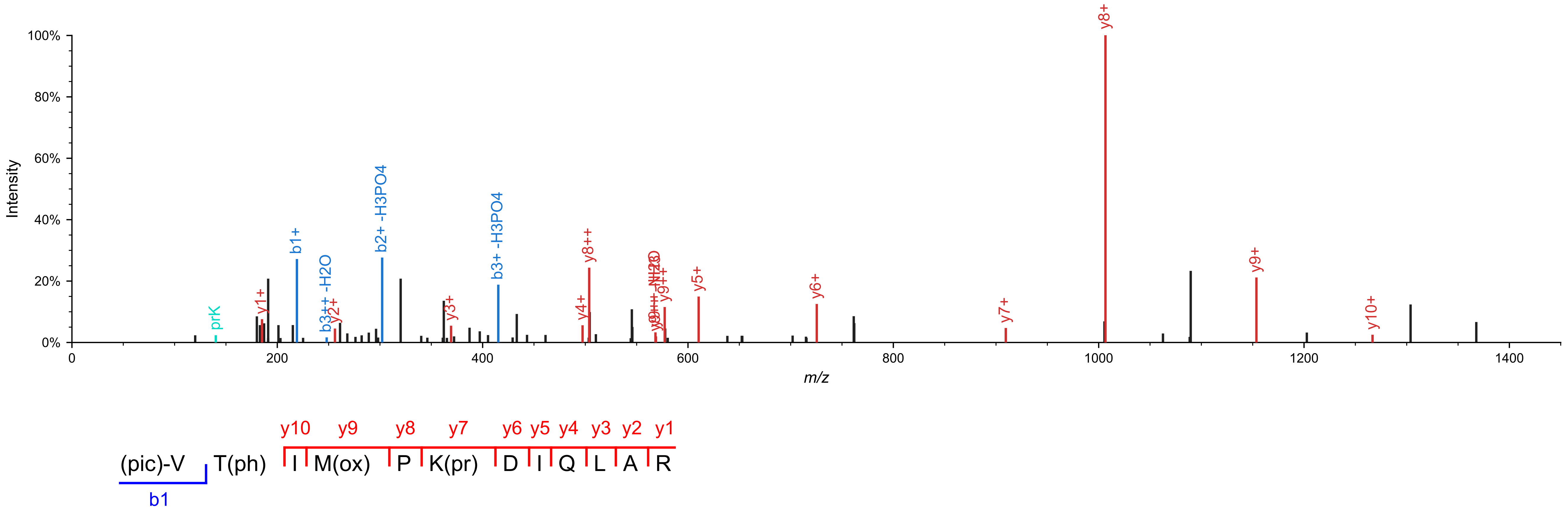

Raw file: QEP220103_RN_SKOV3_2, Scan: 2275, m/z: 550.8247, Charge: 2, RT (min): 8.26, Score: 22.04 (H31K18Lactoyl|Carboxyethyl)

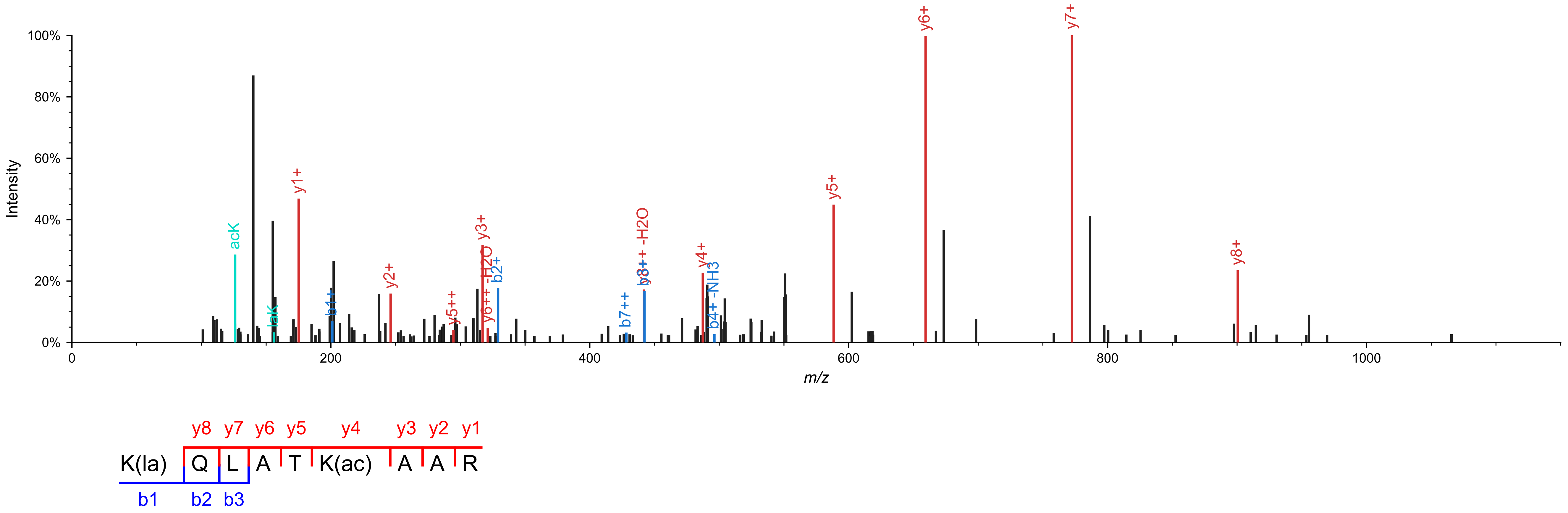

Raw file: QEP220103_RN_SKOV3_2, Scan: 4980, m/z: 487.9523, Charge: 3, RT (min): 13.40, Score: 24.428 (H3Y1K53Formyl)

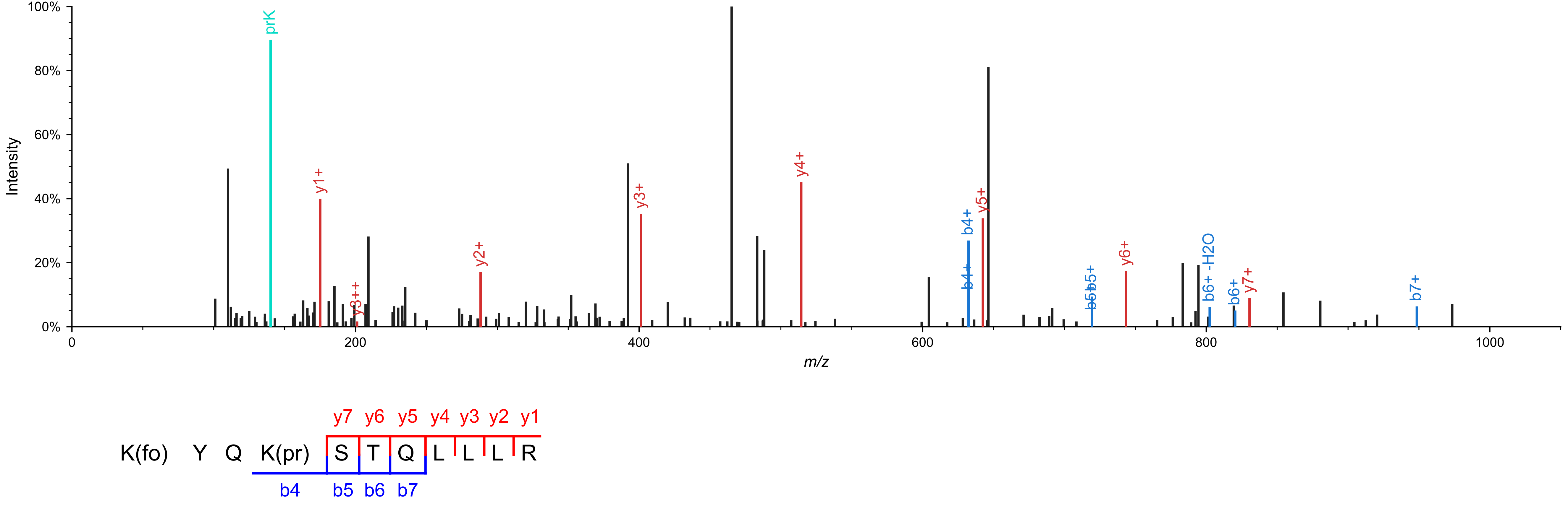

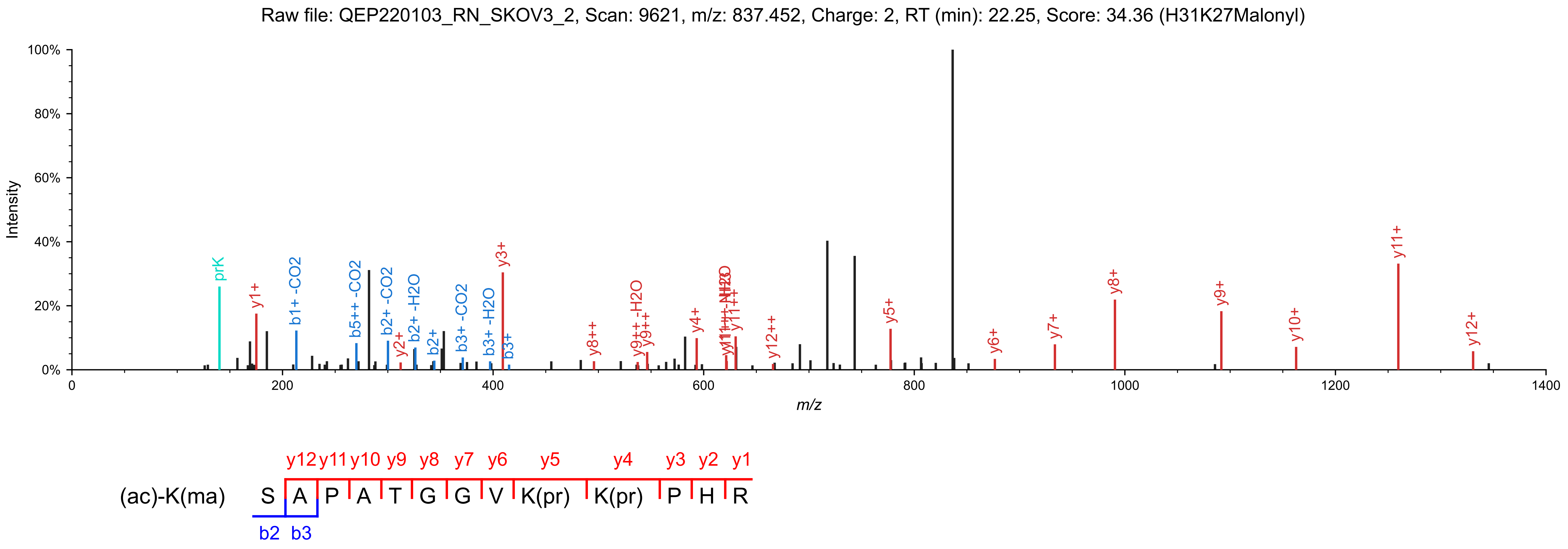

Raw file: QEP220103_RN_SKOV3_2, Scan: 9621, m/z: 837.452, Charge: 2, RT (min): 22.25, Score: 34.36 (H31K27Malonyl)
Intensity
100%
80%
60%
40%
20%
0%
0
200
400
600
800
1000
1200
1400
m/z
prK
y1+
b1+ -CO2
b5++ -CO2
b2+ -CO2
y2+
b2+ -H2O
b2+
b3+ -CO2
b3+ -H2O
b3+
y3+
y8++
y9++ -H2O
y9++
y4+
y12++
y5+
y6+
y7+
y8+
y9+
y10+
y11+
y12+
y12 y11 y10 y9 y8 y7 y6 y5 y4 y3 y2 y1
(ac)-K(ma) S A P A T G G V K(pr) K(pr) P H R
b2 b3

Raw file: QEP220103_RN_SKOV3_2, Scan: 14983, m/z: 869.9739, Charge: 2, RT (min): 32.69, Score: 35.058 (H4K79Lactoyl|Carboxyethyl)

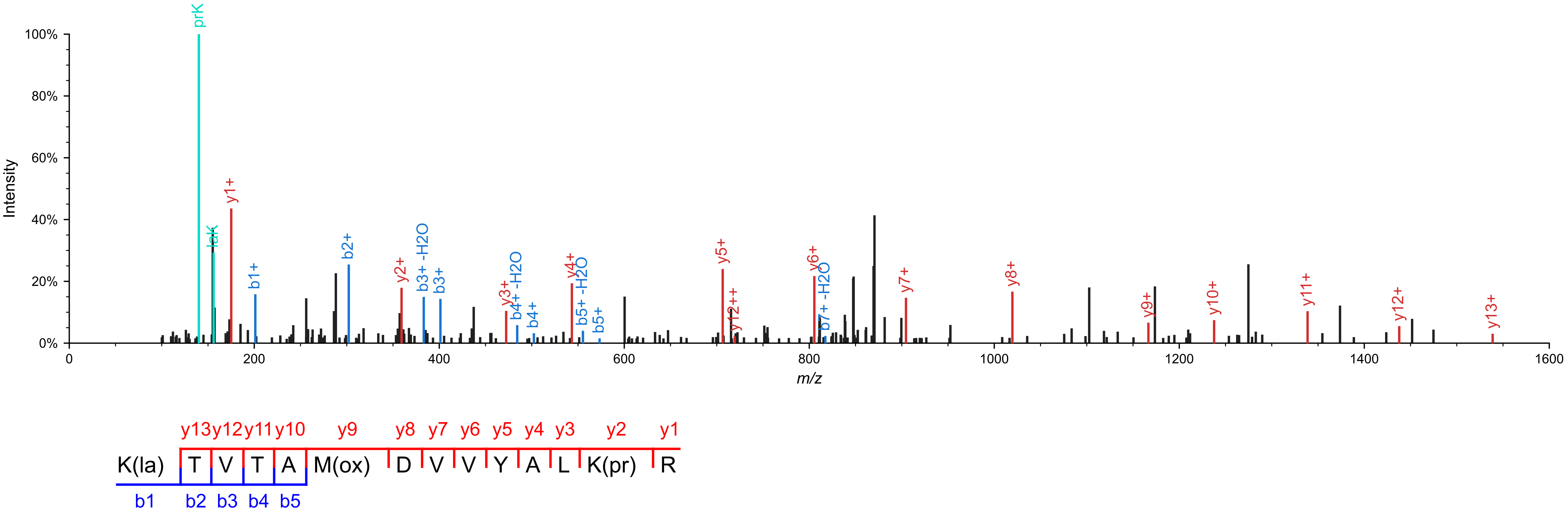

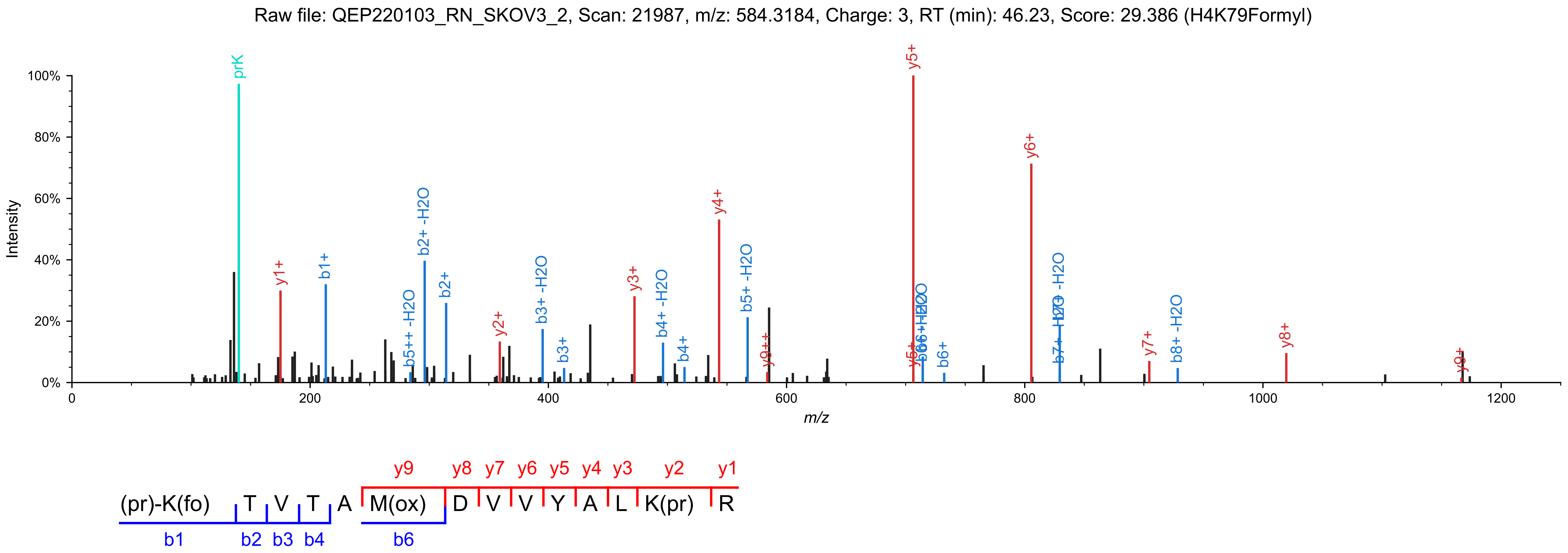

Raw file: QEP220103_RN_SKOV3_2, Scan: 21987, m/z: 584.3184, Charge: 3, RT (min): 46.23, Score: 29.386 (H4K79Formyl)
Intensity
m/z
prK
y1+
b1+
b2+ -H2O
b2+
y2+
b3+ -H2O
b3+
y3+
b4+ -H2O
b4+
y4+
b5+ -H2O
y9++
y5+
y6+
b6+
b8+ -H2O
y7+
y8+
y9+
(pr)-K(fo) T V T A M(ox) D V V Y A L K(pr) R
y9 y8 y7 y6 y5 y4 y3 y2 y1
b1 b2 b3 b4 b6

Raw file: QEP220103_RN_SKOV3_2, Scan: 22018, m/z: 796.4292, Charge: 2, RT (min): 46.29, Score: 32.03 (H31K122Lactoyl|Carboxyethyl)

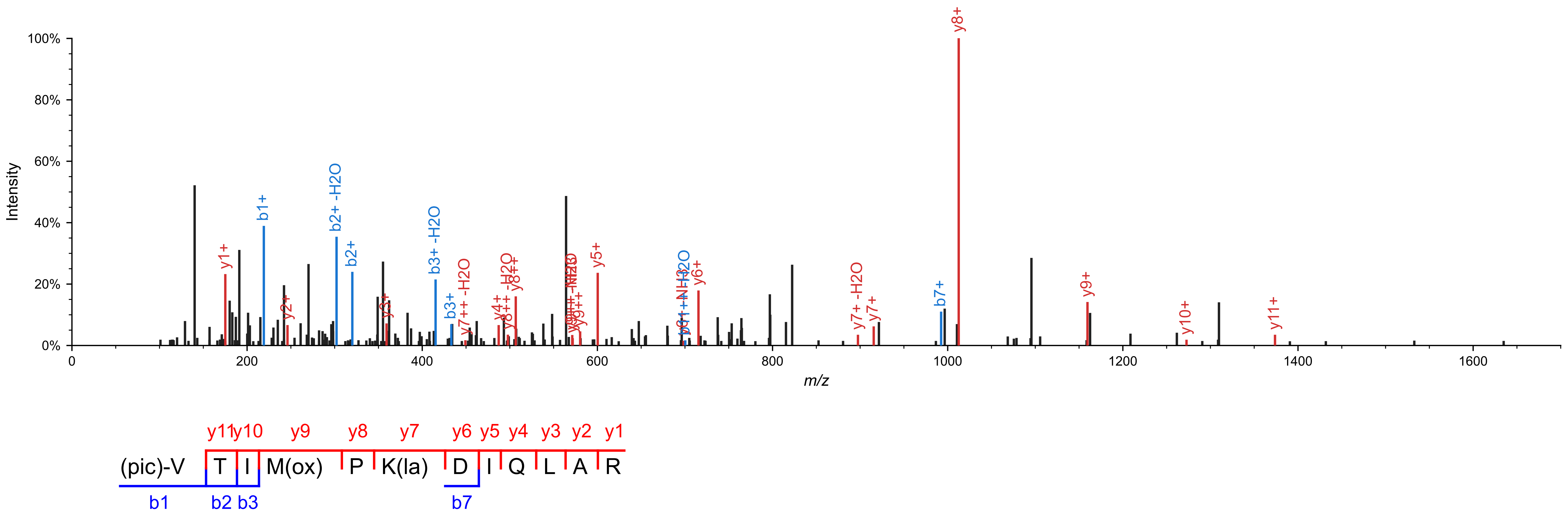

Raw file: QEP220103_RN_SKOV3_3, Scan: 5715, m/z: 630.8691, Charge: 2, RT (min): 14.79, Score: 31.352 (H31K37Formyl)

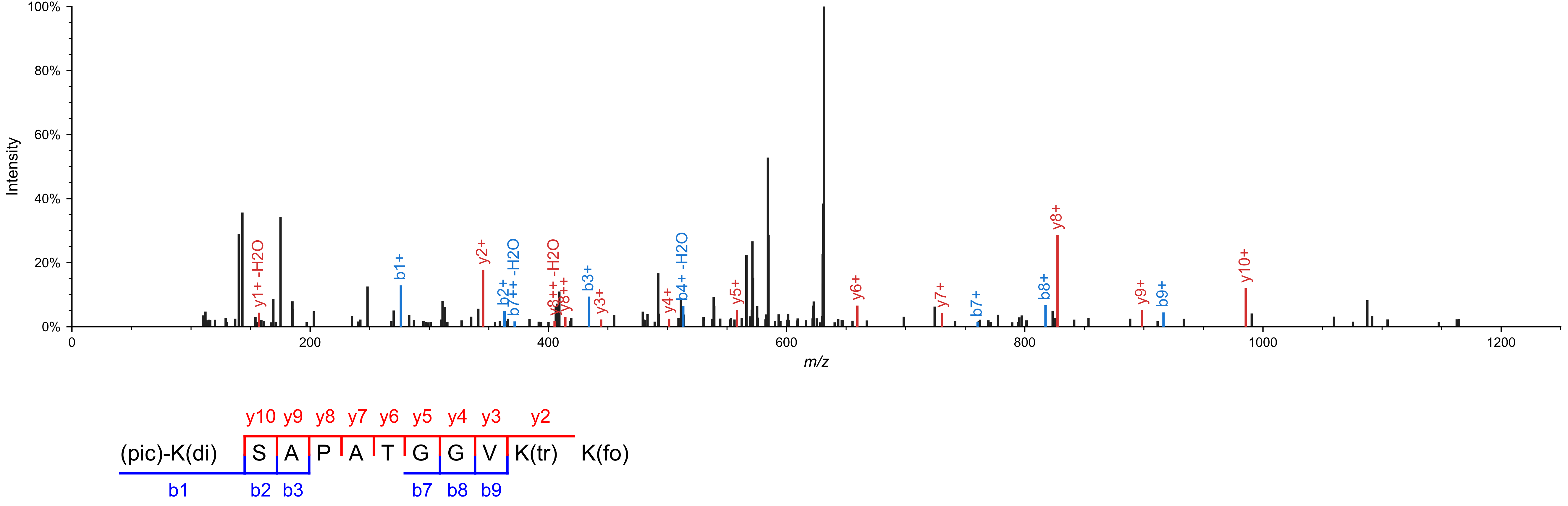

Raw file: QEP220103_RN_SKOV3_3, Scan: 21000, m/z: 699.3747, Charge: 2, RT (min): 44.42, Score: 22.707 (H31K56Formyl)

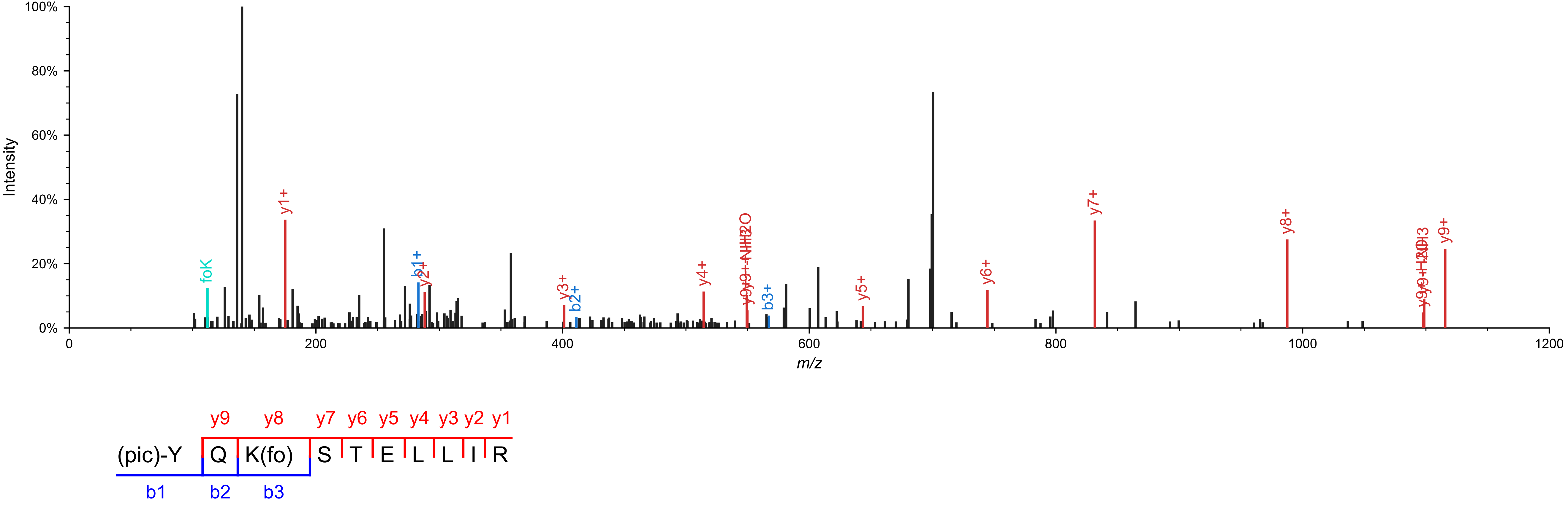

Raw file: QEP220103_RN_SKOV3_3, Scan: 23508, m/z: 763.8778, Charge: 2, RT (min): 49.38, Score: 27.15 (H31K79Lactoyl|Carboxyethyl)

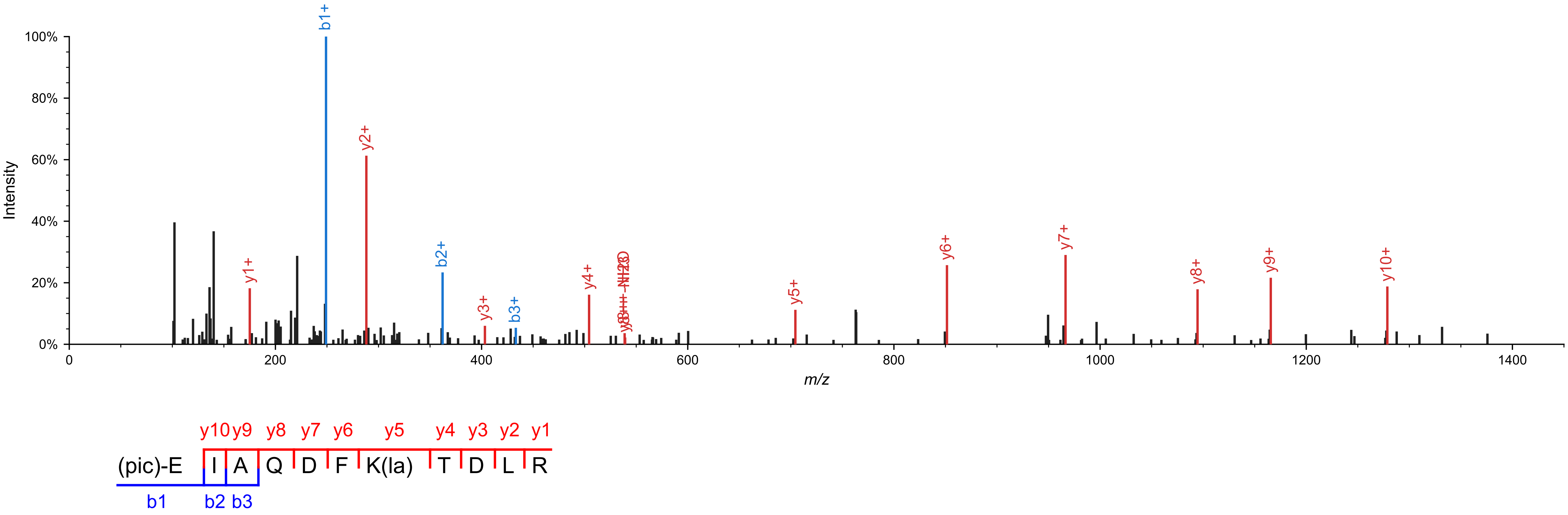

Raw file: QEP220103_RN_SKOV3_3, Scan: 25839, m/z: 907.4794, Charge: 2, RT (min): 54.07, Score: 26.031 (H4K91Formyl)

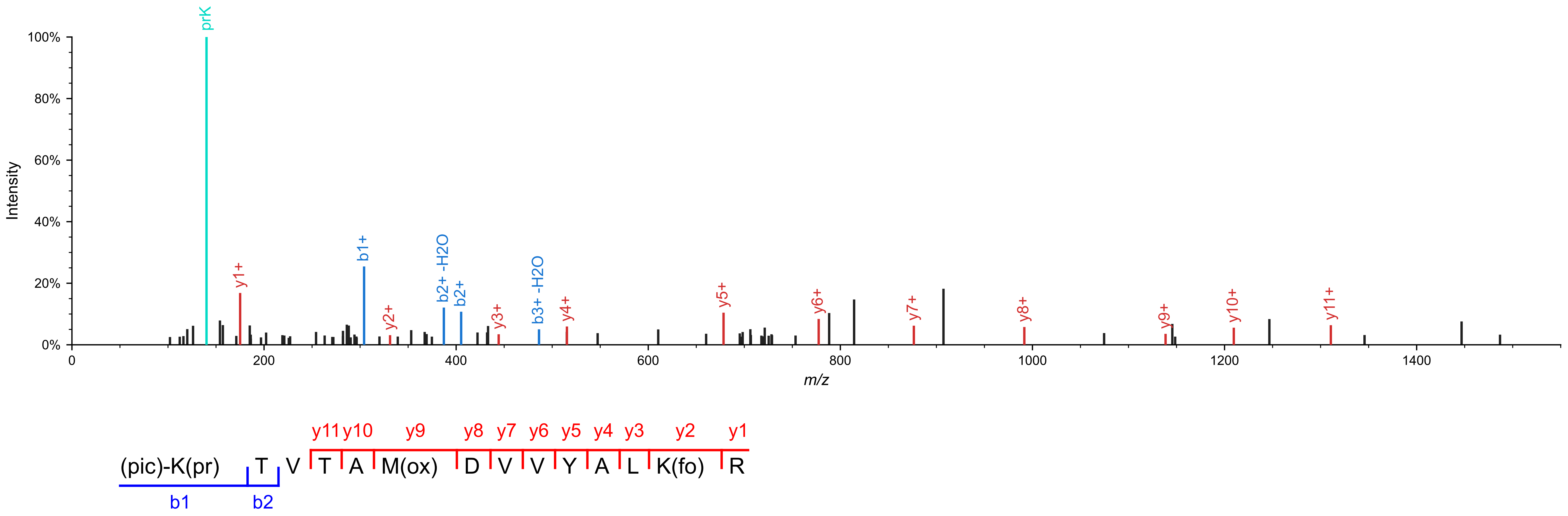

Raw file: QEP220103_RN_SKOV3_3, Scan: 27748, m/z: 897.9689, Charge: 2, RT (min): 58.01, Score: 29.576 (H4K79Malonyl)

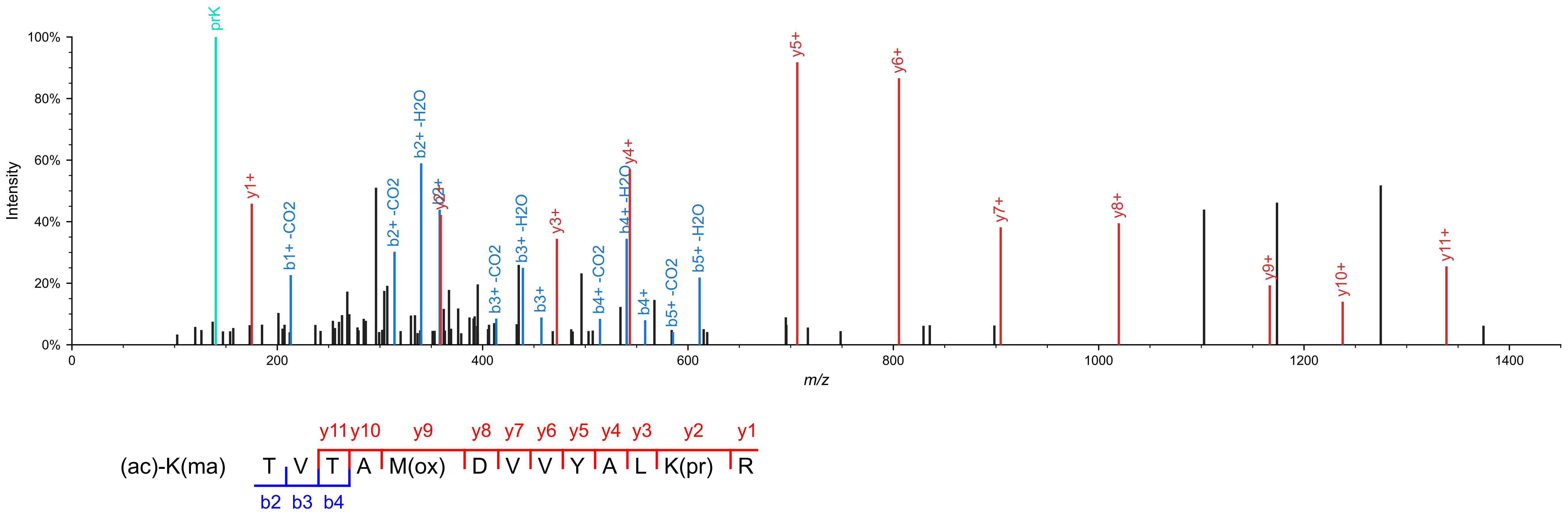

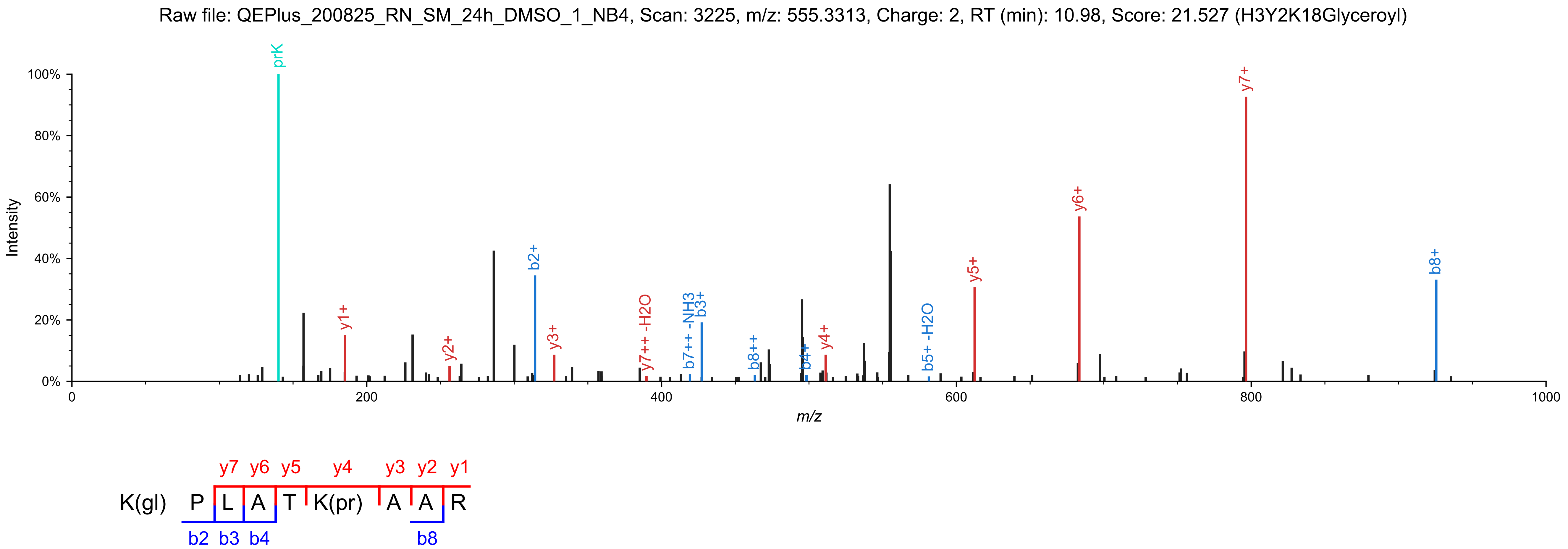

Raw file: QEPlus_200825_RN_SM_24h_DMSO_1_NB4, Scan: 3225, m/z: 555.3313, Charge: 2, RT (min): 10.98, Score: 21.527 (H3Y2K18Glyceroyl)
Intensity
m/z
prK
y1+
y2+
y3+
y4+
y5+
y6+
y7+
y7++ -H2O
b2+
b3+
b4+
b8+
b8++
b7++ -NH3
b5+ -H2O
K(gl) P L A T K(pr) A A R
y7 y6 y5 y4 y3 y2 y1
b2 b3 b4 b8

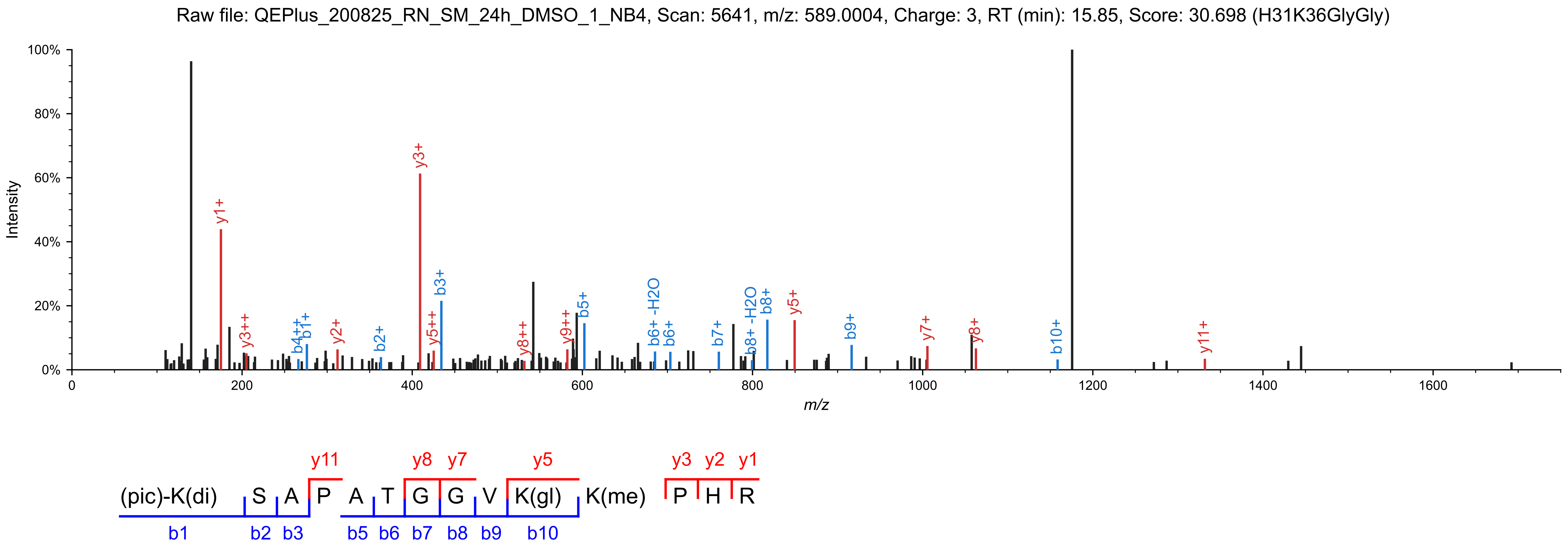

Raw file: QEPlus_200825_RN_SM_24h_DMSO_1_NB4, Scan: 5641, m/z: 589.0004, Charge: 3, RT (min): 15.85, Score: 30.698 (H31K36GlyGly)
Intensity
m/z
(pic)-K(di) S A P A T G G V K(gl) K(me) P H R

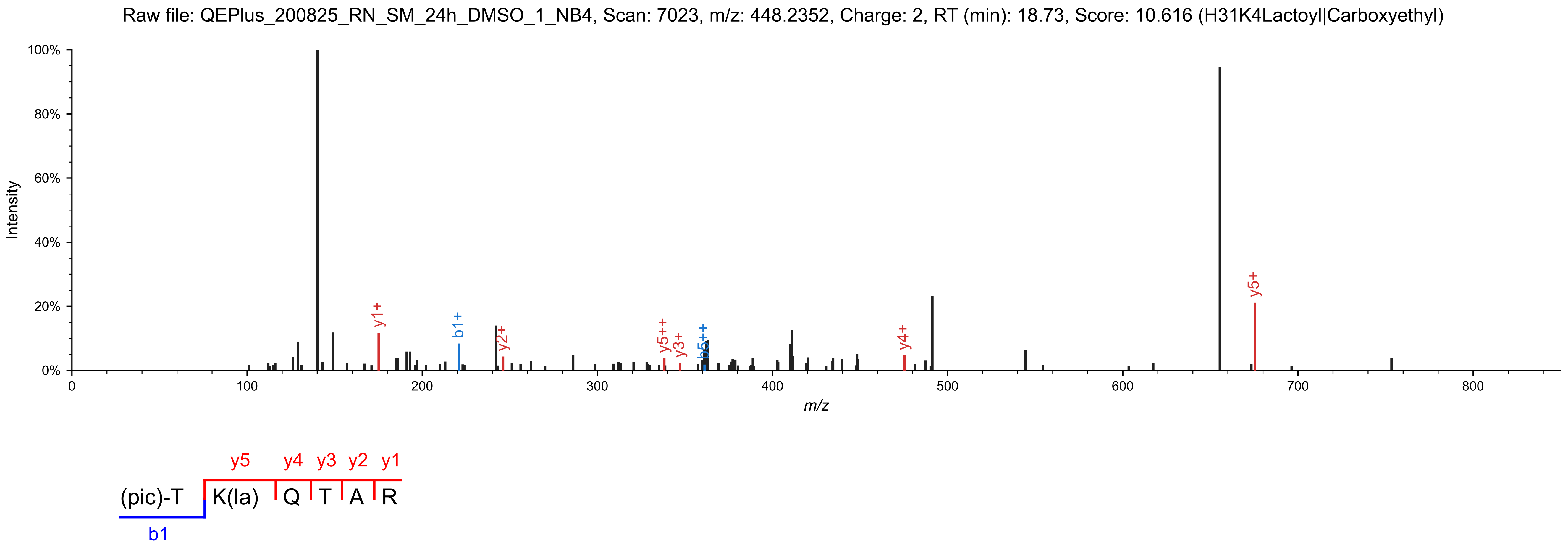

Raw file: QEPlus_200825_RN_SM_24h_DMSO_1_NB4, Scan: 7023, m/z: 448.2352, Charge: 2, RT (min): 18.73, Score: 10.616 (H31K4Lactoyl|Carboxyethyl)
Intensity
100%
80%
60%
40%
20%
0%
0
100
200
300
400
500
600
700
800
m/z
y1+
b1+
y2+
y5++
y3+
b5++
y4+
y5+
y5
y4
y3
y2
y1
(pic)-T
K(la)
Q
T
A
R
b1

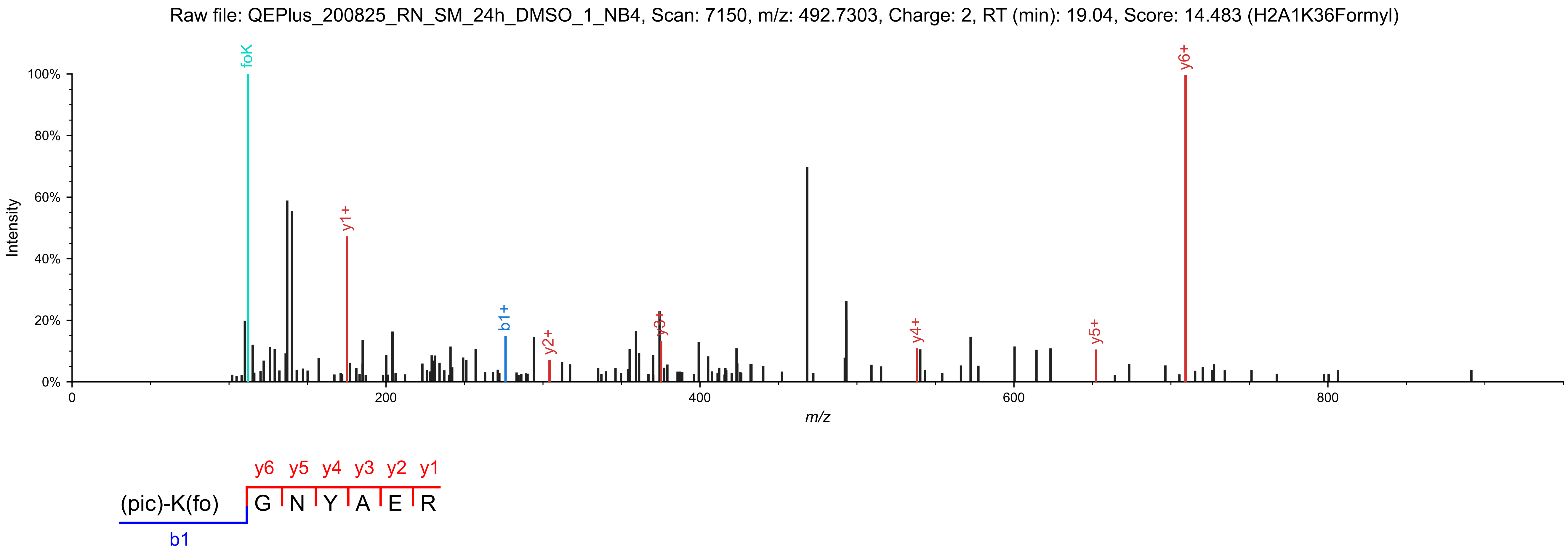
Raw file: QEPlus_200825_RN_SM_24h_DMSO_1_NB4, Scan: 7150, m/z: 492.7303, Charge: 2, RT (min): 19.04, Score: 14.483 (H2A1K36Formyl)
foK
y1+
b1+
y2+
y3+
y4+
y5+
y6+
Intensity
100%
80%
60%
40%
20%
0%
0
200
400
600
800
m/z
y6
y5
y4
y3
y2
y1
(pic)-K(fo)
G
N
Y
A
E
R
b1

Raw file: QEPlus_200825_RN_SM_24h_DMSO_1_NB4, Scan: 9201, m/z: 581.8143, Charge: 2, RT (min): 23.26, Score: 19.679 (H31K9Hydroxyisobutyryl)

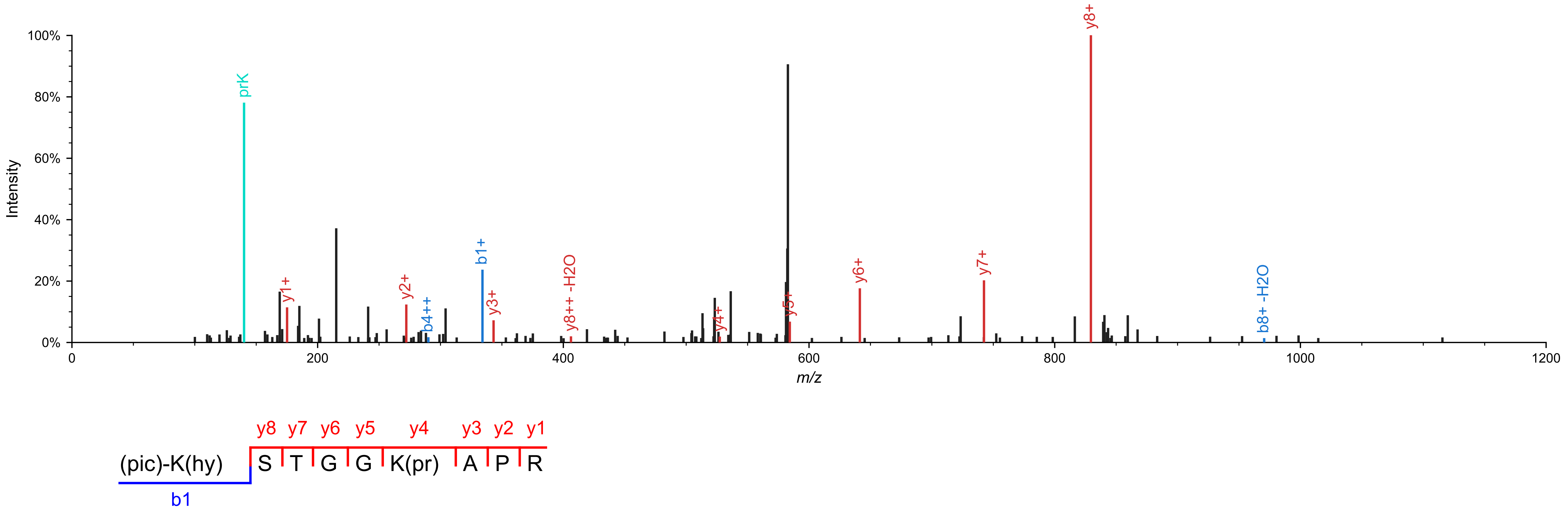

Raw file: QEPlus_200825_RN_SM_24h_DMSO_1_NB4, Scan: 9379, m/z: 880.9876, Charge: 2, RT (min): 23.66, Score: 30.98 (H31K27Hydroxyisobutyryl)

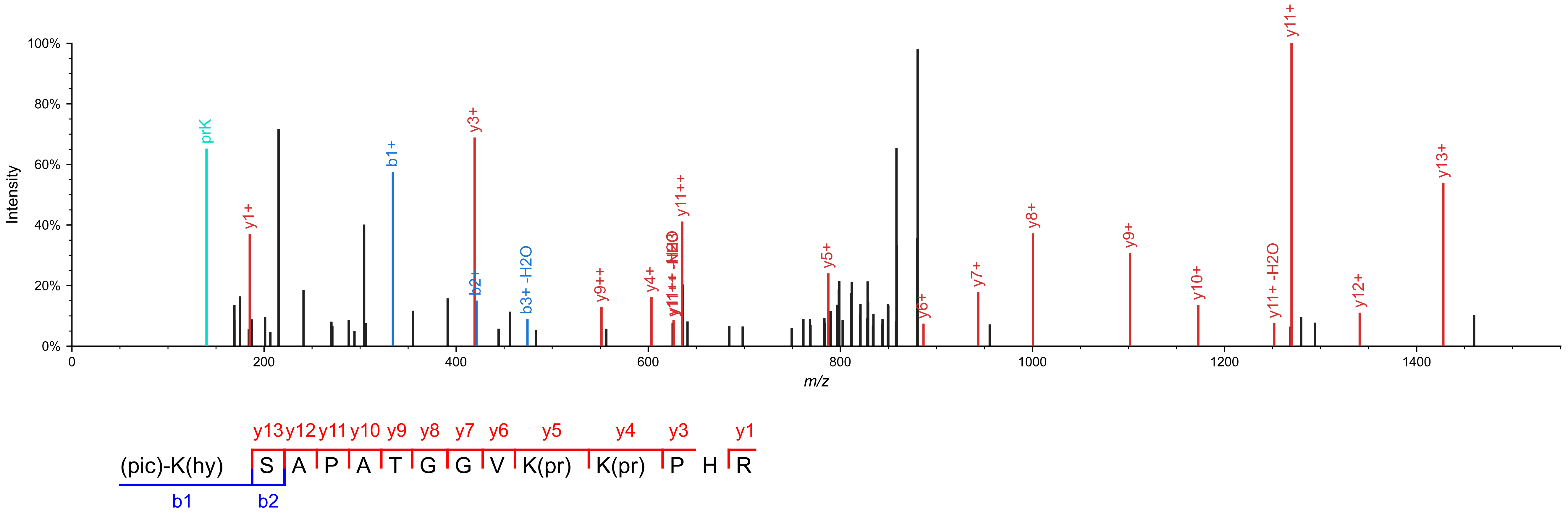

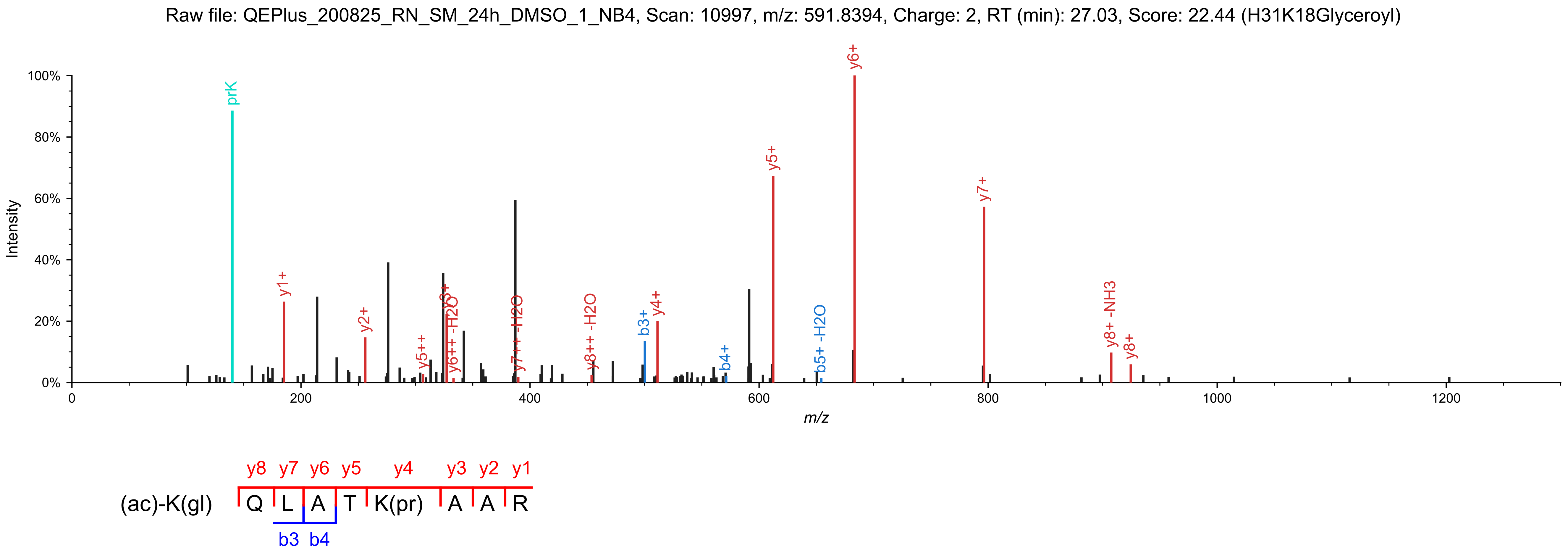

Raw file: QEPlus_200825_RN_SM_24h_DMSO_1_NB4, Scan: 10997, m/z: 591.8394, Charge: 2, RT (min): 27.03, Score: 22.44 (H31K18Glyceroyl)
Intensity
m/z
100%
80%
60%
40%
20%
0%
0
200
400
600
800
1000
1200
prK
y1+
y2+
y5++
y3+
y6++ -H2O
y7++ -H2O
y8++ -H2O
b3+
y4+
b4+
y5+
b5+ -H2O
y6+
y7+
y8+ -NH3
y8+
y8 y7 y6 y5 y4 y3 y2 y1
(ac)-K(gl) Q L A T K(pr) A A R
b3 b4

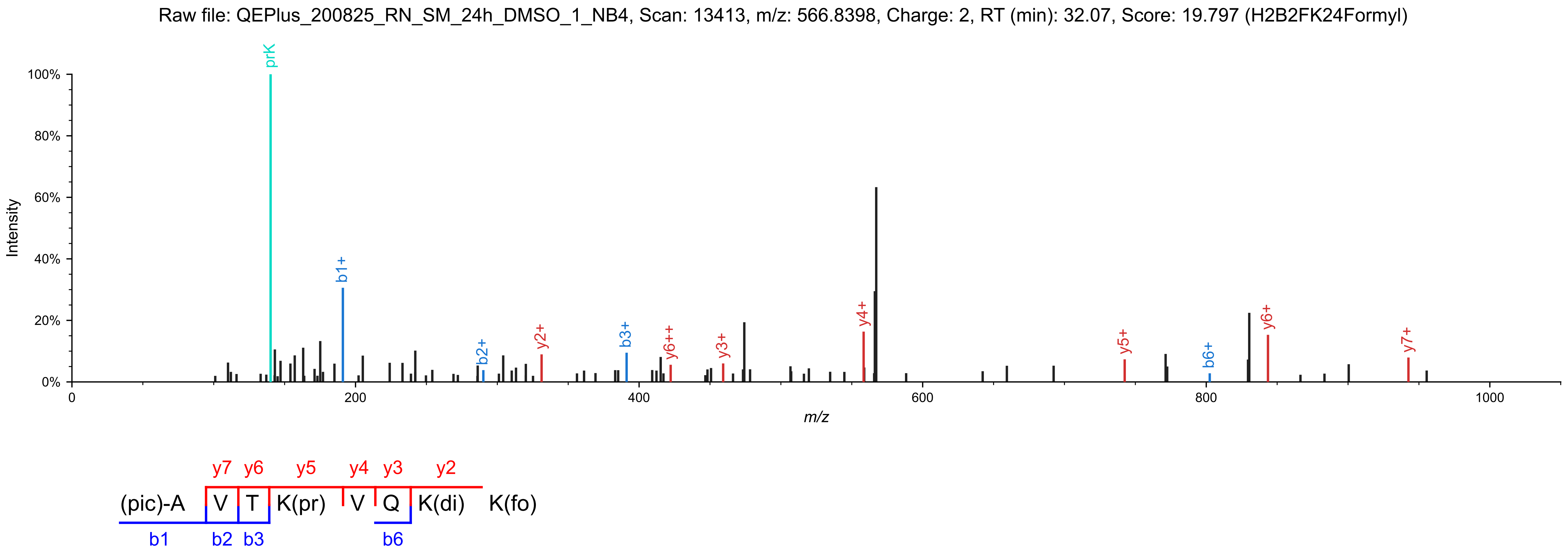
Raw file: QEPlus_200825_RN_SM_24h_DMSO_1_NB4, Scan: 13413, m/z: 566.8398, Charge: 2, RT (min): 32.07, Score: 19.797 (H2B2FK24Formyl)
prK
b1+
b2+
y2+
b3+
y6++
y3+
y4+
y5+
b6+
y6+
y7+
Intensity
100%
80%
60%
40%
20%
0%
0
200
400
600
800
1000
m/z
y7 y6 y5 y4 y3 y2
(pic)-A V T K(pr) V Q K(di) K(fo)
b1 b2 b3 b6

Raw file: QEPlus_200825_RN_SM_24h_DMSO_1_NB4, Scan: 14720, m/z: 611.7963, Charge: 2, RT (min): 34.89, Score: 20.554 (H31S10Phosphoryl)

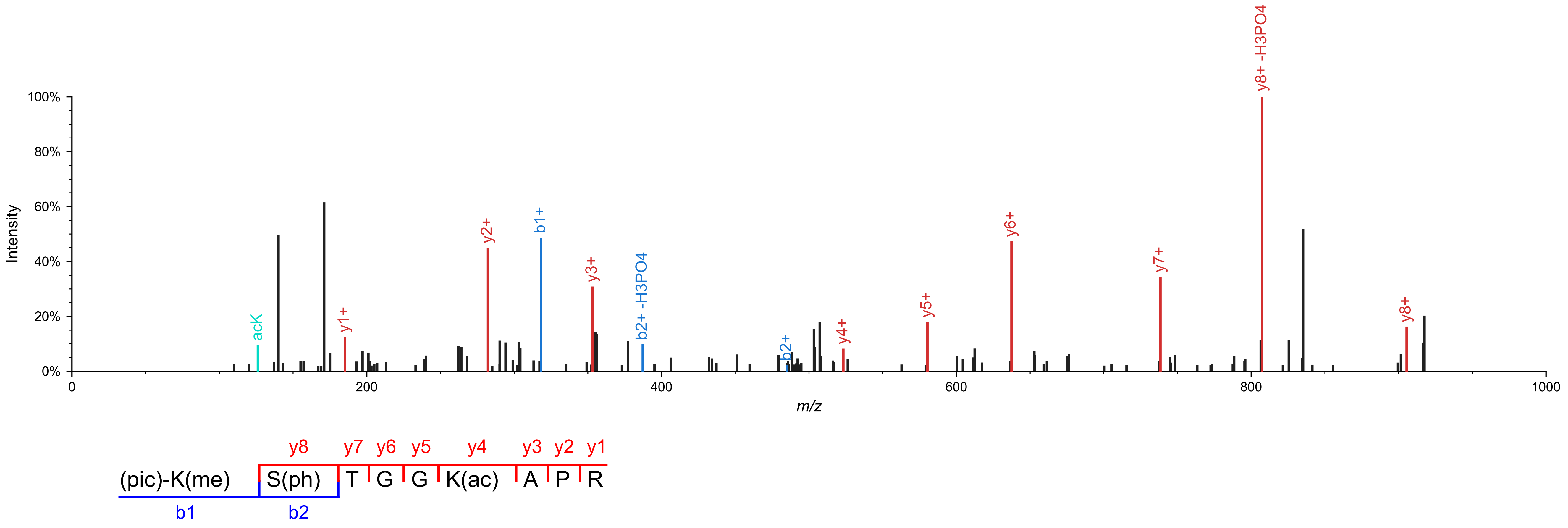

Raw file: QEPlus_200825_RN_SM_24h_DMSO_1_NB4, Scan: 14907, m/z: 600.3421, Charge: 2, RT (min): 35.28, Score: 22.612 (H31K18Formyl)

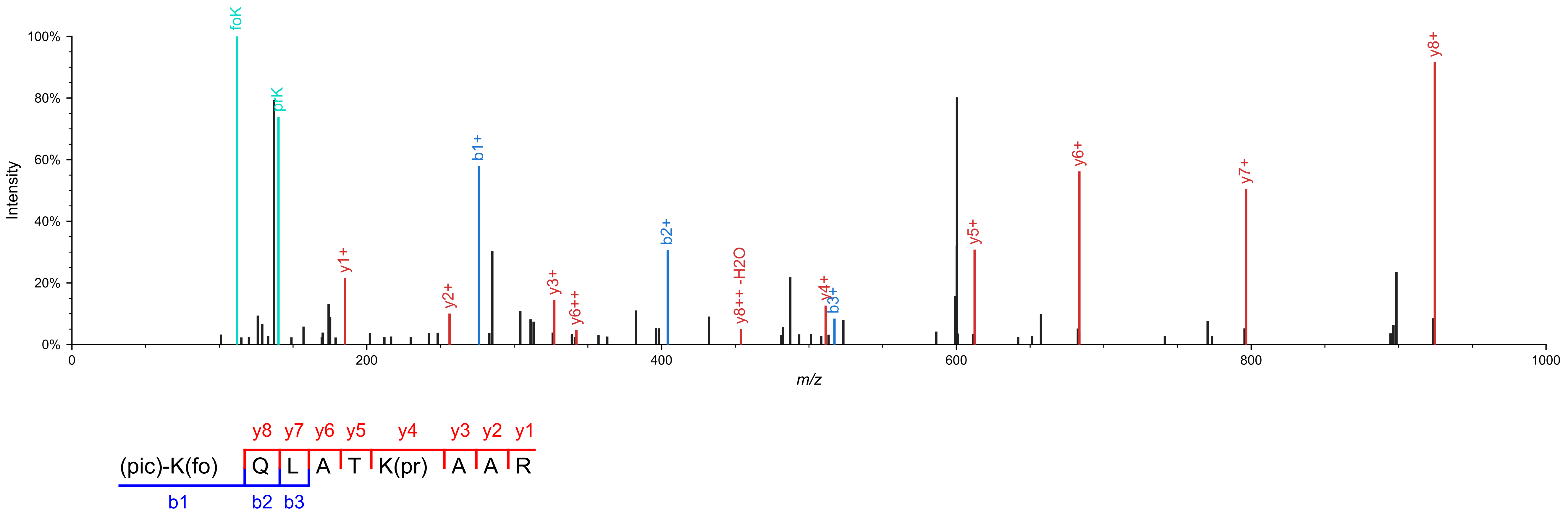

Raw file: QEPlus_200825_RN_SM_24h_DMSO_1_NB4, Scan: 15070, m/z: 595.338, Charge: 2, RT (min): 35.61, Score: 21.407 (H31K23Formyl)

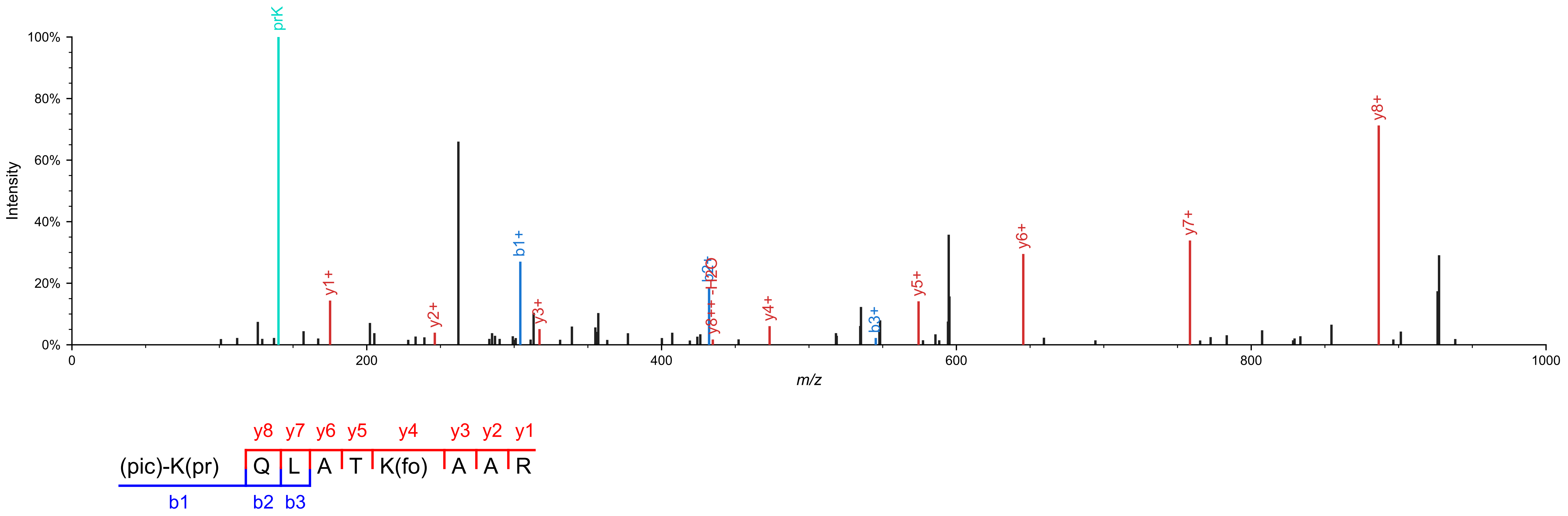

Raw file: QEPlus_200825_RN_SM_24h_DMSO_1_NB4, Scan: 15720, m/z: 765.9173, Charge: 2, RT (min): 36.99, Score: 32.337 (H4K31Hydroxyisobutyryl)

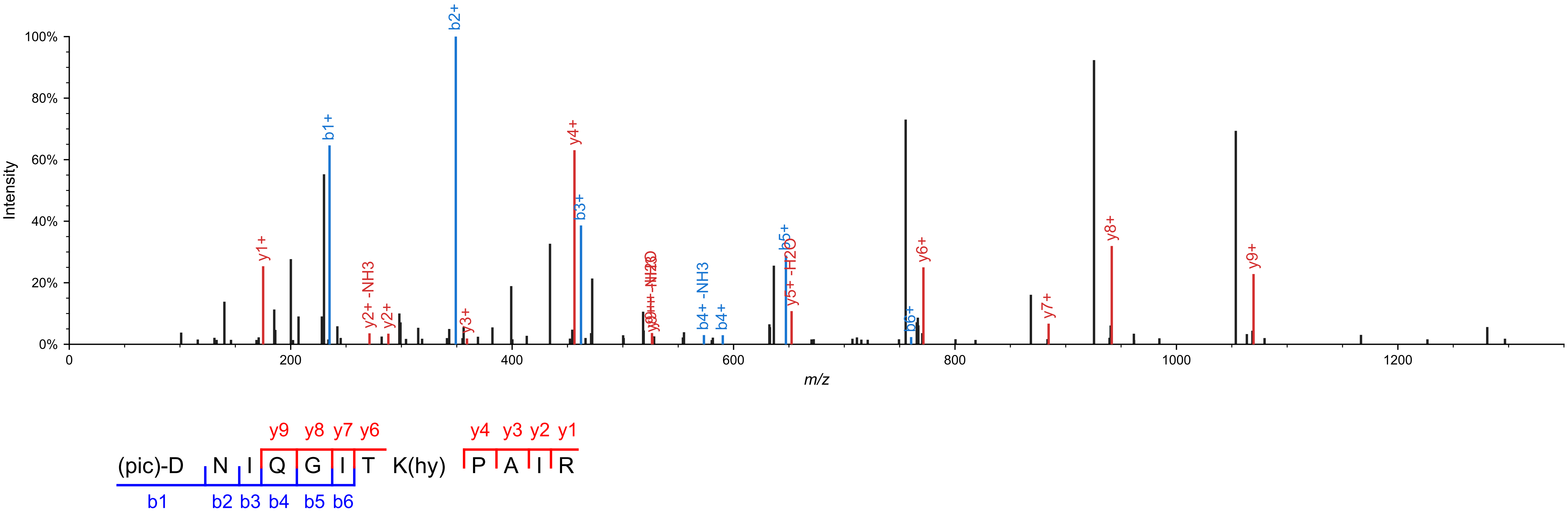

Raw file: QEPlus_200825_RN_SM_24h_DMSO_2_NB4, Scan: 7710, m/z: 546.7828, Charge: 2, RT (min): 19.81, Score: 17.81 (H2B1BK85Lactoyl|Carboxyethyl)

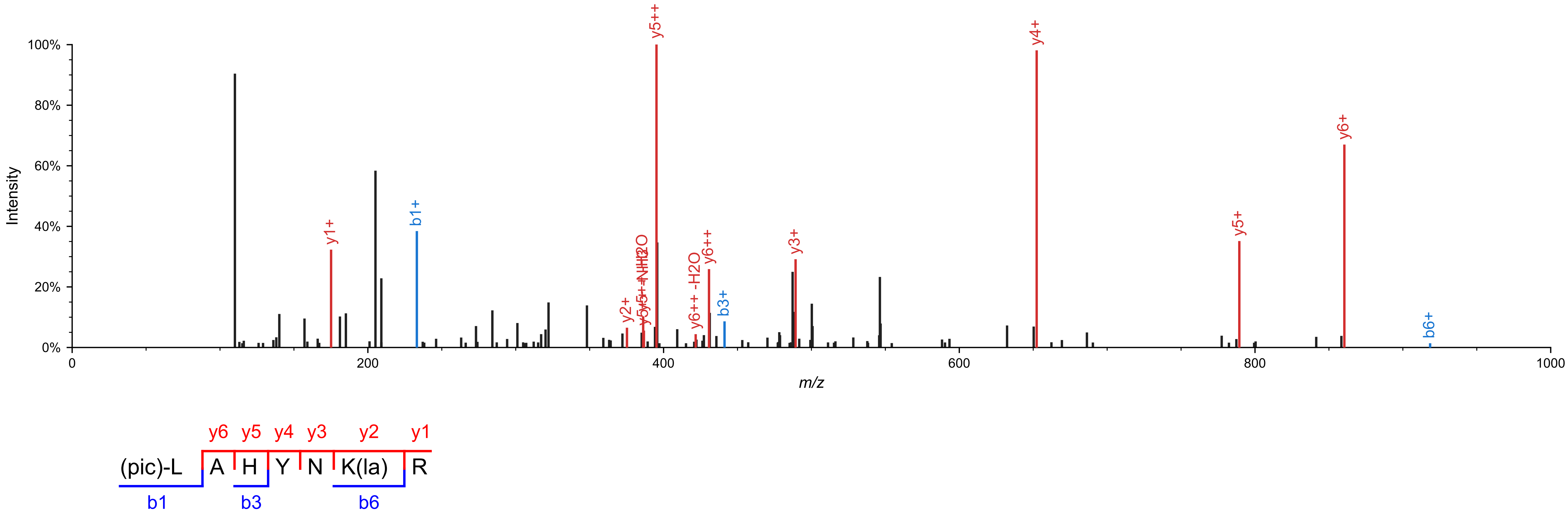

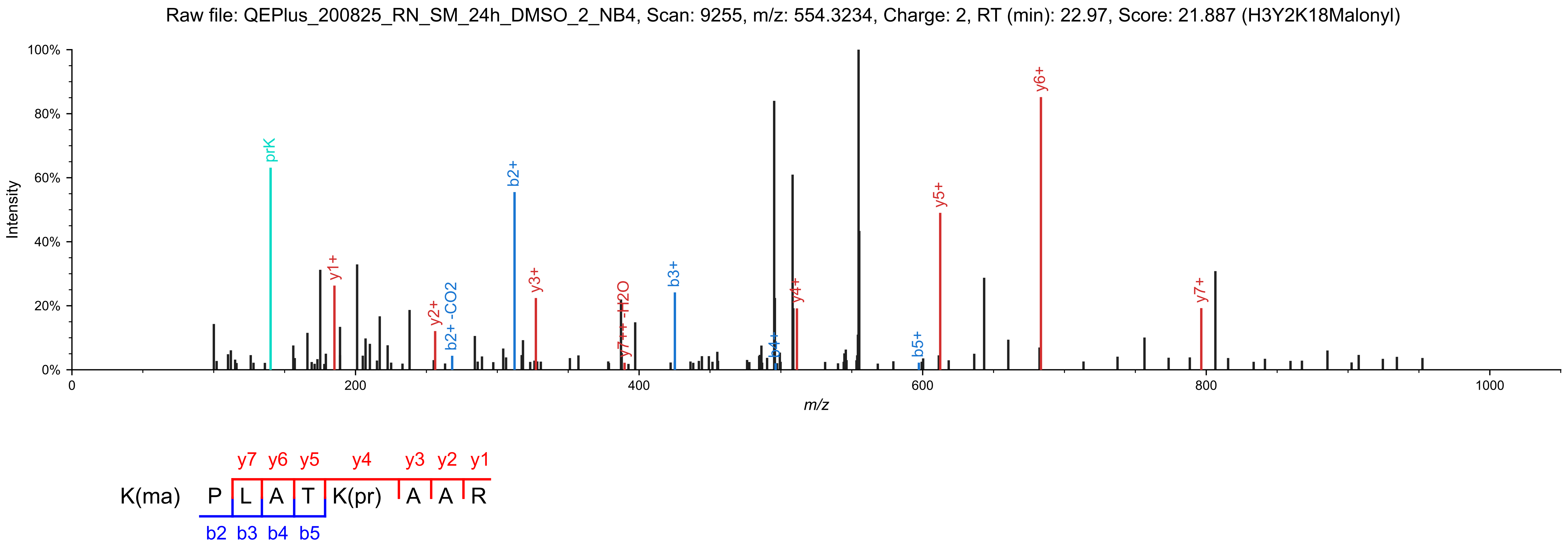

Raw file: QEPlus_200825_RN_SM_24h_DMSO_2_NB4, Scan: 9255, m/z: 554.3234, Charge: 2, RT (min): 22.97, Score: 21.887 (H3Y2K18Malonyl)
Intensity
m/z
prK
y1+
y2+
b2+ -CO2
b2+
y3+
y7++ -H2O
b3+
b4+
y4+
b5+
y5+
y6+
y7+
K(ma) P L A T K(pr) A A R
y7 y6 y5 y4 y3 y2 y1
b2 b3 b4 b5

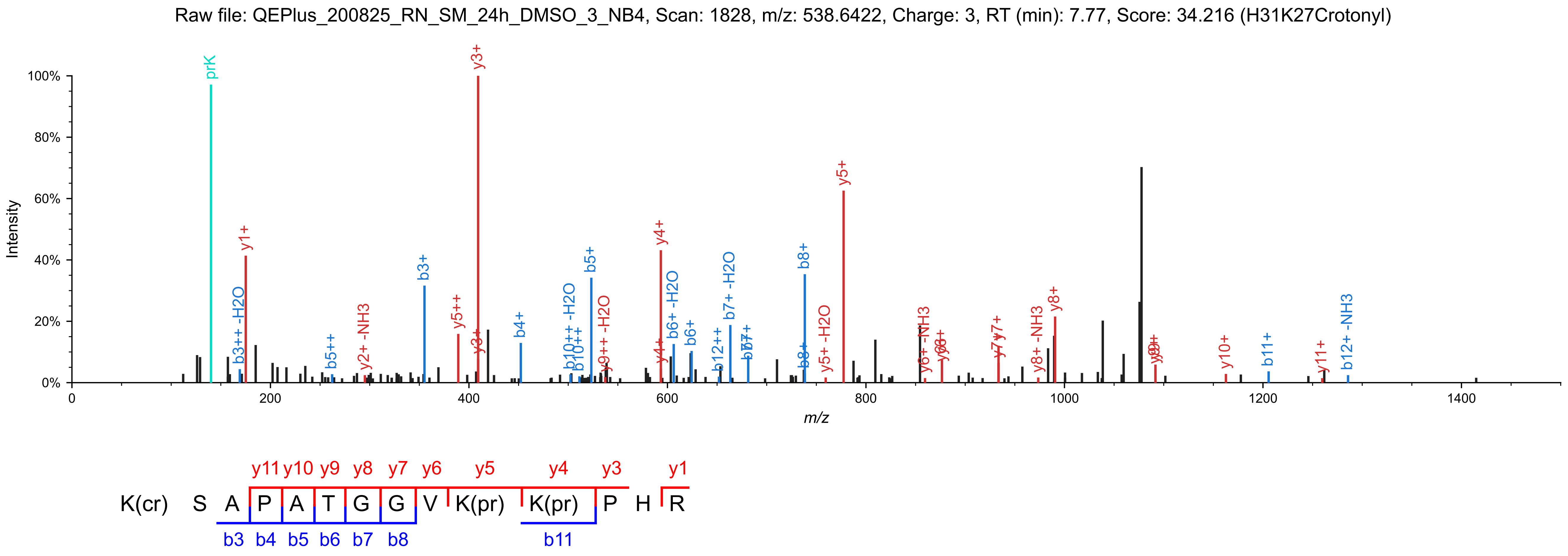
Raw file: QEPlus_200825_RN_SM_24h_DMSO_3_NB4, Scan: 1828, m/z: 538.6422, Charge: 3, RT (min): 7.77, Score: 34.216 (H31K27Crotonyl)
Intensity
100%
80%
60%
40%
20%
0%
0
200
400
600
800
1000
1200
1400
m/z
prK
y3+
y5+
y1+
y4+
b8+
b5+
b3+
y8+
b3++ -H2O
y5++
b4+
b10++ -H2O
b6+ -H2O
b7+ -H2O
y2+ -NH3
y9++ -H2O
y5+ -H2O
y8+ -NH3
y10+
b11+
y11+
b12+ -NH3
b12++
b6+
b5++
y11 y10 y9 y8 y7 y6 y5 y4 y3 y1
K(cr) S A P A T G G V K(pr) K(pr) P H R
b3 b4 b5 b6 b7 b8 b11

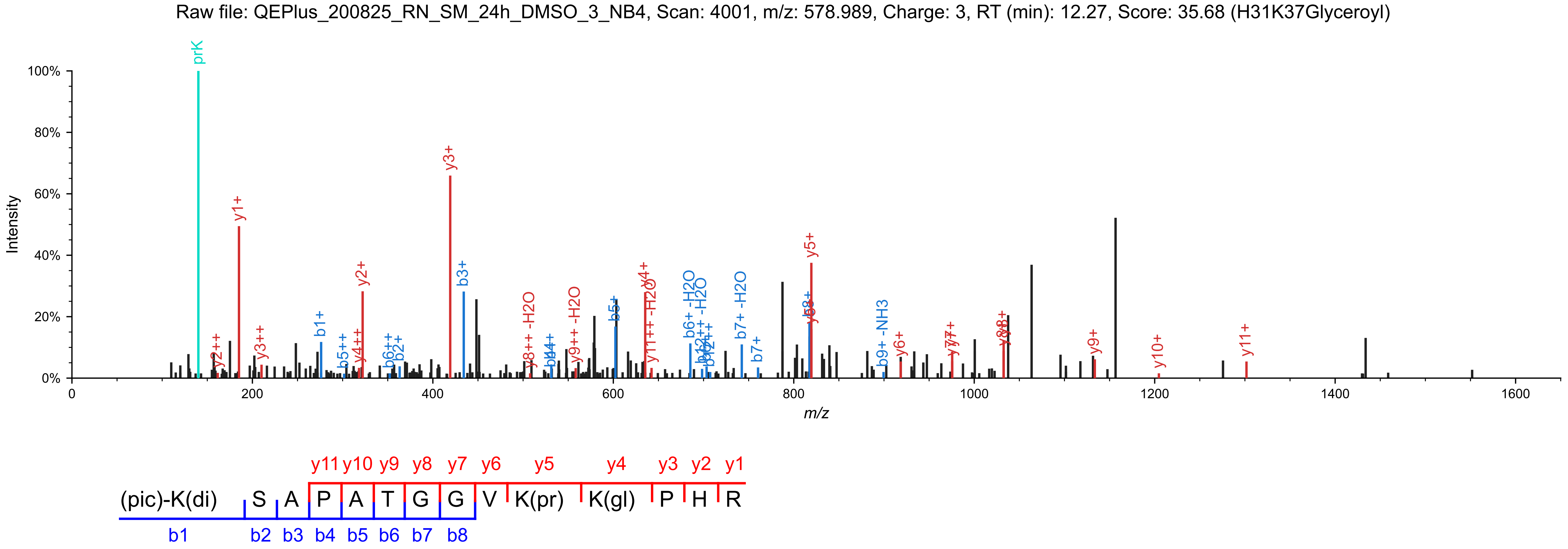
Raw file: QEPlus_200825_RN_SM_24h_DMSO_3_NB4, Scan: 4001, m/z: 578.989, Charge: 3, RT (min): 12.27, Score: 35.68 (H31K37Glyceroyl)
Intensity
m/z
(pic)-K(di) S A P A T G G V K(pr) K(gl) P H R
y11 y10 y9 y8 y7 y6 y5 y4 y3 y2 y1
b1 b2 b3 b4 b5 b6 b7 b8

Raw file: QEPlus_200825_RN_SM_24h_DMSO_3_NB4, Scan: 6014, m/z: 524.7803, Charge: 2, RT (min): 16.42, Score: 19.139 (H2A1K5Lactoyl|Carboxyethyl)

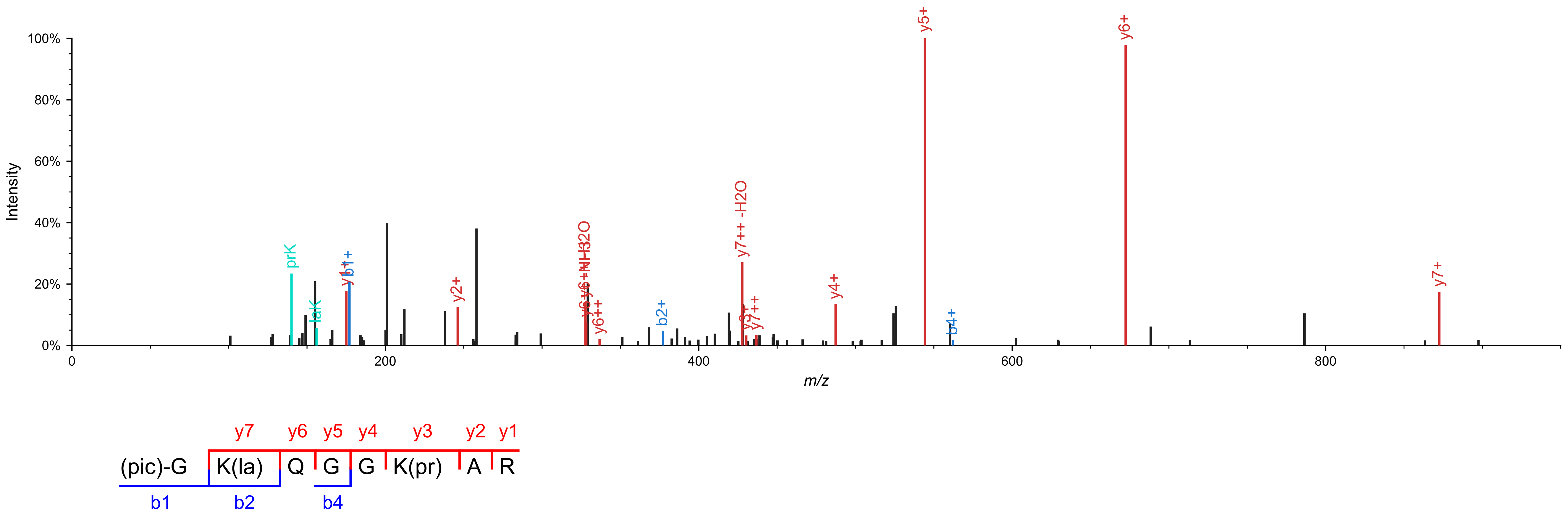

Raw file: QEPlus_200825_RN_SM_24h_DMSO_3_NB4, Scan: 6342, m/z: 577.9975, Charge: 3, RT (min): 17.09, Score: 29.396 (H33K27N-Pentosyl Lysine)

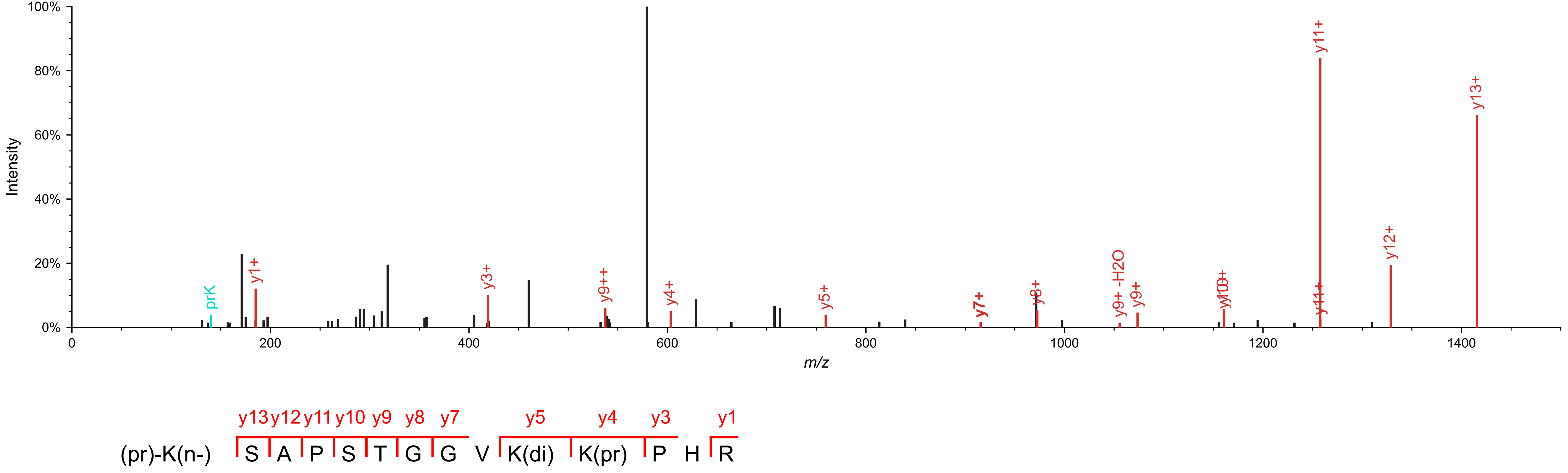

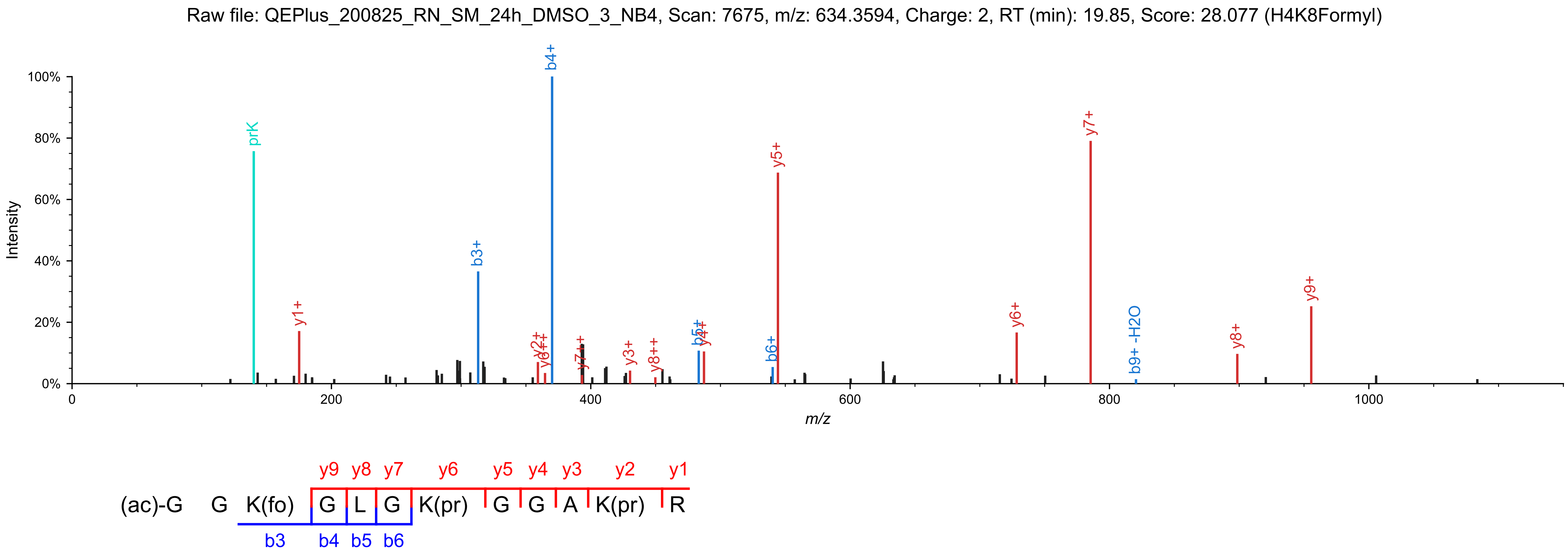

Raw file: QEPlus_200825_RN_SM_24h_DMSO_3_NB4, Scan: 7675, m/z: 634.3594, Charge: 2, RT (min): 19.85, Score: 28.077 (H4K8Formyl)
Intensity
m/z
prK
y1+
b3+
b4+
y2+
y3+
y4+
y5+
y6+
y7+
y8+
y9+
b5+
b6+
y7++
y8++
b9+ -H2O
(ac)-G G K(fo) G L G K(pr) G G A K(pr) R
y9 y8 y7 y6 y5 y4 y3 y2 y1
b3 b4 b5 b6

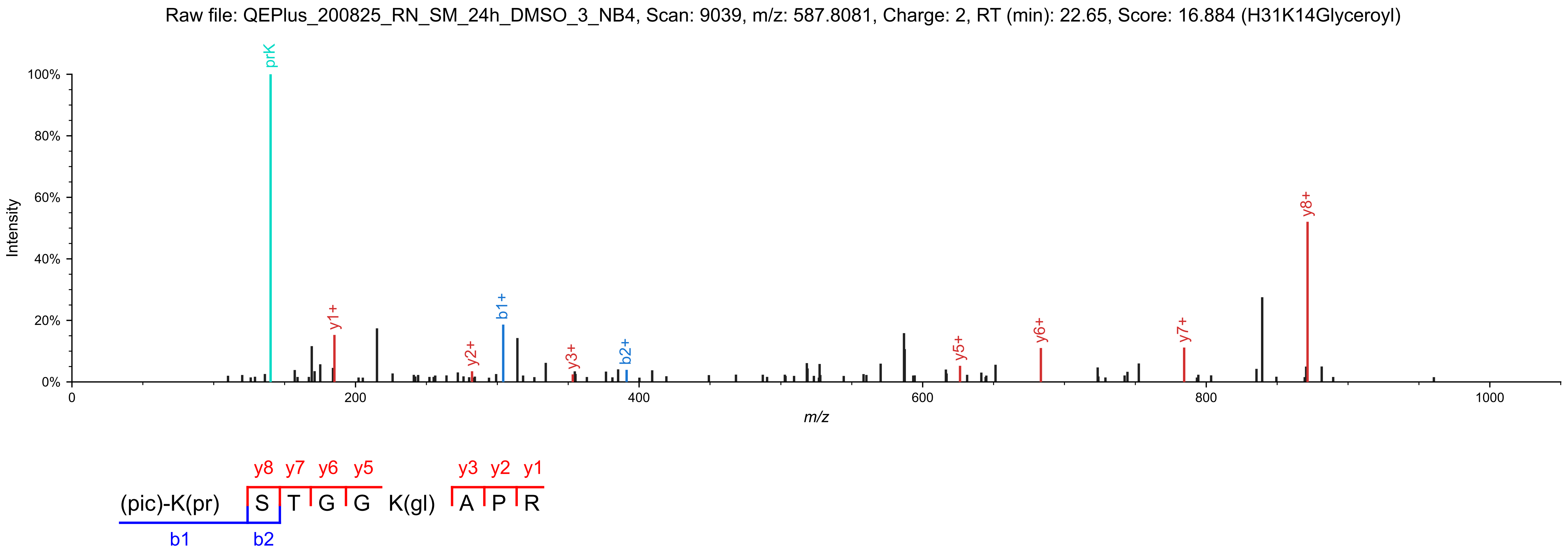

Raw file: QEPlus_200825_RN_SM_24h_DMSO_3_NB4, Scan: 9039, m/z: 587.8081, Charge: 2, RT (min): 22.65, Score: 16.884 (H31K14Glyceroyl)
Intensity
100%
80%
60%
40%
20%
0%
0
200
400
600
800
1000
m/z
prK
y1+
y2+
b1+
y3+
b2+
y5+
y6+
y7+
y8+
y8 y7 y6 y5
y3 y2 y1
(pic)-K(pr) S T G G K(gl) A P R
b1
b2

Linker histones

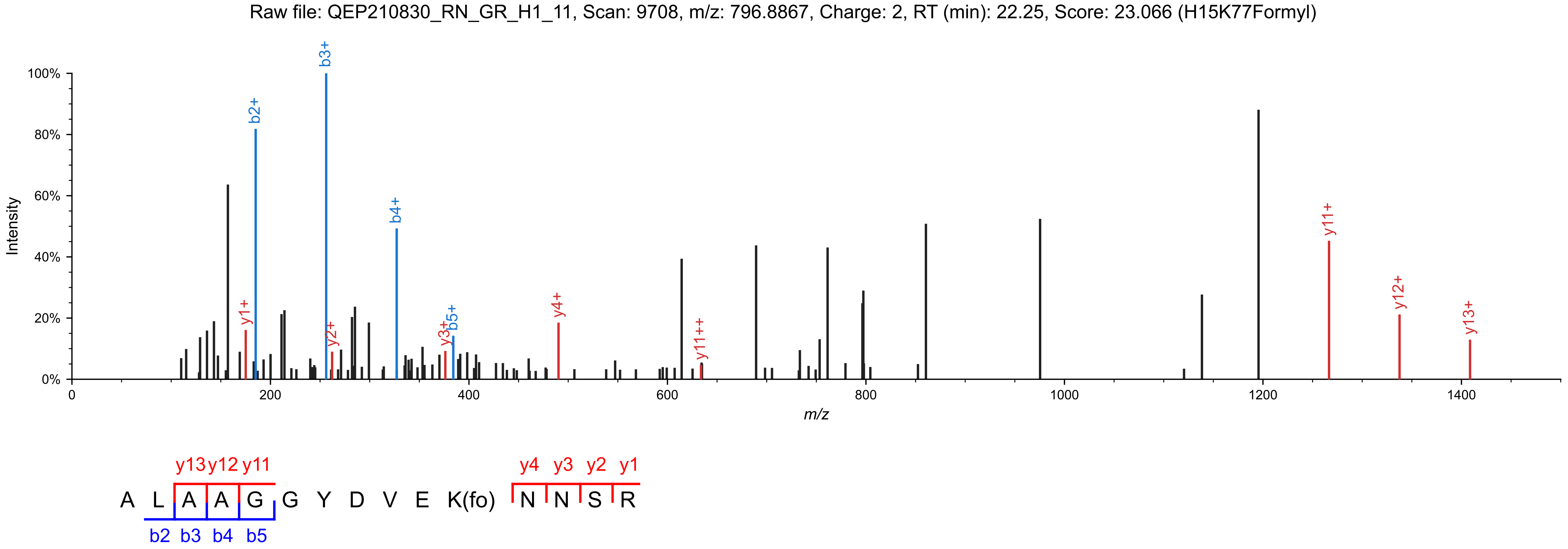

Raw file: QEP210830_RN_GR_H1_11, Scan: 9708, m/z: 796.8867, Charge: 2, RT (min): 22.25, Score: 23.066 (H15K77Formyl)
Intensity
100%
80%
60%
40%
20%
0%
0
200
400
600
800
1000
1200
1400
m/z
b2+
b3+
b4+
b5+
y1+
y2+
y3+
y4+
y11++
y11+
y12+
y13+
y13 y12 y11
y4 y3 y2 y1
A L A A G G Y D V E K(fo) N N S R
b2 b3 b4 b5

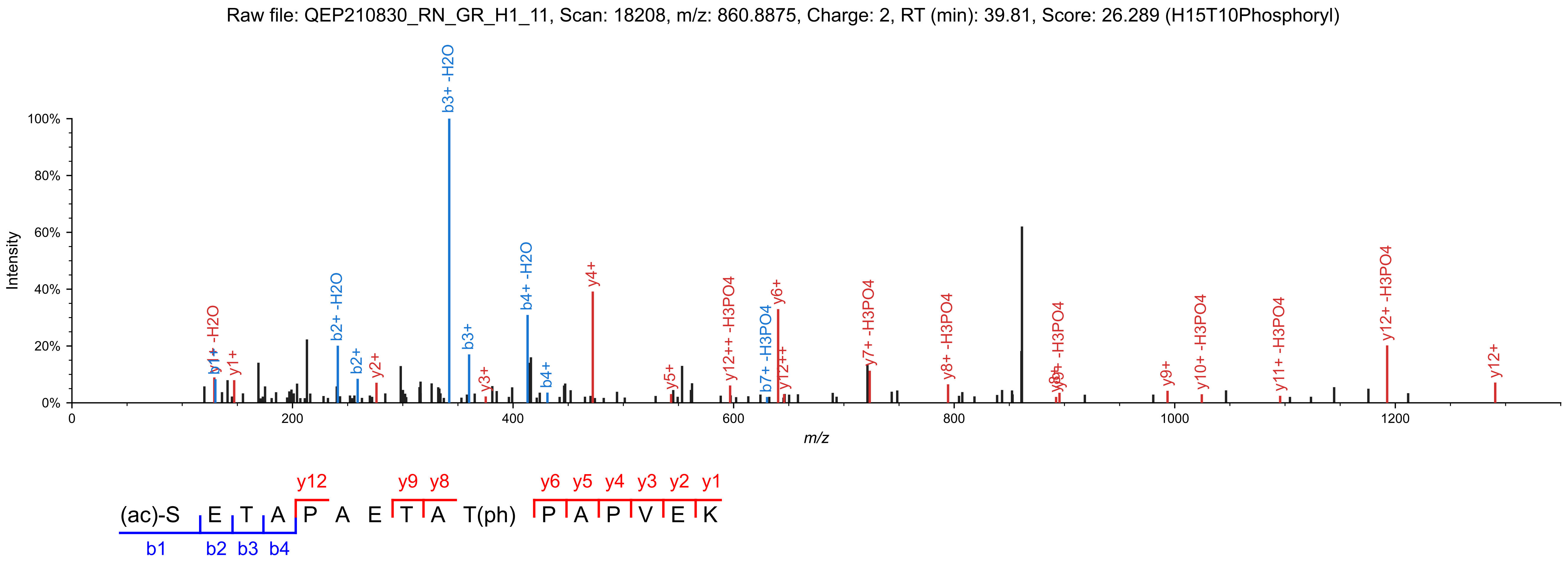

Raw file: QEP210830_RN_GR_H1_11, Scan: 18208, m/z: 860.8875, Charge: 2, RT (min): 39.81, Score: 26.289 (H15T10Phosphoryl)
Intensity
m/z
b3+ -H2O
b4+ -H2O
y4+
y6+
y12+ -H3PO4
b2+ -H2O
y1+ -H2O
b1+
y1+
b2+
y2+
b3+
y3+
b4+
y5+
y12++ -H3PO4
b7+ -H3PO4
y12++
y7+ -H3PO4
y8+ -H3PO4
y9+ -H3PO4
y9+
y10+ -H3PO4
y11+ -H3PO4
y12+
(ac)-S E T A P A E T A T(ph) P A P V E K
y12 y9 y8 y6 y5 y4 y3 y2 y1
b1 b2 b3 b4

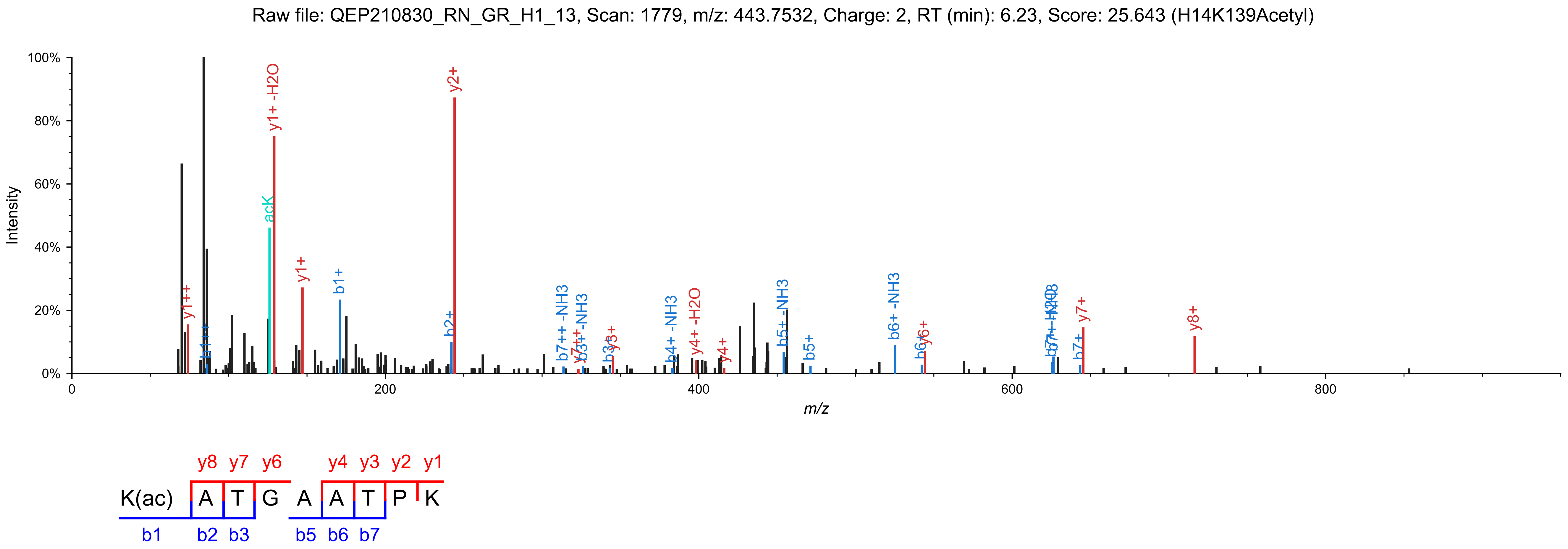
Raw file: QEP210830_RN_GR_H1_13, Scan: 1779, m/z: 443.7532, Charge: 2, RT (min): 6.23, Score: 25.643 (H14K139Acetyl)
Intensity
m/z
y1+ -H2O
y2+
acK
y1+
b1+
y1++
b2+
b7++ -NH3
b3+ -NH3
y4+ -H2O
b4+ -NH3
b5+ -NH3
b6+ -NH3
b5+
y4+
y7+
y8+
b7+
y6+
y8 y7 y6 y4 y3 y2 y1
K(ac) A T G A A T P K
b1 b2 b3 b5 b6 b7

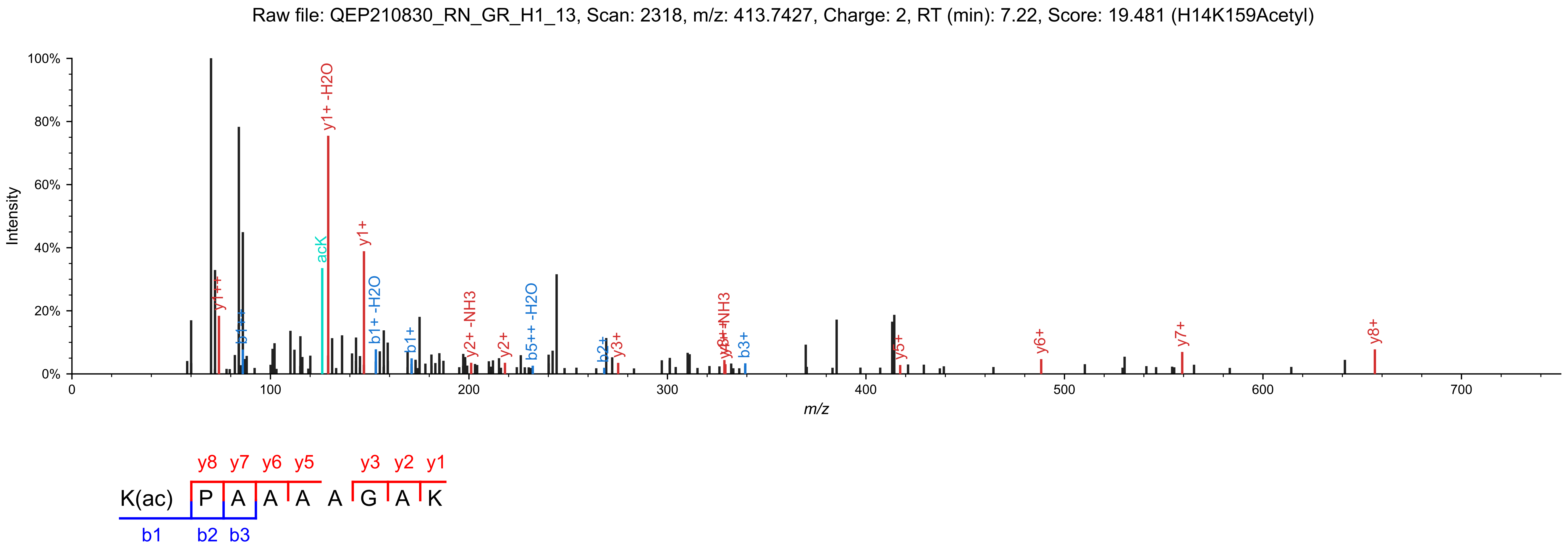

Raw file: QEP210830_RN_GR_H1_13, Scan: 2318, m/z: 413.7427, Charge: 2, RT (min): 7.22, Score: 19.481 (H14K159Acetyl)
Intensity
100%
80%
60%
40%
20%
0%
0
100
200
300
400
500
600
700
m/z
y1++
b1++
acK
y1+ -H2O
y1+
b1+ -H2O
b1+
y2+ -NH3
y2+
b5++ -H2O
b2+
y3+
y8++-NH3
b3+
y5+
y6+
y7+
y8+
y8 y7 y6 y5 y3 y2 y1
K(ac) P A A A A G A K
b1 b2 b3

Raw file: QEP210830_RN_GR_H1_13, Scan: 4519, m/z: 385.7421, Charge: 2, RT (min): 11.69, Score: 19.495 (H10K148Acetyl)

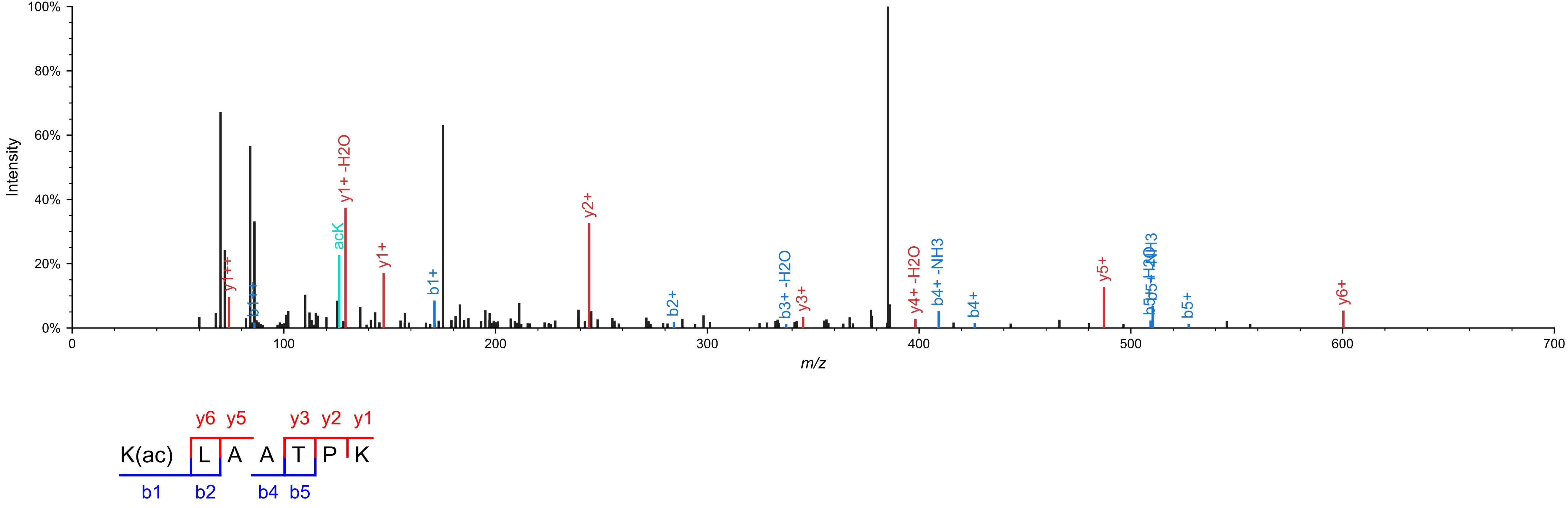

Raw file: QEP210830_RN_GR_H1_13, Scan: 6507, m/z: 672.3437, Charge: 2, RT (min): 15.75, Score: 25.663 (H10K11Acetyl)

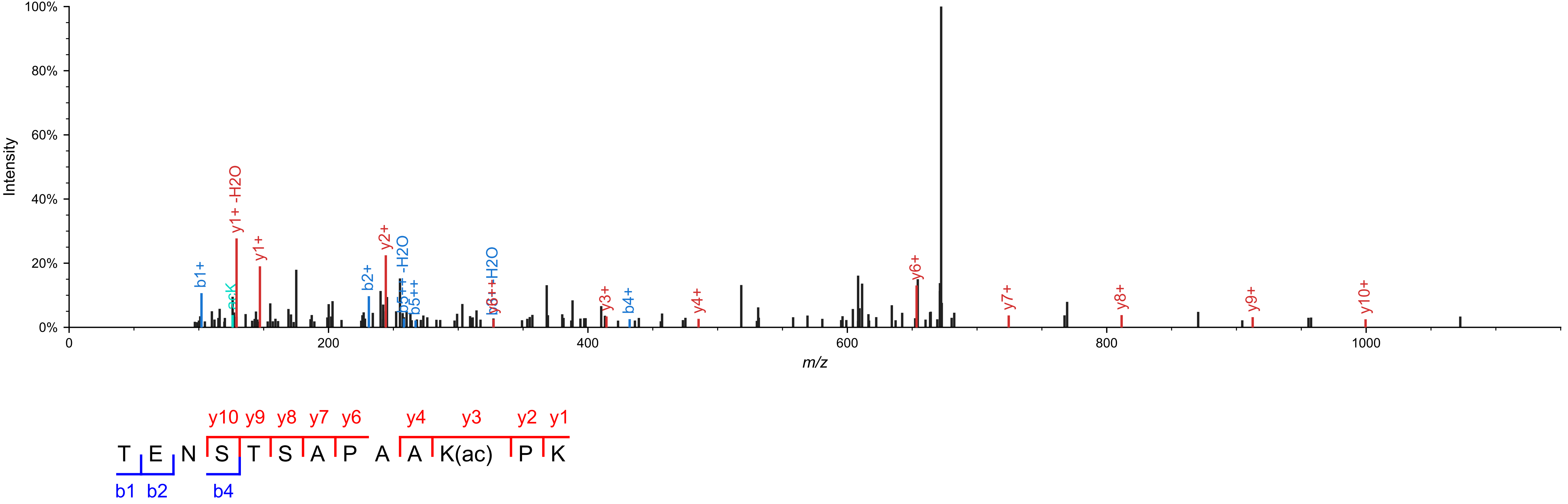

Raw file: QEP210830_RN_GR_H1_13, Scan: 9334, m/z: 532.2617, Charge: 3, RT (min): 21.58, Score: 29.472 (H12K74Methyl)

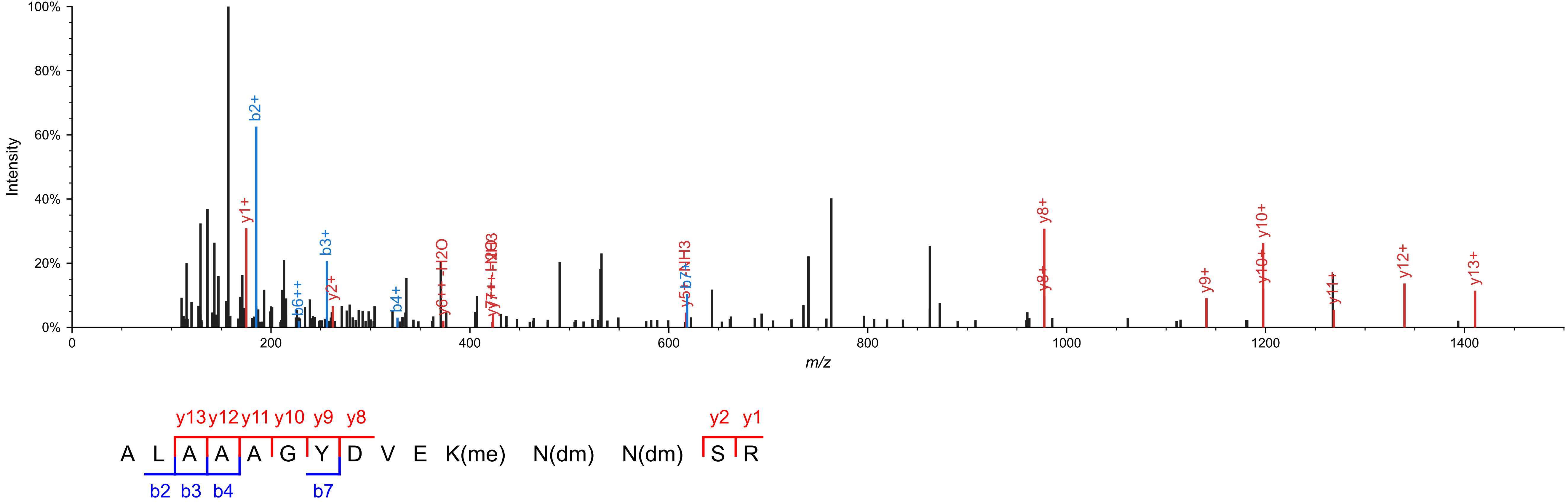

Raw file: QEP210830_RN_GR_H1_13, Scan: 11138, m/z: 686.3411, Charge: 2, RT (min): 25.28, Score: 21.218 (H10K11Formyl)

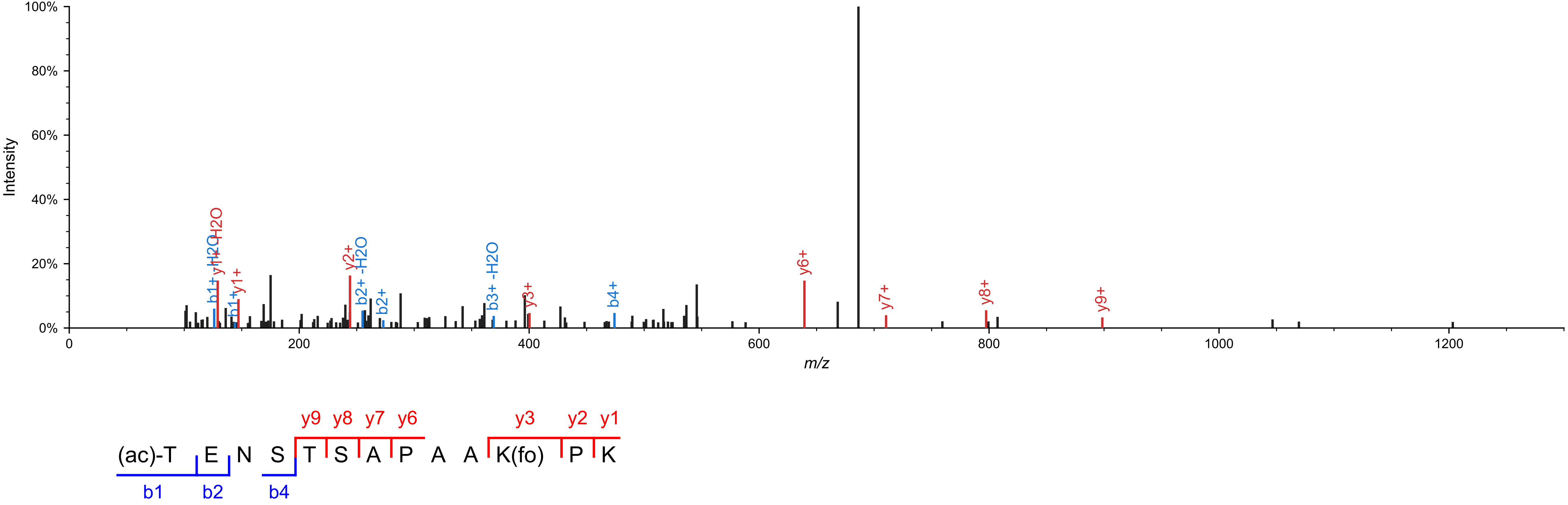

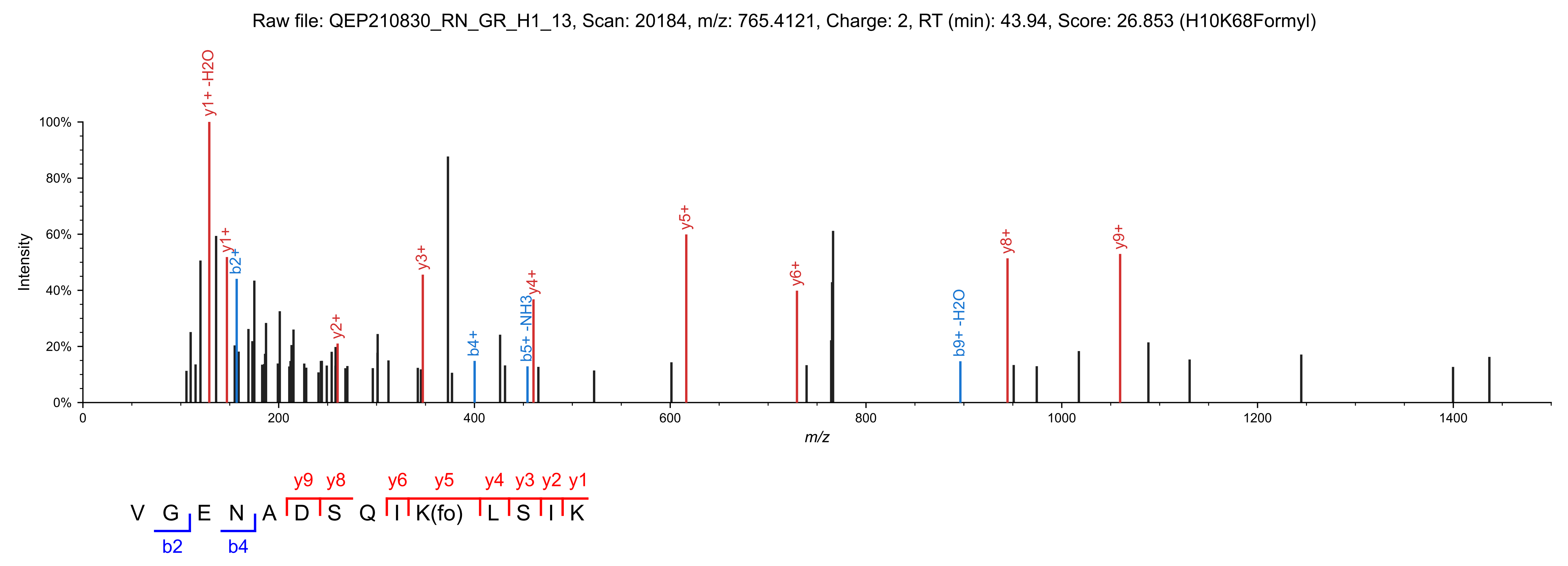
Raw file: QEP210830_RN_GR_H1_13, Scan: 20184, m/z: 765.4121, Charge: 2, RT (min): 43.94, Score: 26.853 (H10K68Formyl)
Intensity
m/z
y1+ -H2O
y1+
b2+
y2+
y3+
b4+
b5+ -NH3
y4+
y5+
y6+
b9+ -H2O
y8+
y9+
V G E N A D S Q I K(fo) L S I K
y9 y8 y6 y5 y4 y3 y2 y1
b2 b4

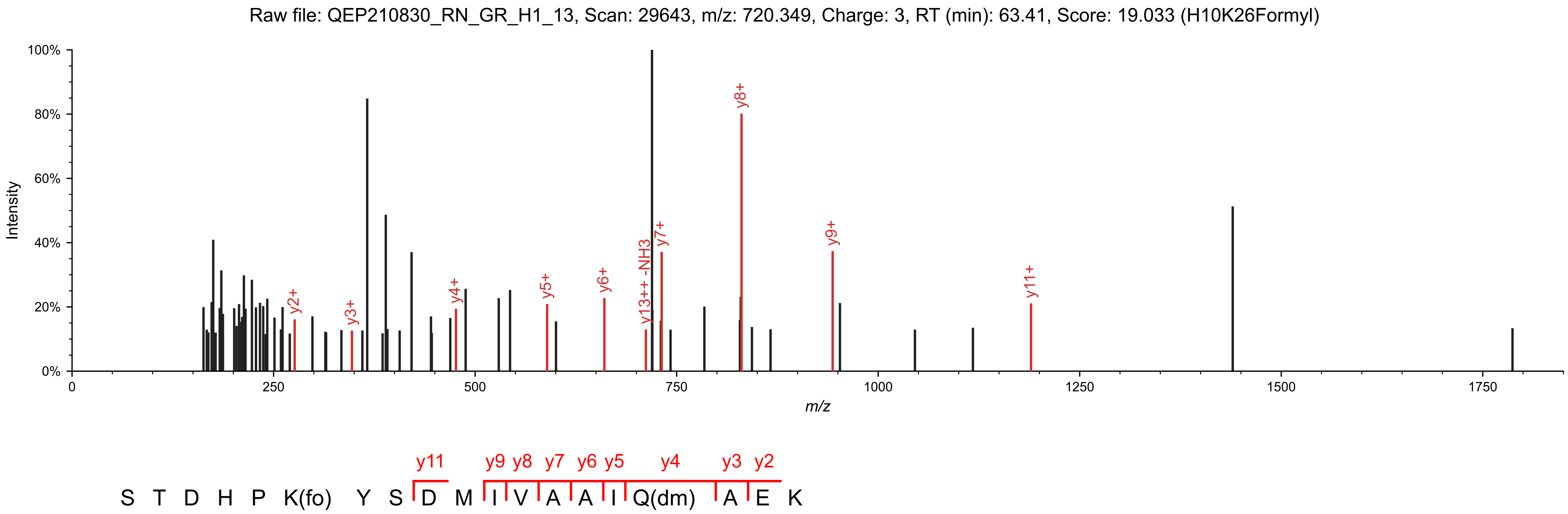

Raw file: QEP210830_RN_GR_H1_13, Scan: 29643, m/z: 720.349, Charge: 3, RT (min): 63.41, Score: 19.033 (H10K26Formyl)
Intensity
100%
80%
60%
40%
20%
0%
0
250
500
750
1000
1250
1500
1750
m/z
y2+
y3+
y4+
y5+
y6+
y13++ -NH3
y7+
y8+
y9+
y11+
y11 y9 y8 y7 y6 y5 y4 y3 y2
S T D H P K(fo) Y S D M I V A A I Q(dm) A E K

Raw file: QEP210830_RN_GR_H1_14, Scan: 19766, m/z: 800.8663, Charge: 2, RT (min): 41.85, Score: 27.994 (H14T3Phosphoryl)

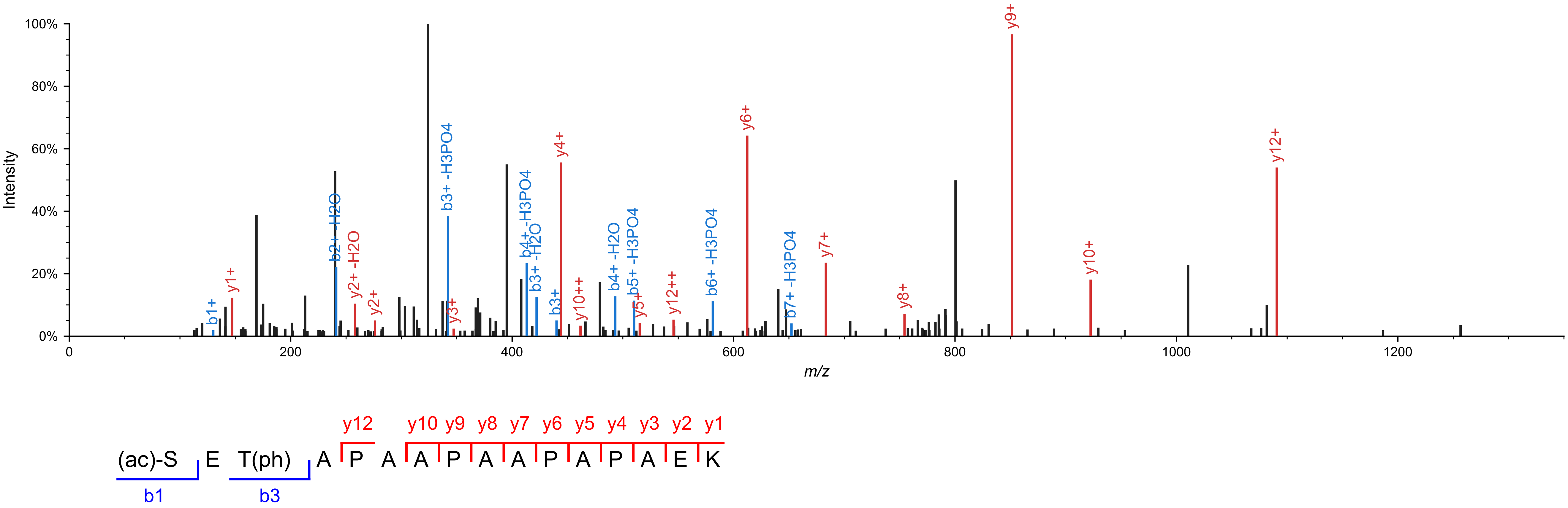

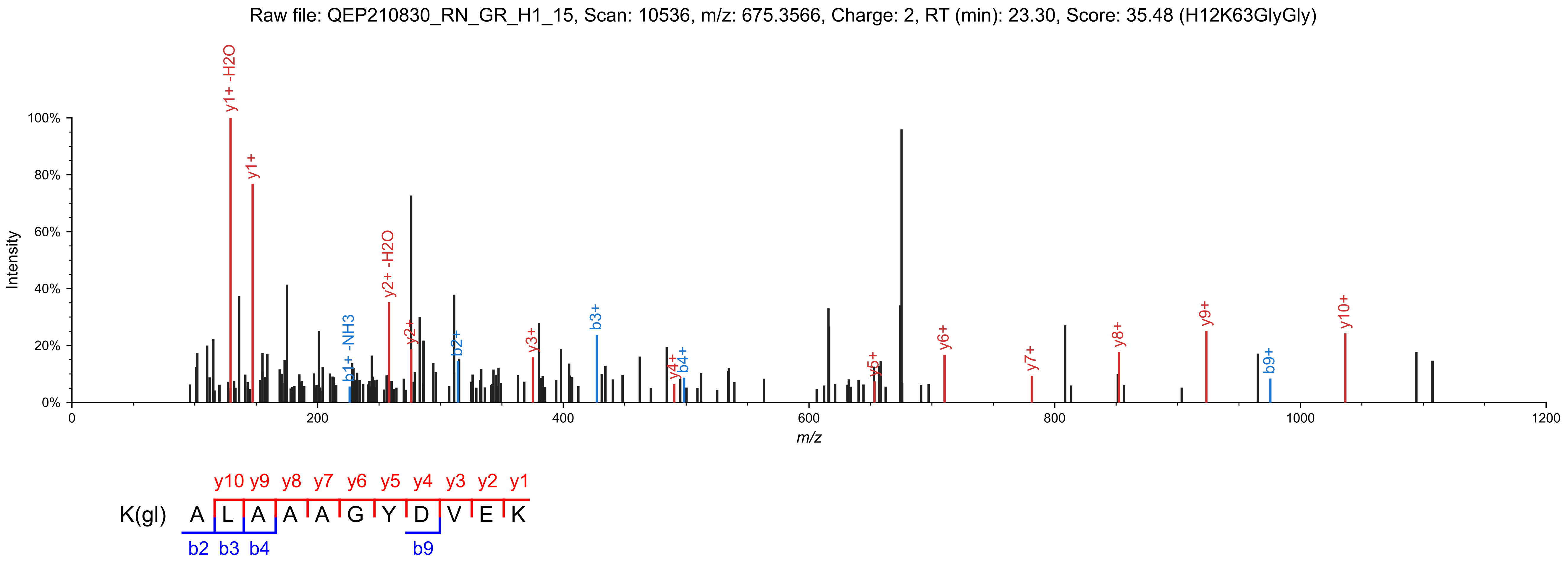

Raw file: QEP210830_RN_GR_H1_15, Scan: 10536, m/z: 675.3566, Charge: 2, RT (min): 23.30, Score: 35.48 (H12K63GlyGly)
Intensity
m/z
y1+ -H2O
y1+
y2+ -H2O
y2+
y3+
y4+
y5+
y6+
y7+
y8+
y9+
y10+
b1+ -NH3
b2+
b3+
b4+
b9+
y10 y9 y8 y7 y6 y5 y4 y3 y2 y1
K(gl) A L A A A G Y D V E K
b2 b3 b4 b9

Raw file: QEP210830_RN_GR_H1_15, Scan: 18984, m/z: 501.3031, Charge: 2, RT (min): 40.02, Score: 23.583 (H12K62Formyl)

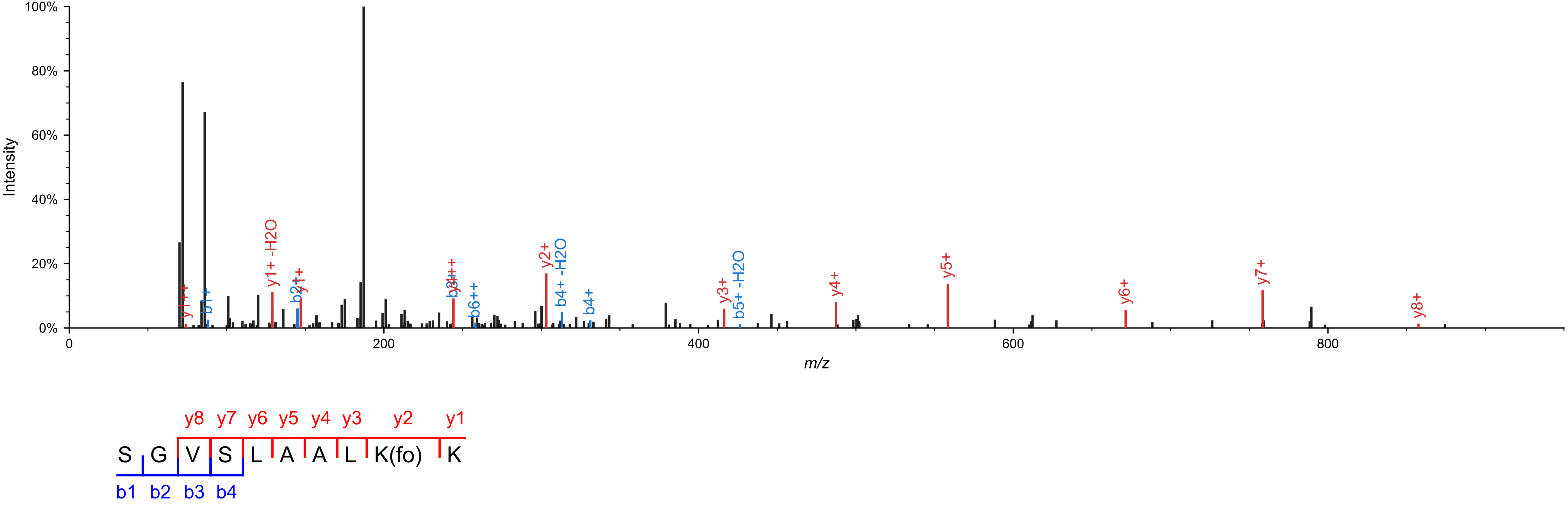

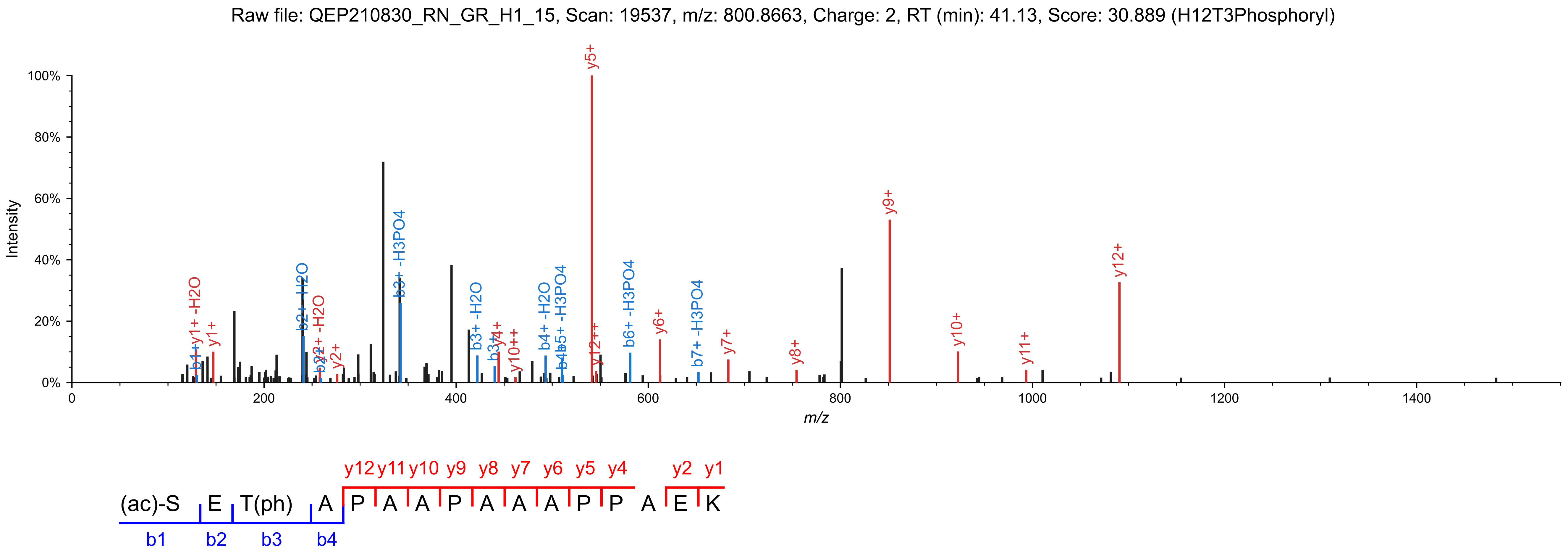

Raw file: QEP210830_RN_GR_H1_15, Scan: 19537, m/z: 800.8663, Charge: 2, RT (min): 41.13, Score: 30.889 (H12T3Phosphoryl)
Intensity
100%
80%
60%
40%
20%
0%
0
200
400
600
800
1000
1200
1400
m/z
y5+
y9+
y12+
b3+ -H3PO4
b2+ -H2O
y1+ -H2O
y1+
b1+
b2+ -H2O
y2+
b3+ -H2O
y4+
y10++
b4+ -H2O
b5+ -H3PO4
y12++
b6+ -H3PO4
y6+
b7+ -H3PO4
y7+
y8+
y10+
y11+
y12 y11 y10 y9 y8 y7 y6 y5 y4 y2 y1
(ac)-S E T(ph) A P A A P A A A P P A E K
b1 b2 b3 b4

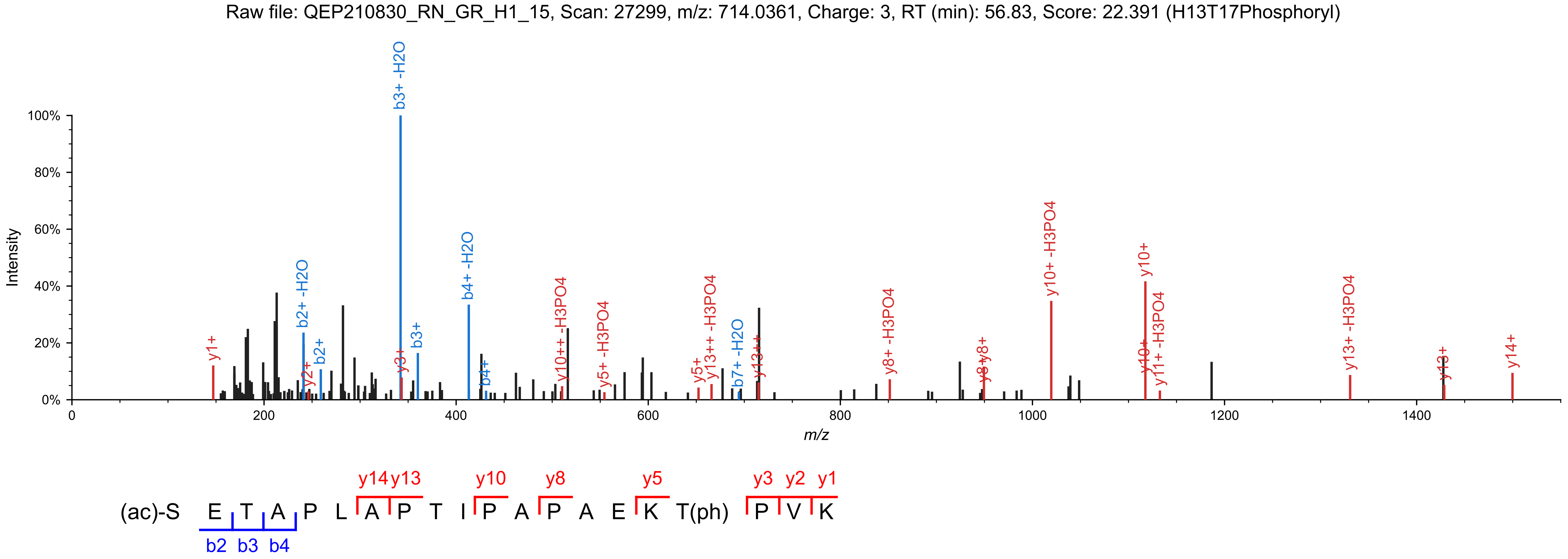
Raw file: QEP210830_RN_GR_H1_15, Scan: 27299, m/z: 714.0361, Charge: 3, RT (min): 56.83, Score: 22.391 (H13T17Phosphoryl)
Intensity
m/z
b3+ -H2O
b4+ -H2O
b2+ -H2O
y10+ -H3PO4
y10+
y13+ -H3PO4
y14+
(ac)-S E T A P L A P T I P A P A E K T(ph) P V K
y14 y13 y10 y8 y5 y3 y2 y1
b2 b3 b4

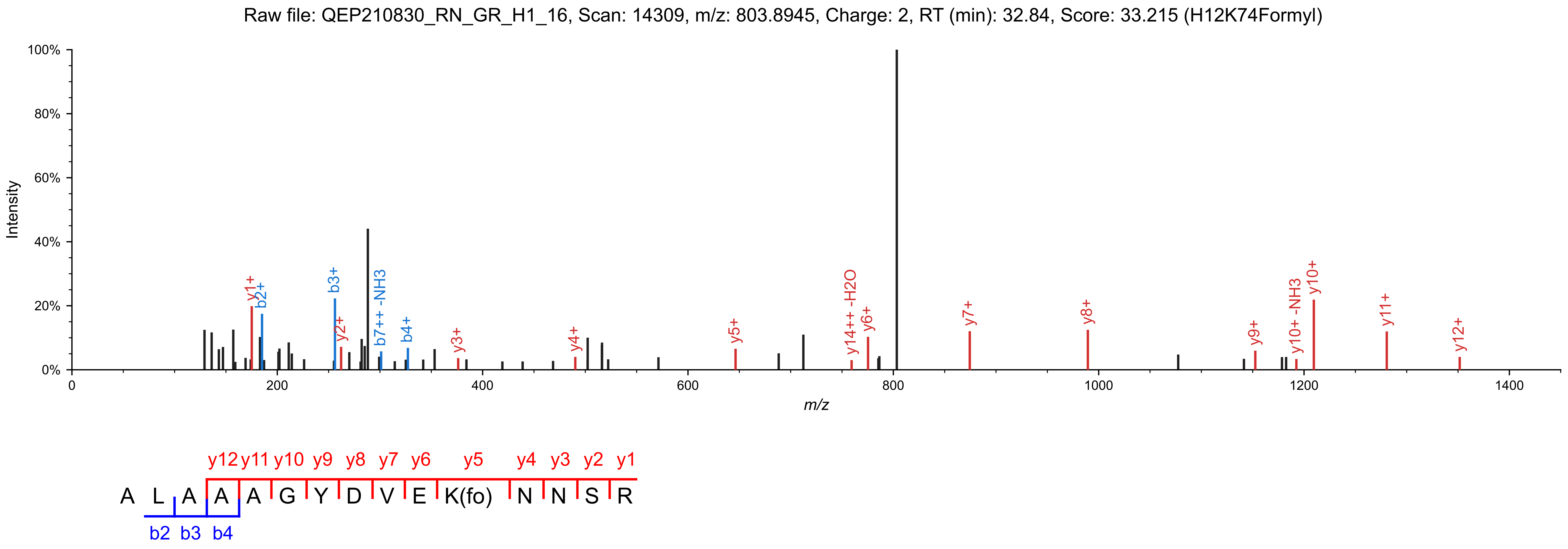

Raw file: QEP210830_RN_GR_H1_16, Scan: 14309, m/z: 803.8945, Charge: 2, RT (min): 32.84, Score: 33.215 (H12K74Formyl)
Intensity
100%
80%
60%
40%
20%
0%
0
200
400
600
800
1000
1200
1400
m/z
y1+
b2+
b3+
y2+
b7++ -NH3
b4+
y3+
y4+
y5+
y14++ -H2O
y6+
y7+
y8+
y9+
y10+ -NH3
y10+
y11+
y12+
y12 y11 y10 y9 y8 y7 y6 y5 y4 y3 y2 y1
A L A A A G Y D V E K(fo) N N S R
b2 b3 b4

Raw file: QEP210830_RN_GR_H1_17, Scan: 9276, m/z: 790.8811, Charge: 2, RT (min): 20.66, Score: 30.919 (H15K77Methyl)

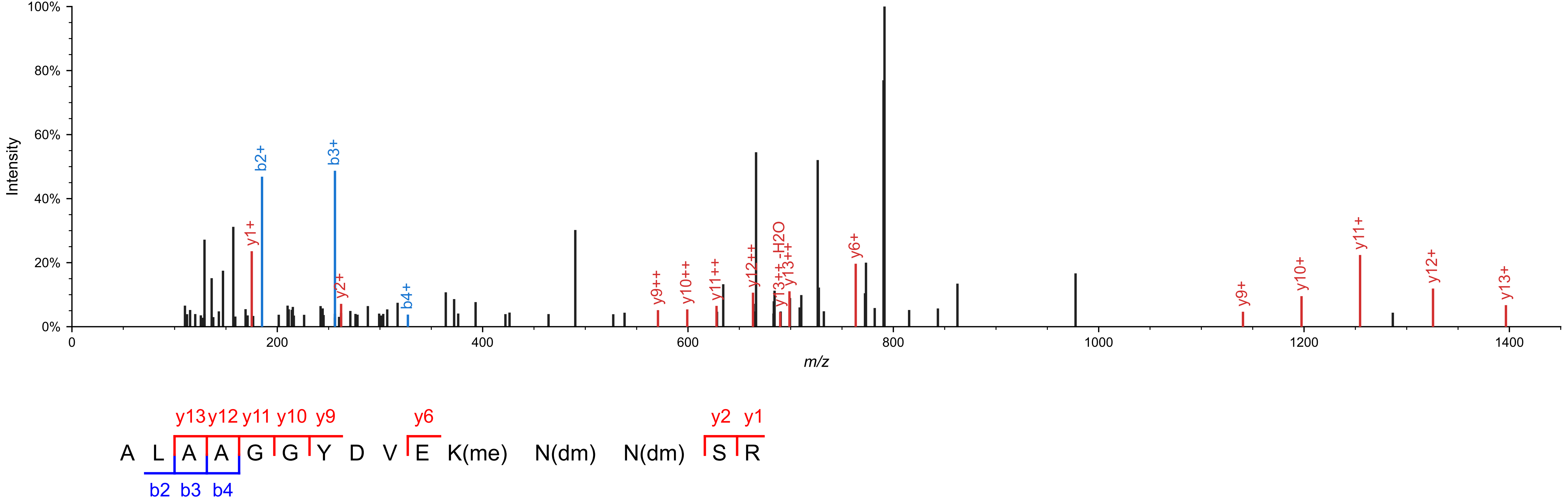

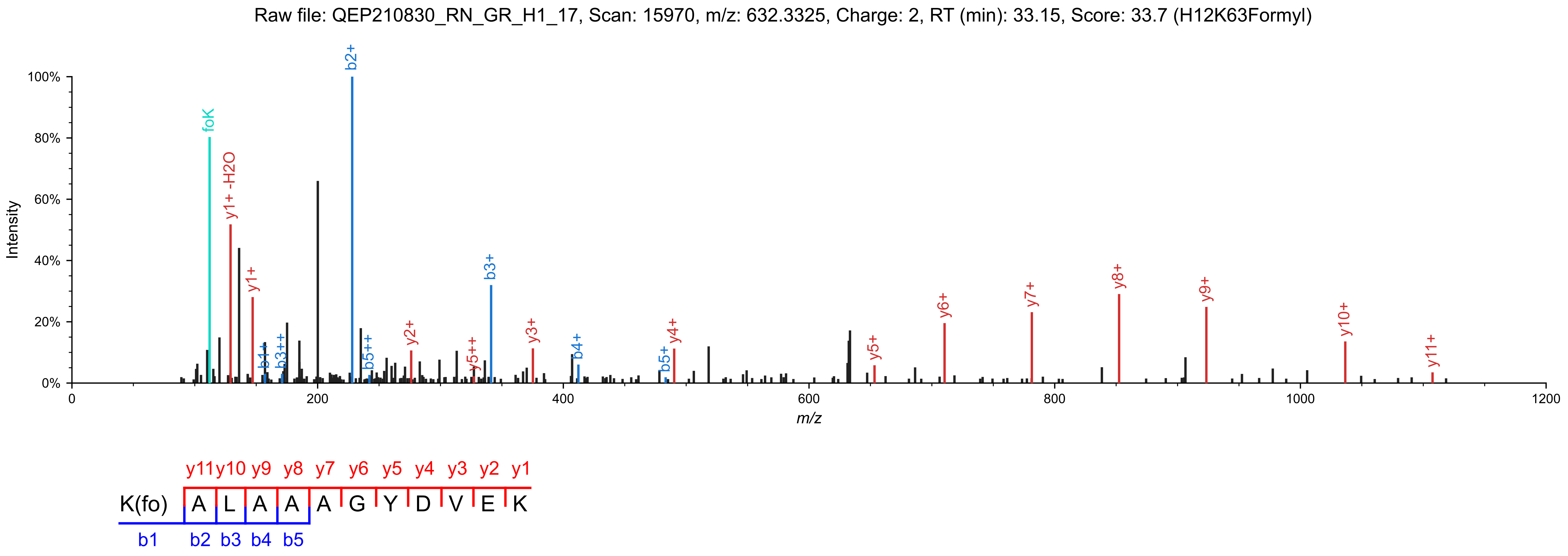
Raw file: QEP210830_RN_GR_H1_17, Scan: 15970, m/z: 632.3325, Charge: 2, RT (min): 33.15, Score: 33.7 (H12K63Formyl)
Intensity
100%
80%
60%
40%
20%
0%
0
200
400
600
800
1000
1200
m/z
foK
y1+ -H2O
y1+
b1+
b3++
b2+
b5++
y2+
y5++
b3+
y3+
b4+
b5+
y4+
y5+
y6+
y7+
y8+
y9+
y10+
y11+
y11 y10 y9 y8 y7 y6 y5 y4 y3 y2 y1
K(fo) A L A A A G Y D V E K
b1 b2 b3 b4 b5

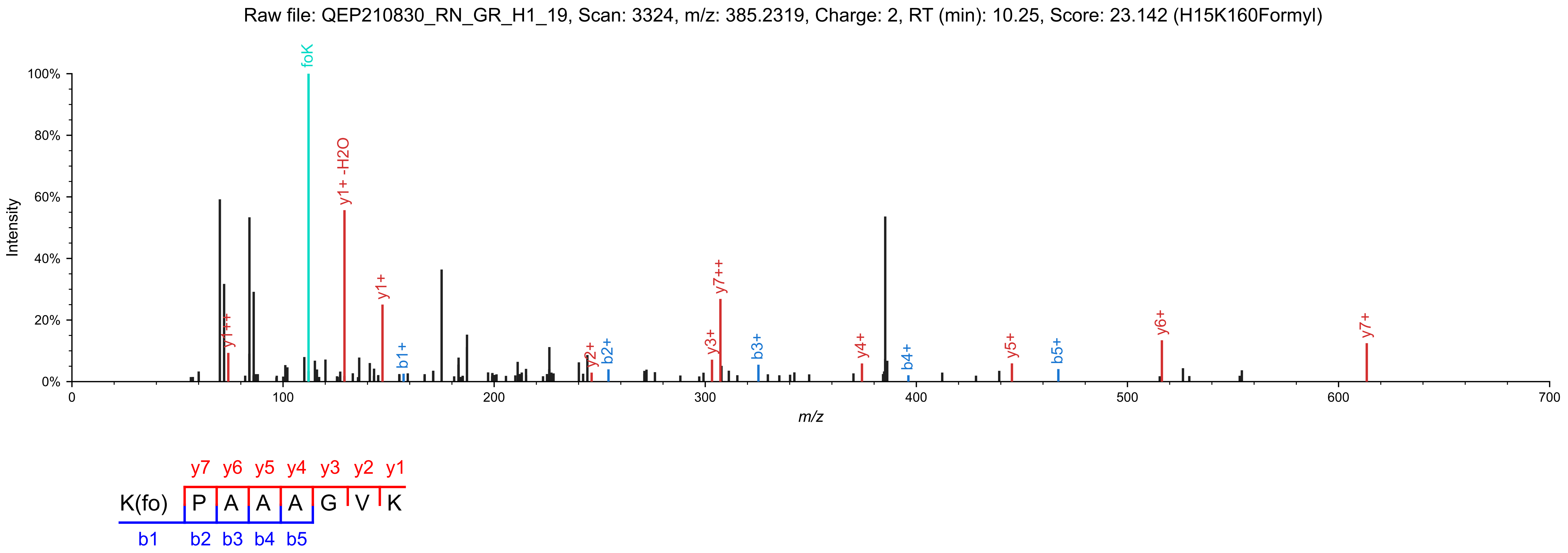

Raw file: QEP210830_RN_GR_H1_19, Scan: 3324, m/z: 385.2319, Charge: 2, RT (min): 10.25, Score: 23.142 (H15K160Formyl)
Intensity
m/z
foK
y1+ -H2O
y1++
y1+
b1+
y2+
b2+
y3+
y7++
b3+
y4+
b4+
y5+
b5+
y6+
y7+
y7 y6 y5 y4 y3 y2 y1
K(fo) P A A A G V K
b1 b2 b3 b4 b5

Raw file: QEP210830_RN_GR_H1_19, Scan: 18215, m/z: 677.8824, Charge: 2, RT (min): 43.51, Score: 30.339 (H12K33Formyl)

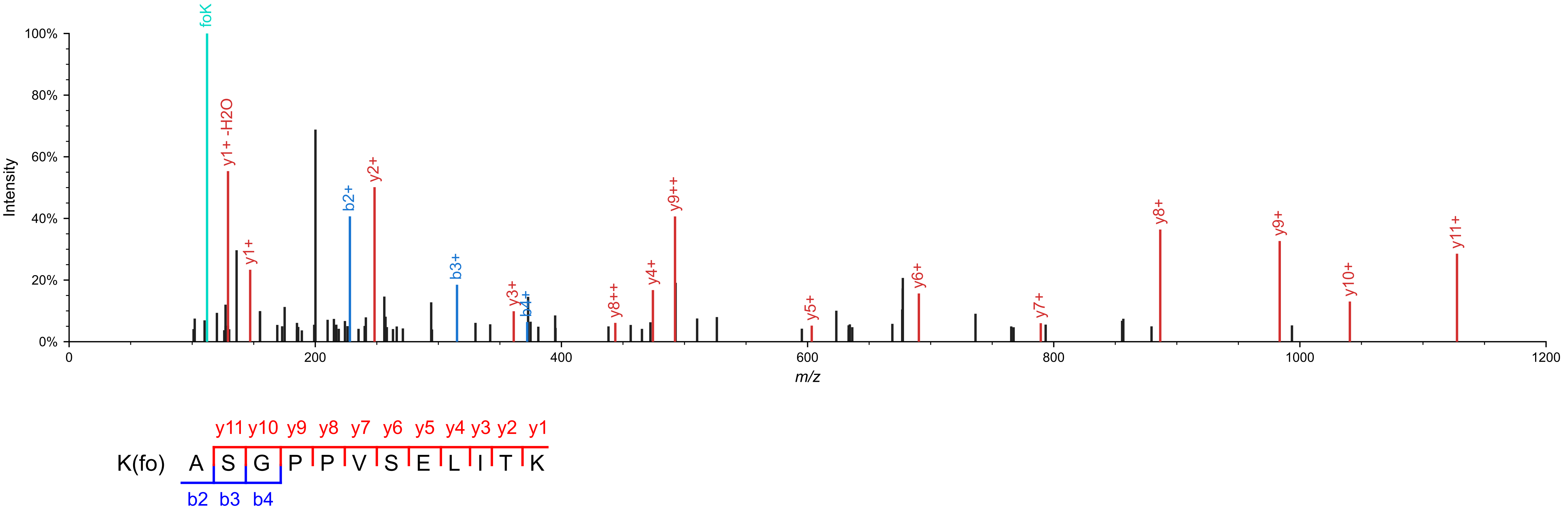

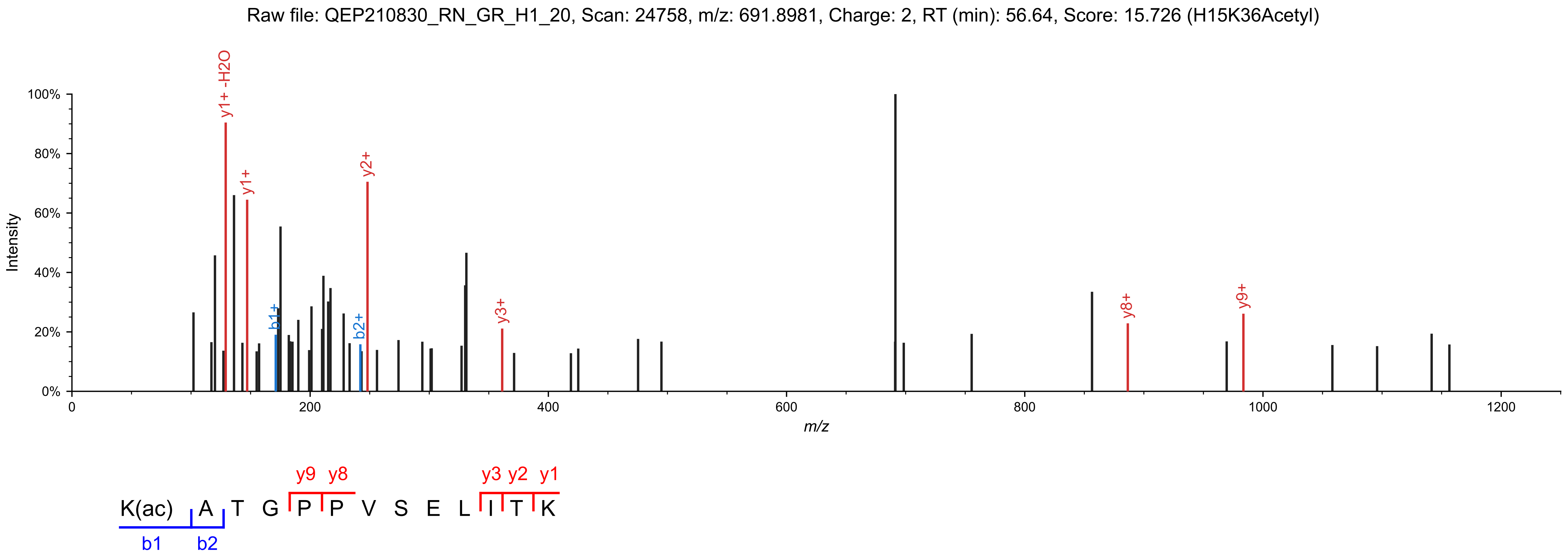
Raw file: QEP210830_RN_GR_H1_20, Scan: 24758, m/z: 691.8981, Charge: 2, RT (min): 56.64, Score: 15.726 (H15K36Acetyl)
Intensity
100%
80%
60%
40%
20%
0%
0
200
400
600
800
1000
1200
m/z
y1+ -H2O
y1+
b1+
b2+
y2+
y3+
y8+
y9+
y9 y8 y3 y2 y1
K(ac) A T G P P V S E L I T K
b1 b2

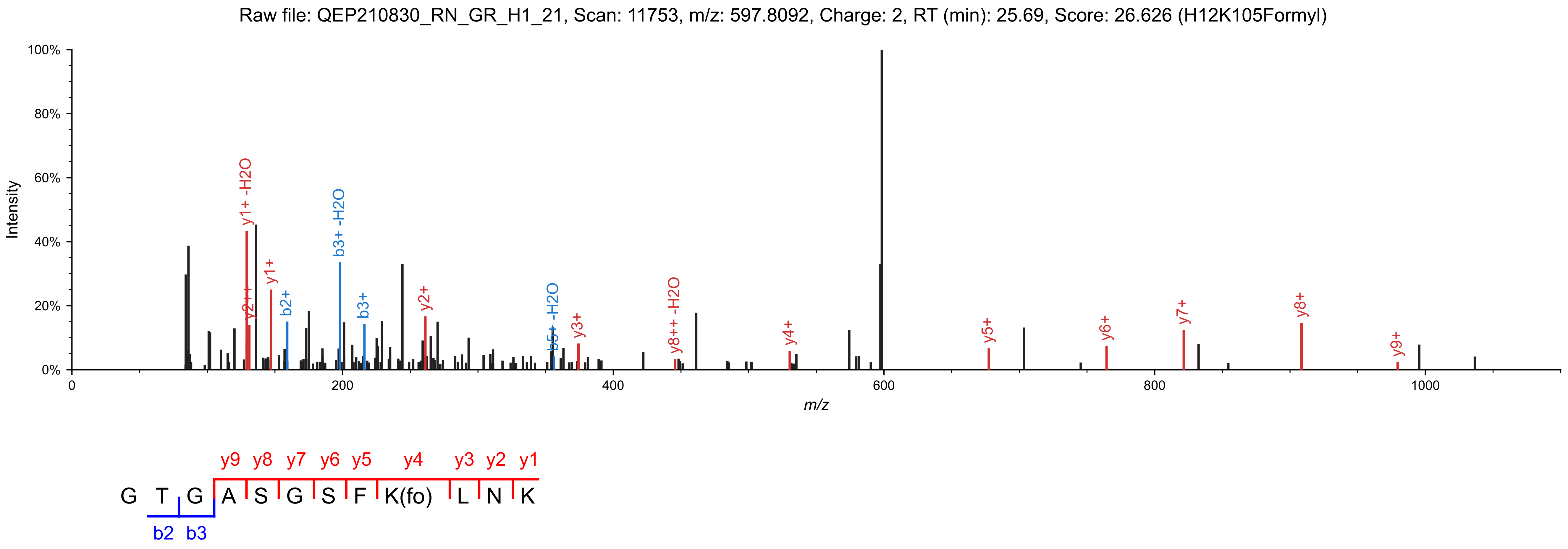
Raw file: QEP210830_RN_GR_H1_21, Scan: 11753, m/z: 597.8092, Charge: 2, RT (min): 25.69, Score: 26.626 (H12K105Formyl)
Intensity
100%
80%
60%
40%
20%
0%
0
200
400
600
800
1000
m/z
y1+ -H2O
y2++
y1+
b2+
b3+ -H2O
b3+
y2+
b5+ -H2O
y3+
y8++ -H2O
y4+
y5+
y6+
y7+
y8+
y9+
y9 y8 y7 y6 y5 y4 y3 y2 y1
G T G A S G S F K(fo) L N K
b2 b3

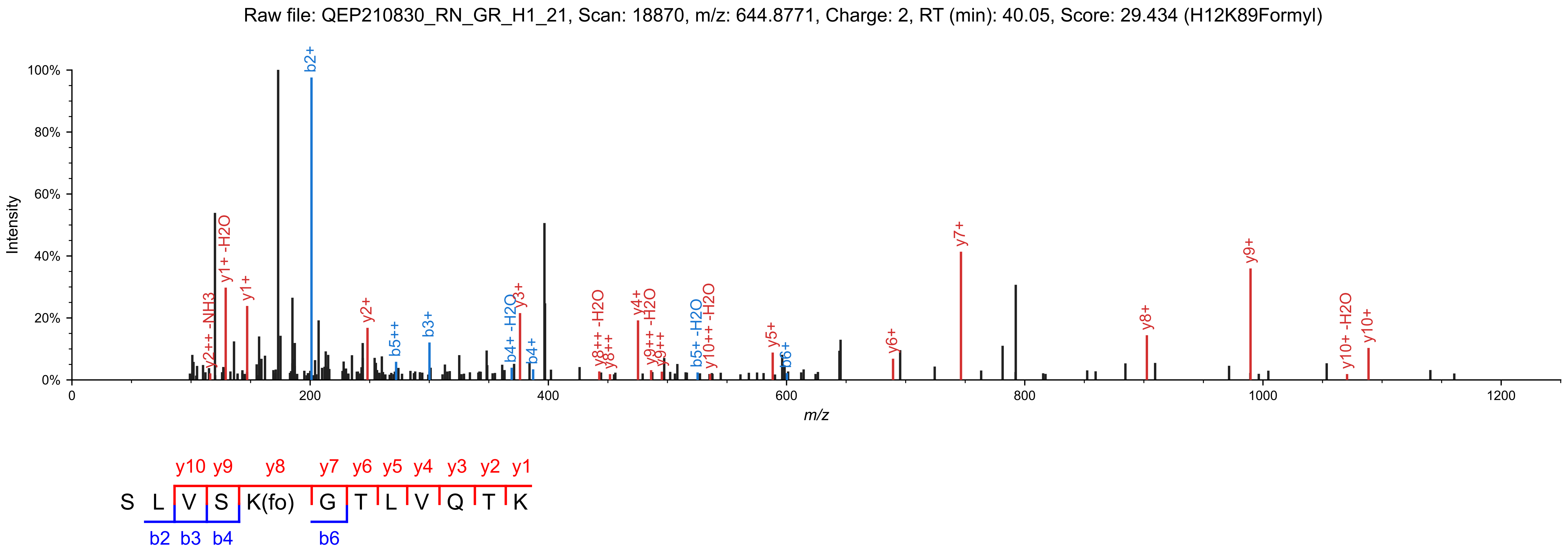
Raw file: QEP210830_RN_GR_H1_21, Scan: 18870, m/z: 644.8771, Charge: 2, RT (min): 40.05, Score: 29.434 (H12K89Formyl)
Intensity
m/z
b2+
y1+ -H2O
y1+
y2++ -NH3
y2+
b5++
b3+
b4+ -H2O
y3+
b4+
y8++ -H2O
y8++
y4+ -H2O
y9++ -H2O
y9++
b5+ -H2O
y10++ -H2O
y5+
b6+
y6+
y7+
y8+
y9+
y10+ -H2O
y10+
y10 y9 y8 y7 y6 y5 y4 y3 y2 y1
S L V S K(fo) G T L V Q T K
b2 b3 b4 b6

Raw file: QEP210830_RN_GR_H1_22, Scan: 4051, m/z: 442.7636, Charge: 2, RT (min): 10.59, Score: 20.387 (H13K140Acetyl)

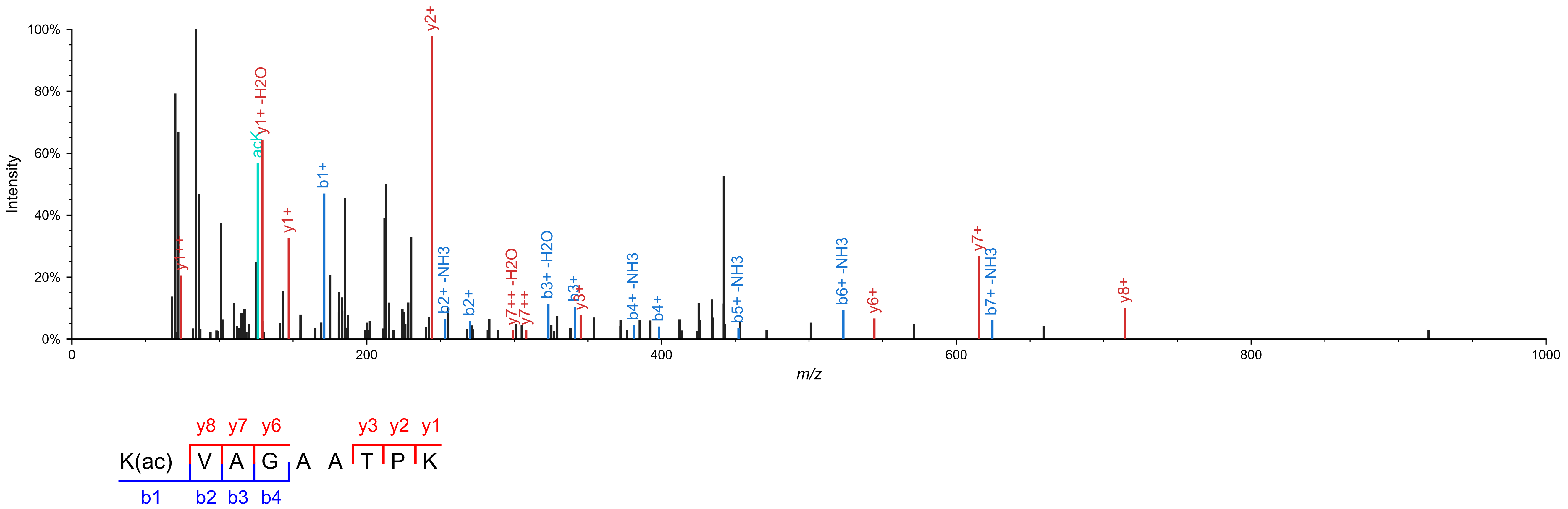

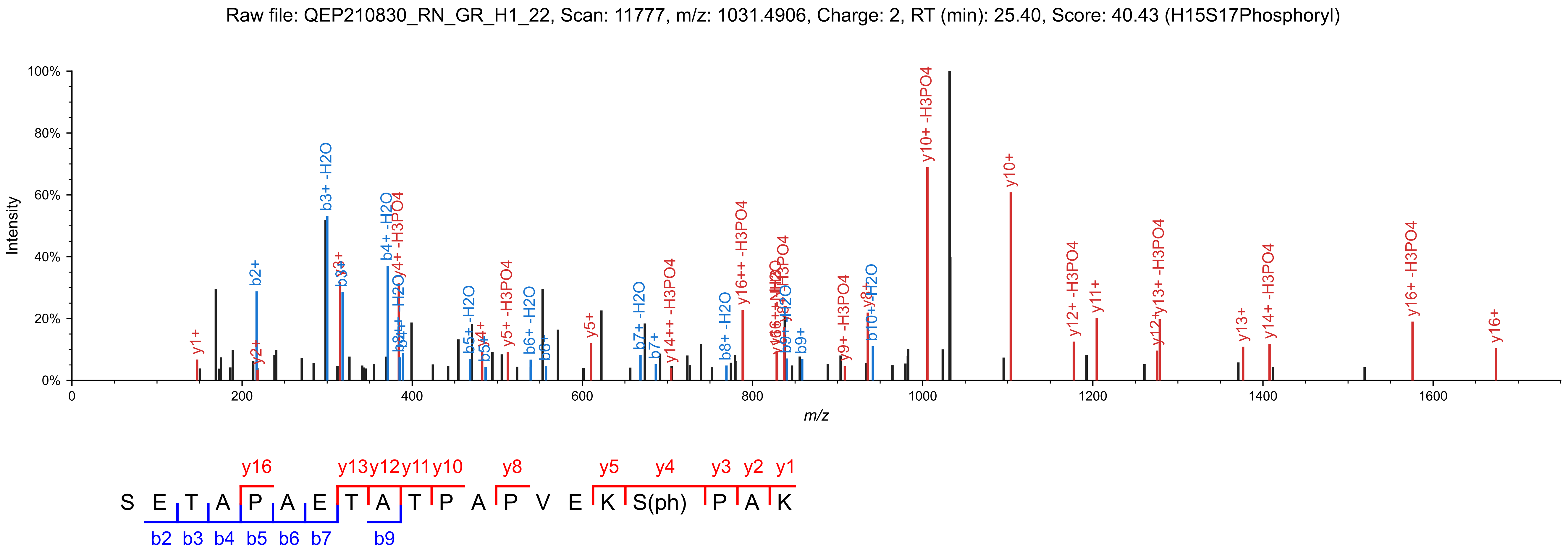

Raw file: QEP210830_RN_GR_H1_22, Scan: 11777, m/z: 1031.4906, Charge: 2, RT (min): 25.40, Score: 40.43 (H15S17Phosphoryl)
Intensity
m/z
y10+ -H3PO4
y10+
b3+ -H2O
b4+ -H2O
y4+ -H3PO4
y16++ -H3PO4
y12+ -H3PO4
y13+ -H3PO4
y14+ -H3PO4
y16+ -H3PO4
y11+
y13+
y16+
y1+
y2+
b2+
y5+
y5+ -H3PO4
y9+ -H3PO4
y14++ -H3PO4
b8+ -H2O
b7+ -H2O
b6+ -H2O
b5+ -H2O
b10+ -H2O
b7+
b9+
S E T A P A E T A T P A P V E K S(ph) P A K
y16 y13 y12 y11 y10 y8 y5 y4 y3 y2 y1
b2 b3 b4 b5 b6 b7 b9

Raw file: QEP210830_RN_GR_H1_22, Scan: 12331, m/z: 687.9012, Charge: 2, RT (min): 26.45, Score: 26.137 (H12K89GlyGly)

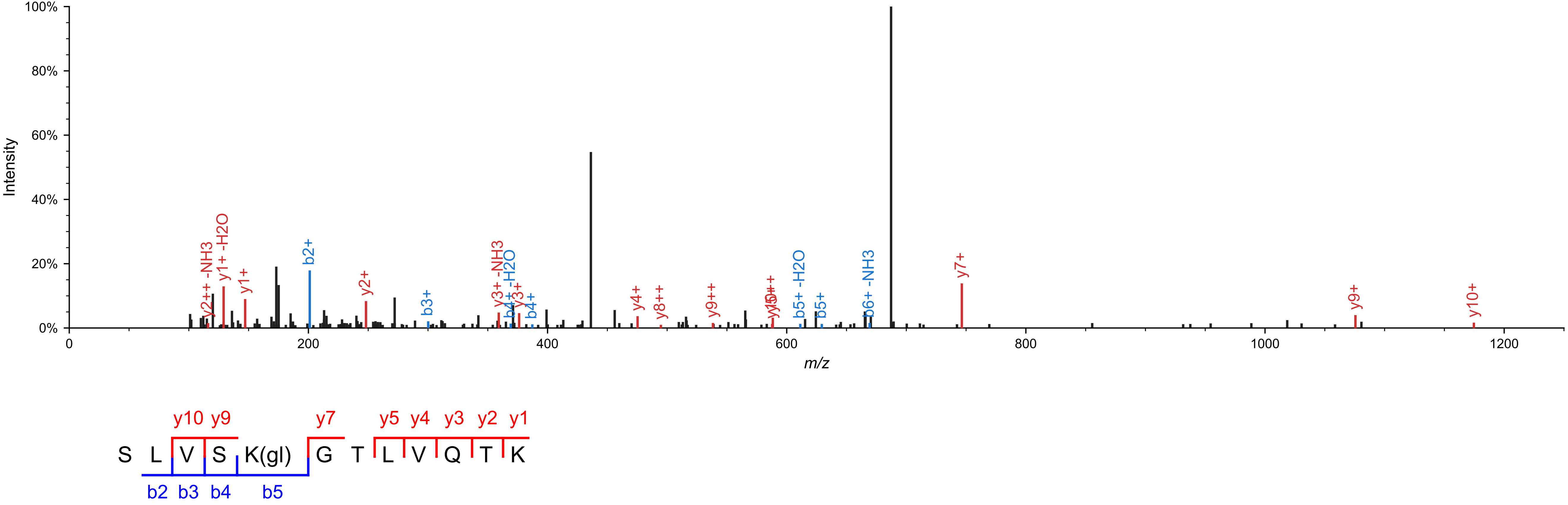

Raw file: QEP210830_RN_GR_H1_22, Scan: 12613, m/z: 647.3378, Charge: 2, RT (min): 26.98, Score: 40.067 (H12K63Carboxymethyl)

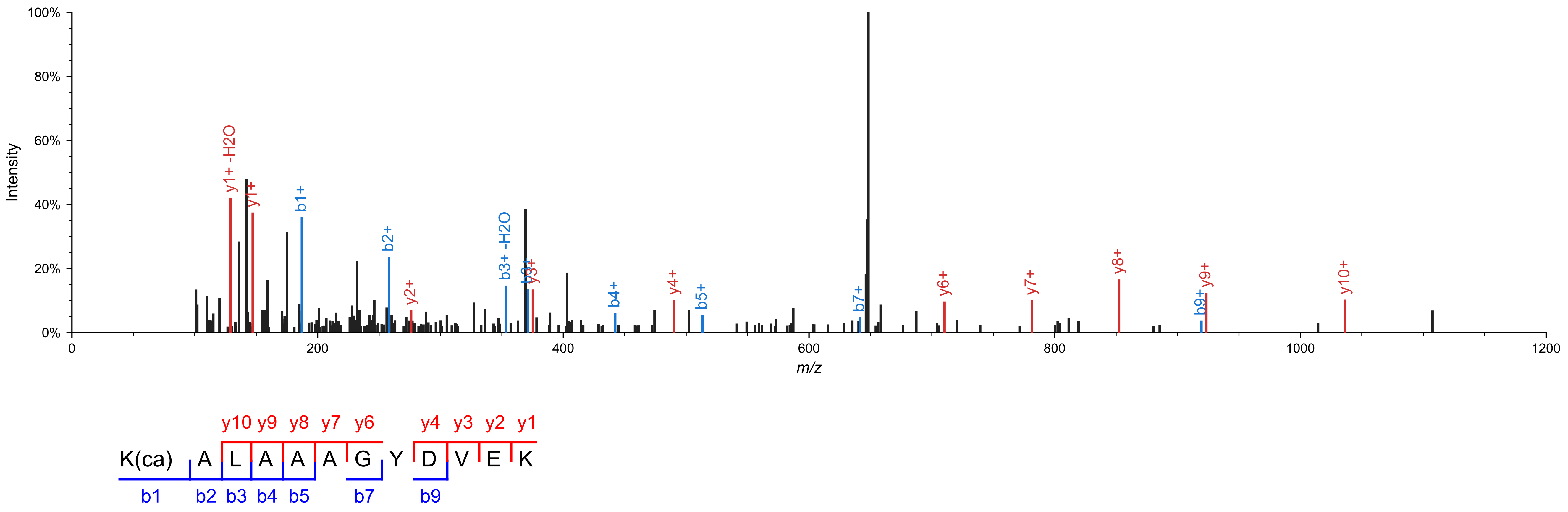

Raw file: QEP210830_RN_GR_H1_22, Scan: 12753, m/z: 532.9457, Charge: 3, RT (min): 27.24, Score: 28.752 (H12K96Carboxymethyl)

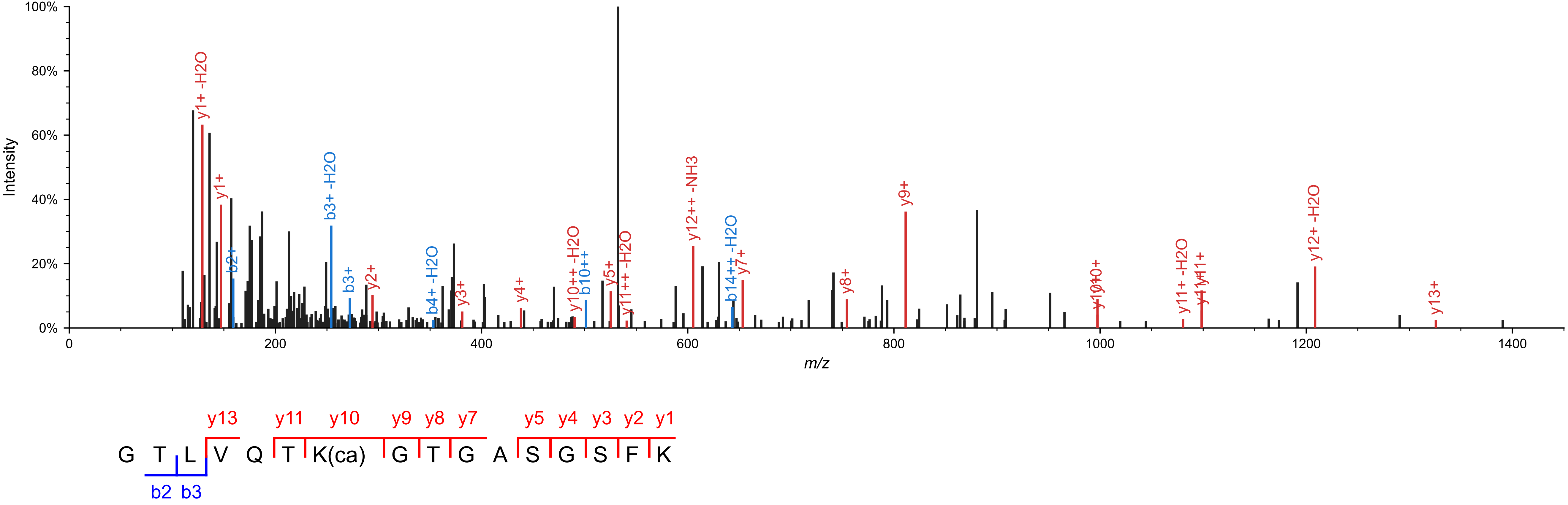

Raw file: QEP210830_RN_GR_H1_22, Scan: 12991, m/z: 632.3326, Charge: 2, RT (min): 27.70, Score: 38.007 (H15K66Acetyl)

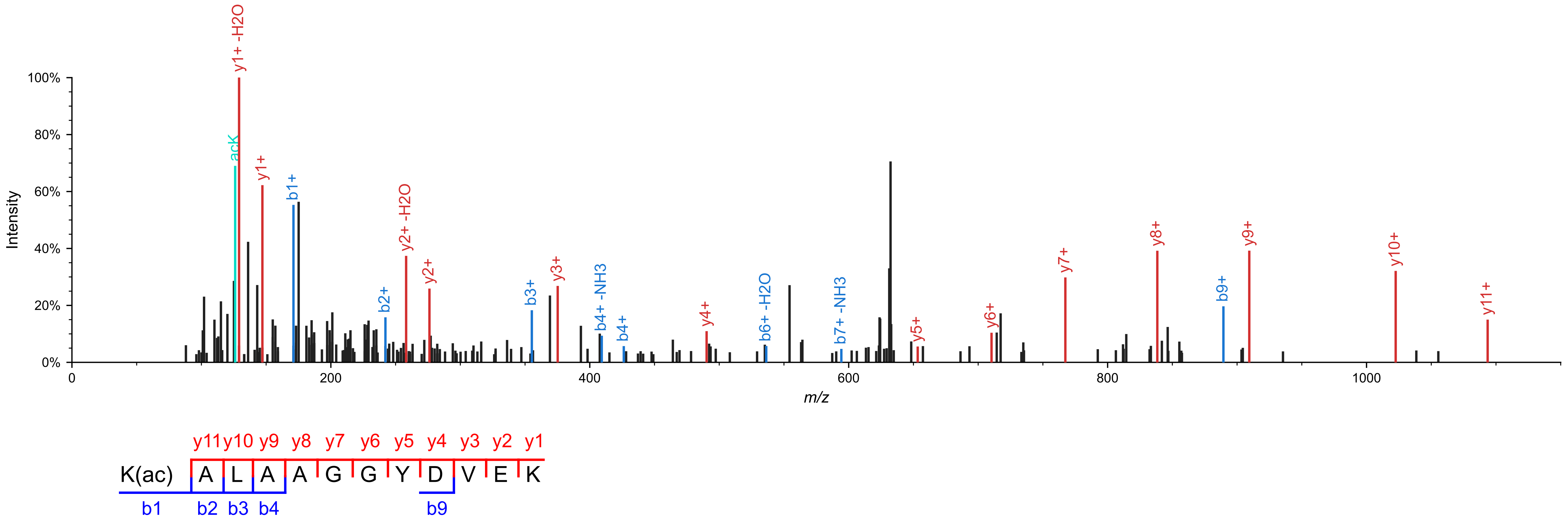

Raw file: QEP210830_RN_GR_H1_22, Scan: 13490, m/z: 611.3091, Charge: 2, RT (min): 28.64, Score: 26.996 (H12K74GlyGly)

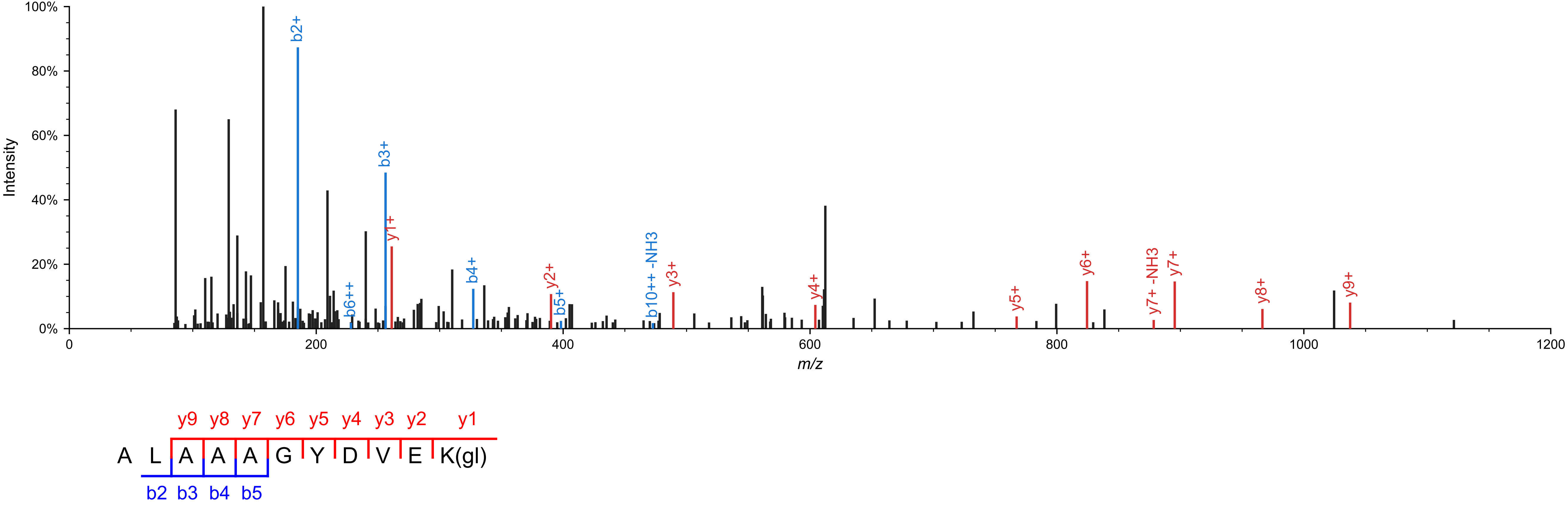

Raw file: QEP210830_RN_GR_H1_22, Scan: 13530, m/z: 611.3035, Charge: 2, RT (min): 28.71, Score: 26.728 (H12K74Glutaryl)

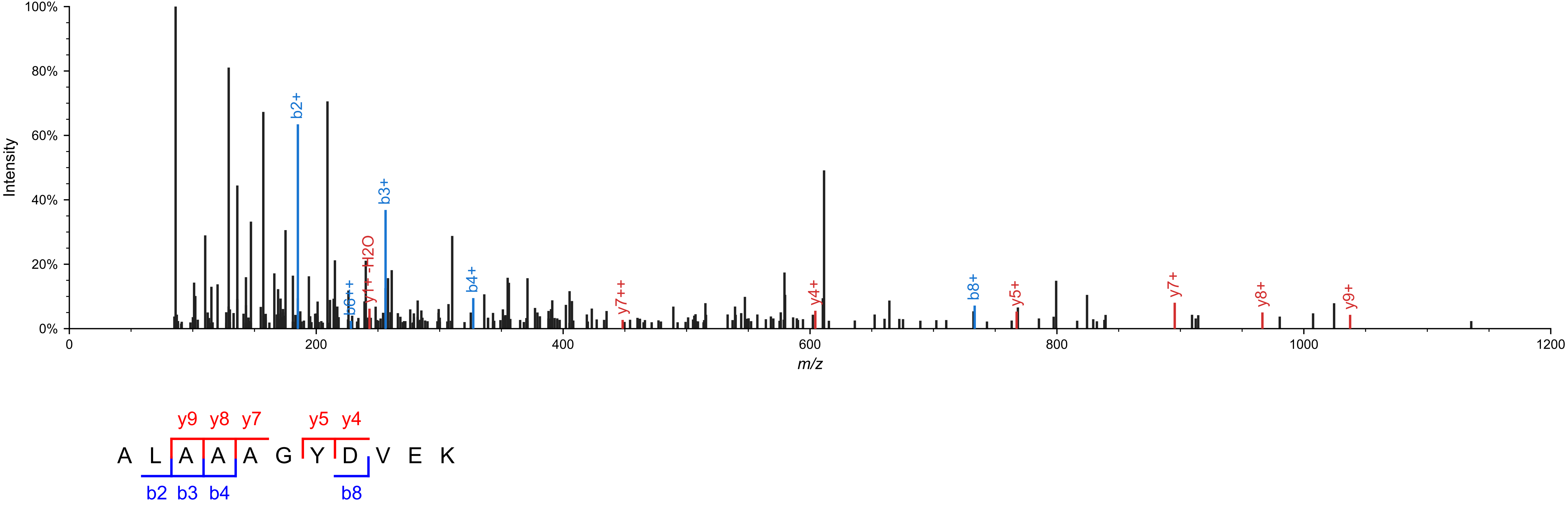

Raw file: QEP210830_RN_GR_H1_22, Scan: 14432, m/z: 659.8825, Charge: 2, RT (min): 30.50, Score: 31.252 (H12K89Carboxymethyl)

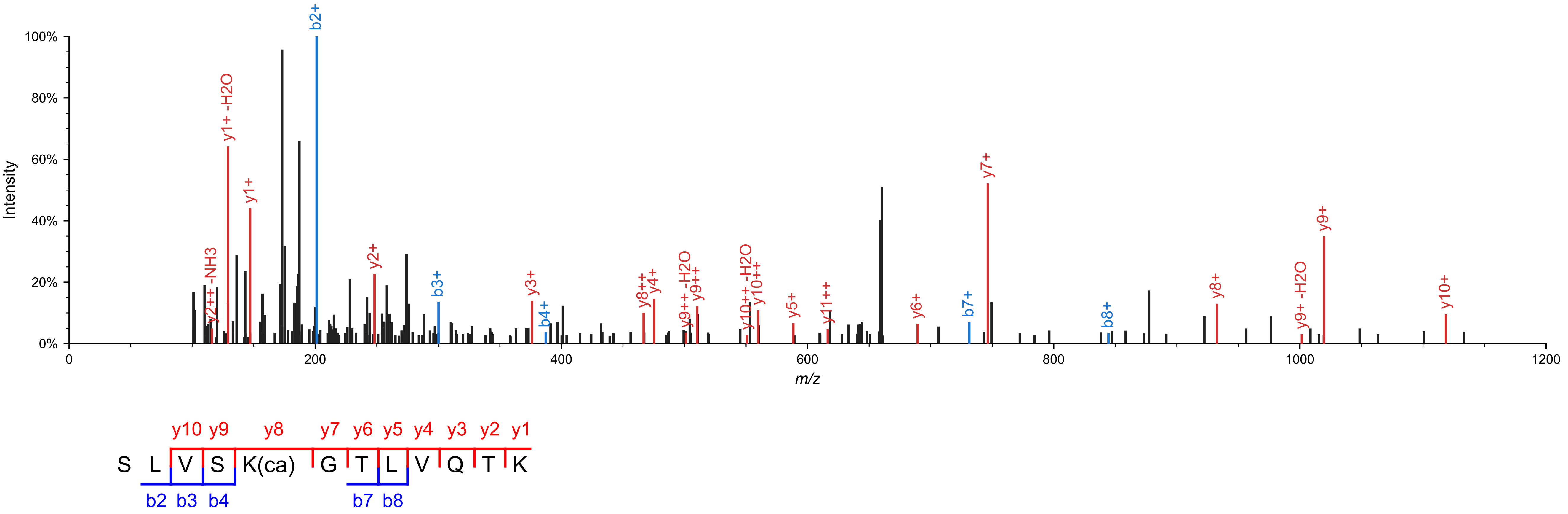

Raw file: QEP210830_RN_GR_H1_22, Scan: 18257, m/z: 692.8877, Charge: 2, RT (min): 37.90, Score: 35.3 (H12K33Carboxymethyl)

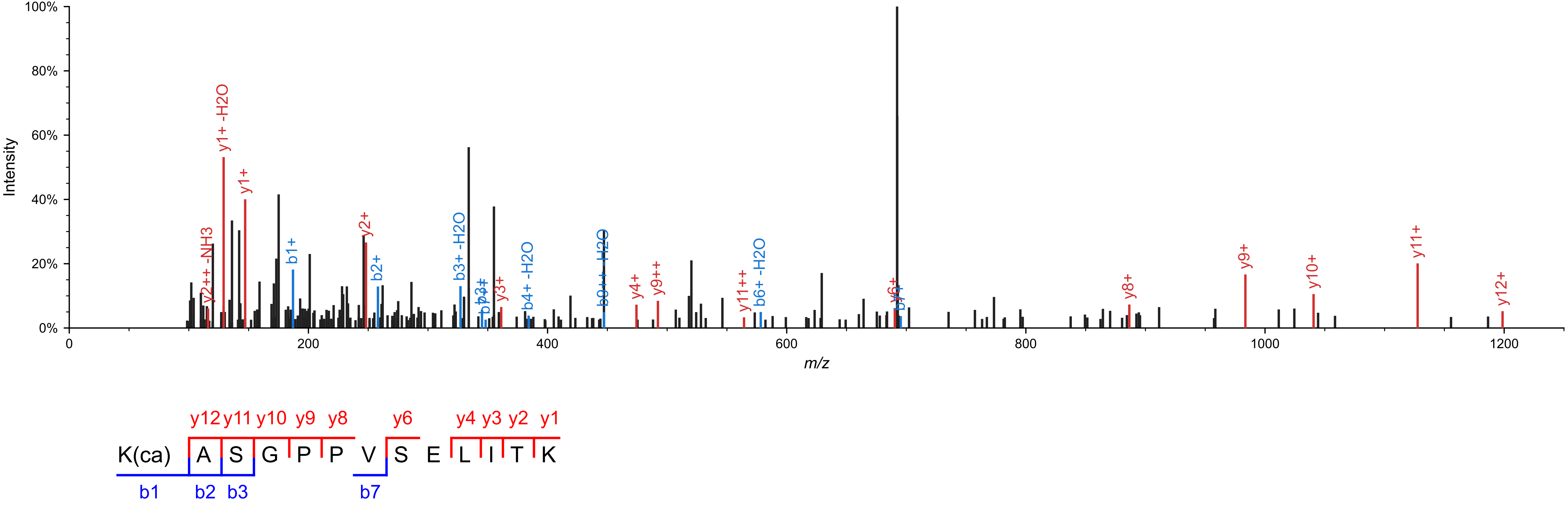

Raw file: QEP210830_RN_GR_H1_22, Scan: 20503, m/z: 1026.5102, Charge: 2, RT (min): 42.30, Score: 35.213 (H15K16Formyl)

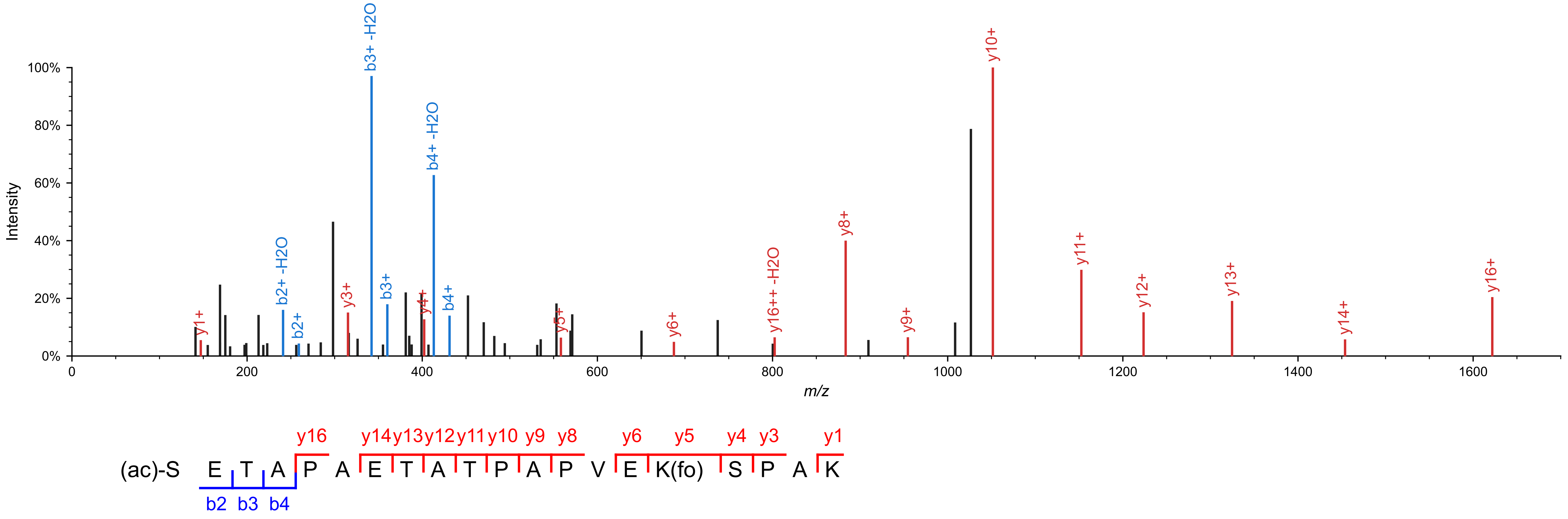

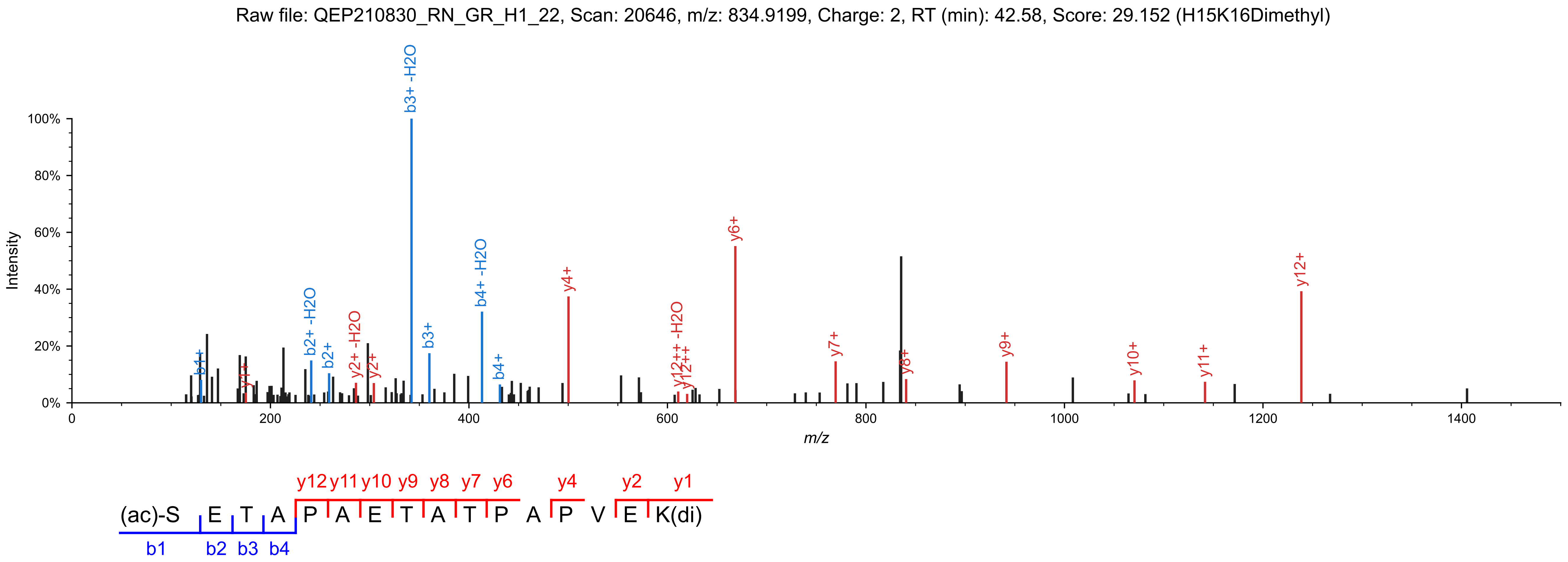

Raw file: QEP210830_RN_GR_H1_22, Scan: 20646, m/z: 834.9199, Charge: 2, RT (min): 42.58, Score: 29.152 (H15K16Dimethyl)
Intensity
m/z
b3+ -H2O
b4+ -H2O
y6+
y12+
y4+
b2+ -H2O
y2+ -H2O
b3+
b2+
y2+
b1+
y1+
b4+
y12++ -H2O
y12++
y7+
y8+
y9+
y10+
y11+
y12 y11 y10 y9 y8 y7 y6 y4 y2 y1
(ac)-S E T A P A E T A T P A P V E K(di)
b1 b2 b3 b4

Raw file: QEP210830_RN_GR_H1_22, Scan: 21562, m/z: 860.8875, Charge: 2, RT (min): 44.39, Score: 36.092 (H15T3Phosphoryl)

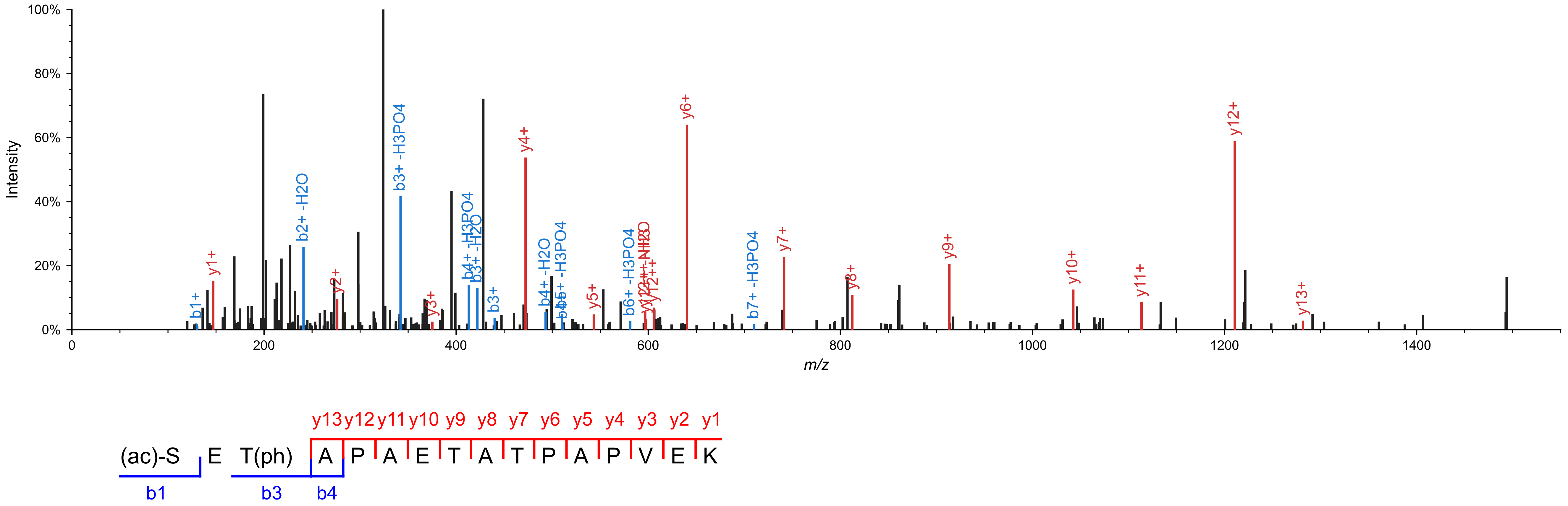

Raw file: QEP210830_RN_GR_H1_22, Scan: 22292, m/z: 521.8164, Charge: 2, RT (min): 45.85, Score: 20.538 (H15K65Formyl)

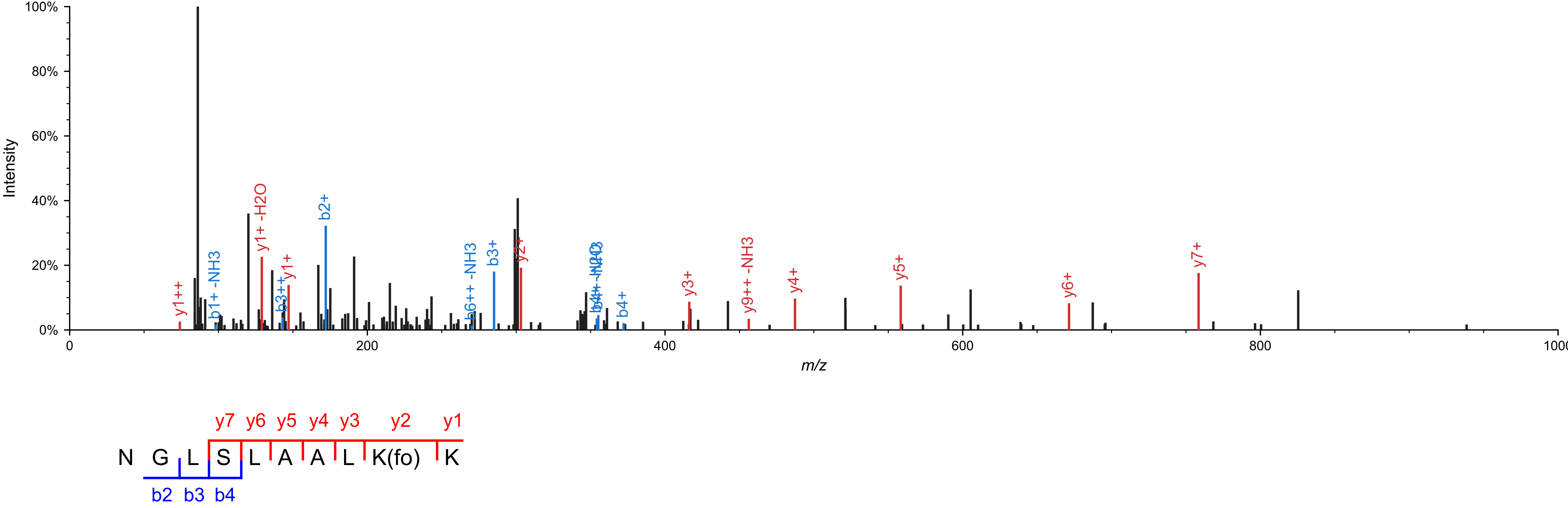

Raw file: QEP210830_RN_GR_H1_26, Scan: 17968, m/z: 684.8903, Charge: 2, RT (min): 37.35, Score: 42.922 (H12K33Acetyl)

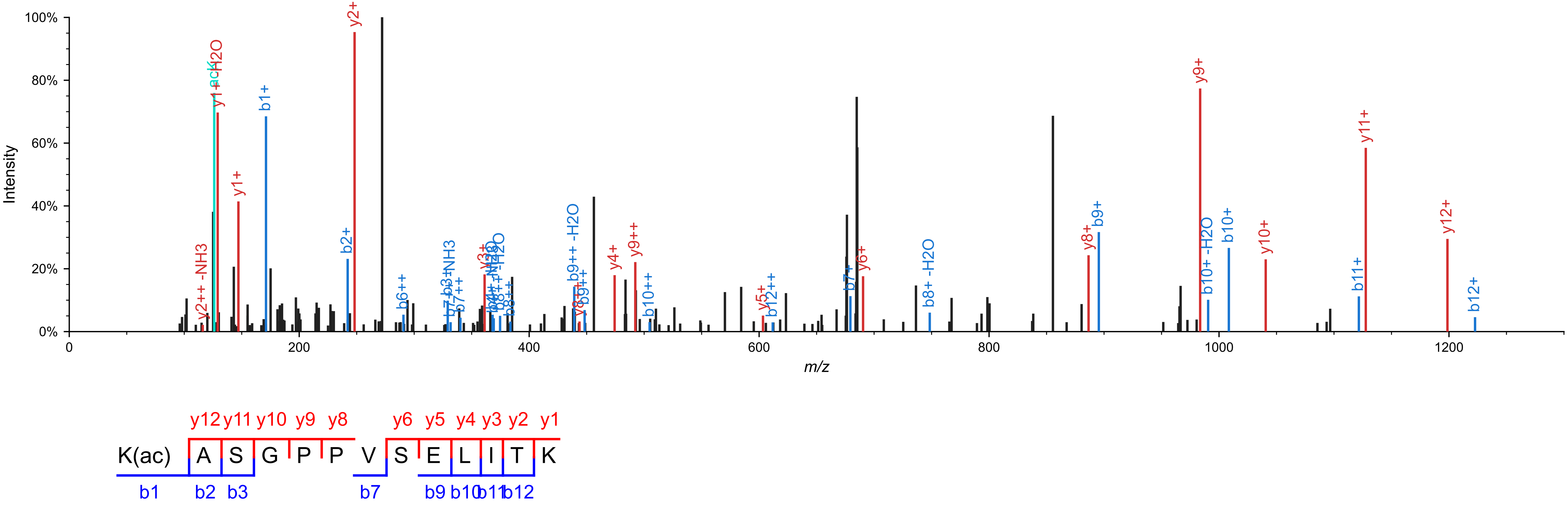

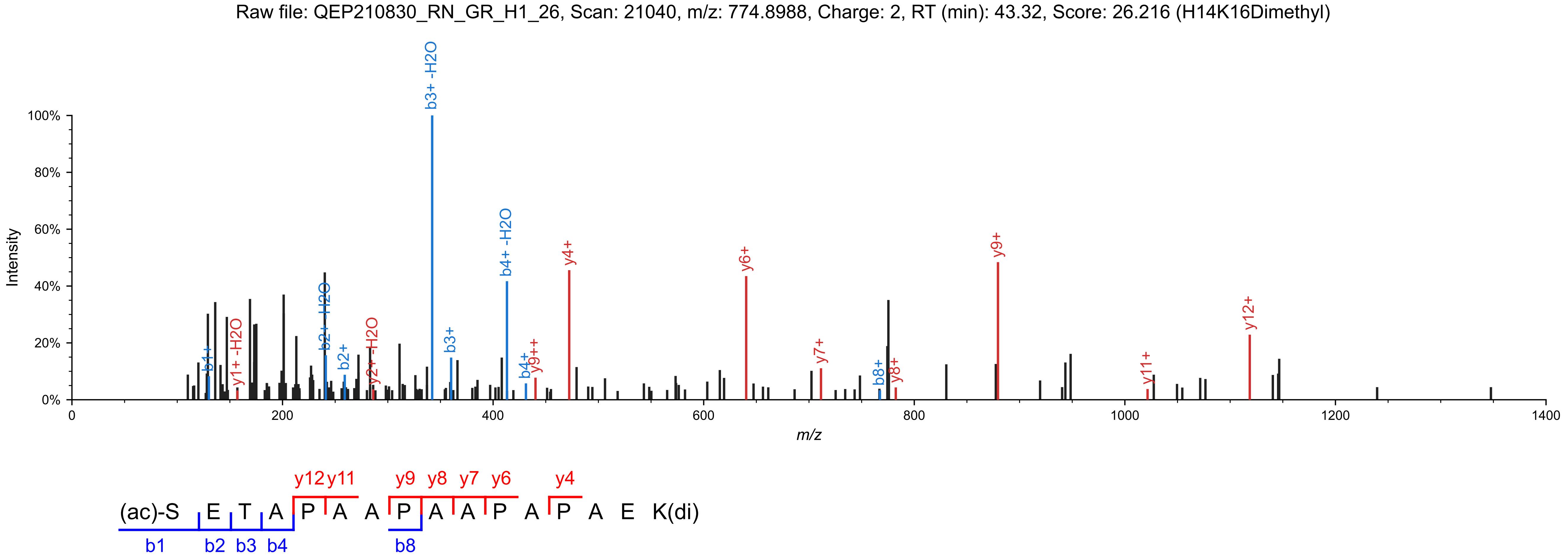
Raw file: QEP210830_RN_GR_H1_26, Scan: 21040, m/z: 774.8988, Charge: 2, RT (min): 43.32, Score: 26.216 (H14K16Dimethyl)
Intensity
m/z
b3+ -H2O
b4+ -H2O
y4+
y6+
y9+
y12+
y11+
y8+
b8+
y7+
b4+
y9++
b3+
b2+
b2+ -H2O
y2+ -H2O
y1+ -H2O
b1+
(ac)-S E T A P A A P A A P A P A E K(di)
y12 y11 y9 y8 y7 y6 y4
b1 b2 b3 b4 b8

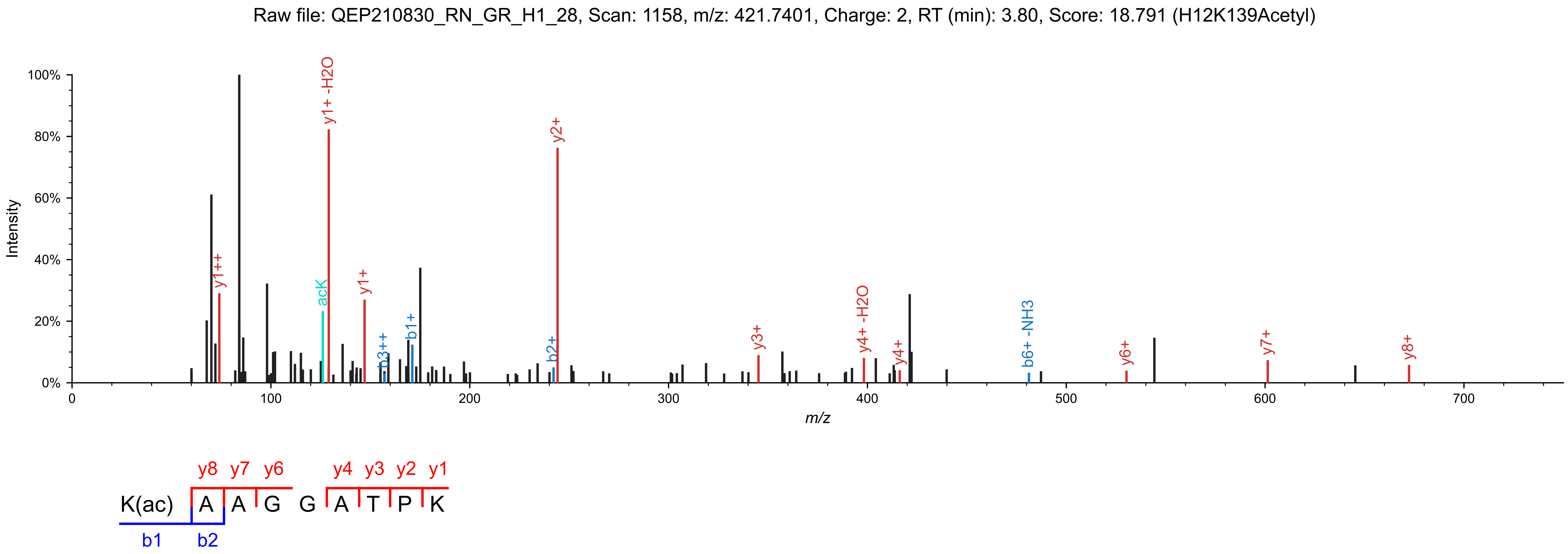
Raw file: QEP210830_RN_GR_H1_28, Scan: 1158, m/z: 421.7401, Charge: 2, RT (min): 3.80, Score: 18.791 (H12K139Acetyl)
Intensity
m/z
y1++
acK
y1+ -H2O
y1+
b3++
b1+
b2+
y2+
y3+
y4+ -H2O
y4+
b6+ -NH3
y6+
y7+
y8+
y8 y7 y6 y4 y3 y2 y1
K(ac) A A G G A T P K
b1 b2

Raw file: QEP210830_RN_GR_H1_28, Scan: 20123, m/z: 508.3109, Charge: 2, RT (min): 41.36, Score: 18.434 (H12K62Acetyl)

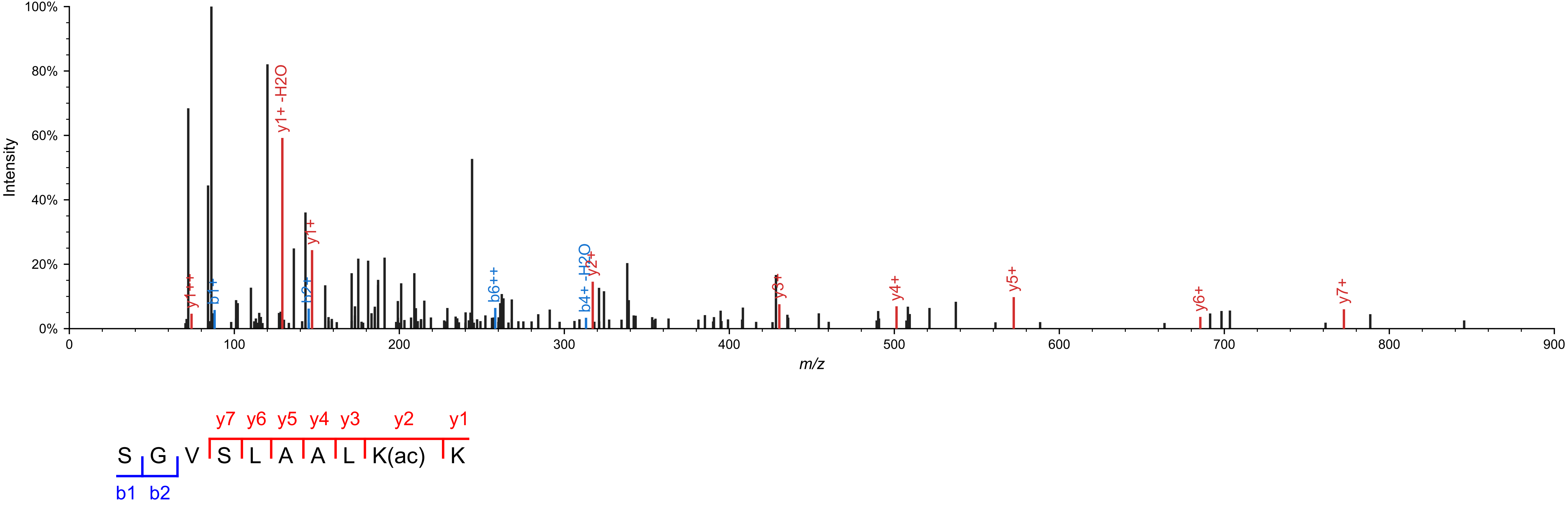

Raw file: QEP210830_RN_GR_H1_28, Scan: 21304, m/z: 774.8988, Charge: 2, RT (min): 43.68, Score: 26.848 (H12K16Dimethyl)

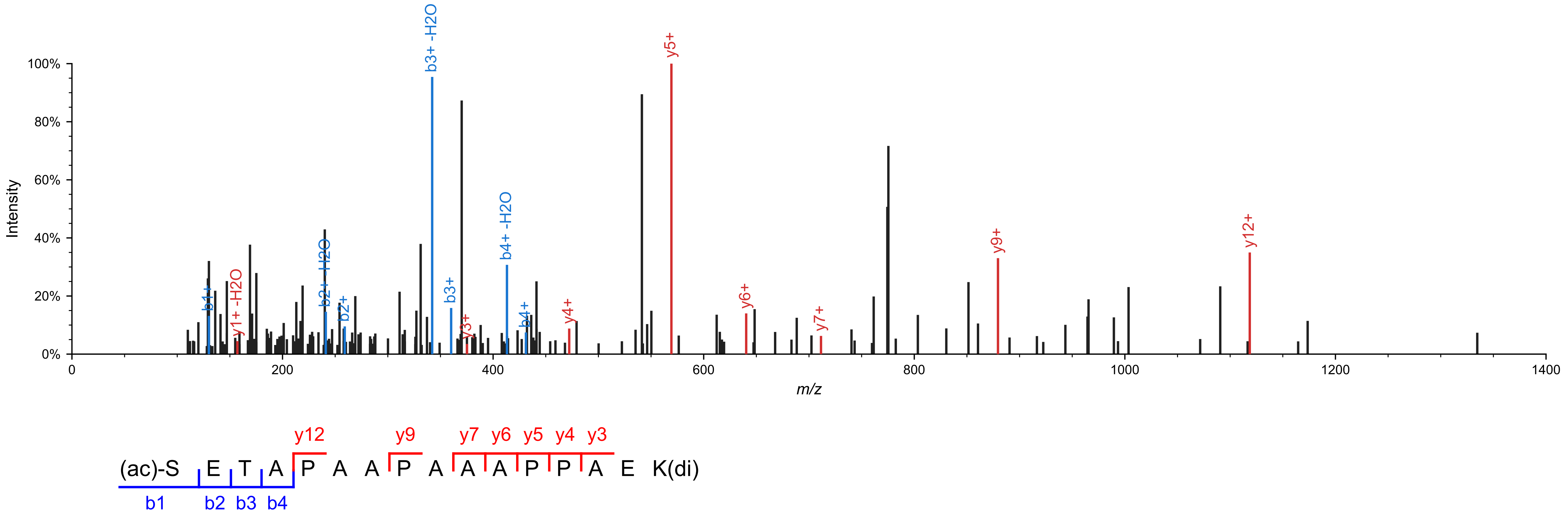

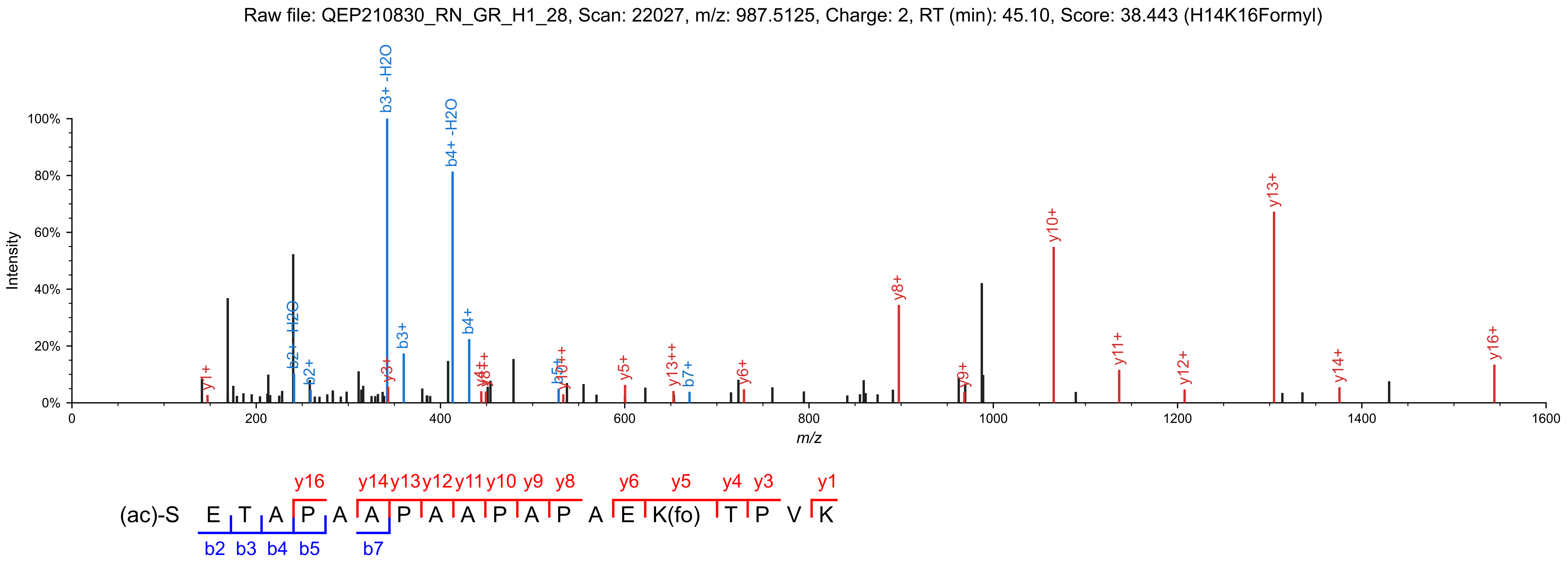
Raw file: QEP210830_RN_GR_H1_28, Scan: 22027, m/z: 987.5125, Charge: 2, RT (min): 45.10, Score: 38.443 (H14K16Formyl)
Intensity
m/z
b3+ -H2O
b4+ -H2O
y13+
y10+
y8+
y16+
y11+
y12+
y14+
b4+
b3+
b2+ -H2O
b2+
y1+
y3+
b5+
y5+
y13++
b7+
y6+
y9+
(ac)-S E T A P A A P A A P A P A E K(fo) T P V K
y16 y14 y13 y12 y11 y10 y9 y8 y6 y5 y4 y3 y1
b2 b3 b4 b5 b7

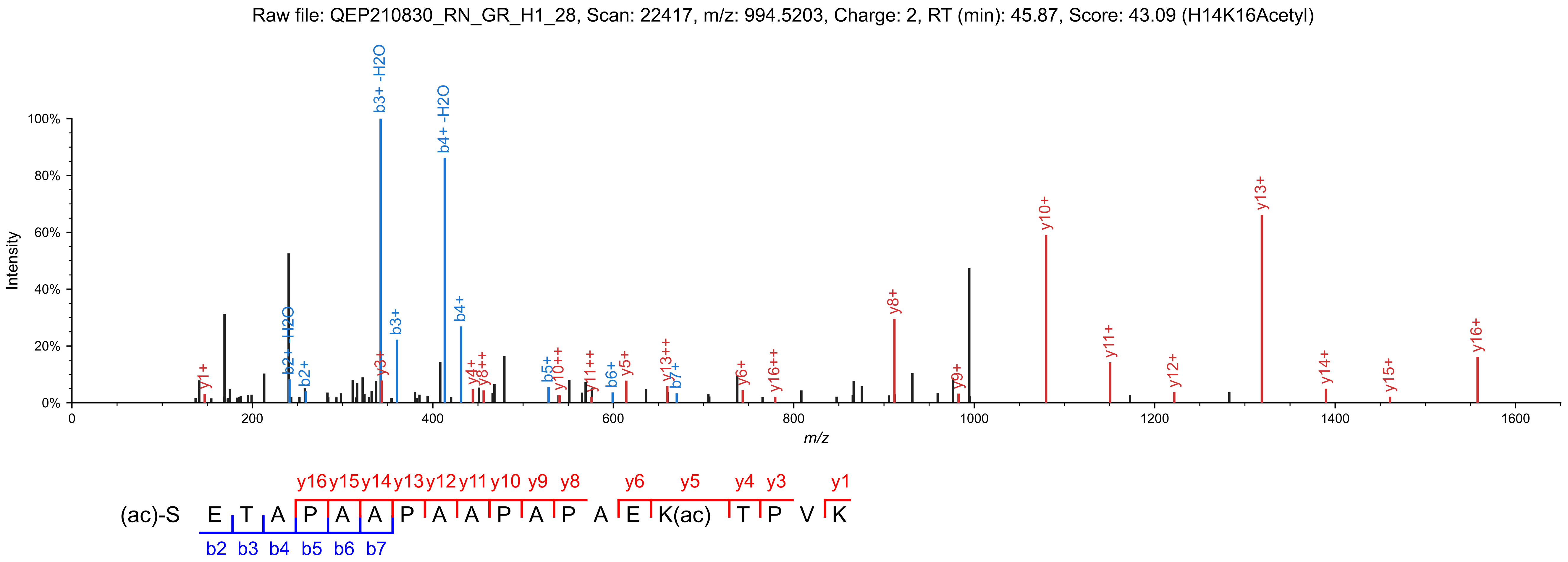
Raw file: QEP210830_RN_GR_H1_28, Scan: 22417, m/z: 994.5203, Charge: 2, RT (min): 45.87, Score: 43.09 (H14K16Acetyl)
Intensity
m/z
b3+ -H2O
b4+ -H2O
y13+
y10+
y8+
b4+
b3+
y16+
y11+
b2+ -H2O
b2+
y1+
y3+
y4+
y8++
b5+
y10++
y11++
b6+
y5+
y13++
b7+
y6+
y16++
y9+
y12+
y14+
y15+
(ac)-S E T A P A A P A A P A P A E K(ac) T P V K
y16 y15 y14 y13 y12 y11 y10 y9 y8 y6 y5 y4 y3 y1
b2 b3 b4 b5 b6 b7

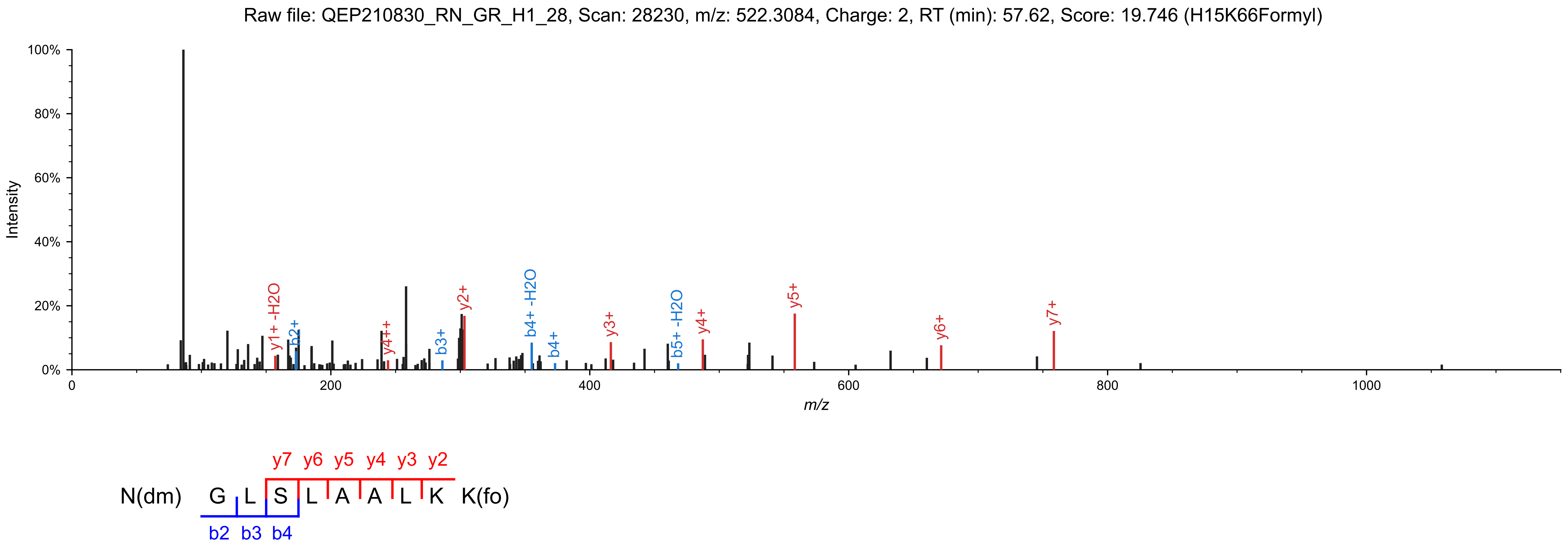

Raw file: QEP210830_RN_GR_H1_28, Scan: 28230, m/z: 522.3084, Charge: 2, RT (min): 57.62, Score: 19.746 (H15K66Formyl)
100%
80%
60%
40%
20%
0%
Intensity
0
200
400
600
800
1000
m/z
y1+ -H2O
b2+
y4++
b3+
y2+
b4+ -H2O
b4+
y3+
b5+ -H2O
y4+
y5+
y6+
y7+
y7 y6 y5 y4 y3 y2
N(dm) G L S L A A L K K(fo)
b2 b3 b4

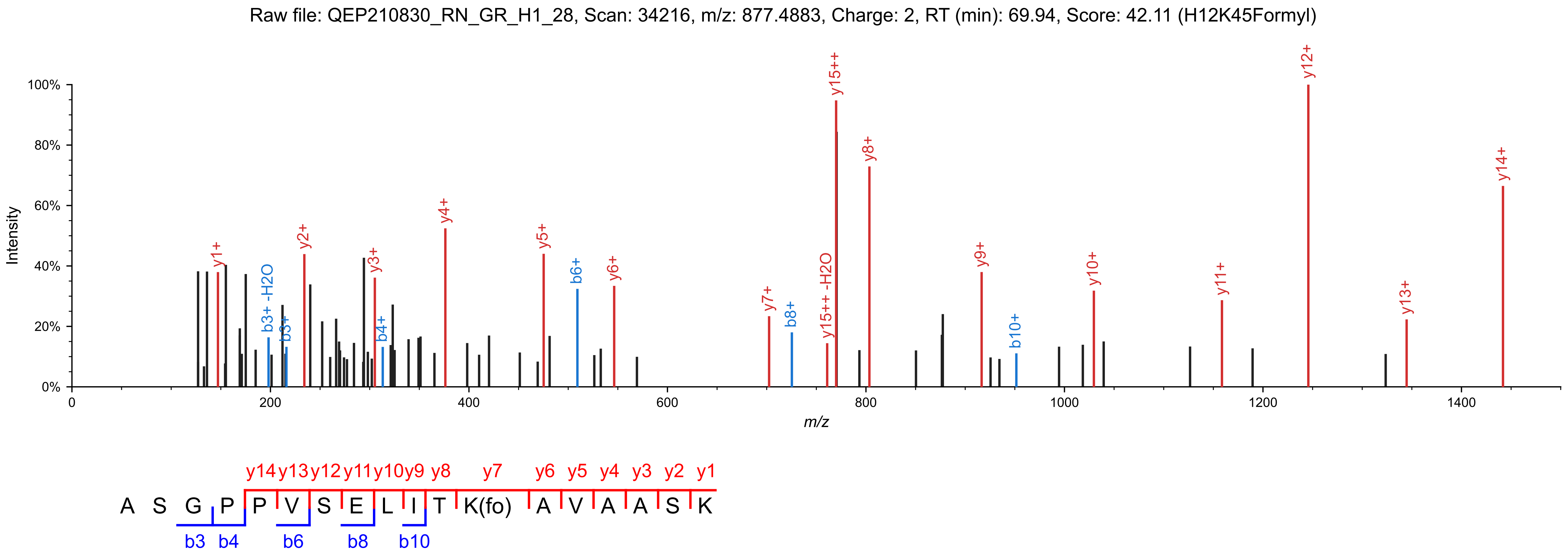

Raw file: QEP210830_RN_GR_H1_28, Scan: 34216, m/z: 877.4883, Charge: 2, RT (min): 69.94, Score: 42.11 (H12K45Formyl)
Intensity
m/z
y1+
b3+ -H2O
b3+
y2+
y3+
b4+
y4+
y5+
b6+
y6+
y7+
b8+
y15++ -H2O
y15++
y8+
y9+
b10+
y10+
y11+
y12+
y13+
y14+
A S G P P V S E L I T K(fo) A V A A S K
y14 y13 y12 y11 y10 y9 y8 y7 y6 y5 y4 y3 y2 y1
b3 b4 b6 b8 b10

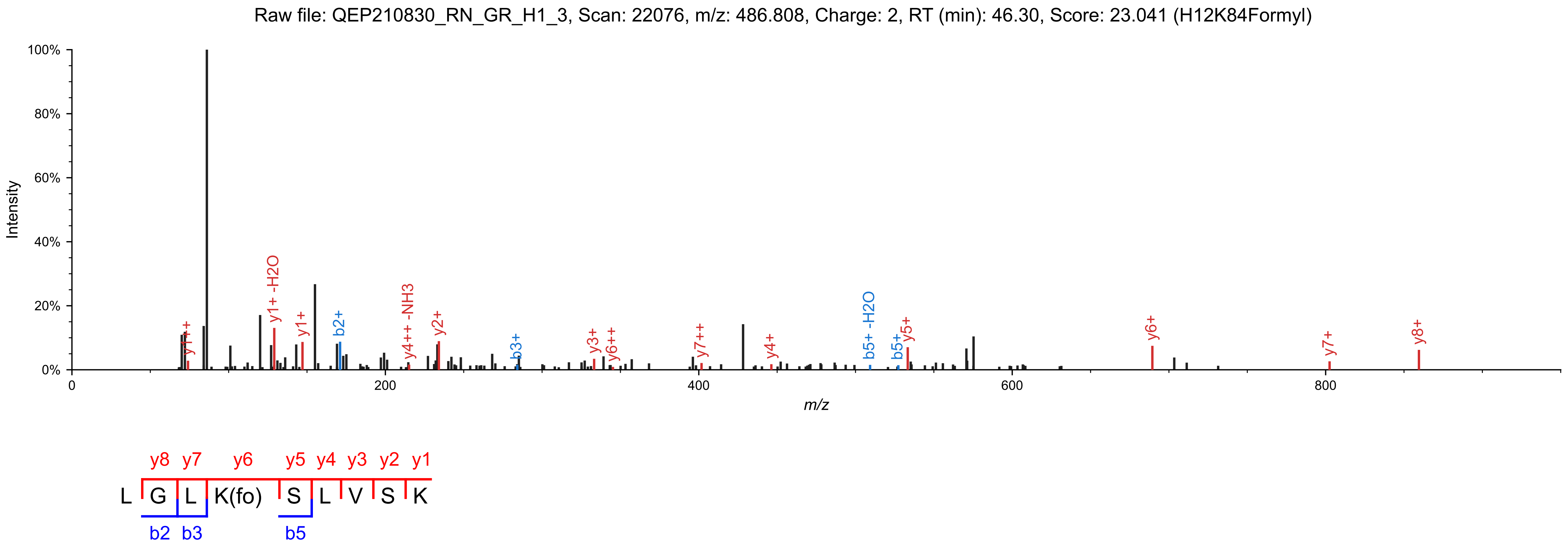

Raw file: QEP210830_RN_GR_H1_3, Scan: 22076, m/z: 486.808, Charge: 2, RT (min): 46.30, Score: 23.041 (H12K84Formyl)
Intensity
m/z
y8 y7 y6 y5 y4 y3 y2 y1
L G L K(fo) S L V S K
b2 b3 b5

Raw file: QEP210830_RN_GR_H1_3, Scan: 29438, m/z: 748.6999, Charge: 3, RT (min): 61.31, Score: 20.613 (H10K26Glutaryl)

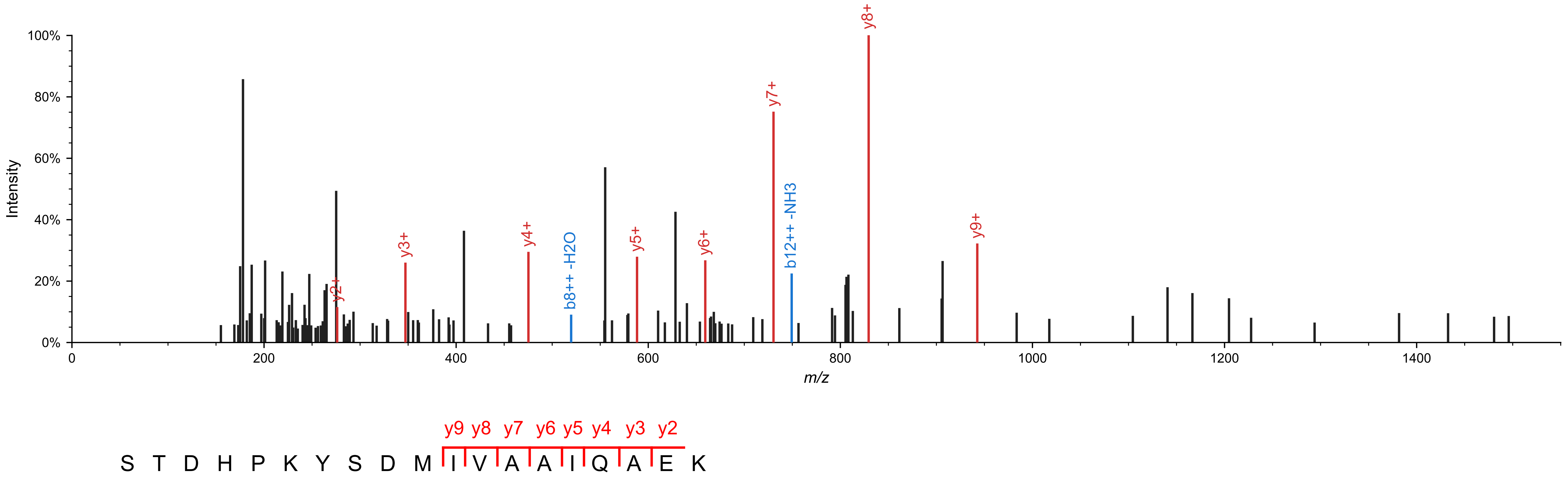

Raw file: QEP210830_RN_GR_H1_30, Scan: 14181, m/z: 639.3404, Charge: 2, RT (min): 30.15, Score: 34.411 (H12K63Acetyl)

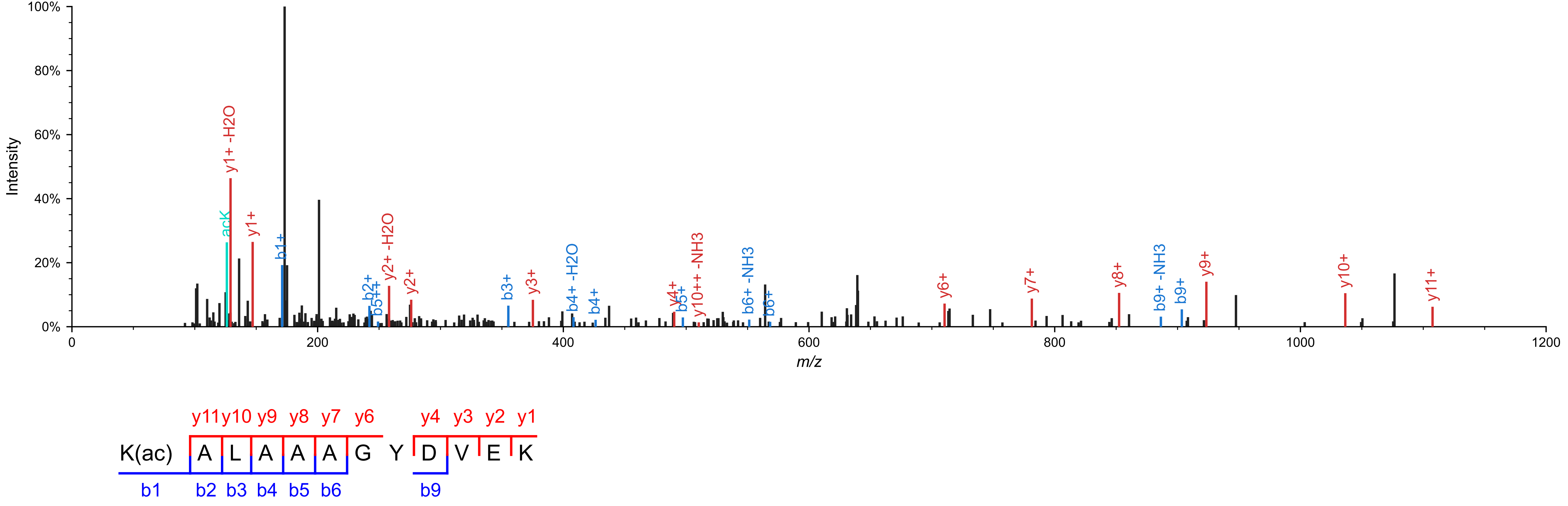

Raw file: QEP210830_RN_GR_H1_30, Scan: 15610, m/z: 783.9097, Charge: 2, RT (min): 32.93, Score: 35.523 (H12K96Formyl)

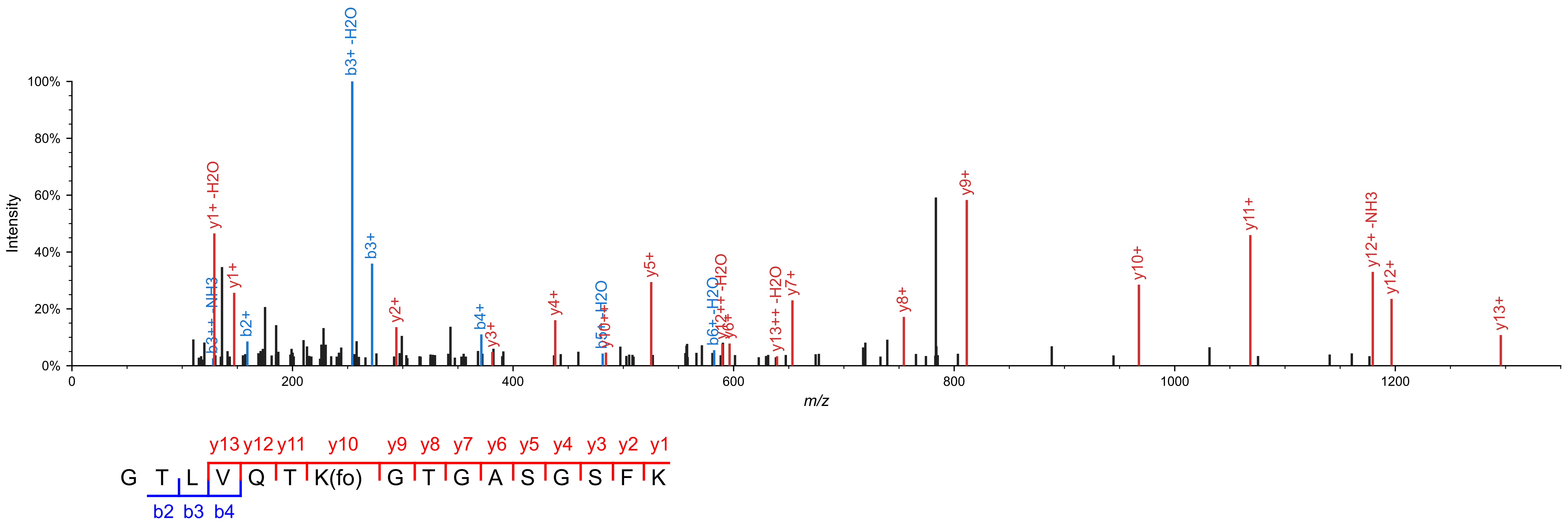

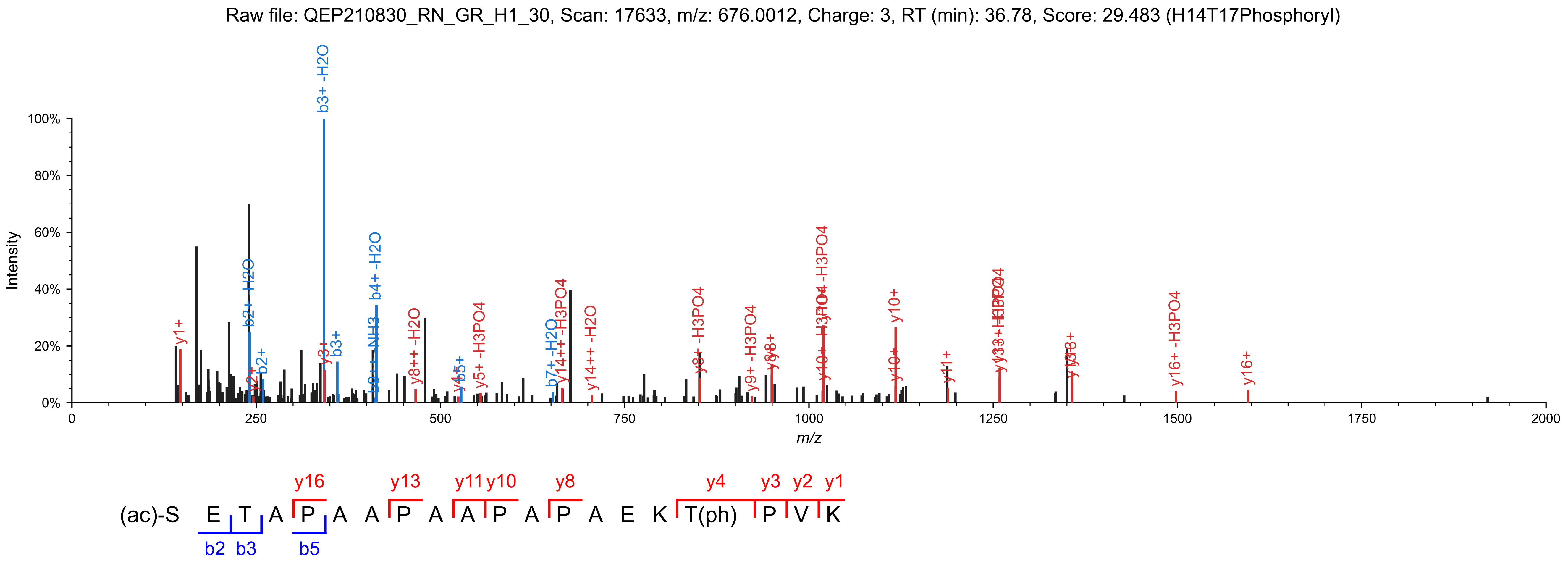

Raw file: QEP210830_RN_GR_H1_30, Scan: 17633, m/z: 676.0012, Charge: 3, RT (min): 36.78, Score: 29.483 (H14T17Phosphoryl)
Intensity
100%
80%
60%
40%
20%
0%
0
250
500
750
1000
1250
1500
1750
2000
m/z
b3+ -H2O
b4+ -H2O
b2+ -H2O
b2+
b3+
y1+
y3+
y8++ -H2O
y5+ -H3PO4
b7+ -H2O
y14++ -H2O
y8+ -H3PO4
y9+ -H3PO4
y10+
y11+
y16+ -H3PO4
y16+
(ac)-S E T A P A A P A A P A P A E K T(ph) P V K
y16
y13
y11
y10
y8
y4
y3
y2
y1
b2
b3
b5

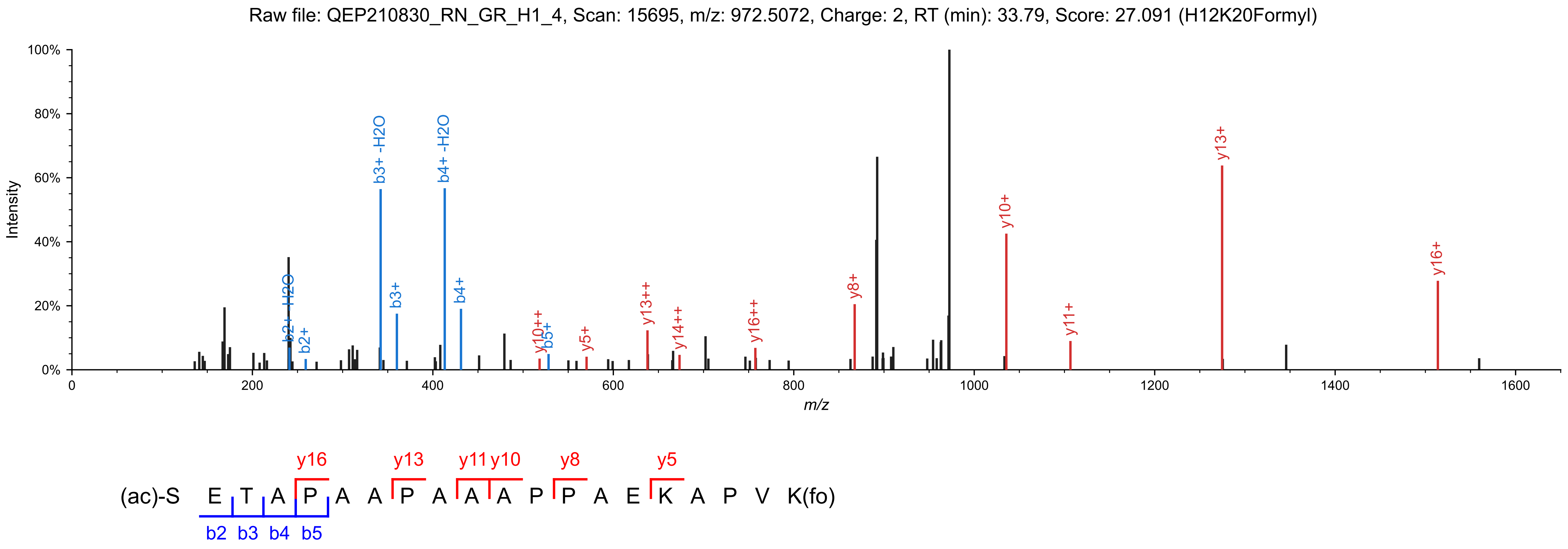

Raw file: QEP210830_RN_GR_H1_4, Scan: 15695, m/z: 972.5072, Charge: 2, RT (min): 33.79, Score: 27.091 (H12K20Formyl)
Intensity
100%
80%
60%
40%
20%
0%
0
200
400
600
800
1000
1200
1400
1600
m/z
b2+ -H2O
b2+
b3+ -H2O
b3+
b4+ -H2O
b4+
b5+
y10++
y5+
y13++
y14++
y16++
y8+
y10+
y11+
y13+
y16+
y16
y13
y11
y10
y8
y5
(ac)-S E T A P A A P A A A P P A E K A P V K(fo)
b2
b3
b4
b5

Raw file: QEP210830_RN_GR_H1_4, Scan: 33151, m/z: 857.9185, Charge: 2, RT (min): 69.26, Score: 30.596 (H13T3Phosphoryl)

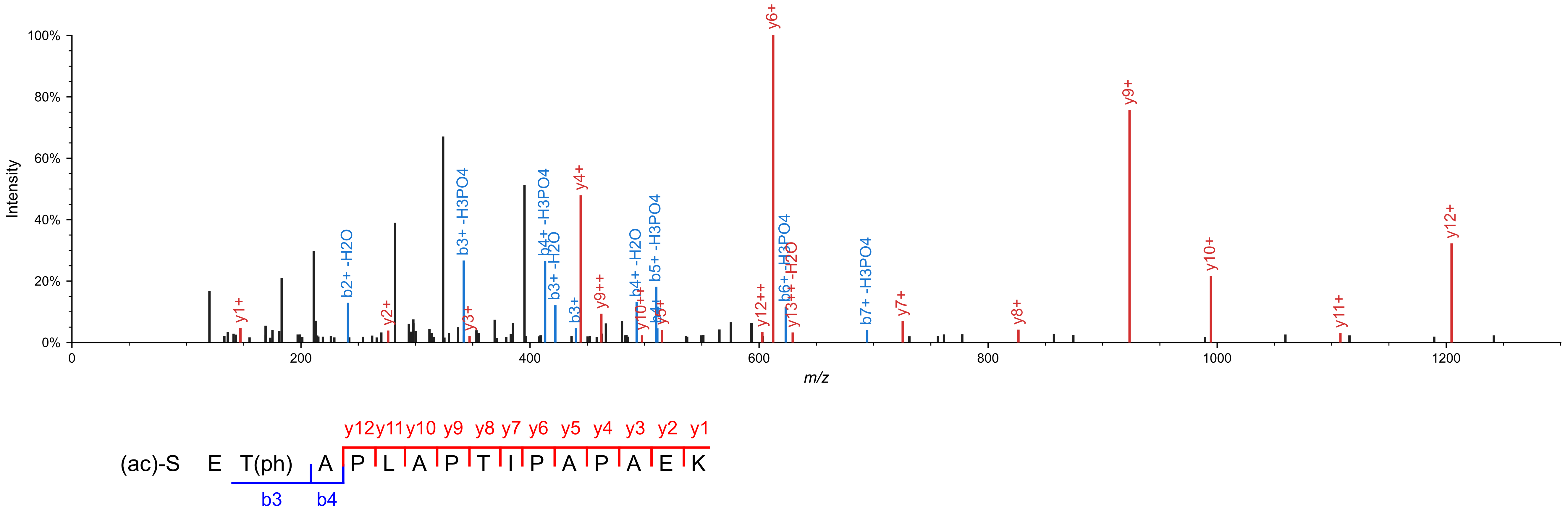

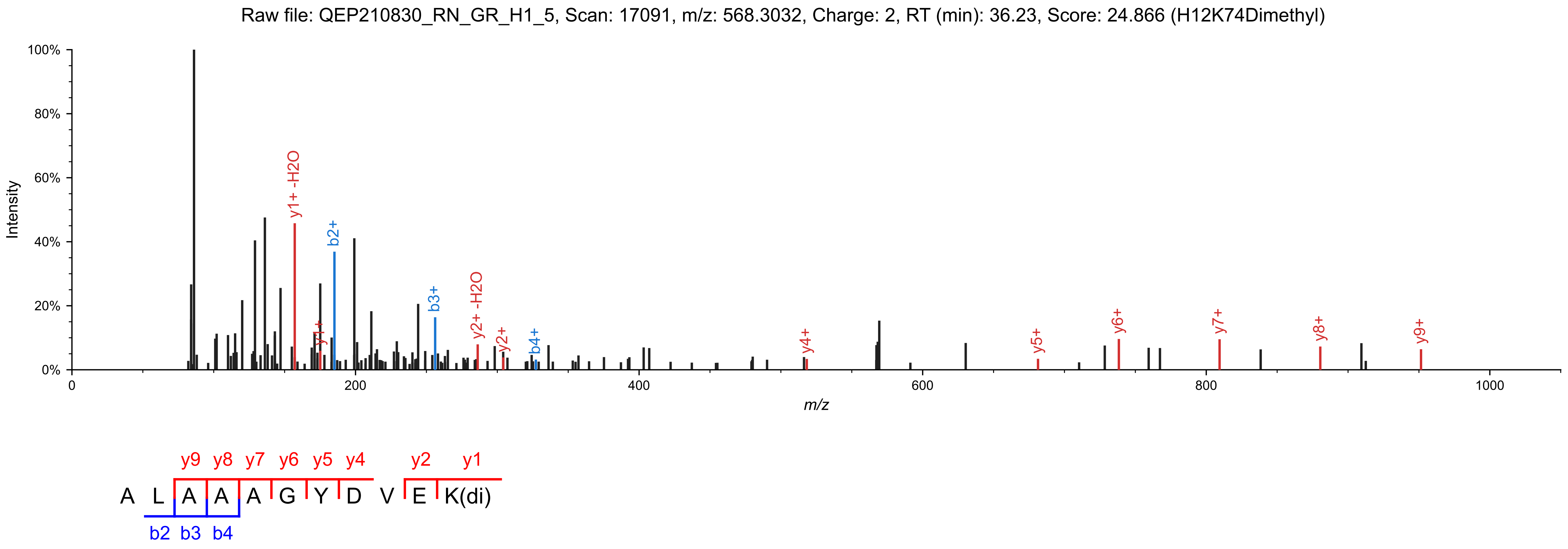
Raw file: QEP210830_RN_GR_H1_5, Scan: 17091, m/z: 568.3032, Charge: 2, RT (min): 36.23, Score: 24.866 (H12K74Dimethyl)
Intensity
100%
80%
60%
40%
20%
0%
0
200
400
600
800
1000
m/z
y1+ -H2O
b2+
b3+
y2+ -H2O
y1+
y2+
b4+
y4+
y5+
y6+
y7+
y8+
y9+
y9 y8 y7 y6 y5 y4 y2 y1
A L A A A G Y D V E K(di)
b2 b3 b4

Raw file: QEP210830_RN_GR_H1_7, Scan: 14648, m/z: 447.5976, Charge: 3, RT (min): 33.81, Score: 26.015 (H12K33Methyl)

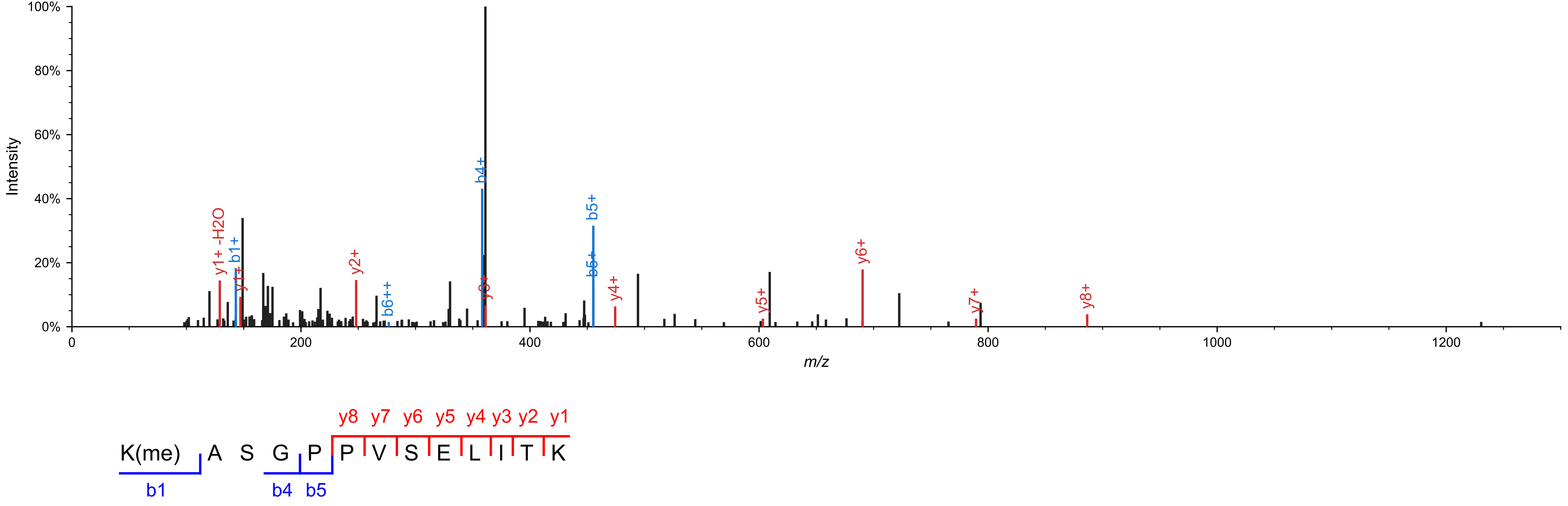

Supplement: Annotated Spectra [file mmc6.pdf]
